# Supplementary material for: Enchained growth and cluster dislocation: A possible mechanism for microbiota homeostasis
Source: PLoS Comput Biol. 2019 May 3;15(5):e1006986. doi: 10.1371/journal.pcbi.1006986 (PMC6519844; doi:10.1371/journal.pcbi.1006986)
Supplement: S1 Text — (PDF) [file pcbi.1006986.s001.pdf]

Appendix: Enchained growth and cluster dislocation: a possible mechanism for microbiota homeostasis

F Bansept, K Schumann-Moor, M Diard, W-D Hardt, E Slack, C Loverdo

A Order of magnitude of the encounter time between two bacteria

The typical time to find one target of radius a in a sphere of radius b by diffusion is of the order of $b^3/(Da)$, so the typical time when there are N bacteria in a volume V is of the order of $V/(NDa)$. For bacteria, a is in the micrometer range. Bacteria such as salmonella or E.coli typically swim at $10\mu m/s$, and change direction every second, which gives a diffusion coefficient of the order of $10^{-10}m^2/s$ [1, 2, 3] (The peristaltic motions of the digesta are large scale movement rather than local diffusion, so we assume they have a smaller effect on diffusion). The mouse's cecum has a volume of the order of (1cm)^3. In experiments of [4], the smallest inoculum consists in $N = 10^5$ bacteria, which is already large compared to what could be a realistic number of pathogenic bacteria in food poisoning (10^5 is the typical number of *Salmonella* for food poisoning in humans [5], which are much larger than mice). With these numbers, the typical encounter time is of the order of $10^5 s$, i.e 30h, about 10 times longer than the typical digestion time in mice.

B Argument for high enchainment probability upon replication

When a bacterium replicates, the time for septation is of the order of a few minutes. We intuitively think that this time is much larger than the time τ_k required for bacteria to stick together when they randomly meet. The aim of this section is to check this intuition by giving an overestimate of τ_k.

If the diffusion coefficient is high enough, the time for bacteria to stick to each other will be limited by which proportion of the time they spend in close vicinity, and the rate k at which bacteria stick to each other when they are in close vicinity, k being the inverse of τ_k. If the diffusion coefficient is smaller, then the time to first encounter will also play a role, but as we calculate an overestimate of τ_k, we can neglect this scenario.

We use the data on figure 1k of [4] about non-dividing bacteria (so the only sticking is from random encounters). The majority of them are aggregated

after τ_{exp} up to 8 hours (from the inoculum ingestion to the sampling used for imaging) for a concentration of $10^7 - 10^8$ bacteria. As we will see, this estimate of τ_k is proportional to τ_{exp} and N, so to be conservative, as we will calculate an overestimate of τ_k, we take the highest concentration and the maximum experimental time, i.e. $N = 10^8$ bacteria in $V = 1cm^3$ (cecum volume) and $\tau_{exp} = 8$ hour.

The bacteria typical size is a few micrometers, we thus take $3\mu m$ as an overestimate of the maximum bacterial size. Thus to be in close contact, two bacteria must be at most at $a = 3\mu m$ away. Let us assume that then, the volume of possible contact is $4/3\pi a^3$, which is also an overestimate, because only certain orientations will allow bacteria to touch each other. Then, the proportion of time spent in close contact will be of the order of $(N4\pi a^3)/(3V)$. Then the typical time to stick to each other will be $\tau_{exp} = \tau_k 3V/(N4\pi a^3)$. Then $\tau_k = \tau_{exp} N4\pi a^3/(3V)$. Numerically, we obtain about 5 minutes as an overestimate of τ_k.

Note that this is a large overestimate. Indeed, when bacteria get clumped to each other, their effective concentration decreases, thus it takes longer for the last bacteria to meet others, and thus the time for most bacteria to be clumped will be significantly larger than the inverse of the early clumping rate.

With all these highly conservative estimates, we find τ_k at the very most of the same order of magnitude as the septation time, and very likely much smaller. Hence the probability for bacteria to escape enchainment is small, which justifies that we take in general the limit of no escape.

C Model with bacterial escape ($\delta > 0$) and differential loss ($c \neq c'$).

Figure A shows how the growth rate depends on r for different δ, δ', δ'', c and c'.

Our numerical study of the system showed us that there is some critical value δ_c below which the behavior is qualitatively similar to the behavior of the system with $\delta = 0$, i.e. with a finite maximum of the growth rate of the free bacteria as a function of the replication rate; and above which the growth rate continues to increase with replication rate. Actually, for $\delta > 0.5$, the growth rate necessarily continues to increase with the replication rate. Indeed, upon replication, one free bacteria becomes two daughter bacteria, an average of 2δ of them staying free. Thus the net gain in free bacteria is $2\delta - 1$. Thus for $\delta > 0.5$, the growth rate of free bacteria is at minimum $r(2\delta - 1)$. Consequently, $\delta_c \leq 0.5$.

We detail here how to obtain the approximation for the chain length distribution. In the long time limit, the number of chains of length i is of the order of $Cp_i \exp(\lambda t)$, with λ the largest eigenvalue. Equation (8) of main text simplifies to:

$$\lambda p_i = r(2\delta' - i)p_i + rp_{i-1}(i - 1 - 2\delta' + 3\delta'' - i\delta'') - (i-1)p_i\alpha + 2\alpha p_{i+1} - c'p_i$$

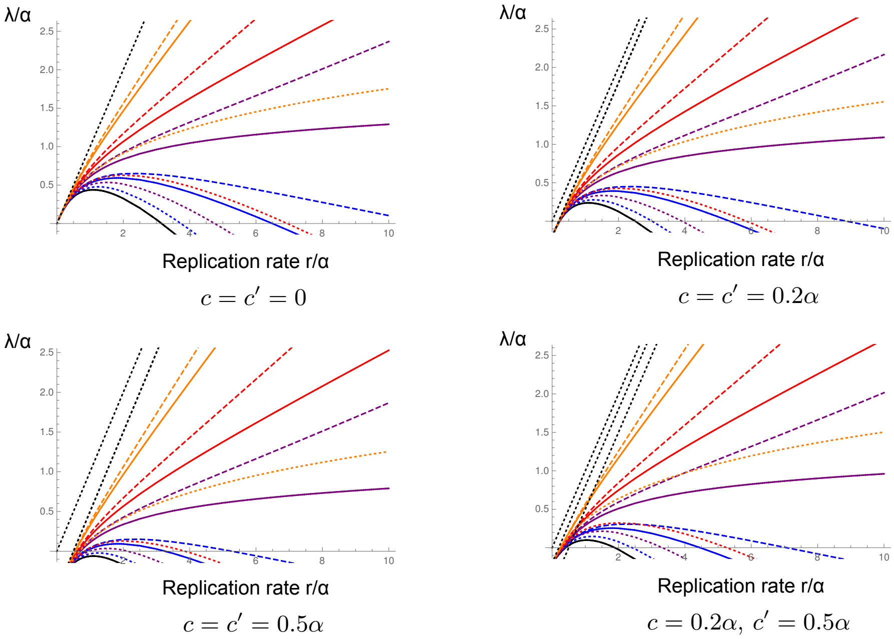

Figure A: Growth rate λ as a function of the replication rate r, both in units of α. Numerical results (colors), with $\delta = \delta' = \delta''$ (solid lines), $\delta = \delta'$, and $\delta'' = 0$ (dashed lines), $\delta' = \delta'' = 0$ (dotted lines). $\delta = 0$, 0.1, 0.2, 0.3, 0.5. Note that for $\delta = 0$, solid, dashed and dotted lines collapse, as expected. The black dotted lines are either r/α, $(r - c)/\alpha$ or $(r - c')/\alpha$. As expected, if $c = c'$, the resulting growth rate are the same than when $c = c' = 0$, minus c. If $c \neq c'$, the results are closer for small r/α to the results if both c and c' had the c value. For the numerical results, $n_{max} = 40$.

Assuming that i is large,

$$p_i \simeq (1 - \delta'')\frac{r}{r + \alpha} p_{i-1}$$

is required. Using this approximation for all i, the proportion of chains of length k is:

$$p_k = \left(1 - (1 - \delta'')\frac{r}{r + \alpha}\right)\left((1 - \delta'')\frac{r}{r + \alpha}\right)^{k-1}$$

Free bacteria are released at a rate $2r\delta' + 2\alpha$ per chain. This rate is independent of the chain length. The direct contributions to the increase of free bacteria from chains of length i compared to all the larger chains will be (with $K = (1 - \delta'')r/(r + \alpha)$):

$$\frac{contribution\ larger}{contribution\ i} = \frac{(2r\delta' + 2\alpha)\sum_{j=i+1}^{\infty}(1 - K)K^j}{(2r\delta' + 2\alpha)(1 - K)K^i} = \sum_{j=1}^{\infty} K^j = \frac{K}{1 - K} = \frac{(1 - \delta'')r}{\alpha + r\delta''}$$

If r is small compared to α (replication rate $\ll$ breaking rate), then this ratio is small. Thus the larger chains are quickly negligible. Indeed, in this regime, chains typically dislocate before new replications, so there are few larger chains.

Figures B, C, D, E show how the chain length distribution depends on δ, δ', δ'', c and c'.

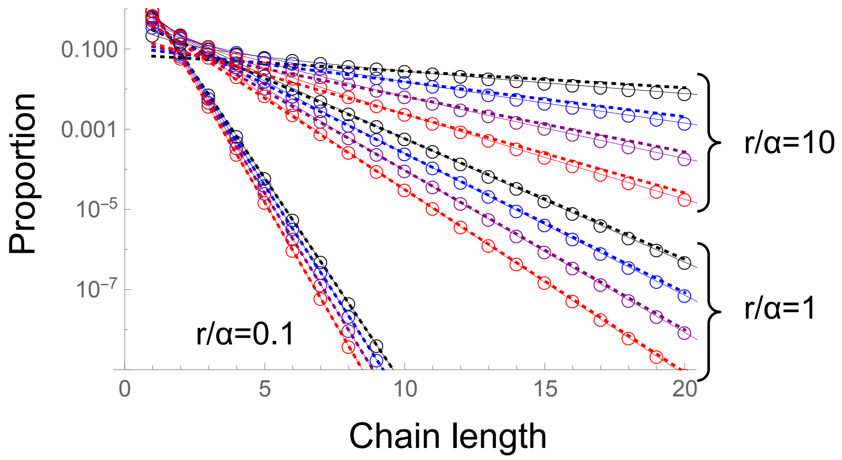

Figure B: Chain length distribution. All as in main figure 2D (model with bacterial escape) (numerical results: points linked by solid lines), except that the approximation (9) of main text (dotted lines) is rescaled by the numerical value at $n = 10$, i.e. instead of representing $\log(p_{i,approx})$, what is represented is $\log(p_{i,approx} * p_{10,numeric}/p_{10,approx}) = \log(p_{i,approx}) + \log(p_{10,numeric}/p_{10,approx})$. This shows that the approximation captures well the length distribution of large chains. We do this because the base for the analytical approximation is the ratio p_{i+1}/p_i, for which we get a limit expression valid for large i. To calculate the whole distribution p_i, we assume that this limit expression for the ratio is valid for any i, whereas this will not be the case for small i. If the limit expression for p_{i+1}/p_i is correct for large i but not for small i, the slope in log scale plot will be correct, but with some offset dependent on how wrong we got the small i case. Making this renormalization enables to check more easily whether the slope is correct. $\delta = \delta' = \delta'' = 0$ (black), 0.1 (blue), 0.2 (purple), 0.3 (red). $n_{max} = 40$.

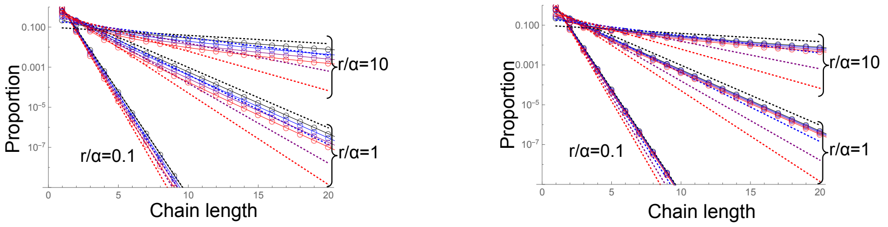

$\delta = \delta' \geq 0$, $\delta'' = 0$ $\delta \geq 0$, $\delta' = \delta'' = 0$

Figure C: Chain length distribution. All as in figure 2D (model with bacterial escape) (numerical results: points linked by solid lines), except the values of δ' and δ'': $\delta = 0$, 0.1, 0.2, 0.3. Dotted lines: approximation (5) of main text. Approximation (9) of the main text predicts that the distribution should depend only on δ'', and not δ nor δ'. In these figure where $\delta'' = 0$ but δ (and in the left pannel δ') have non-zero values, we do observe that the distribution, in particular its slope, is closest to the result for $\delta = \delta' = \delta'' = 0$. $c = c' = 0$, $n_{max=40}$.

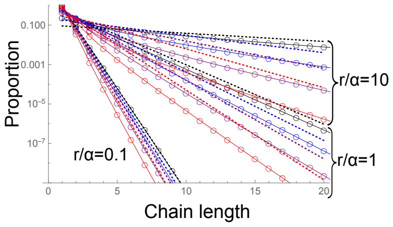

Dotted lines : approximation (9) of main text

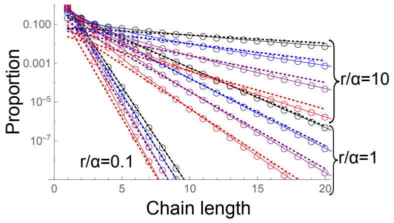

Dotted lines: approximation (9) of main text, rescaled by the numerical value at $n = 10$ similarly to figure B.

Figure D: Chain length distribution, for $\delta = \delta' = 2\delta''$. Other parameters as in figure 2D of main text : $\delta = 0$, 0.1, 0.2, 0.3. $c = c' = 0$, $n_{max} = 40$. Dotted lines: approximation (9) of main text. The approximation does not work as well as when $\delta = \delta' = \delta''$.

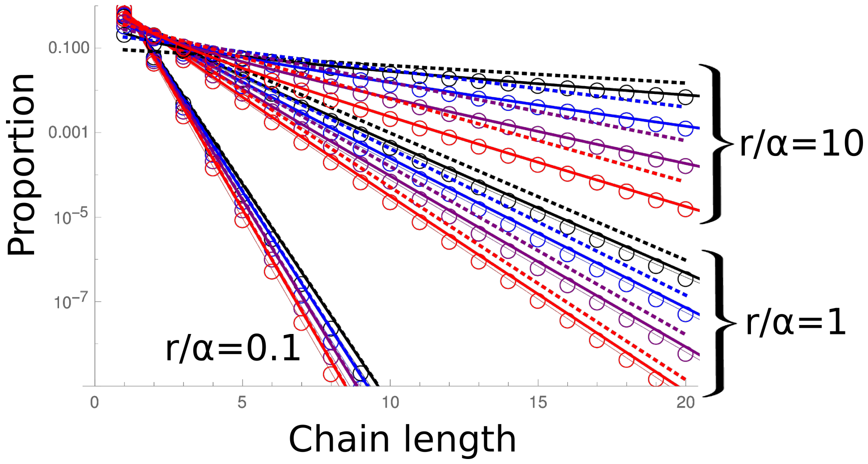

Figure E: Chain length distribution. Similar to figure 2D of main text. $\delta = \delta' = \delta'' =$ 0, 0.1, 0.2, 0.3. Thick solid lines with no markers are for $c = c' = 0$, Thin solid lines with $\circ$ are for $c = 0.2\alpha$, $c' = 0.5\alpha$. Dotted lines: approximation (9) of main text. There is very little change in the chain length distribution. $n_{max} = 40$.

D Chain length distribution with a fixed replication time - approximation

Below, we present in details the assumptions and calculations to obtain the approximation of the chain length distribution when bacteria replicate every τ.

We define $n_i(t)$ the number of chains of length i at t with t taken just before a replication. Assuming i even,

$$n_i(t+\tau) = \sum_{j=0}^{\infty} n_{i/2+j}(t) l(i+2j, 2j, \tau).$$

This is because just before a replication, there are $n_{i/2+j}(t)$ chains of length $i/2+j$. Then, just after the replication, these chains are of length $i+2j$. Time $t+\tau$ is just before the next replication. With probability $l(i+2j, 2j, \tau)$, these chains of length $i+2j$ have lost $2j$ bacteria on their edges and are now chains of length i. We sum over all the possible j. In the long time, $n_i(t) = Cp_i \exp(\lambda t)$, with λ the long term growth rate, that is such that $\exp(\lambda\tau) = \mathcal{N}$, with $\mathcal{N}$ the largest eigenvalue of the matrix. Replacing $l(i+2j, 2j, \tau)$ by its expression as in equation (11) of the main text, the previous equation leads to:

$$\mathcal{N} p_i = \sum_{j=0}^{\infty} p_{\frac{i}{2}+j} e^{-\alpha\tau(i-1+2j)} (e^{\alpha\tau} - 1)^{2j} \frac{2^{2j}}{(2j)!}.$$

We compare the 1st term of the sum to the rest of the sum. The first term is$p_{\frac{i}{2}} e^{-\alpha\tau(i-1)}$, the rest of the sum is:

$$\sum_{j=1}^{\infty} p_{\frac{i}{2}+j} e^{-\alpha\tau(i-1+2j)} (e^{\alpha\tau} - 1)^{2j} \frac{2^{2j}}{(2j)!}.$$

We divide both by $e^{-\alpha\tau(i-1)}$. Then this is equivalent of comparing $p_{i/2}$ with:

$$S = \sum_{j=1}^{\infty} p_{\frac{i}{2}+j} e^{-2j\alpha\tau} (e^{\alpha\tau} - 1)^{2j} \frac{2^{2j}}{(2j)!}.$$

When $\alpha\tau$ is large, links typically break before the next replication, so there is little cluster formation, and it is thus expected that the chain length distribution decreases fast with i, so that for $j > 0$, $p_{\frac{i}{2}+j} \ll p_{i/2}$. When $\alpha\tau$ is small, replication is fast compared to the typical time for one link to break. However, for a chain of length $i/2$, τ has to be compared to $(i/2-1)/\alpha$, the typical first link breaking time, thus we expect n_i to decrease with i for i large enough, thus $p_{\frac{i}{2}+j} \lesssim p_{i/2}$ for $j > 0$. We define B such as $p_{\frac{i}{2}+j} \leq B, \forall j > 0$. For $\alpha\tau$ large, $B \ll p_{i/2}$, and for $\alpha\tau$ small, if i is large enough, $B \lesssim p_{i/2}$. Then:

$$S \leq \sum_{j=1}^{\infty} B(1 - e^{-\alpha\tau})^{2j} \frac{2^{2j}}{(2j)!} = B\left(cosh\left(2(1 - \exp(-\alpha\tau))\right) - 1\right)$$

For $\alpha\tau$ large, $\left(cosh\left(2(1 - \exp(-\alpha\tau))\right) - 1\right) \simeq cosh(2) - 1 \simeq 2.7$. For $\alpha\tau$ small, $\left(cosh\left(2(1 - \exp(-\alpha\tau))\right) - 1\right) \simeq 2(\alpha\tau)^2 \ll 1$.

Thus in the case of $\alpha\tau$ large, S is small relative to $p_{i/2}$ because S is smaller than a few units times B, with B much smaller than $p_{i/2}$. In the case of $\alpha\tau$ small, S is small relative to $p_{i/2}$ because S is of the order of $(\alpha\tau)^2 B$, with B of the order of $p_{i/2}$. Then this justifies the assumption that only the first term of the sum matters:

$$p_i \simeq \frac{1}{\mathcal{N}} p_{i/2} \exp\left(-\alpha\tau\,(i-1)\right).$$

We assume $i = 2^k$, with k an integer. This is obviously true only for a very restricted set of i, but however this still yields an approximation for how the distribution depends on i for large i. Then, by recursion,

$$p_i \simeq \frac{1}{\mathcal{N}^k} p_1 \exp\left(-\alpha\tau\left(i(1+1/2+1/2^2+...+1/2^{k-1})-k\right)\right).$$

If i is large enough, $1+1/2+1/2^2+...+1/2^{k-1} \simeq 2$. Remembering that k was defined as $i = 2^k$, the result is:

$$p_i \simeq p_1 i^{\frac{\alpha\tau - \log(\mathcal{N})}{\log(2)}} \exp\left(-2\alpha\tau i\right).$$

When $\alpha\tau \gg 1$, links typically break before the next replication, thus there is little impact of the clustering on the growth. Consequently, the growth will be close to its value in the absence of clustering, i.e. doubling every τ, and thus in this limit $\mathcal{N} = 2$:

$$p_i \simeq p_1 i^{\frac{\alpha\tau}{\log(2)}-1} \exp\left(-2\alpha\tau i\right).$$

This rough approximation allows to explain the core of the observed distribution.

E Model with force-dependent breaking rate

E.1 Model and equations

A link between bacteria may consist of several sIgA bonds, and the number of bound sIgA may not be exactly the same from one inter-bacteria link to the next, but as sIgA are likely well mixed, many per bacteria and that bacteria are similar to each other, let us assume that link heterogeneity is negligible. The links could break if there was some process degrading the sIgA, but the sIgA are thought to be very stable[6]. Another possible explanation for link breaking is that the antigen get extracted from the bacterial membrane, at a rate which may depend exponentially with the force applied on the link[7][8]. If the forces are produced by the bacteria themselves (such as by flagella rotation), there are likely to fluctuate on timescales which are short compared to the time between two bacterial replications, and their distribution is likely to be the same for all links, so it would be appropriate to model their effect as a fixed breaking rate, the same for all the links. Another force is the hydrodynamical force exerted by the flow on the bacterial chain.

The flow in the digestive system is complex and not precisely characterized. Longer bacterial chains may also bend and their shape have complex interactions with the flow. Here, we present the simplest model taking into account the forces exerted by the flow on the link breaking rate. We aim to capture the main plausible effects of the flow when the link breaking rate is force-dependent.

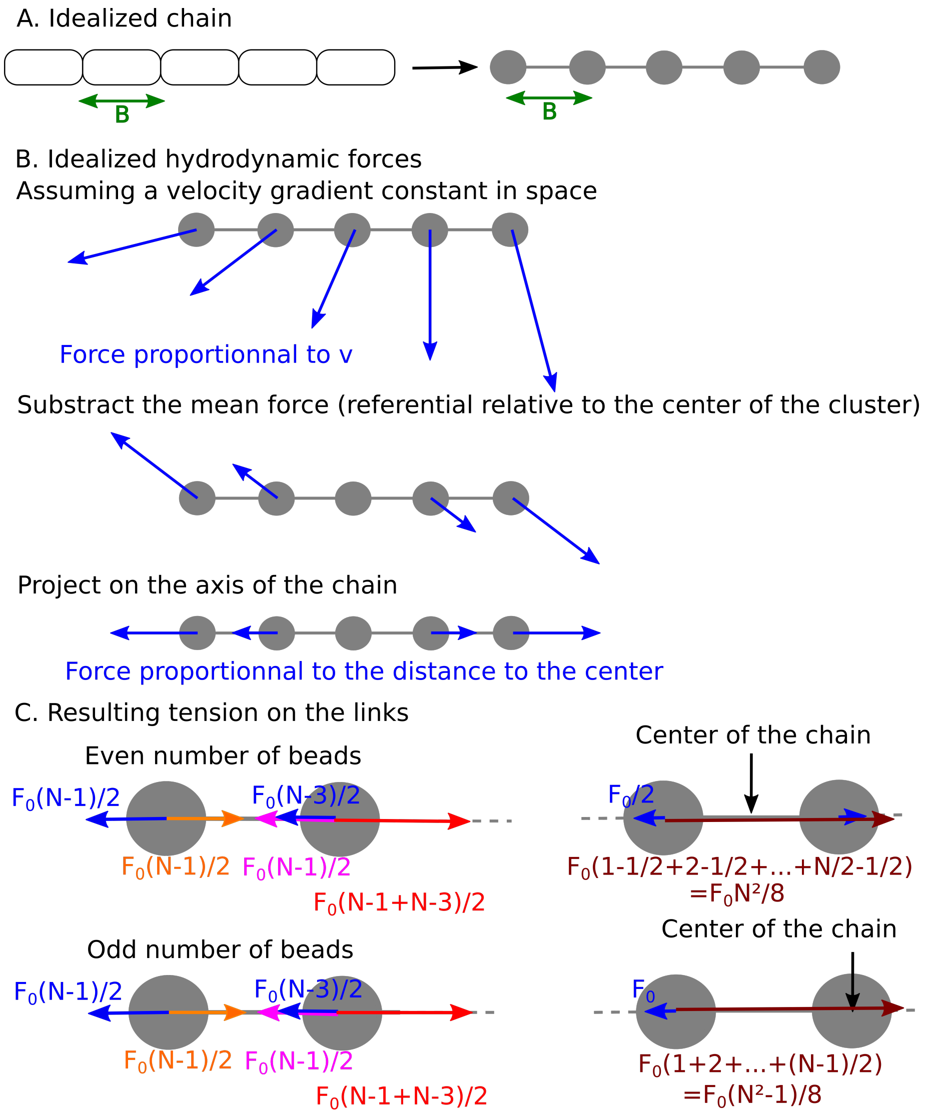

Figure F: Schematic of the forces applied to the chain. **A** We assume a straight chain of beads with no hydrodynamic interactions between them. **B** We substract the average force to put ourselves in the referential of the center of the chain, as the total force will translate the whole chain and not impact on the forces on the links. We focus on the forces parallel to the chain that will impact the tension between the links. **C** Sum of the forces on each bead, for chains with even and odd number of beads.

Let us take a linear chain of N bacteria, each of length B. Let us approximate it by a rigid chain with beads linked by straight rods of length B (pannel A of figure F). Let us assume that the rods are infinitely thin so they do not interact with the flow, and let us neglect the hydrodynamical interaction between the beads, so they each are subject to the same frictional force for a given fluid

velocity, and, given that the typical Reynolds numbers in the digestive tract are relatively low[9], then the viscous force on each bead is proportional to the flow velocity.

Then, let us assume that the velocity gradient in the fluid is constant around the chain. The rationale for this approximation is that the typical scales of the flow are of the order of the centimeter / millimeter (for instance in a mouse, the cecum typical size is in the cm range), much larger than typical bacterial chains (the length of one bacteria is about $2\mu m$, so even chains of dozens of bacteria remain small compared to the typical flow scale), thus we take a linear approximation of the velocity field in the vicinity of a bacterial chain.

Then, if we take the sum of the forces on the whole chain, it will be equal on mN multiplied by the acceleration of the center of mass of the chain, with m the mass of each bead. When all the beads move together, there is no force on the links, thus let us take the referential relative to the center of the chain, and subtract the mean force on each bead (panel B of figure F). Then, there remain forces perpendicular to the axis of the chains, and forces parallel to the axis of the chain. The forces perpendicular to the axis of the chain will make it rotate, and as they are perpendicular, they have no effect on the tension on the rods. Then, let us consider only the forces parallel to the chain.

In the example portrayed here, the chain is elongated. The reverse could happen, but in this case, the chain would likely buckle, and the force applied on the links would be small. The flow varies considerably in time, due to peristaltic motions[10][9]. There would be moments with no force and little breaking, and moments with larger forces and more breaking. The flow due to peristaltic motions changes on time scales short compared to the typical bacterial division time, thus we will assume that periods of low breaking and high breaking rates will be equivalent to an average effective breaking rate. Then let us consider the case of elongation only, as portrayed here.

As we assume here that the velocity gradient is constant, the relative fluid velocity grows linearly with the distance from the center of mass of the chain. Then the force on each bead is equal to F_0 multiplied by the distance to the center divided by B. We assume, following [7][8], that the breaking rate is dependent on the force. Thus, we define α and β such that the breaking rate of a link is $\alpha \exp(\beta F/F_0)$ if a force F is applied to the link. In the limit of small force, the breaking rate will be α, the same for all links, as in the base model. β is some constant caracterizing how much the stability of the link is force-dependent.

We can write the force on each bead (pannel C of figure F). Then, here, because the chain is rigid and straight, the sum of the forces on each bead has to be zero. The tension on the outermost link will simply be equal to the flow force on the outermost bead, i.e. F_0 multiplied by its distance to the center divided by B, i.e. $(N-1)/2$ (both for chains of odd and even number of beads). On the next link, the tension has to compensate for the flow force on the second bead, plus the tension applied by the outermost link. Thus the tension on this link is $F_0((N-1)/2+(N-1)/2-1)$, and so forth (this is analogous to modelling of breaking of polymer chains in elongational flows, as in[11]).

For N even, the force on the jth link starting from the outermost link will

be:

$$F_{jth\ link,N\ even} = F_0 \sum_{k=N/2-j+1}^{N/2} (k-1/2)$$

Using $\sum_{i=1}^{n} i = n(n+1)/2$, it can be rewritten as:

$$F_{jth\ link,N\ even} = F_0 \left(\frac{N^2}{8} - \frac{(N-2j)^2}{8} \right).$$

There are two links j^{th} away from the extremities, for j from 1 to $N/2-1$, and one central link, for which $j = N/2$. The breaking rate of a given link is $\alpha \exp(\beta F/F_0)$ with F the total force applied to the link. Then the total breaking rate of one chain of length N even is:

$$\alpha \exp\left(\frac{\beta N^2}{8}\right) \left(1 + 2\sum_{k=2}^{N/2} \exp\left(-\frac{\beta}{2}(k-1)^2\right)\right). \tag{S1}$$

An outermost link of a chain of length $N+1$ (with N even, $N+1$ is odd) breaks at rate $\alpha \exp(\beta N/2)$. There are two such links for each chain. This and equation (S1) lead to equation (30) of the main text:

$$\frac{dn_i}{dt} = -rin_i - \alpha n_i \exp\left(\frac{\beta i^2}{8}\right) \left(1 + 2\sum_{k=2}^{i/2} \exp\left(-\frac{\beta}{2}(k-1)^2\right)\right) + r(i-1)n_{i-1} + 2\alpha n_{i+1} \exp\left(\frac{\beta i}{2}\right)$$

For N odd, the force on the jth link starting from the outermost link will be:

$$F_{jth\ link,N\ odd} = F_0 \sum_{k=(N-1)/2-j+1}^{(N-1)/2} k.$$

Simiarly to the N even case, we can rewrite:

$$F_{jth\ link,N\ odd} = F_0 \left(\frac{N^2}{8} - \frac{(N-2j)^2}{8} \right)$$

Because of the two sides, there are two links j for each chain, for j from 1 to $(N-1)/2$. The breaking rate of a given link is $\alpha \exp(\beta F/F_0)$ with F the total force applied to the link. Then the total breaking rate of one chain of length N odd is:

$$2\alpha \exp\left(\frac{\beta N^2}{8}\right) \sum_{k=1}^{(N-1)/2} \exp\left(-\frac{\beta}{2}\left(k - \frac{1}{2}\right)^2\right) \tag{S2}$$

An outermost link of a chain of length $N+1$ (with N odd, $N+1$ is even) breaks at rate $\alpha \exp(\beta N/2)$. There are two such links for each chain. Then, this and equation (S2) lead to equation (31) of the main text for the evolution in time of the mean number of chains of odd length i:

$$\frac{dn_i}{dt} = -rin_i - 2\alpha n_i \exp\left(\frac{\beta i^2}{8}\right) \sum_{k=1}^{(i-1)/2} \exp\left(-\frac{\beta}{2}\left(k - \frac{1}{2}\right)^2\right) + r(i-1)n_{i-1} + 2\alpha n_{i+1} \exp\left(\frac{\beta i}{2}\right).$$

E.2 Additional figure for the force-dependent model: replication rate maximizing the growth rate as a function of β

Figure G shows that the rate of replication maximizing the growth rate of free bacteria increases exponentially with β, which represent the strength of the dependence of the breaking rate on the force applied to the link.

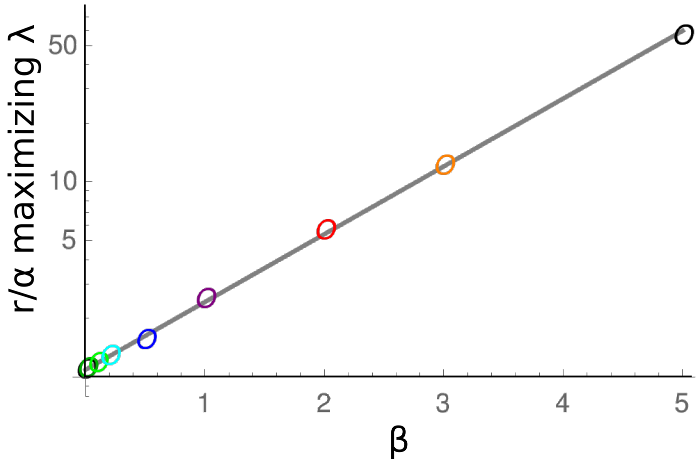

Figure G: Log of the value of r/α maximizing the growth rate in the force-dependent breaking rate model as a function of β. The points are numerical maximums, the line is $1.09 \times \exp(0.8\beta)$. 1.09 is the value of (r/α) maximizing the growth rate for the base model (i.e. for $\beta \to 0$).

E.3 Force-dependent model: approximation for the chain length distribution.

We start from equations (30) and (31), and assume that for t long enough, $n_i \simeq C p_i \exp(\lambda t)$ (with λ the largest eigenvalue). Then,

$$\lambda p_i = -r i p_i - \alpha p_i \exp(\beta i^2/8) X + r(i-1)p_{i-1} + 2\alpha p_{i+1}\exp(\beta i/2) \tag{S3}$$

with $X = 1 + 2\sum_{j=1}^{i/2-1}\exp(-\beta j^2/2)$ (i even) or $X = 2\sum_{j=1}^{(i-1)/2}\exp(-\beta(j-1/2)^2/2)$ (i odd). Let us now determine which terms dominate in this expression.

For i large enough, $\lambda \ll ri$. Thus λp_i is negligible relative to $r i p_i$.

For both i even and odd, X is a converging sum which tends to a finite number when i increases. Let us denote its limit $Y = 1+2\sum_{j=1}^{\infty}\exp(-\beta j^2/2) = \theta_3(0, \exp(-\beta/2)))$ in the even case, and $Z = 2\sum_{j=1}^{\infty}\exp(-\beta(j-1/2)^2/2) = \theta_2(0, \exp(-\beta/2)))$ in the odd case, with θ_i the Jacobi Theta functions. Thus, because β is positive, for i large enough, $ri \ll \alpha \exp(\beta i^2/8)X$.

The remaining main terms in equation (S3) are:

$$-\alpha p_i \exp(\beta i^2/8)X + r(i-1)p_{i-1} + 2\alpha p_{i+1}\exp(\beta i/2) \simeq 0.$$

The first term is negative, the two others are positive. Then we have to determine which of $r(i-1)p_{i-1}$ and $2\alpha p_{i+1}\exp(\beta i/2)$ dominates. If $2\alpha p_{i+1}\exp(\beta i/2)$ dominates, $\alpha p_i \exp(\beta i^2/8)X \simeq 2\alpha p_{i+1}\exp(\beta i/2)$, thus $p_{i+1}/p_i \simeq \exp(\beta i(i/8 - 1/2))X$, which for i large enough means that the long the chain, the more of it, which would diverge and does not make sense in this system. Thus $\alpha p_i \exp(\beta i^2/8)X \simeq r(i-1)p_{i-1}$,

$$\frac{n_i}{n_{i-1}} \to \frac{p_i}{p_{i-1}} \simeq \frac{r}{\alpha}\frac{i-1}{X}\exp\left(-\beta\frac{i^2}{8}\right). \tag{S4}$$

This approximation is valid for large chain sizes. We assume that it is valid for any chain length. As this expression is small and decreasing quickly with increasing i, p_1 will be close to 1. Then, as:

$$\frac{p_i}{p_1} = \Pi_{j=2}^{i}\frac{p_j}{p_{j-1}},$$

and using the known expression for the sum of the squares $\sum_{j=1}^{i} j^2 = (n + 3n^2 + 2n^3)/6$ and expression (S4):

$$p_{i,even} \simeq \left(\frac{r}{\alpha}\right)^{i-1}\frac{(i-1)!}{Y^{i/2}Z^{i/2-1}}\exp\left(-\frac{\beta}{8}\left(-1+\frac{i+3i^2+2i^3}{6}\right)\right)$$

$$p_{i,odd} \simeq \left(\frac{r}{\alpha}\right)^{i-1}\frac{(i-1)!}{Y^{(i-1)/2}Z^{(i-1)/2}}\exp\left(-\frac{\beta}{8}\left(-1+\frac{i+3i^2+2i^3}{6}\right)\right)$$

These two equations can be combined, and ultimately lead to:

$$p_i \simeq \left(\frac{r}{\alpha}\right)^{i-1}\frac{(i-1)!}{Y^{floor(i/2)}Z^{floor((i-1)/2)}}\exp\left(-\frac{\beta}{8}\left(-1+\frac{i+3i^2+2i^3}{6}\right)\right)$$

E.4 Additional figure for the force-dependent model: chain length distribution for other values of r/α

In panel 2J of the main text, we represented the distribution of chain lengths in the model with force-dependent link breaking rate for $r/\alpha = 1$. In figure H we represent the distributions for different values of r/α. Overall, the shapes are similar, and the smaller r/α is (as well as the larger β is), the better the analytical approximation works.

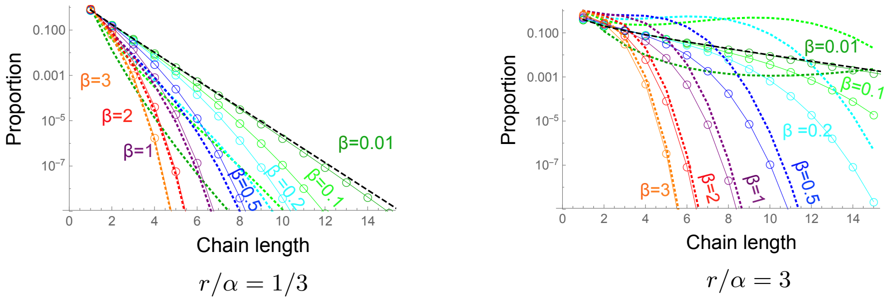

Figure H: Chain length distribution, as in figure 2J, except for the value of r/α: model with force-dependent breaking rates. Each color represents a different β: $\beta = 0.01$ ($n_{max} = 20$), $\beta = 0.1$ ($n_{max} = 15$), $\beta = 0.2$ ($n_{max} = 15$), $\beta = 0.5$ ($n_{max} = 15$), $\beta = 1$ ($n_{max} = 15$), $\beta = 2$ ($n_{max} = 10$), $\beta = 3$ ($n_{max} = 10$). The black dashed lines are the numerical results for the base model, equivalent to $\beta = 0$. The curves for $\beta = 0.01$ (dark green) are almost overlaid with the curves for $\beta = 0$. The colored dotted lines the analytical approximation (equation (18) of the main text), and the black dotted line the approximation for the base model (equation (5) of the main text).

F Zoomed-in distribution for all models

Figure I represents the zoomed-in chain length distributions of the right panels of figure 2 of the main text.

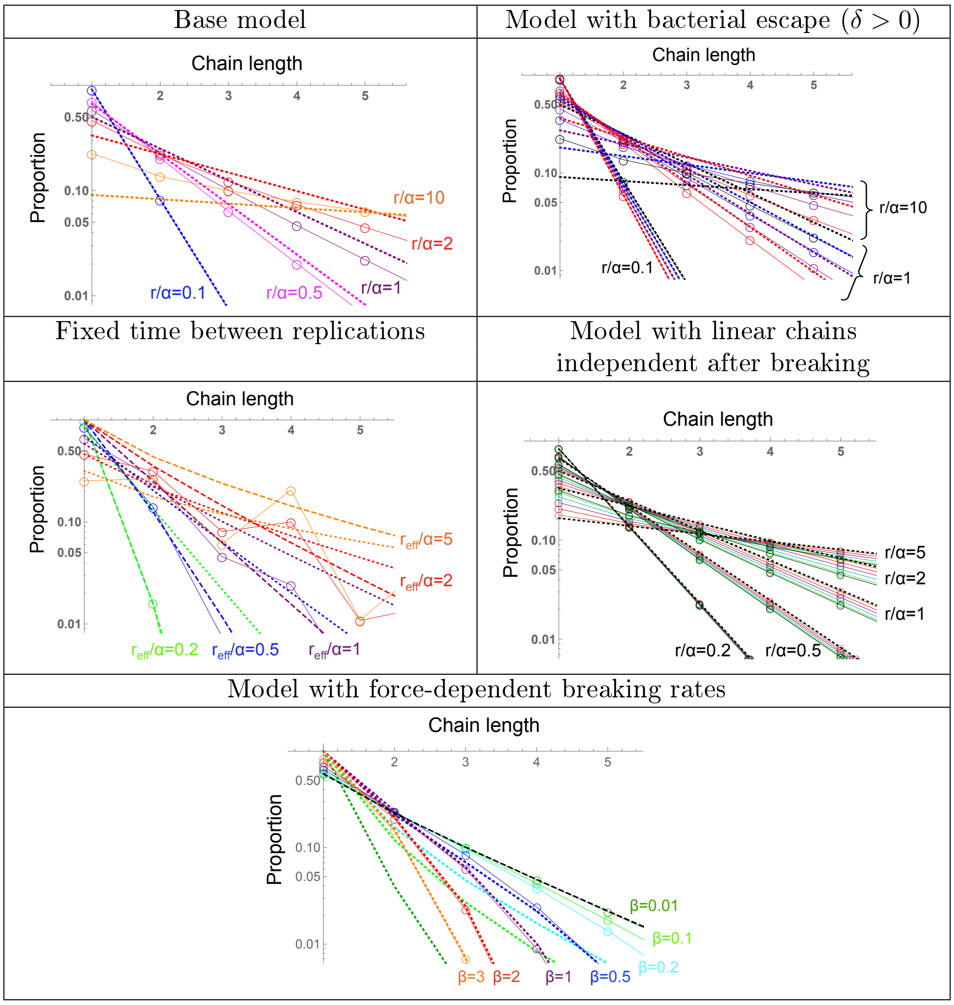

Figure I: Zoomed-in chain length distributions: everything as in the right panels of figure 2 of the main text, but centered on the head of the distributions. Solid lines and open circles: numerical results. **Base model**: $n_{max} = 40$, dotted lines: approximation (5) of the main text (almost overlaid with the numerical results for $r/\alpha = 0.1$). **Model with bacterial escape**: $\delta = \delta' = \delta'' = \mathbf{0}$, 0.1, 0.2, 0.3, $c = c' = 0$, $n_{max} = 40$. Dotted lines: approximation (9) of the main text. **Fixed time between replications**: $r_{eff} = \log(2)/\tau$, $n_{max} = 32$. Approximation (18) (dashed lines), numerical result in the base model (dotted lines). $r/\alpha = 0.2$, 0.5, 1, 2, 5. **Model with linear chains independent after breaking**: $n_{max} = 100$. The dotted black lines are the approximate distribution (27) of the main text for each r/α, which is the exact distribution for $q = 1$. The colors represent $q = \beta = 0$, 0.1, 0.2, 0.3, 0.4, 0.5, 0.6, 0.7, 0.8, 0.9, **1**. All curves are almost overlaid for small r. **Model with force-dependent breaking rates**: chain length distribution for $r/\alpha = 1$. Each color represents a different β: $\beta = 0.01$ ($n_{max} = 20$), $\beta = 0.1$ ($n_{max} = 15$), $\beta = 0.2$ ($n_{max} = 15$), $\beta = 0.5$ ($n_{max} = 15$), $\beta = 1$ ($n_{max} = 15$), $\beta = 2$ ($n_{max} = 10$), $\beta = 3$ ($n_{max} = 10$). The colored dotted lines the analytical approximation (18) of the main text, and the black dotted line the approximation (5) of the main text for the base model.

G Experimental data

G.1 Methods

We perform a new analysis on images that were produced for [4]. We briefly describe below the experiments from which the images were produced, and describe our analysis.

Mice, which were previously vaccinated with a peracetic-acid inactivated S.Typhimurium strain (PA-S.Tm), were pretreated with 0.8g/kg ampicillin sodium salt in sterile PBS. 24h later, mice received 10^5 CFU of a 1:1 mix of mCherry-(pFPV25.1) and GFP-(pM965) expressing attenuated S. Tm M2702. For imaging, cecum content was diluted gently 1:10 w/v in sterile PBS containing 6μg/ml chloramphenicol to prevent growth during imaging. 200μl of the suspension were transferred to an 8-well Nunc Lab-Tek Chambered Coverglass (Thermo Scientific) and imaged at 100x using the Zeiss Axiovert 200m microscope. To determine the distribution of bacteria in aggregates, n=25 high power fields per mouse were randomly selected and imaged for mCherry and GFP fluorescence. For some mice, sequential sampling was done, these mice were terminally anaesthetised and artificially respirated cecum content was sampled by tying off part of the cecum each hour for 3h. More details about the experimental procedures can be found in [4].

We analyzed all the images for the early data points (4 and 5 hours) of experiments starting from a low inoculum (10^5), to minimize the clustering from random encounters. Only the linear chains were counted. Images are for the red and green fluorescence, so complex clusters with two colors were not counted. The data were analyzed manually. The images are available as supplementary materials:

- File S1 images4h.zip contains the images for 3 of the mice only sampled at 4h.
- File S2 images4h_others.zip contains the images for the other 4 mice only sampled at 4h.
- File S3 images5h.zip contains the images of the mice only sampled at 5h
- File S4 imagesseq4h.zip contains the images at 4h of mice sampled sequentially
- File S5 imagesseq5h.zip contains the images at 5h of mice sampled sequentially

.

G.2 Results

For linear chains, we obtained the length distribution detailed in table 1 and shown on figure 4 of the main text.

Given the bumpy shape of the experimental distribution, we chose to fit the data with the fixed replication time model for the figure 4 of the main text. In this model, the only adjustable parameter is r_{eff}/α. For a given r_{eff}/α, we obtain the theoretical chain length distribution p_i by numerical resolution

chain length	4h PI o (7 mice)	4h PI s (2 mice)	5h PI o (4 mice)	5h PI s (2 mice)	total
2	21	30	17	38	106
3	22	4	9	5	40
4	51	9	25	9	94
5	7	0	1	3	11
6	5	3	3	4	15
7	10	1	5	3	19
8	12	0	4	3	19
9	1	0	0	0	1
10	1	0	0	1	2
11	1	0	0	0	1
12	0	0	1	1	2
13	0	0	1	1	2
14	0	0	1	0	1

Table 1: Table of the linear chains counted on the images from several experiments, either with mice sampled once (o), or with mice sampled sequentially (s).

of the equations. As the data concerns only chains of length 2 and longer, we renormalize this distribution as $p_i/(1-p_1)$. Then, the likelihood observing k_2 chains of length 2, k_3 chains of length 3, ... k_i chains of length i is $\Pi_{i=2}^{\infty}\left(\frac{p_i}{1-p_1}\right)^{k_i}$ multiplied by a combinatorial factor dependent only on the k_i, to express the number of possible ways to choose k_2, k_3, ... k_i, ... among the total number of clusters observed. Thus the log-likelihood is equal to:

$$\log(likelihood) = \sum_{i=2}^{\infty} k_i \log\left(\frac{p_i}{1-p_1}\right) + constant(independent\ of\ r_{eff})$$

In practice, here, there are no clusters observed longer than $i_{max} = 14$. We compute numerically this log-likelihood as a function of r_{eff} (p_i depends on r_{eff}). The value of r_{eff} maximizing the log likelihood is 4.1.

A confidence interval of 95% can be approximated by the interval of r_{eff} for which the difference between the log likelihood and its maximum is less than 1.92 [12]. This results in a confidence interval of $3.7 \leq r_{eff} \leq 4.6$.

To quantify our impression that there are fewer long chains observed than expected, we performed the following calculations. Taking $r_{eff}/\alpha = 4.1$ and $N_{exp} = 313$, the expected number of chains of length 15 or longer is 3.7, whereas none is observed, which for a multinomial distribution has a probability $\simeq 0.024$ to occur.This probability seems low. Either this is a low probability but still happened (and if we look at a bit shorter chains, the expected average number of chains of length 9 and longer is 11.7, and 9 of them are actually observed, which is relatively close); or there is some process limiting the number of long chains. There are two main possibilities for the number of long chains to be limited: there could be an experimental bias limiting the observation of long chains (see discussion below); or there could be some force-dependence of the

breaking rates, which would effectively act as a cut-off for the chain length (see figure 3 of main text), as in this case, breaking rates increase considerably with chain length.

G.3 Discussion

The data may be biased. The mass of one bacterium is about one pg, and its density is about 10% more than the water density[13, 14], the thermal energy at ambient temperature is of the order of $4.10^{-21}J$, and gravity g is of the order of $10m/s^2$, thus thermal fluctuations will lift an individual bacterium by typically 4 μm higher than the bottom. Thermal fluctuations will have two effects:

- The average height of the center of gravity of chains will decrease with their length. This is confocal microscopy, which typical optical section is less than 1μm, focused close to the cover slip. This may bias the distribution by missing smaller chains.
- Longer chains are not rod-like, their shape fluctuate. It is apparent on the microscopy images that parts of long chains may get out of focus. The longer the chain, the less likely that it is entirely in the focus, and thus chains will look smaller than they are.

We focus on the chain length distribution because this quantity is more easily accessible by experimental measurements, at the end of an experiment. Comparing models and experiments enables to check whether the data is compatible with a process of growing and breaking of clusters; and determine which specific model is closest to the data. However, some models cannot be distinguished, no matter how much data is available for the chain length distribution. For example in the model with bacterial escape and the model where chains can remain independent after breaking, there are two parameters to fit (r/α and δ, or r/α and q). It is likely that fitting would mainly select a value for r/α, since the distribution does not depend much on the second parameter in both cases. These models could not be distinguished from the base model. On the other hand, models with different distribution shapes – either in the force-dependent model or in the fixed division time one– could be distinguished, provided that the bias can be overcome, and that more data can be collected.
We could fit the fixed replication time model to the data, and this strengthened our hypothesis that the chains are generated by a process of enchained growth and link breaking. However, there is somewhat less long chains observed than expected (especially in the range of lengths 14 to 16). One possibility could be that the breaking rate is force dependent. If we had 10 times more of unbiased data, we could answer whether there really is a deficit of longer chains. If there is indeed a deficit of longer chains, then we should combine the model of force-dependent breaking rates with the model with fixed replication time, to be able to make quantitative comparisons. To be more effective, comparison would likely require more data, as there would be two free parameters, r/α and β. If there is no deficit of longer chains in the range up to 16, then the simple model with fixed replication time predicts that we would get access to the distribution up to length 24 (and be at the limit for lengths 28 and 32) with 100 times more data than in current experiments (see figure J). Thus overall we would

need at least 10 times and likely 100 times more data for a more quantitative assessment.

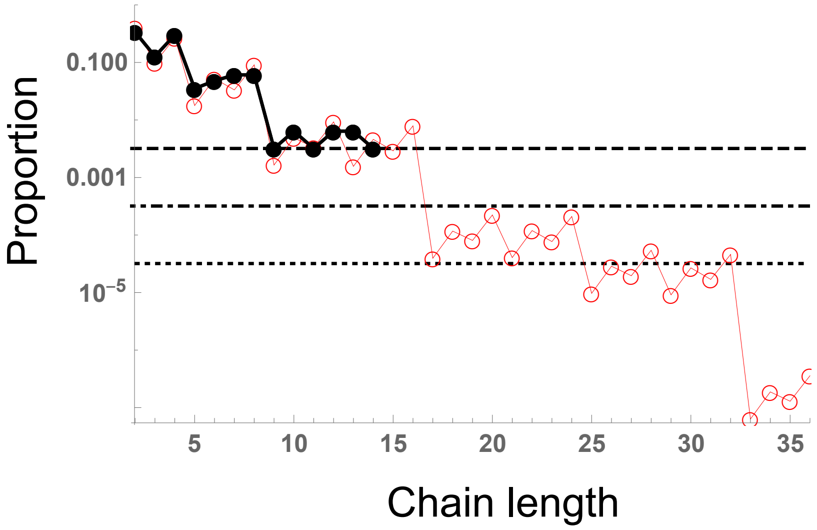

Figure J: Comparison of chain length distribution for the experimental data (black) and the model with fixed replication time (red). This figure is as figure 4 of main text, but extended up to length 36. The black dashed line represents limit of one chain in the data. The black dot-dashed line represents what this limit would be if there was 10 times more data, and the black dotted line represents what this limit would be if there was 100 times more data. $r_{eff}/\alpha = 4.1$ (with $r_{eff}/\alpha = \log(2)/(\alpha\tau)$).

Increasing the amount of data would not necessarily require to sacrifice more mice, but merely to take more images for each cecum content. The challenge would be to do so with no bias, and with very standardized conditions so that the images are taken in conditions close enough so as to automate the chain detection and length count.

It would be also very useful if there would be ways to estimate the breaking rate in independent experiments, for instance injecting (without breaking them nor perturbing the system) chains of non-replicating bacteria of controlled length, and measuring how the length distribution changes over time. Then, as the replication rate can be estimated by other measures (dilution of non-replicating plasmids), we could get an estimate of the replication rate over the breaking rate, which would considerably constrain the fitting of the chain length distribution, and thus give more strength to the conclusions achieved.

References

[1] Berg HC. Random walks in biology. Princeton University Press; 1993. 1

[2] Berg HC. E. coli in Motion. Springer Science & Business Media; 2008. 1

[3] Elgeti J, Winkler RG, Gompper G. Physics of microswimmers—single particle motion and collective behavior: a review. Reports on progress in physics. 2015;78(5):056601. 1

[4] Moor K, Diard M, Sellin ME, Felmy B, Wotzka SY, Toska A, et al. High-avidity IgA protects the intestine by enchaining growing bacteria. Nature. 2017;544(7651):498–502. doi:10.1038/nature22058. 1, 16

[5] World Health Organization & Food and Agriculture Organization. Risk assessments of Salmonella in eggs and broiler chickens. vol. http://www.who.int/foodsafety/publications/micro/salmonella/en/; 2002. 1

[6] Brandtzaeg P. Role of secretory antibodies in the defence against infections. International Journal of Medical Microbiology. 2003;293(1):3–15. doi:10.1078/1438-4221-00241. 7

[7] Evans E, Berk D, Leung A. Detachment of agglutinin-bonded red blood cells. I. Forces to rupture molecular-point attachments. Biophysical Journal. 1991;59(4):838–848. doi:10.1016/S0006-3495(91)82296-2. 7, 9

[8] Evans EA, Calderwood DA. Forces and bond dynamics in cell adhesion. Science. 2007;316(5828):1148–1153. 7, 9

[9] Lentle R, Janssen P. Physical characteristics of digesta and their influence on flow and mixing in the mammalian intestine: a review. Journal of Comparative Physiology B. 2008;178(6):673–690. 9

[10] Hulls C, Lentle RG, de Loubens C, Janssen PW, Chambers P, Stafford KJ. Spatiotemporal mapping of ex vivo motility in the caecum of the rabbit. Journal of Comparative Physiology B. 2012;182(2):287–297. 9

[11] Odell J, Keller A. Flow-induced chain fracture of isolated linear macromolecules in solution. Journal of Polymer Science Part B: Polymer Physics. 1986;24(9):1889–1916. 9

[12] Owen A, et al. Empirical likelihood ratio confidence regions. The Annals of Statistics. 1990;18(1):90–120. 17

[13] Bratbak G, Dundas I. Bacterial dry matter content and biomass estimations. Applied and environmental microbiology. 1984;48(4):755–757. 18

[14] Baldwin WW, Myer R, Powell N, Anderson E, Koch AL. Buoyant density of Escherichia coli is determined solely by the osmolarity of the culture medium. Archives of microbiology. 1995;164(2):155–157. 18
