# Supplementary figures and images for: Enchained growth and cluster dislocation: A possible mechanism for microbiota homeostasis (part 2 of 10)
Source: PLoS Comput Biol. 2019 May 3;15(5):e1006986. doi: 10.1371/journal.pcbi.1006986 (PMC6519844; doi:10.1371/journal.pcbi.1006986)

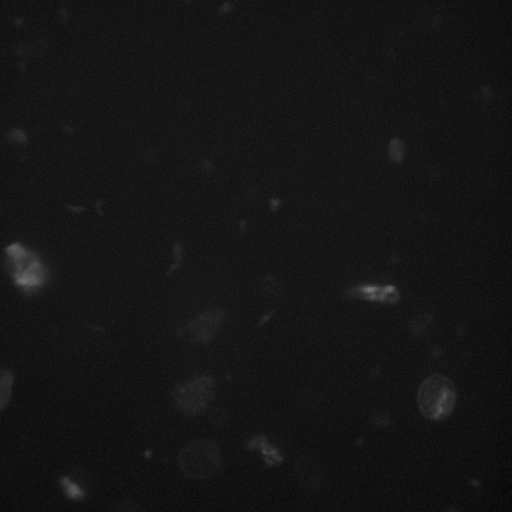

Supplement: S1 File — (ZIP) [file pcbi.1006986.s002.zip › extrait4h/4h_Z125_1_w1sdcRFP.tif]

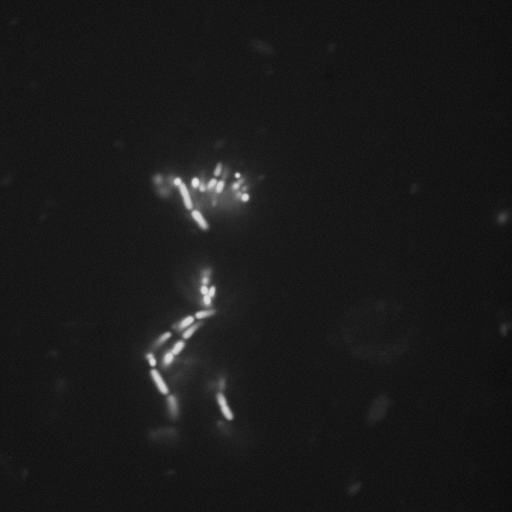

Supplement: S1 File — (ZIP) [file pcbi.1006986.s002.zip › extrait4h/4h_Z129_30_w2sdcGFP.tif]

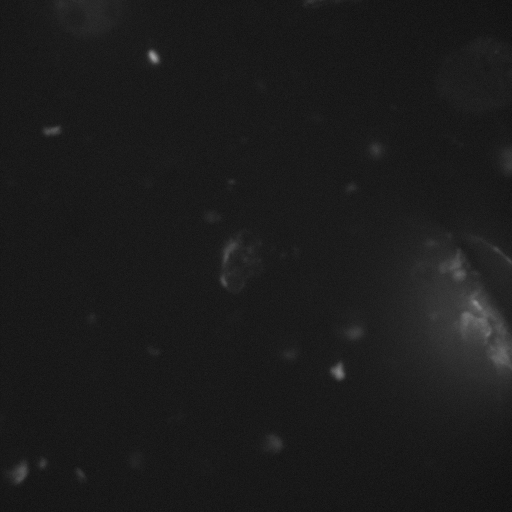

Supplement: S1 File — (ZIP) [file pcbi.1006986.s002.zip › extrait4h/4h_Z125_4_w2sdcGFP.tif]

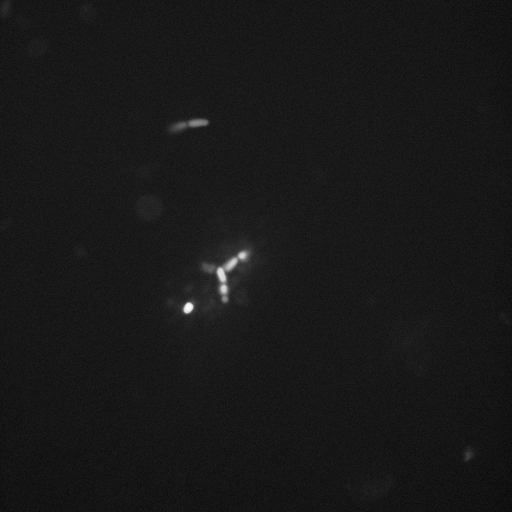

Supplement: S1 File — (ZIP) [file pcbi.1006986.s002.zip › extrait4h/4h_Z129_3_w1sdcRFP.tif]

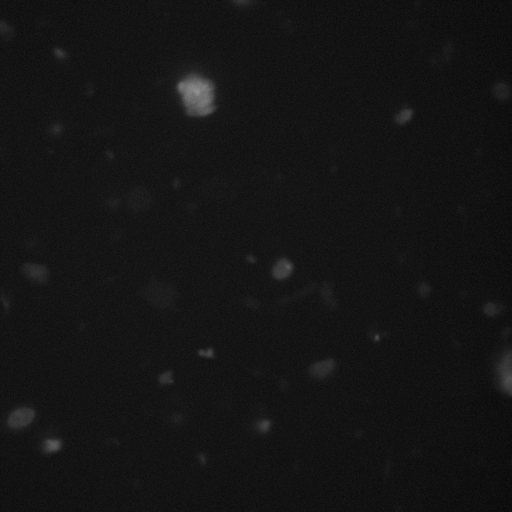

Supplement: S1 File — (ZIP) [file pcbi.1006986.s002.zip › extrait4h/4h_Z128_29_w2sdcGFP.tif]

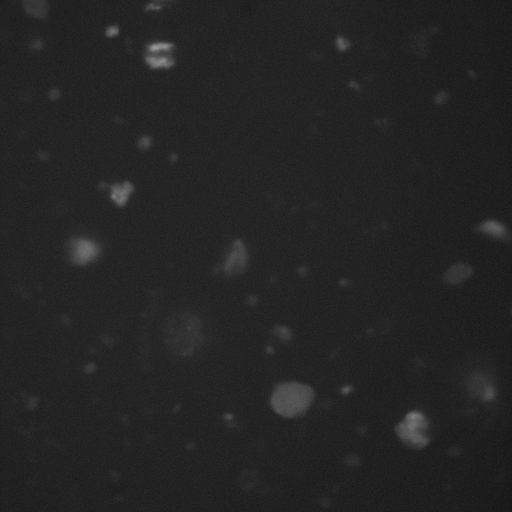

Supplement: S1 File — (ZIP) [file pcbi.1006986.s002.zip › extrait4h/4h_Z129_25_w2sdcGFP.tif]

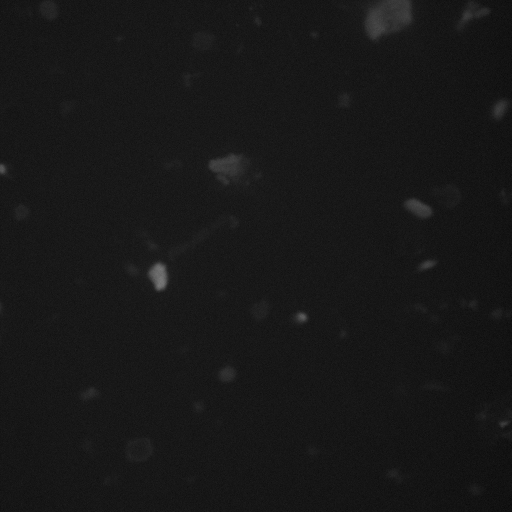

Supplement: S1 File — (ZIP) [file pcbi.1006986.s002.zip › extrait4h/4h_Z128_7_w2sdcGFP.tif]

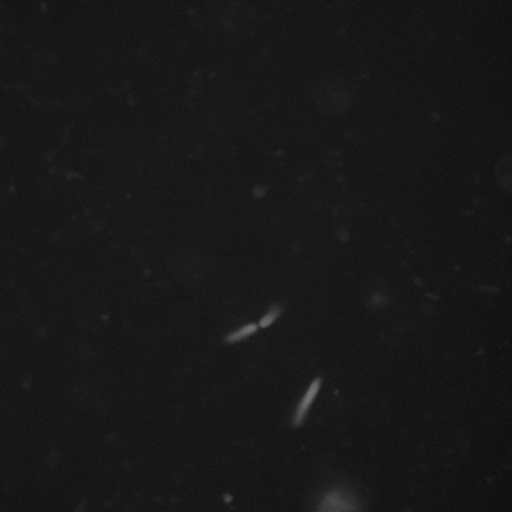

Supplement: S1 File — (ZIP) [file pcbi.1006986.s002.zip › extrait4h/4h_Z125_25_w2sdcGFP.tif]

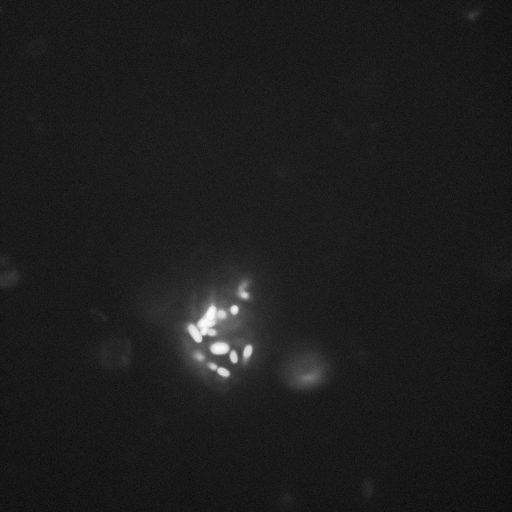

Supplement: S1 File — (ZIP) [file pcbi.1006986.s002.zip › extrait4h/4h_Z129_4_w1sdcRFP.tif]

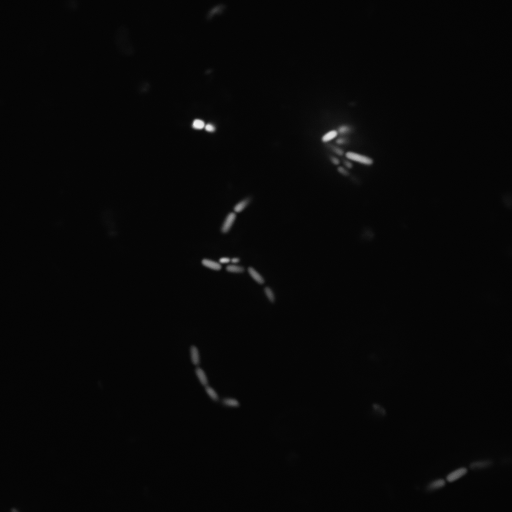

Supplement: S1 File — (ZIP) [file pcbi.1006986.s002.zip › extrait4h/4h_Z129_42_w1sdcRFP.tif]

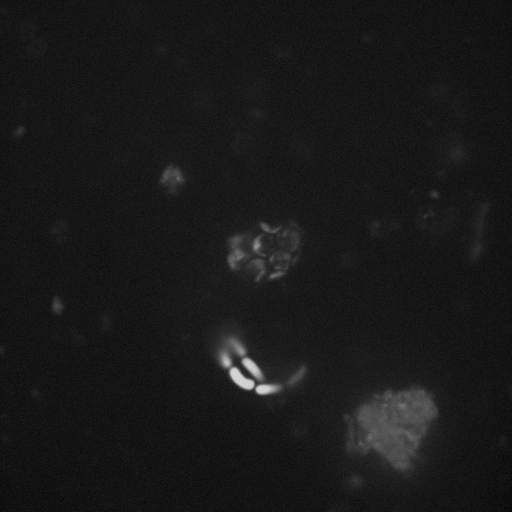

Supplement: S1 File — (ZIP) [file pcbi.1006986.s002.zip › extrait4h/4h_Z125_15_w1sdcRFP.tif]

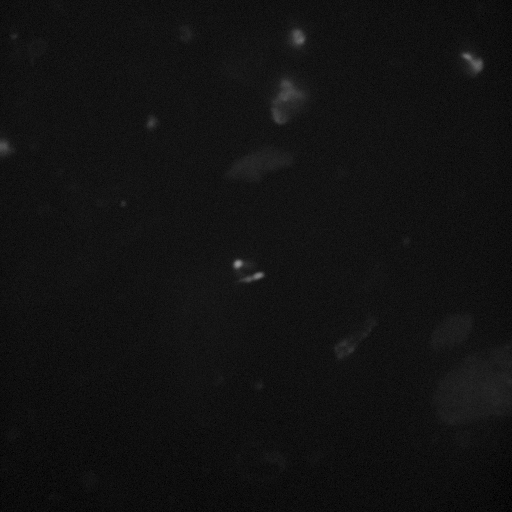

Supplement: S1 File — (ZIP) [file pcbi.1006986.s002.zip › extrait4h/4h_Z128_19_w1sdcRFP.tif]

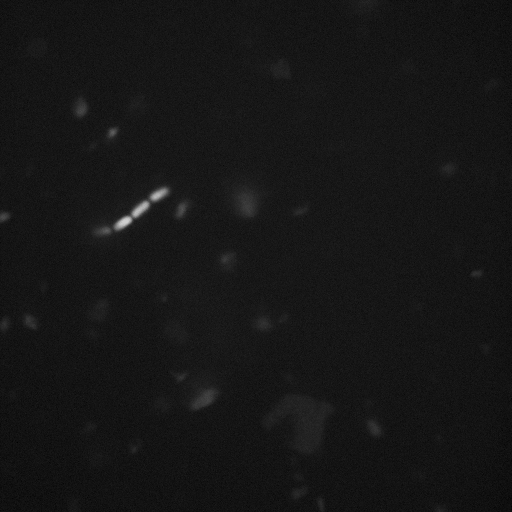

Supplement: S1 File — (ZIP) [file pcbi.1006986.s002.zip › extrait4h/4h_Z125_22_w1sdcRFP.tif]

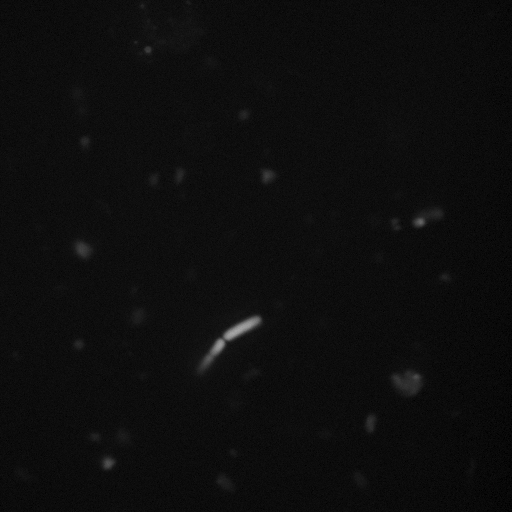

Supplement: S1 File — (ZIP) [file pcbi.1006986.s002.zip › extrait4h/4h_Z128_17_w2sdcGFP.tif]

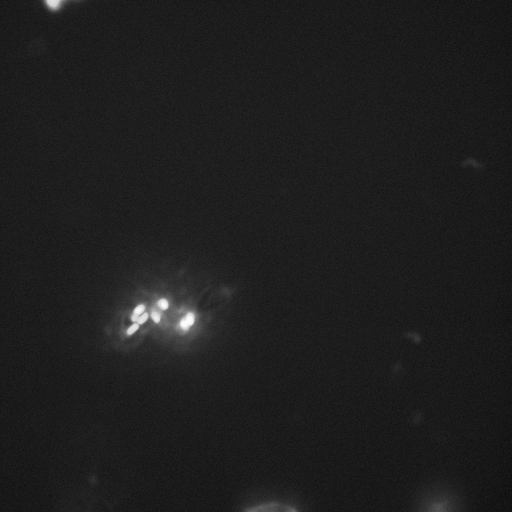

Supplement: S1 File — (ZIP) [file pcbi.1006986.s002.zip › extrait4h/4h_Z129_2_w1sdcRFP.tif]

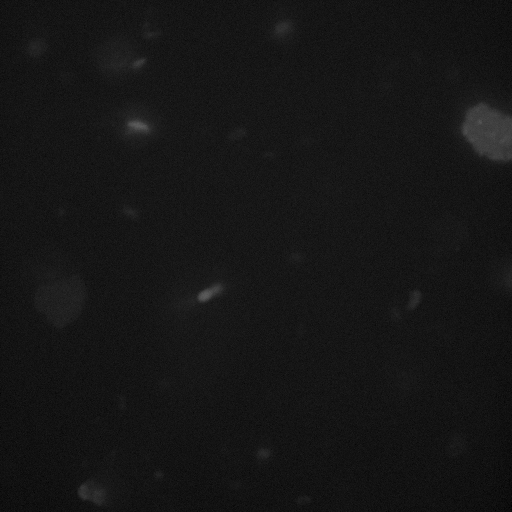

Supplement: S1 File — (ZIP) [file pcbi.1006986.s002.zip › extrait4h/4h_Z125_17_w1sdcRFP.tif]

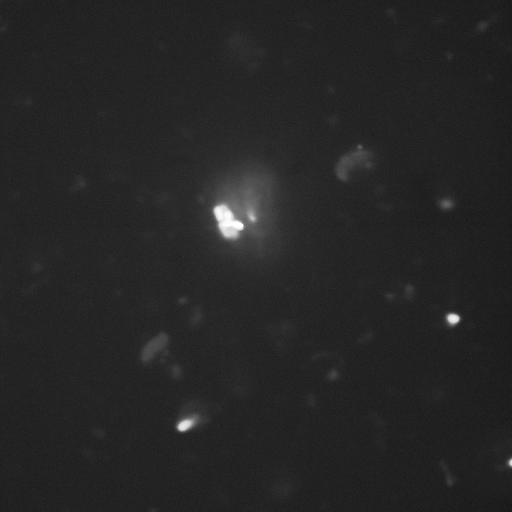

Supplement: S1 File — (ZIP) [file pcbi.1006986.s002.zip › extrait4h/4h_Z129_22_w2sdcGFP.tif]

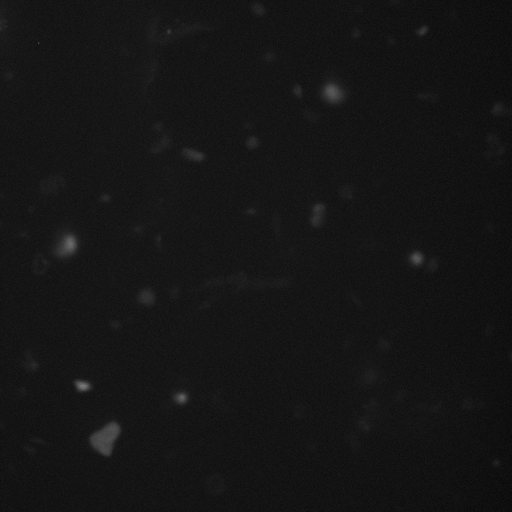

Supplement: S1 File — (ZIP) [file pcbi.1006986.s002.zip › extrait4h/4h_Z128_41_w2sdcGFP.tif]

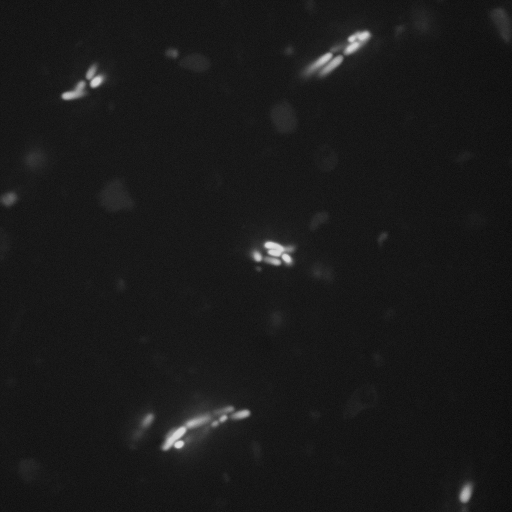

Supplement: S1 File — (ZIP) [file pcbi.1006986.s002.zip › extrait4h/4h_Z129_38_w2sdcGFP.tif]

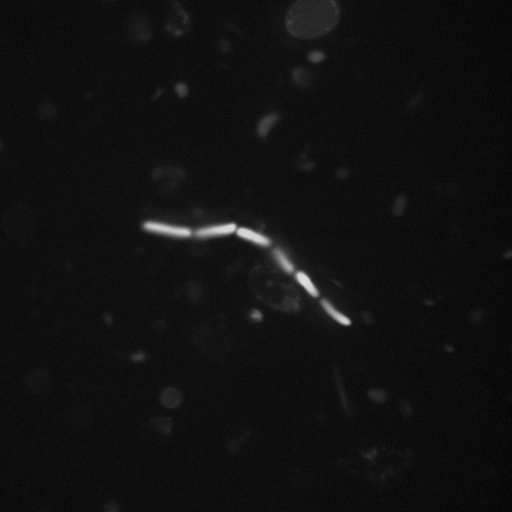

Supplement: S1 File — (ZIP) [file pcbi.1006986.s002.zip › extrait4h/4h_Z128_44_w2sdcGFP.tif]

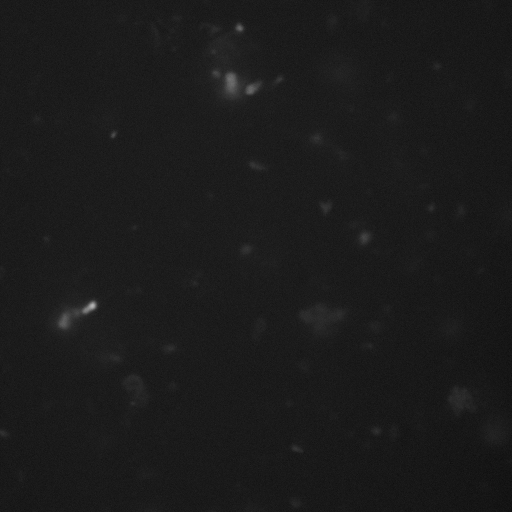

Supplement: S1 File — (ZIP) [file pcbi.1006986.s002.zip › extrait4h/4h_Z125_9_w2sdcGFP.tif]

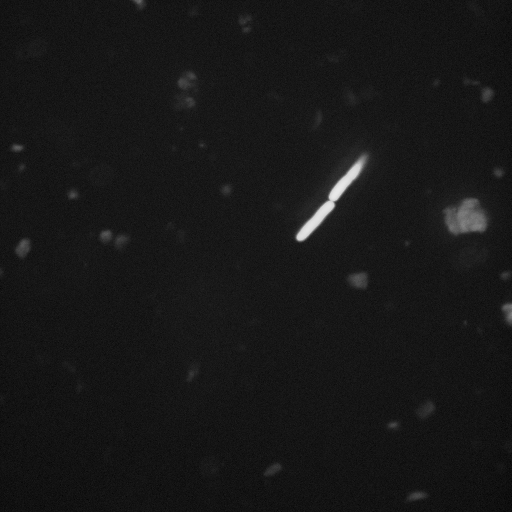

Supplement: S1 File — (ZIP) [file pcbi.1006986.s002.zip › extrait4h/4h_Z125_30_w1sdcRFP.tif]

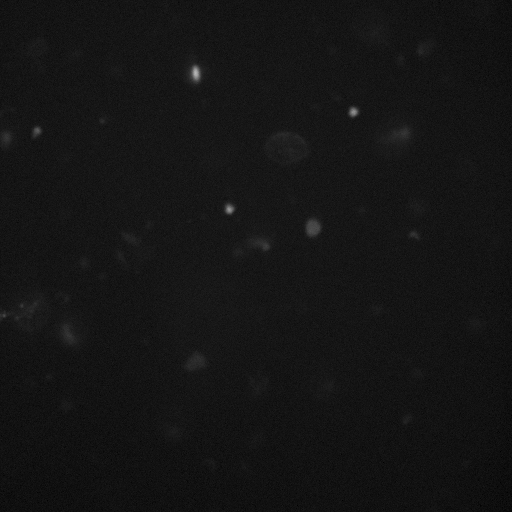

Supplement: S1 File — (ZIP) [file pcbi.1006986.s002.zip › extrait4h/4h_Z128_34_w1sdcRFP.tif]

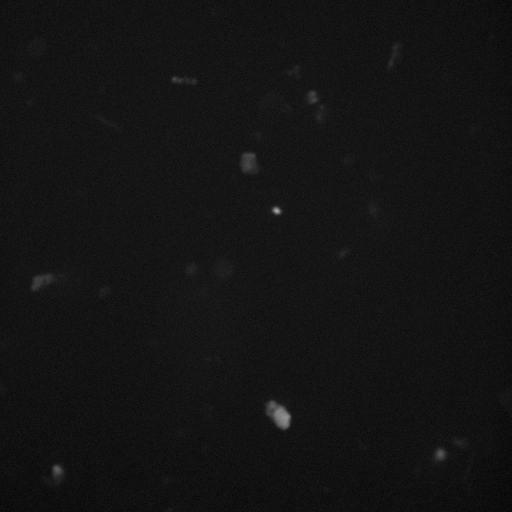

Supplement: S1 File — (ZIP) [file pcbi.1006986.s002.zip › extrait4h/4h_Z128_25_w1sdcRFP.tif]

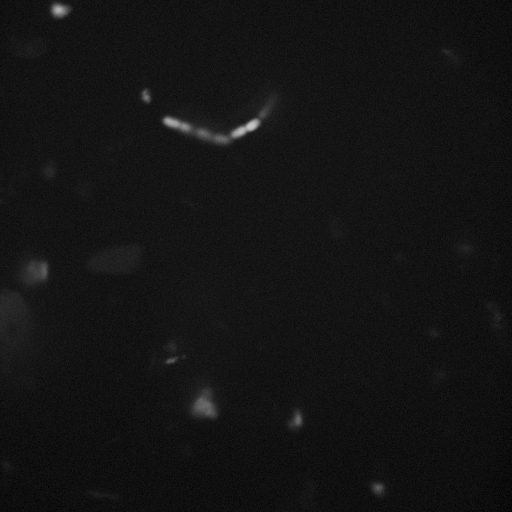

Supplement: S1 File — (ZIP) [file pcbi.1006986.s002.zip › extrait4h/4h_Z128_33_w1sdcRFP.tif]

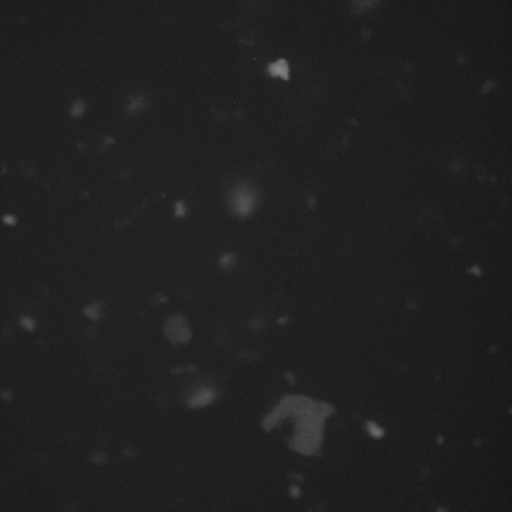

Supplement: S1 File — (ZIP) [file pcbi.1006986.s002.zip › extrait4h/4h_Z125_22_w2sdcGFP.tif]

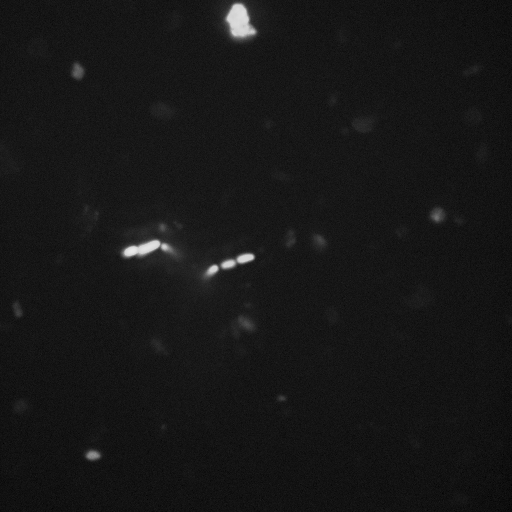

Supplement: S1 File — (ZIP) [file pcbi.1006986.s002.zip › extrait4h/4h_Z129_33_w1sdcRFP.tif]

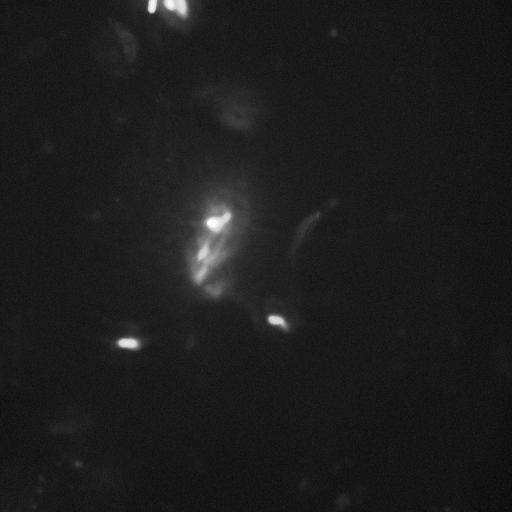

Supplement: S1 File — (ZIP) [file pcbi.1006986.s002.zip › extrait4h/4h_Z129_34_w1sdcRFP.tif]

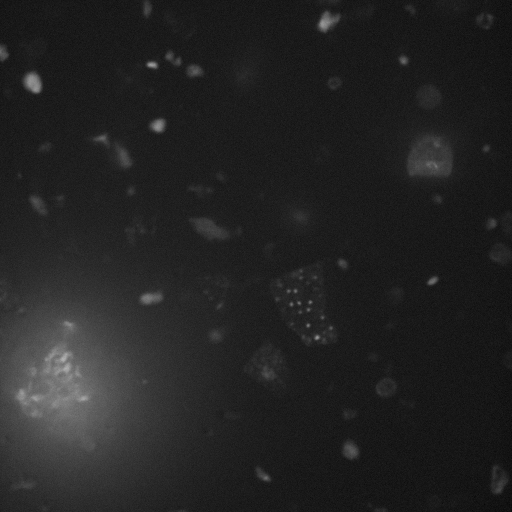

Supplement: S1 File — (ZIP) [file pcbi.1006986.s002.zip › extrait4h/4h_Z125_20_w1sdcRFP.tif]

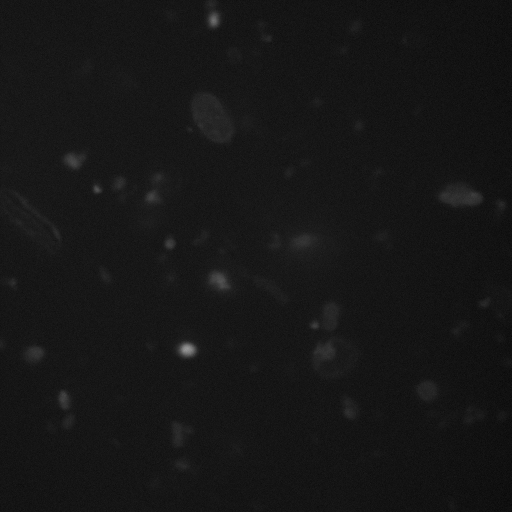

Supplement: S1 File — (ZIP) [file pcbi.1006986.s002.zip › extrait4h/4h_Z128_18_w2sdcGFP.tif]

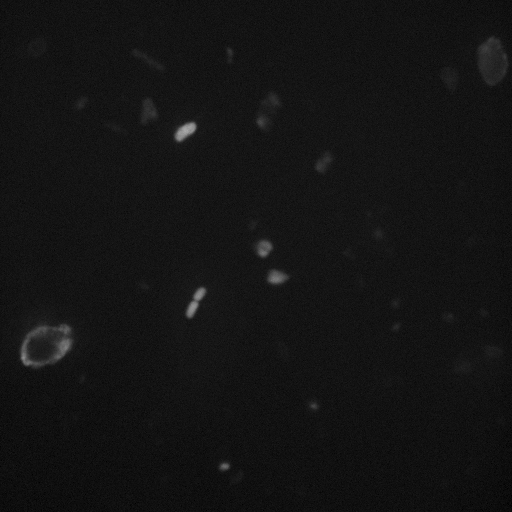

Supplement: S1 File — (ZIP) [file pcbi.1006986.s002.zip › extrait4h/4h_Z128_8_w1sdcRFP.tif]

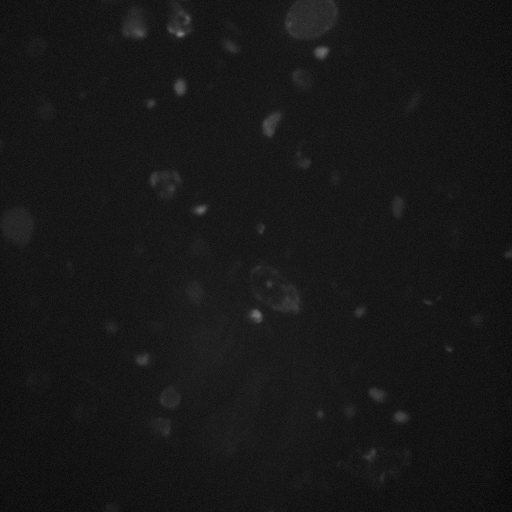

Supplement: S1 File — (ZIP) [file pcbi.1006986.s002.zip › extrait4h/4h_Z128_44_w1sdcRFP.tif]

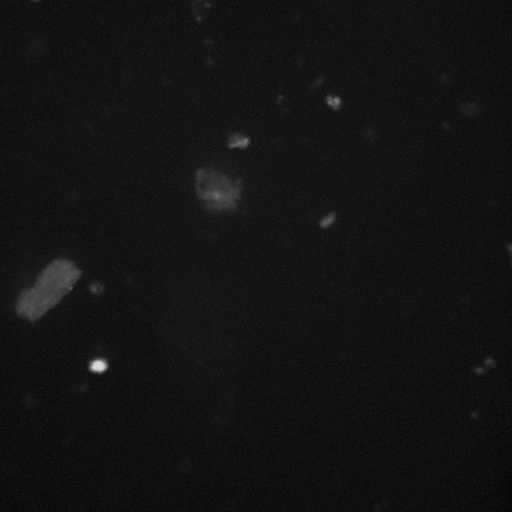

Supplement: S1 File — (ZIP) [file pcbi.1006986.s002.zip › extrait4h/4h_Z128_23_w1sdcRFP.tif]

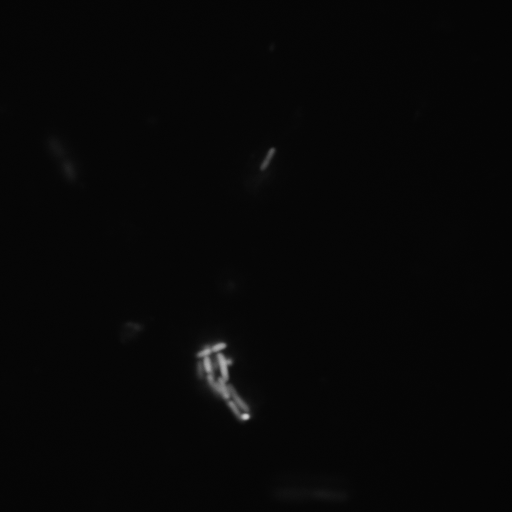

Supplement: S1 File — (ZIP) [file pcbi.1006986.s002.zip › extrait4h/4h_Z129_27_w2sdcGFP.tif]

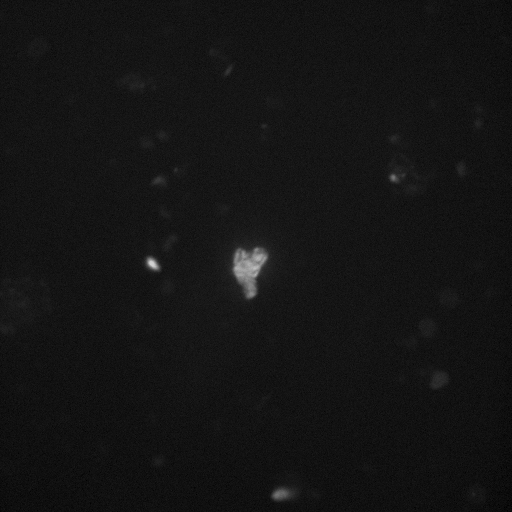

Supplement: S1 File — (ZIP) [file pcbi.1006986.s002.zip › extrait4h/4h_Z128_31_w1sdcRFP.tif]

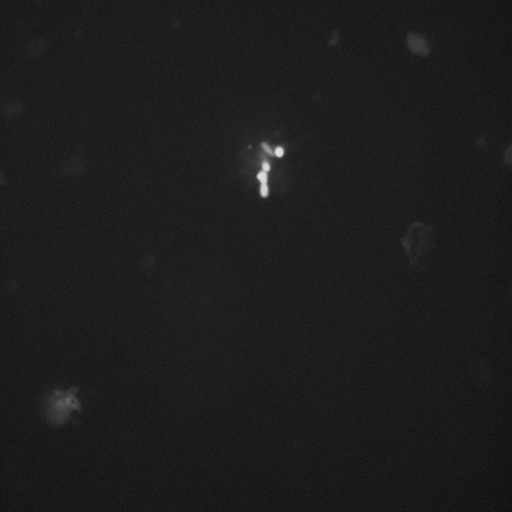

Supplement: S1 File — (ZIP) [file pcbi.1006986.s002.zip › extrait4h/4h_Z129_1_w1sdcRFP.tif]

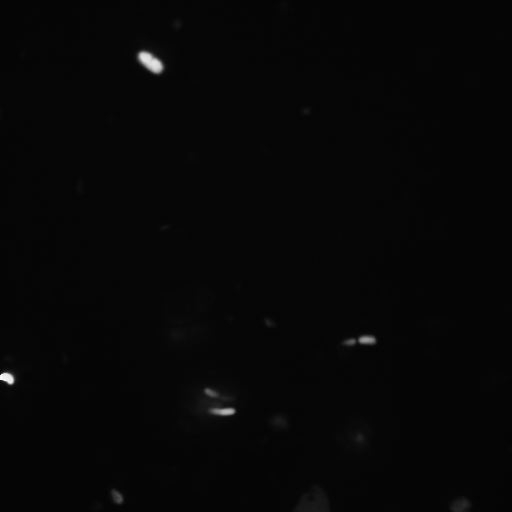

Supplement: S1 File — (ZIP) [file pcbi.1006986.s002.zip › extrait4h/4h_Z129_29_w1sdcRFP.tif]

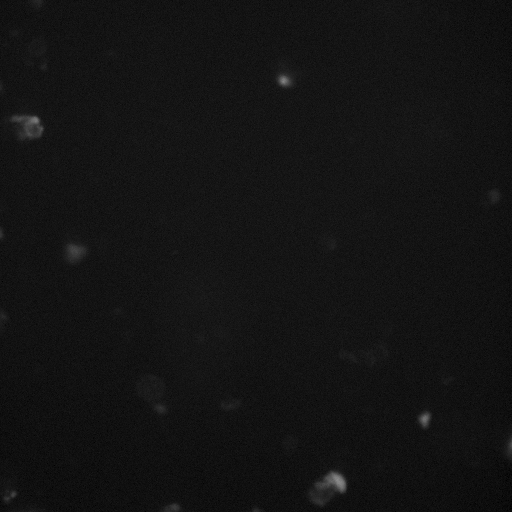

Supplement: S1 File — (ZIP) [file pcbi.1006986.s002.zip › extrait4h/4h_Z128_30_w1sdcRFP.tif]

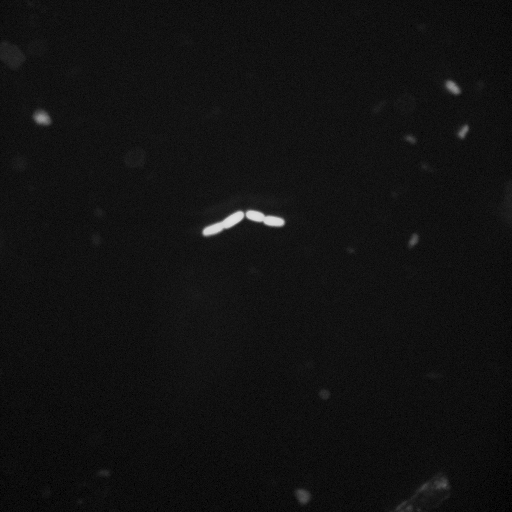

Supplement: S1 File — (ZIP) [file pcbi.1006986.s002.zip › extrait4h/4h_Z128_20_w1sdcRFP.tif]

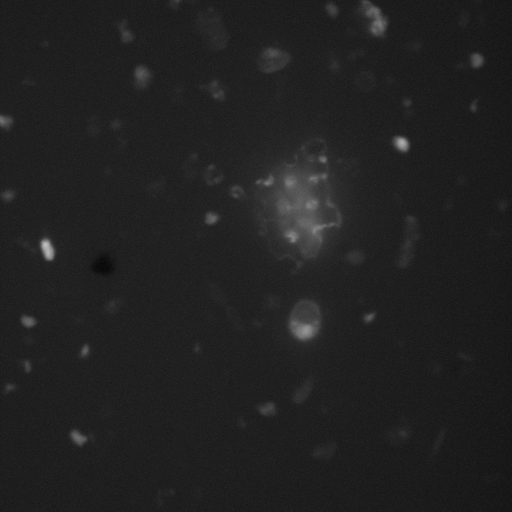

Supplement: S1 File — (ZIP) [file pcbi.1006986.s002.zip › extrait4h/4h_Z125_14_w2sdcGFP.tif]

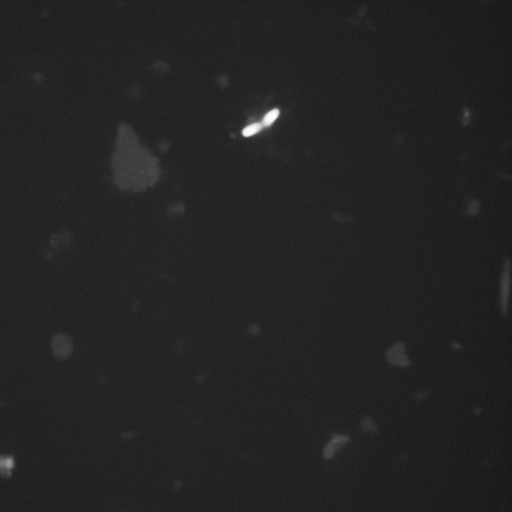

Supplement: S1 File — (ZIP) [file pcbi.1006986.s002.zip › extrait4h/4h_Z129_8_w2sdcGFP.tif]

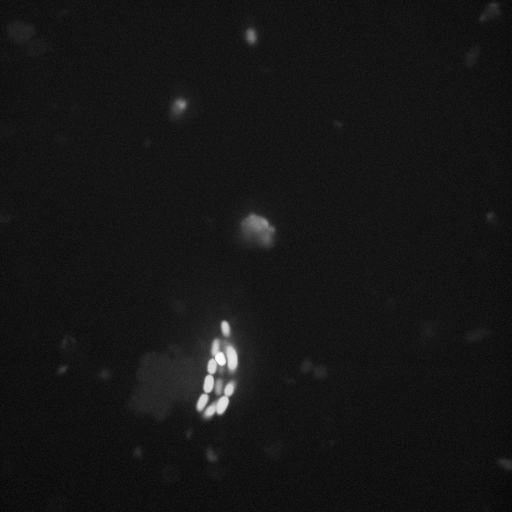

Supplement: S1 File — (ZIP) [file pcbi.1006986.s002.zip › extrait4h/4h_Z128_42_w1sdcRFP.tif]

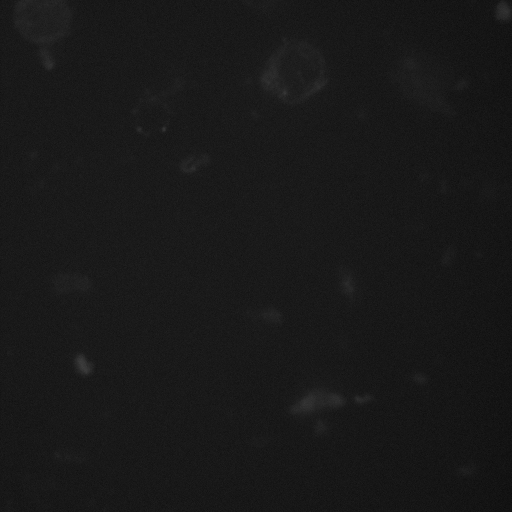

Supplement: S1 File — (ZIP) [file pcbi.1006986.s002.zip › extrait4h/4h_Z128_1_w2sdcGFP.tif]

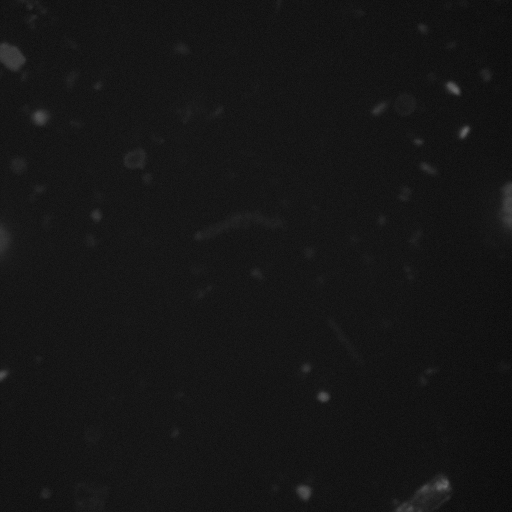

Supplement: S1 File — (ZIP) [file pcbi.1006986.s002.zip › extrait4h/4h_Z128_20_w2sdcGFP.tif]

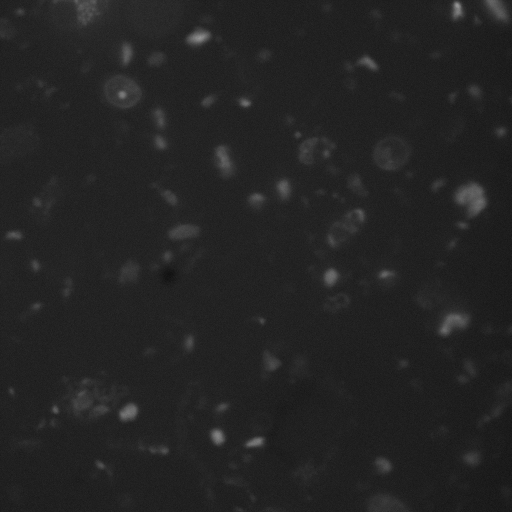

Supplement: S1 File — (ZIP) [file pcbi.1006986.s002.zip › extrait4h/4h_Z125_27_w2sdcGFP.tif]

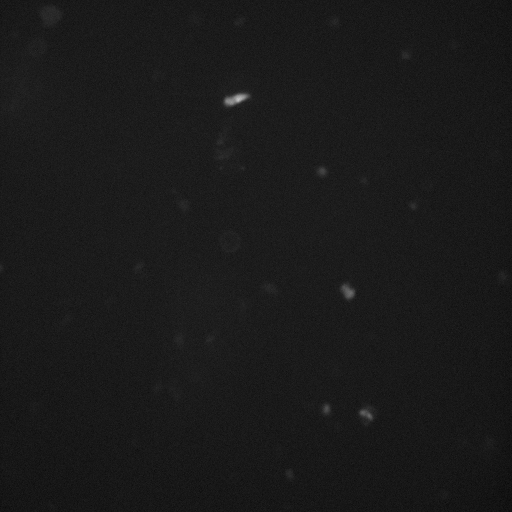

Supplement: S1 File — (ZIP) [file pcbi.1006986.s002.zip › extrait4h/4h_Z129_9_w1sdcRFP.tif]

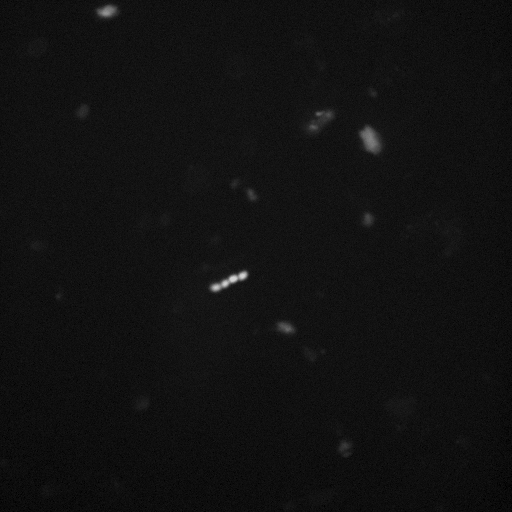

Supplement: S1 File — (ZIP) [file pcbi.1006986.s002.zip › extrait4h/4h_Z128_27_w1sdcRFP.tif]

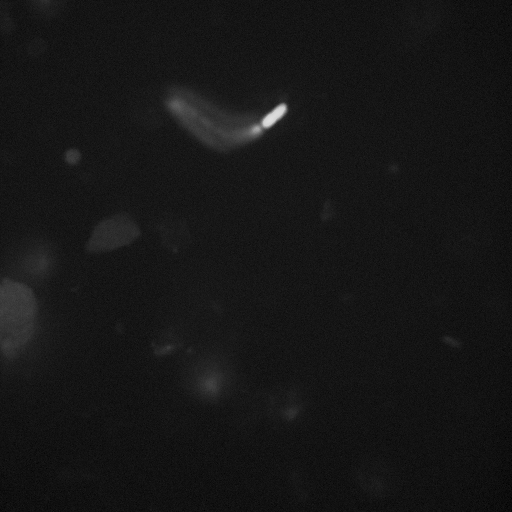

Supplement: S1 File — (ZIP) [file pcbi.1006986.s002.zip › extrait4h/4h_Z128_32_w1sdcRFP.tif]

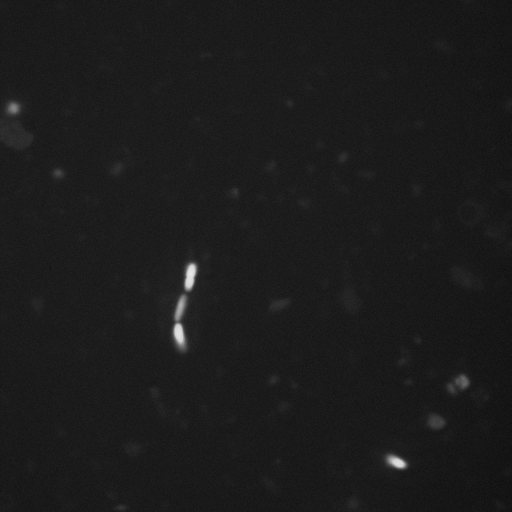

Supplement: S1 File — (ZIP) [file pcbi.1006986.s002.zip › extrait4h/4h_Z129_18_w2sdcGFP.tif]

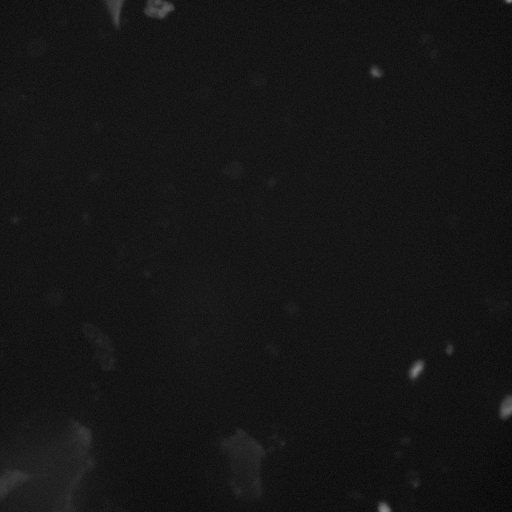

Supplement: S1 File — (ZIP) [file pcbi.1006986.s002.zip › extrait4h/4h_Z128_15_w1sdcRFP.tif]

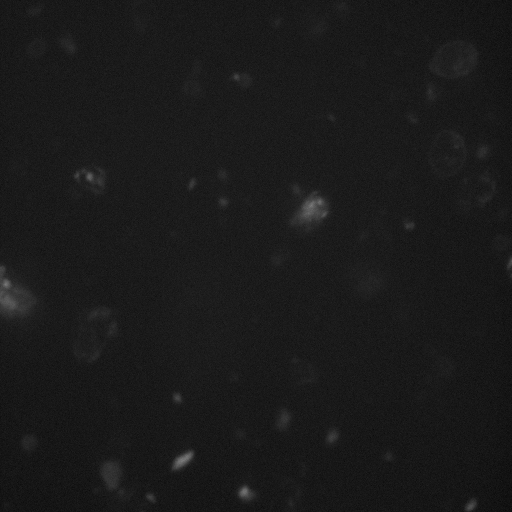

Supplement: S1 File — (ZIP) [file pcbi.1006986.s002.zip › extrait4h/4h_Z125_12_w1sdcRFP.tif]

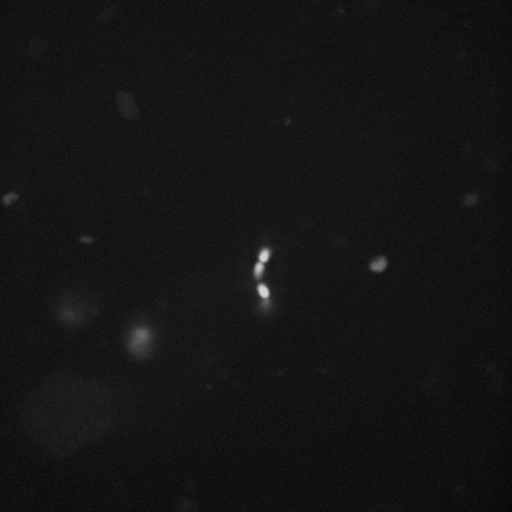

Supplement: S1 File — (ZIP) [file pcbi.1006986.s002.zip › extrait4h/4h_Z128_10_w1sdcRFP.tif]

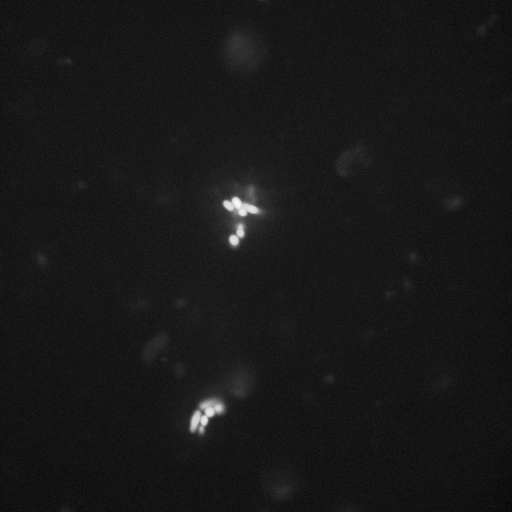

Supplement: S1 File — (ZIP) [file pcbi.1006986.s002.zip › extrait4h/4h_Z129_22_w1sdcRFP.tif]

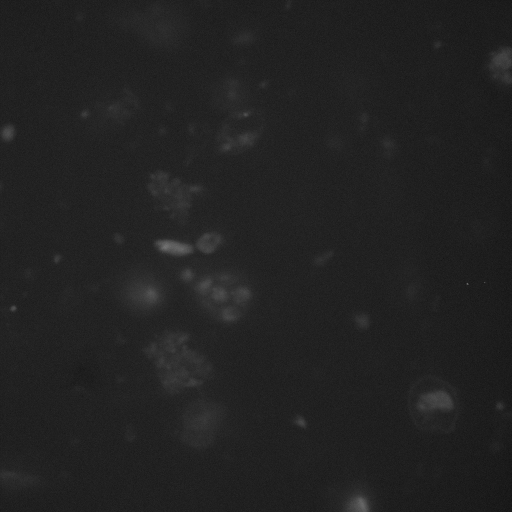

Supplement: S1 File — (ZIP) [file pcbi.1006986.s002.zip › extrait4h/4h_Z125_10_w2sdcGFP.tif]

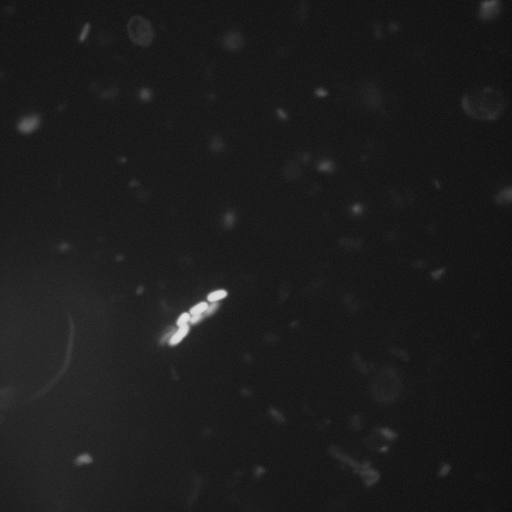

Supplement: S1 File — (ZIP) [file pcbi.1006986.s002.zip › extrait4h/4h_Z125_19_w2sdcGFP.tif]

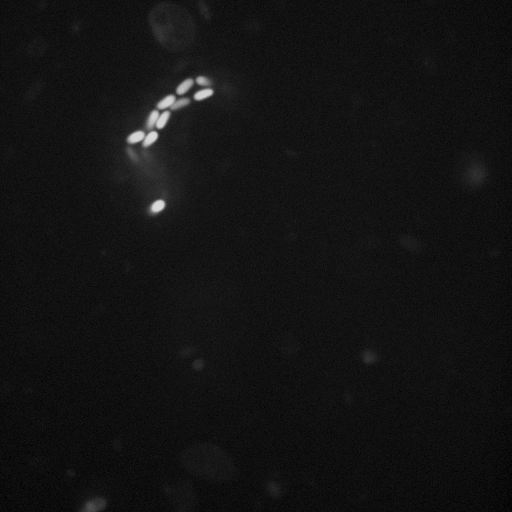

Supplement: S1 File — (ZIP) [file pcbi.1006986.s002.zip › extrait4h/4h_Z128_46_w1sdcRFP.tif]

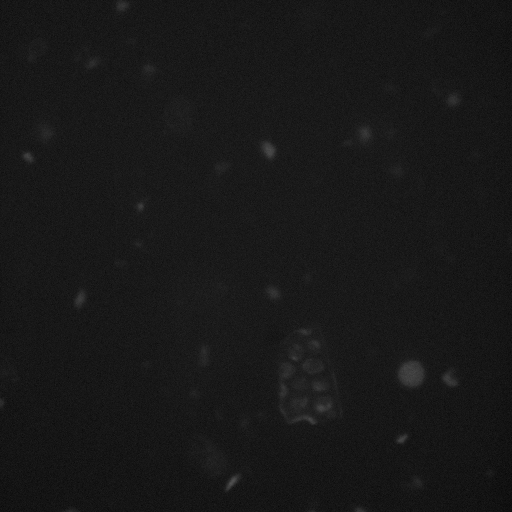

Supplement: S1 File — (ZIP) [file pcbi.1006986.s002.zip › extrait4h/4h_Z125_5_w1sdcRFP.tif]

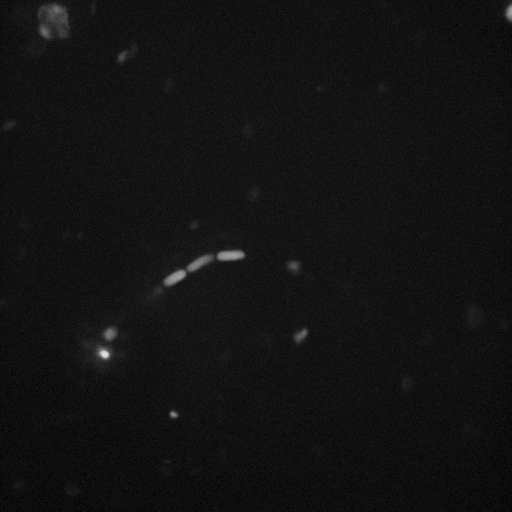

Supplement: S1 File — (ZIP) [file pcbi.1006986.s002.zip › extrait4h/4h_Z128_37_w1sdcRFP.tif]

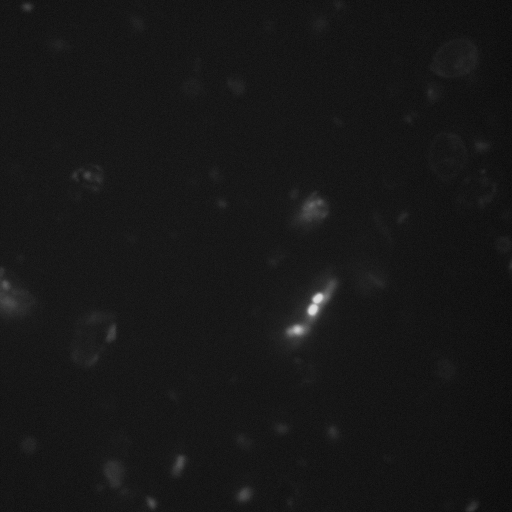

Supplement: S1 File — (ZIP) [file pcbi.1006986.s002.zip › extrait4h/4h_Z125_12_w2sdcGFP.tif]

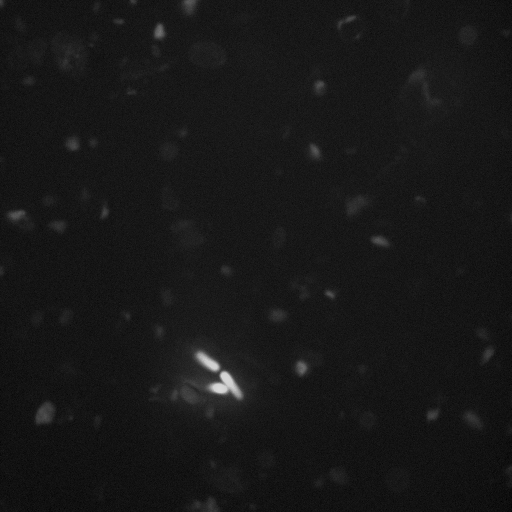

Supplement: S1 File — (ZIP) [file pcbi.1006986.s002.zip › extrait4h/4h_Z125_28_w1sdcRFP.tif]

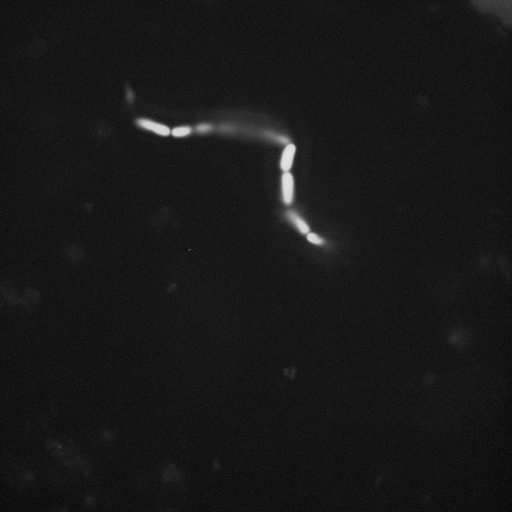

Supplement: S1 File — (ZIP) [file pcbi.1006986.s002.zip › extrait4h/4h_Z125_18_w1sdcRFP.tif]

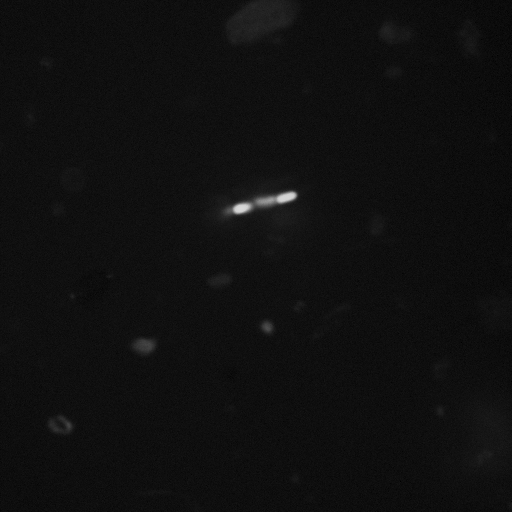

Supplement: S1 File — (ZIP) [file pcbi.1006986.s002.zip › extrait4h/4h_Z128_12_w2sdcGFP.tif]

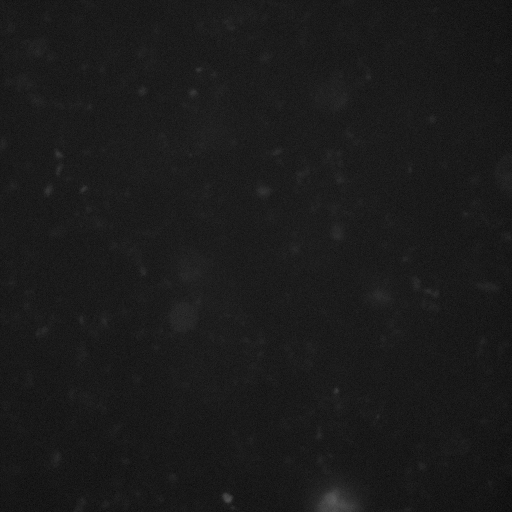

Supplement: S1 File — (ZIP) [file pcbi.1006986.s002.zip › extrait4h/4h_Z125_25_w1sdcRFP.tif]

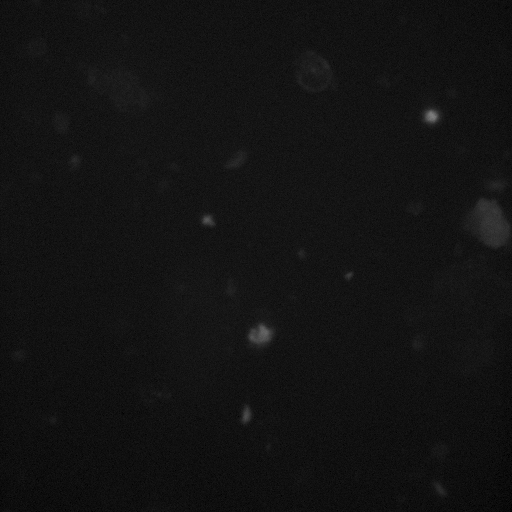

Supplement: S1 File — (ZIP) [file pcbi.1006986.s002.zip › extrait4h/4h_Z128_22_w1sdcRFP.tif]

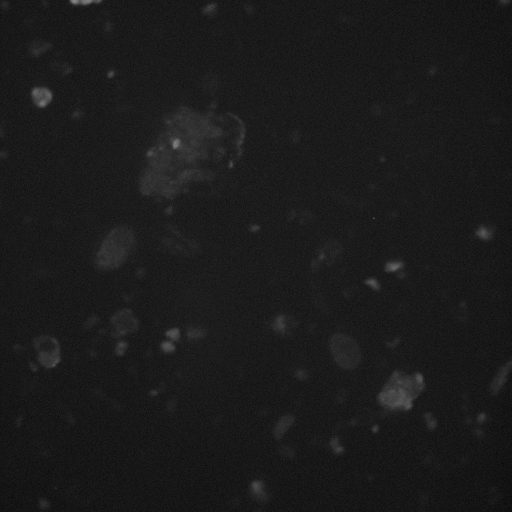

Supplement: S1 File — (ZIP) [file pcbi.1006986.s002.zip › extrait4h/4h_Z125_31_w1sdcRFP.tif]

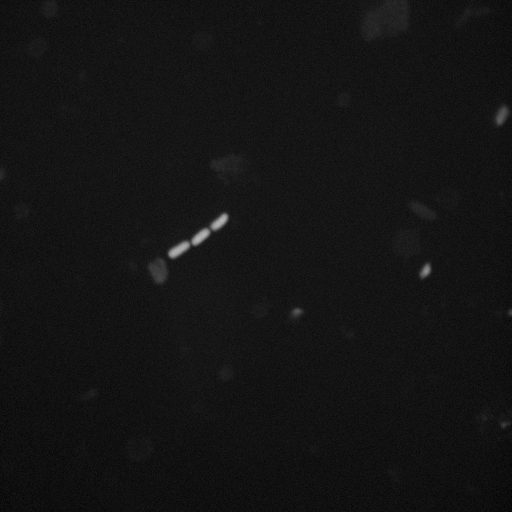

Supplement: S1 File — (ZIP) [file pcbi.1006986.s002.zip › extrait4h/4h_Z128_7_w1sdcRFP.tif]

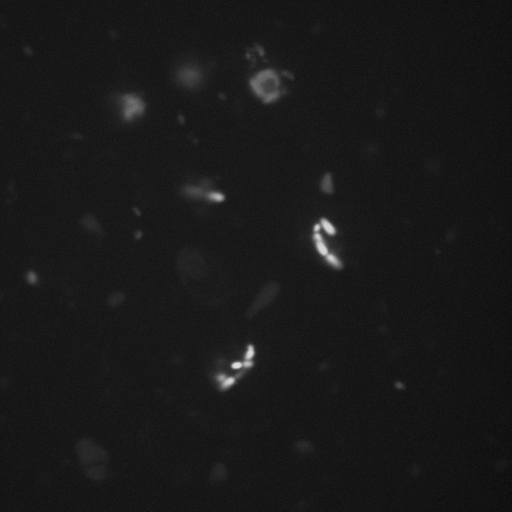

Supplement: S1 File — (ZIP) [file pcbi.1006986.s002.zip › extrait4h/4h_Z129_36_w2sdcGFP.tif]

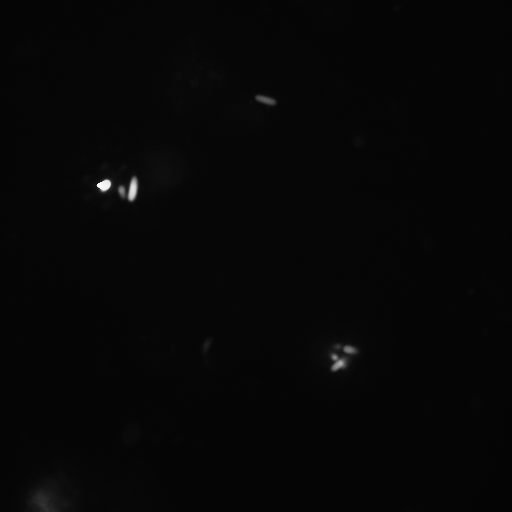

Supplement: S1 File — (ZIP) [file pcbi.1006986.s002.zip › extrait4h/4h_Z129_17_w1sdcRFP.tif]

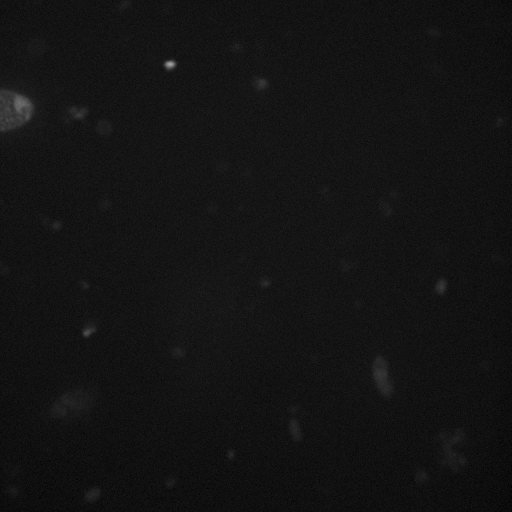

Supplement: S1 File — (ZIP) [file pcbi.1006986.s002.zip › extrait4h/4h_Z128_24_w1sdcRFP.tif]

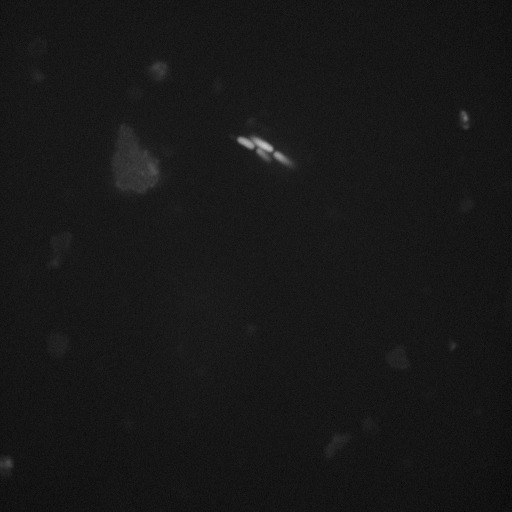

Supplement: S1 File — (ZIP) [file pcbi.1006986.s002.zip › extrait4h/4h_Z129_8_w1sdcRFP.tif]

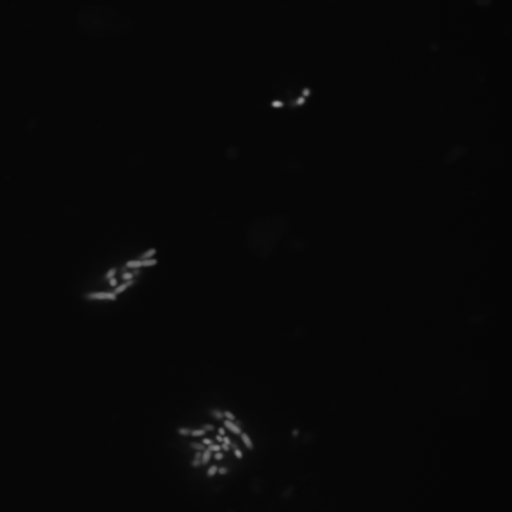

Supplement: S1 File — (ZIP) [file pcbi.1006986.s002.zip › extrait4h/4h_Z129_21_w2sdcGFP.tif]

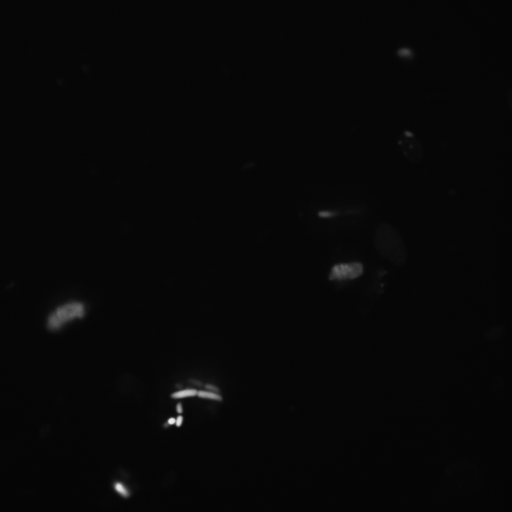

Supplement: S1 File — (ZIP) [file pcbi.1006986.s002.zip › extrait4h/4h_Z129_12_w1sdcRFP.tif]

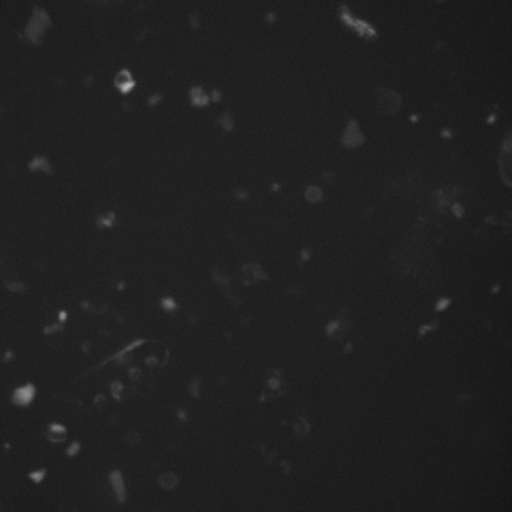

Supplement: S1 File — (ZIP) [file pcbi.1006986.s002.zip › extrait4h/4h_Z125_11_w2sdcGFP.tif]

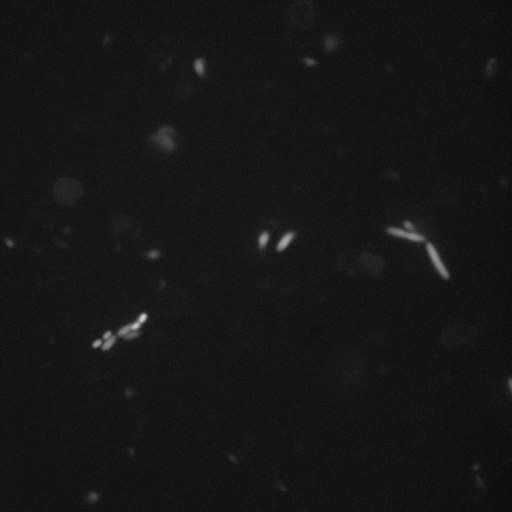

Supplement: S1 File — (ZIP) [file pcbi.1006986.s002.zip › extrait4h/4h_Z129_37_w2sdcGFP.tif]

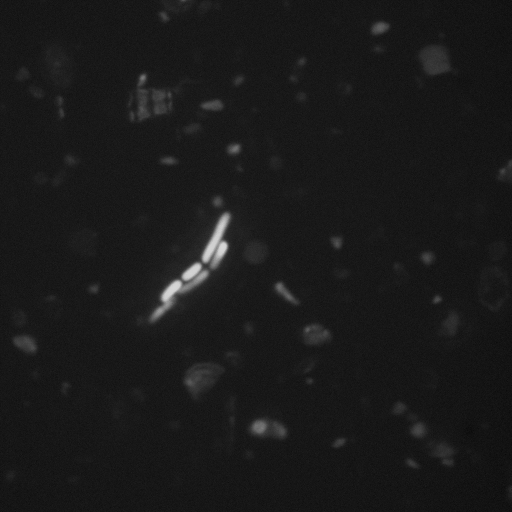

Supplement: S1 File — (ZIP) [file pcbi.1006986.s002.zip › extrait4h/4h_Z125_24_w2sdcGFP.tif]

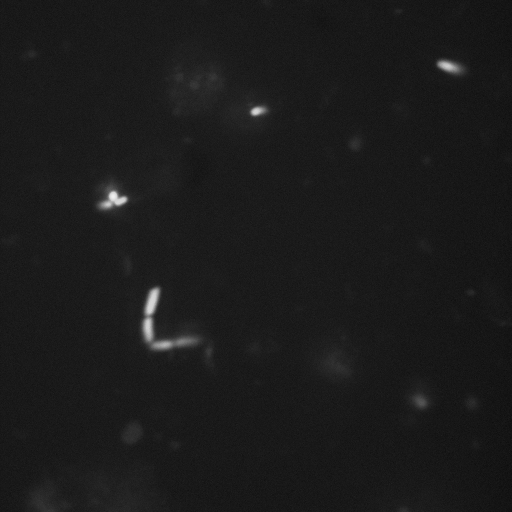

Supplement: S1 File — (ZIP) [file pcbi.1006986.s002.zip › extrait4h/4h_Z129_17_w2sdcGFP.tif]

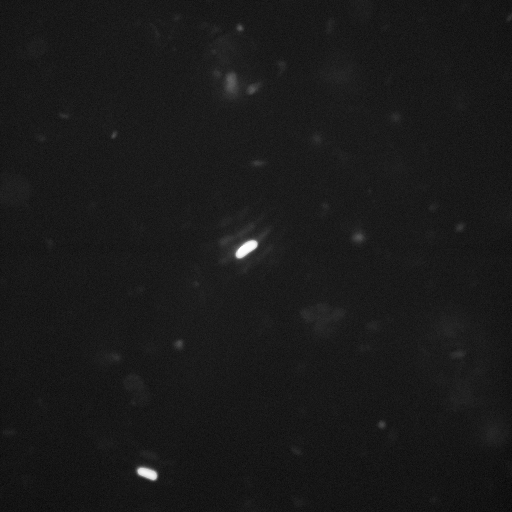

Supplement: S1 File — (ZIP) [file pcbi.1006986.s002.zip › extrait4h/4h_Z125_9_w1sdcRFP.tif]

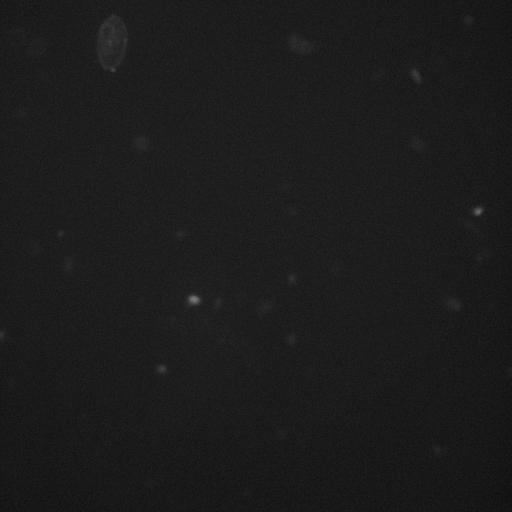

Supplement: S1 File — (ZIP) [file pcbi.1006986.s002.zip › extrait4h/4h_Z129_5_w1sdcRFP.tif]

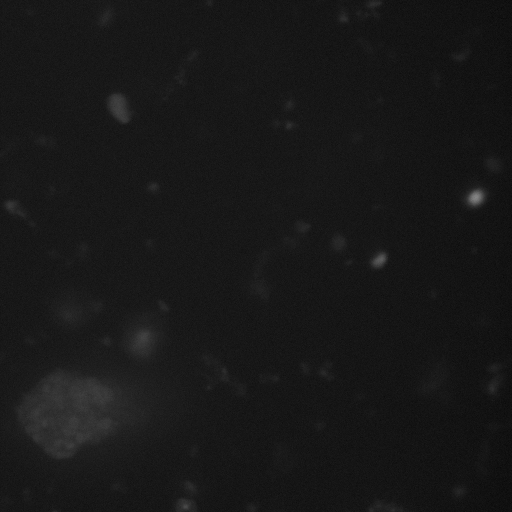

Supplement: S1 File — (ZIP) [file pcbi.1006986.s002.zip › extrait4h/4h_Z128_10_w2sdcGFP.tif]

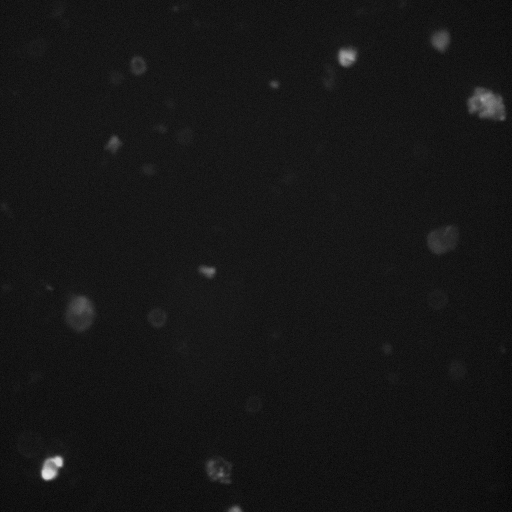

Supplement: S1 File — (ZIP) [file pcbi.1006986.s002.zip › extrait4h/4h_Z128_13_w1sdcRFP.tif]

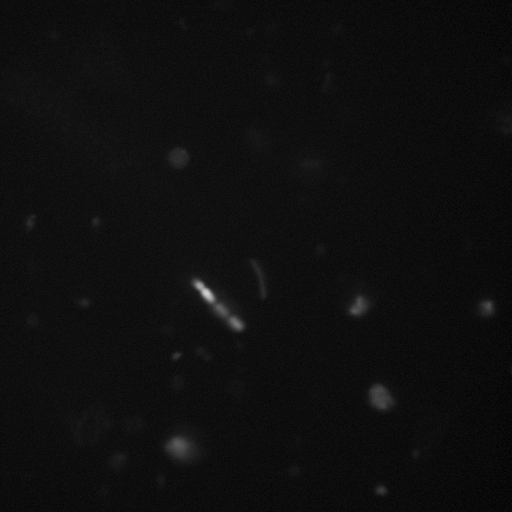

Supplement: S1 File — (ZIP) [file pcbi.1006986.s002.zip › extrait4h/4h_Z128_16_w2sdcGFP.tif]

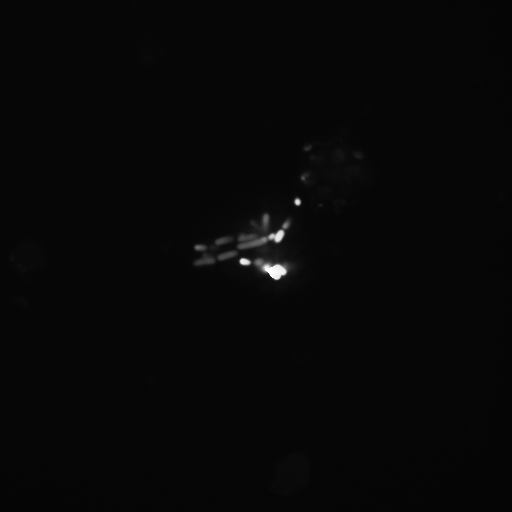

Supplement: S1 File — (ZIP) [file pcbi.1006986.s002.zip › extrait4h/4h_Z129_7_w1sdcRFP.tif]

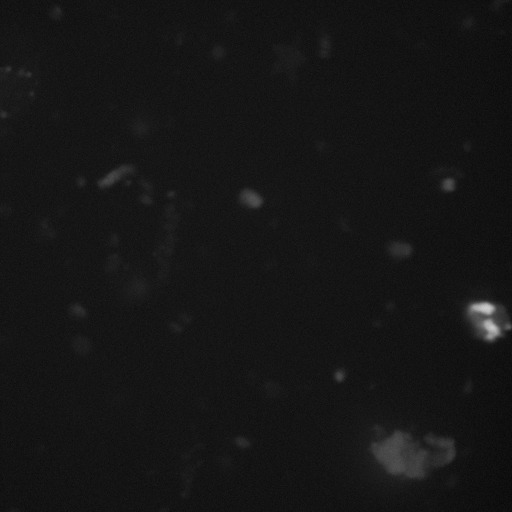

Supplement: S1 File — (ZIP) [file pcbi.1006986.s002.zip › extrait4h/4h_Z128_39_w2sdcGFP.tif]

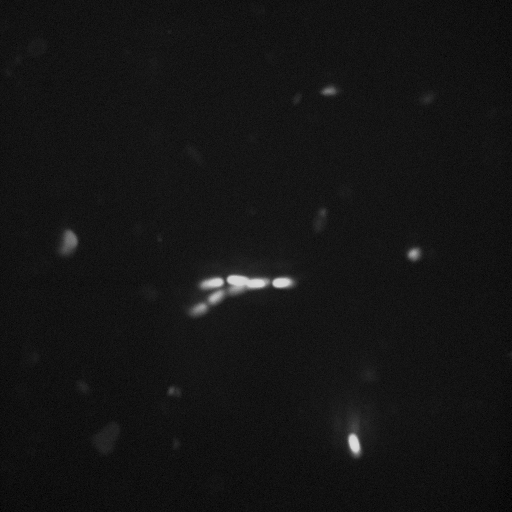

Supplement: S1 File — (ZIP) [file pcbi.1006986.s002.zip › extrait4h/4h_Z128_41_w1sdcRFP.tif]

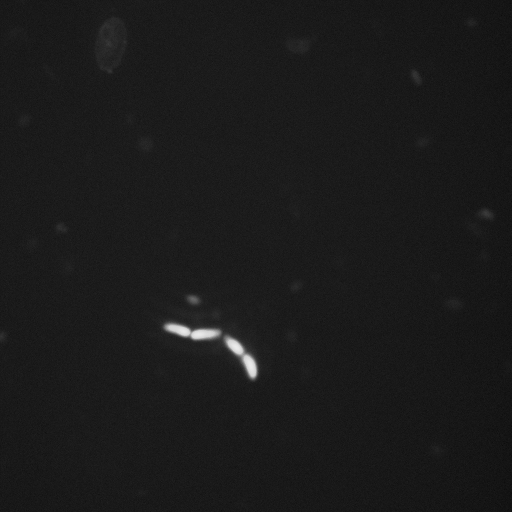

Supplement: S1 File — (ZIP) [file pcbi.1006986.s002.zip › extrait4h/4h_Z129_5_w2sdcGFP.tif]

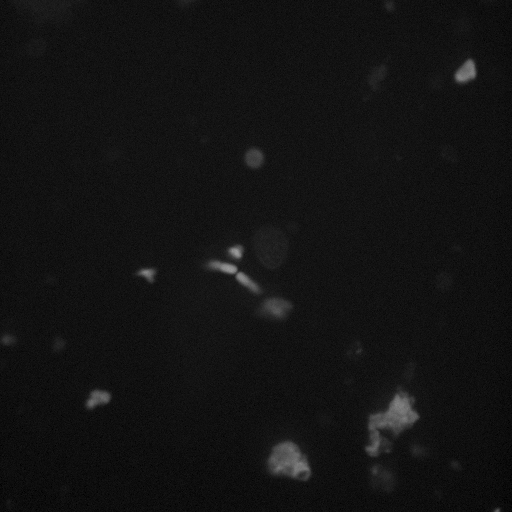

Supplement: S1 File — (ZIP) [file pcbi.1006986.s002.zip › extrait4h/4h_Z128_26_w1sdcRFP.tif]

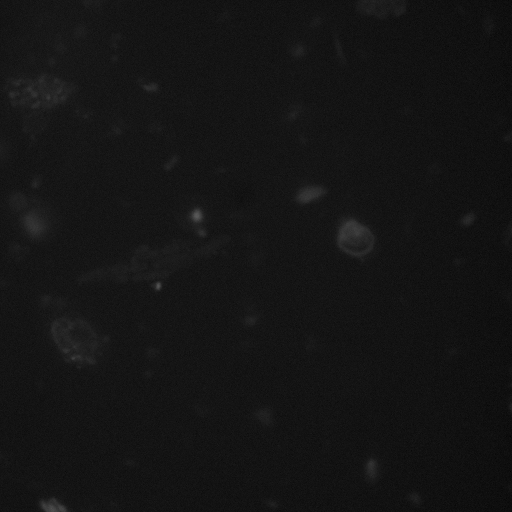

Supplement: S1 File — (ZIP) [file pcbi.1006986.s002.zip › extrait4h/4h_Z128_36_w2sdcGFP.tif]

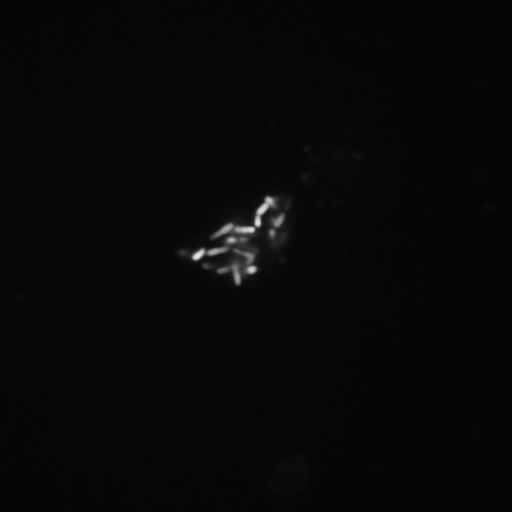

Supplement: S1 File — (ZIP) [file pcbi.1006986.s002.zip › extrait4h/4h_Z129_7_w2sdcGFP.tif]

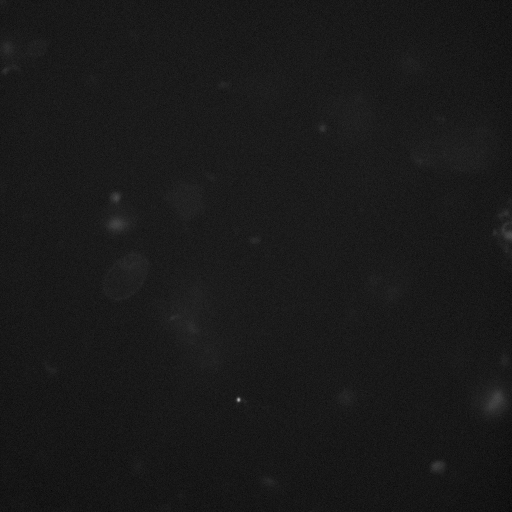

Supplement: S1 File — (ZIP) [file pcbi.1006986.s002.zip › extrait4h/4h_Z125_3_w1sdcRFP.tif]

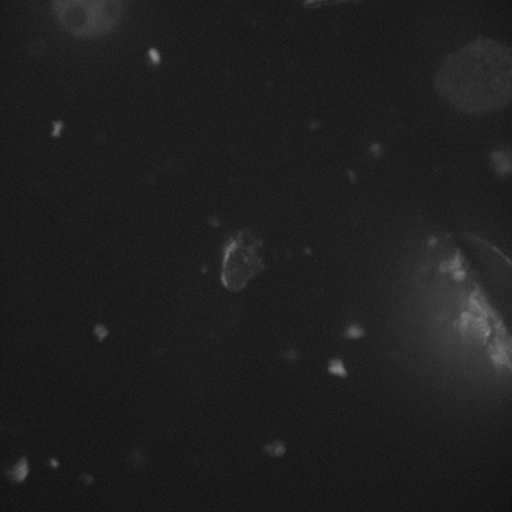

Supplement: S1 File — (ZIP) [file pcbi.1006986.s002.zip › extrait4h/4h_Z125_4_w1sdcRFP.tif]

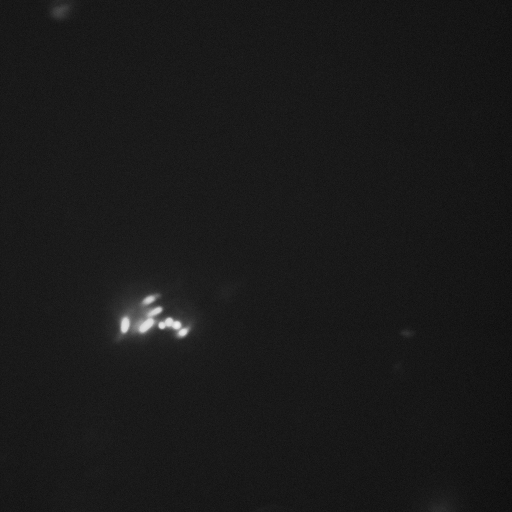

Supplement: S1 File — (ZIP) [file pcbi.1006986.s002.zip › extrait4h/4h_Z129_2_w2sdcGFP.tif]

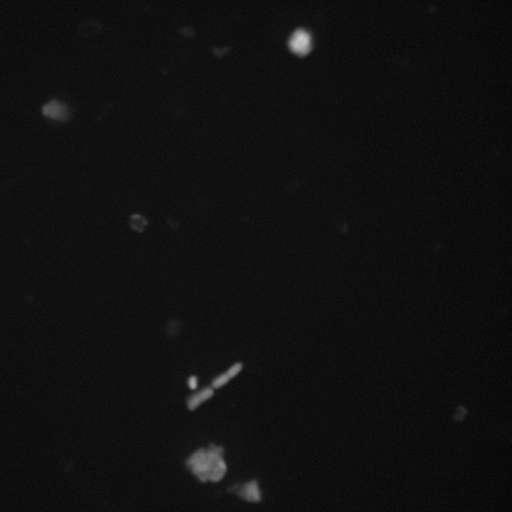

Supplement: S1 File — (ZIP) [file pcbi.1006986.s002.zip › extrait4h/4h_Z128_3_w2sdcGFP.tif]

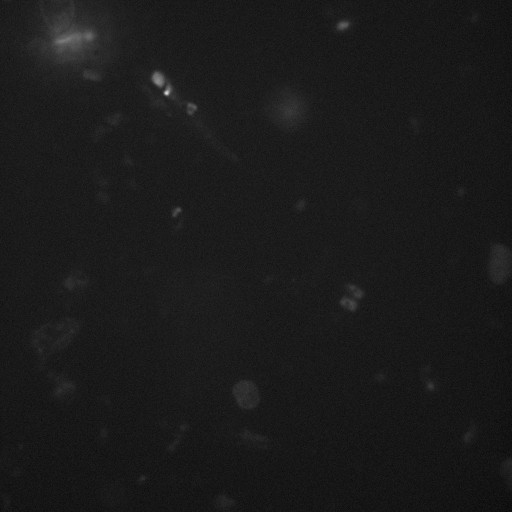

Supplement: S1 File — (ZIP) [file pcbi.1006986.s002.zip › extrait4h/4h_Z129_15_w1sdcRFP.tif]

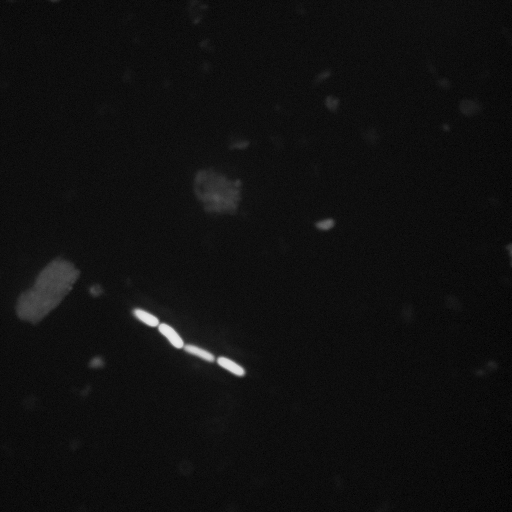

Supplement: S1 File — (ZIP) [file pcbi.1006986.s002.zip › extrait4h/4h_Z128_23_w2sdcGFP.tif]

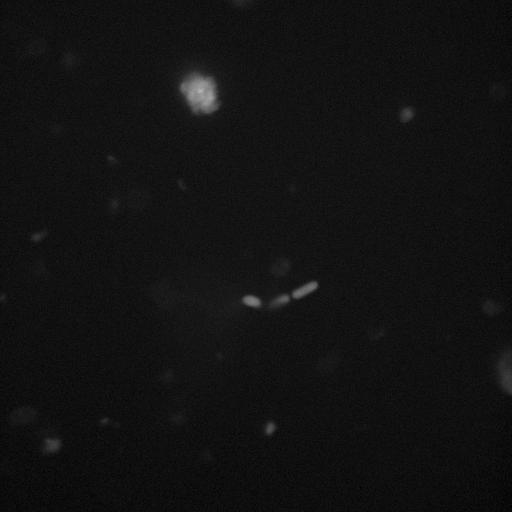

Supplement: S1 File — (ZIP) [file pcbi.1006986.s002.zip › extrait4h/4h_Z128_29_w1sdcRFP.tif]

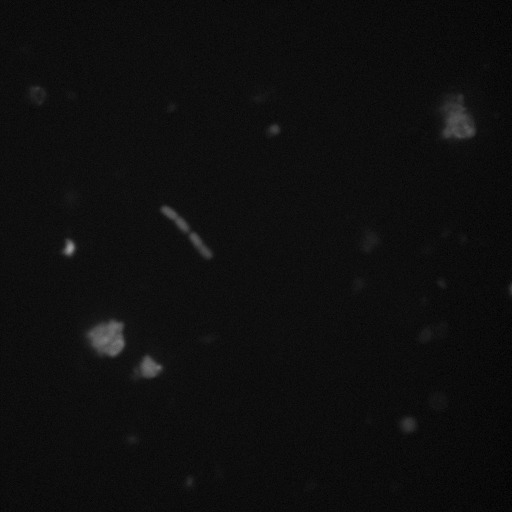

Supplement: S1 File — (ZIP) [file pcbi.1006986.s002.zip › extrait4h/4h_Z128_4_w2sdcGFP.tif]

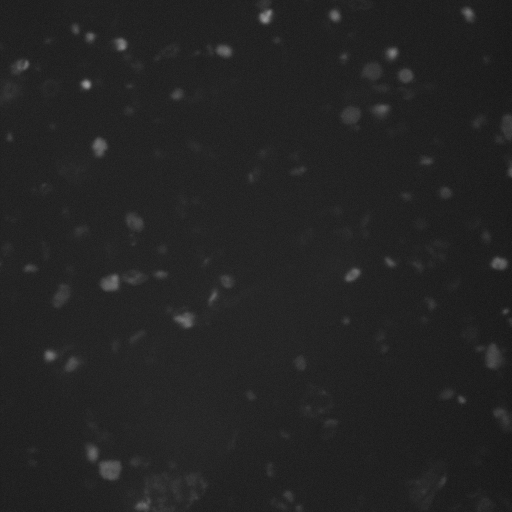

Supplement: S1 File — (ZIP) [file pcbi.1006986.s002.zip › extrait4h/4h_Z125_23_w2sdcGFP.tif]

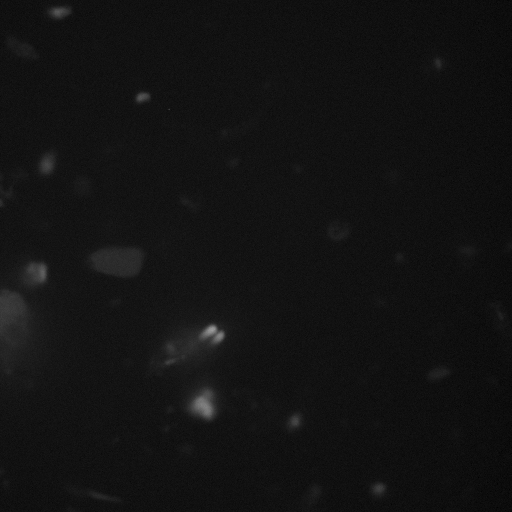

Supplement: S1 File — (ZIP) [file pcbi.1006986.s002.zip › extrait4h/4h_Z128_33_w2sdcGFP.tif]

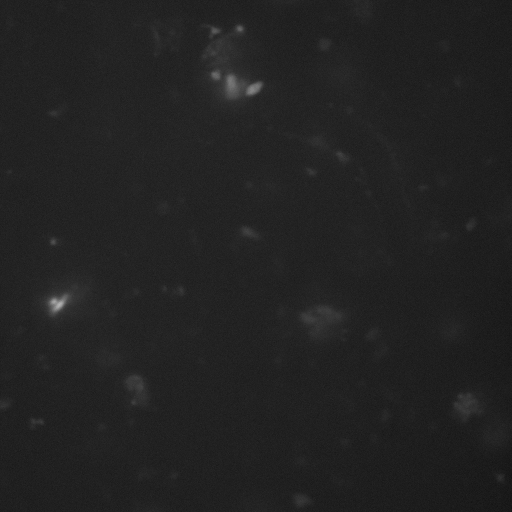

Supplement: S1 File — (ZIP) [file pcbi.1006986.s002.zip › extrait4h/4h_Z125_8_w2sdcGFP.tif]

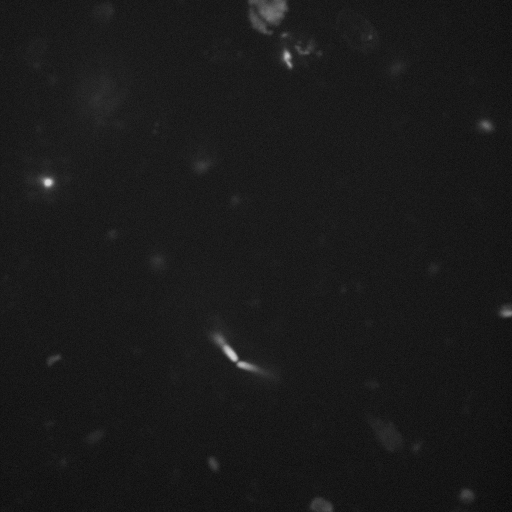

Supplement: S1 File — (ZIP) [file pcbi.1006986.s002.zip › extrait4h/4h_Z129_19_w1sdcRFP.tif]
