# Supplementary figures and images for: Enchained growth and cluster dislocation: A possible mechanism for microbiota homeostasis (part 1 of 10)
Source: PLoS Comput Biol. 2019 May 3;15(5):e1006986. doi: 10.1371/journal.pcbi.1006986 (PMC6519844; doi:10.1371/journal.pcbi.1006986)

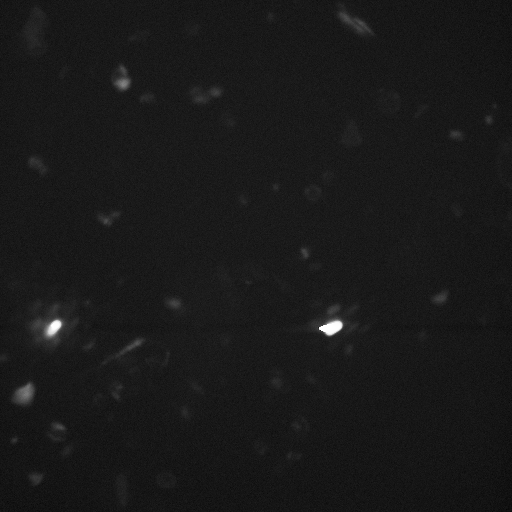

Supplement: S1 File — (ZIP) [file pcbi.1006986.s002.zip › extrait4h/4h_Z125_11_w1sdcRFP.tif]

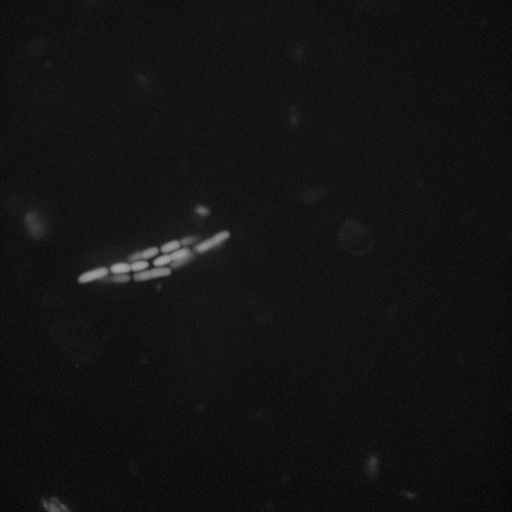

Supplement: S1 File — (ZIP) [file pcbi.1006986.s002.zip › extrait4h/4h_Z128_36_w1sdcRFP.tif]

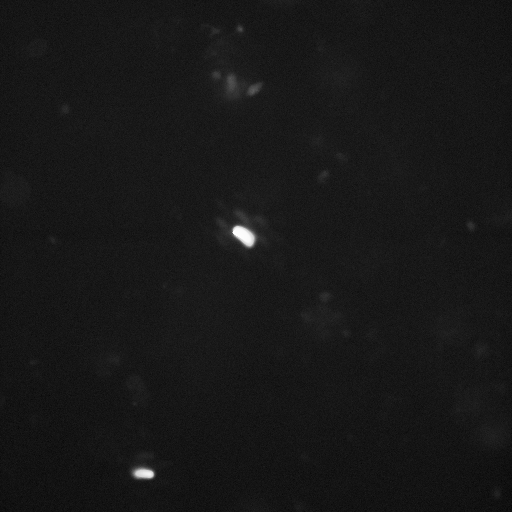

Supplement: S1 File — (ZIP) [file pcbi.1006986.s002.zip › extrait4h/4h_Z125_8_w1sdcRFP.tif]

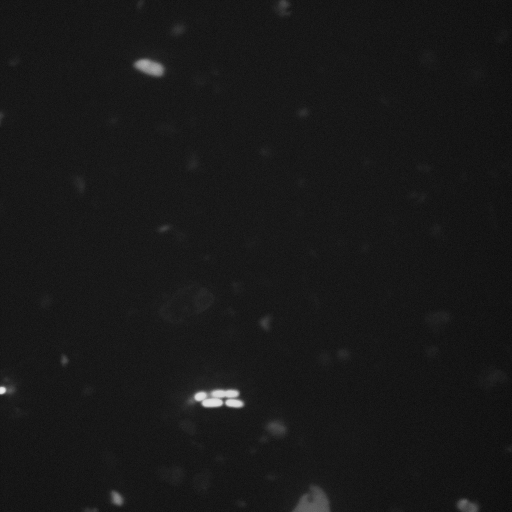

Supplement: S1 File — (ZIP) [file pcbi.1006986.s002.zip › extrait4h/4h_Z129_29_w2sdcGFP.tif]

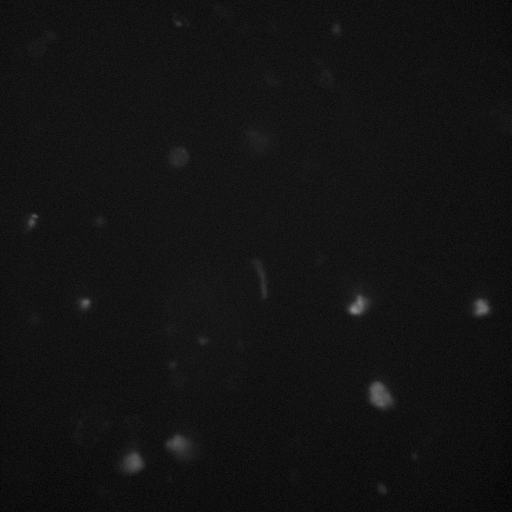

Supplement: S1 File — (ZIP) [file pcbi.1006986.s002.zip › extrait4h/4h_Z128_16_w1sdcRFP.tif]

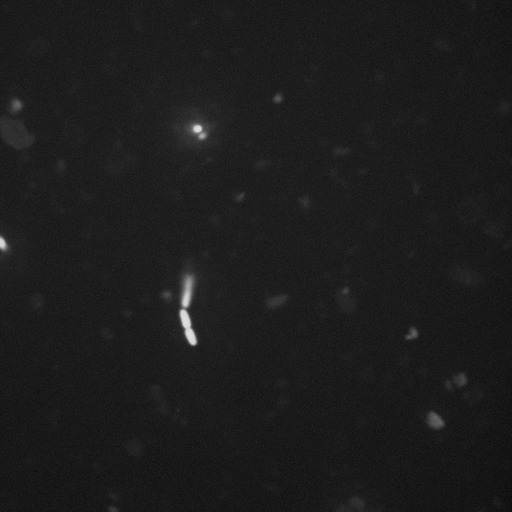

Supplement: S1 File — (ZIP) [file pcbi.1006986.s002.zip › extrait4h/4h_Z129_18_w1sdcRFP.tif]

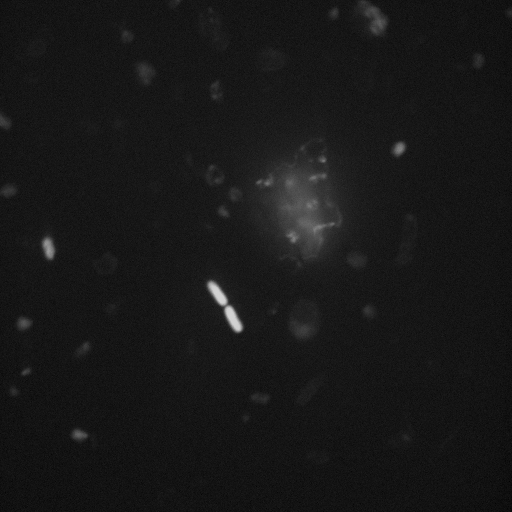

Supplement: S1 File — (ZIP) [file pcbi.1006986.s002.zip › extrait4h/4h_Z125_14_w1sdcRFP.tif]

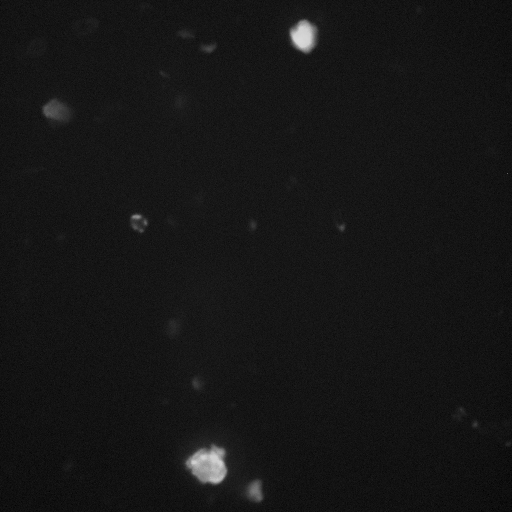

Supplement: S1 File — (ZIP) [file pcbi.1006986.s002.zip › extrait4h/4h_Z128_3_w1sdcRFP.tif]

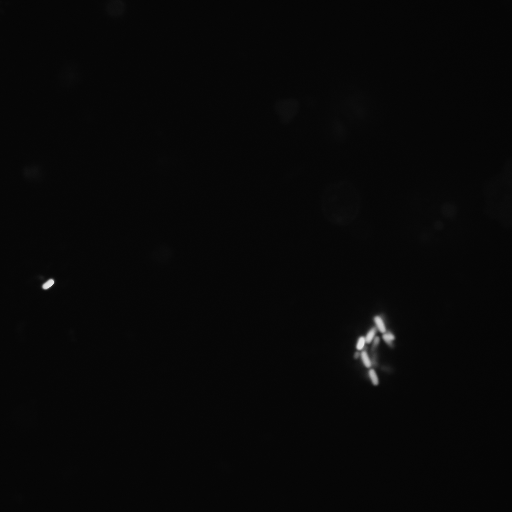

Supplement: S1 File — (ZIP) [file pcbi.1006986.s002.zip › extrait4h/4h_Z129_6_w2sdcGFP.tif]

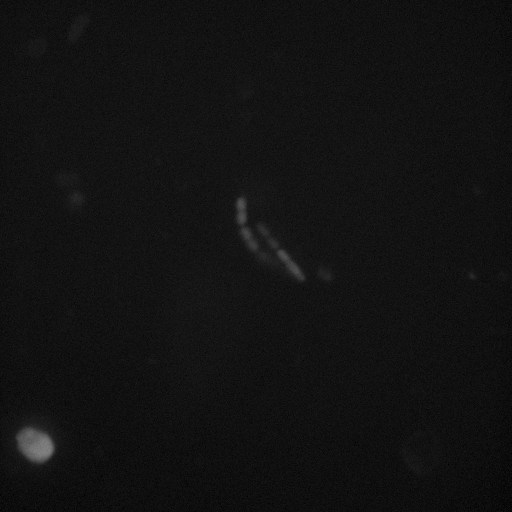

Supplement: S1 File — (ZIP) [file pcbi.1006986.s002.zip › extrait4h/4h_Z128_9_w1sdcRFP.tif]

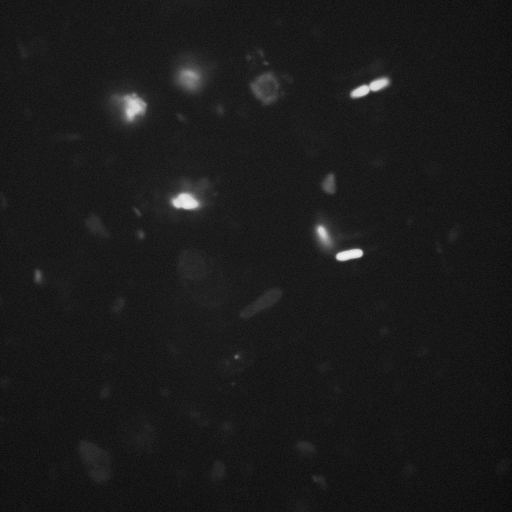

Supplement: S1 File — (ZIP) [file pcbi.1006986.s002.zip › extrait4h/4h_Z129_36_w1sdcRFP.tif]

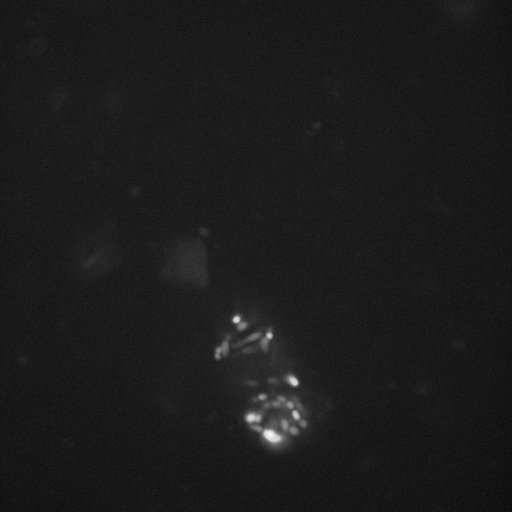

Supplement: S1 File — (ZIP) [file pcbi.1006986.s002.zip › extrait4h/4h_Z129_14_w1sdcRFP.tif]

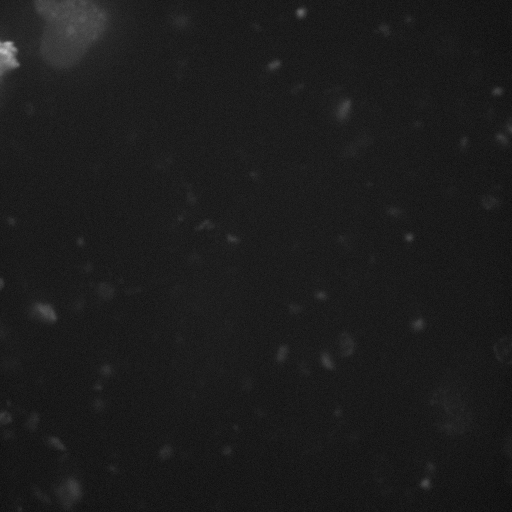

Supplement: S1 File — (ZIP) [file pcbi.1006986.s002.zip › extrait4h/4h_Z125_6_w1sdcRFP.tif]

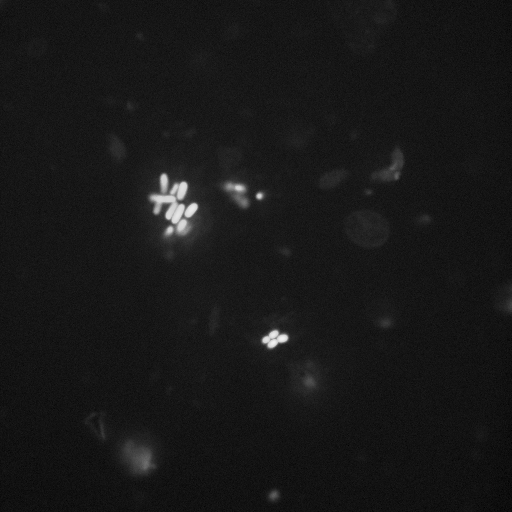

Supplement: S1 File — (ZIP) [file pcbi.1006986.s002.zip › extrait4h/4h_Z129_40_w1sdcRFP.tif]

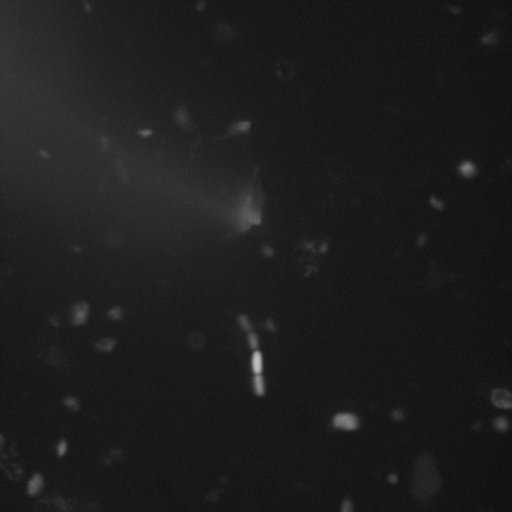

Supplement: S1 File — (ZIP) [file pcbi.1006986.s002.zip › extrait4h/4h_Z125_13_w2sdcGFP.tif]

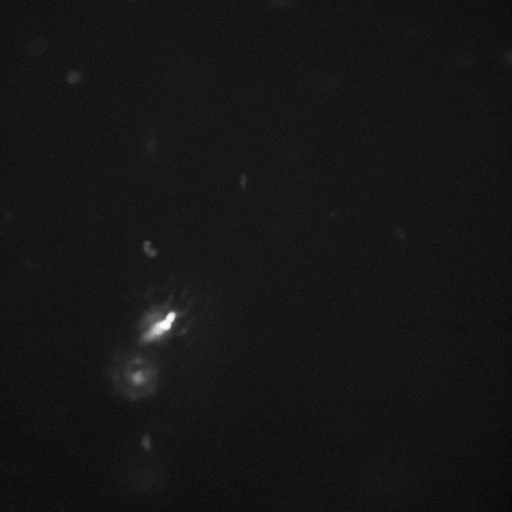

Supplement: S1 File — (ZIP) [file pcbi.1006986.s002.zip › extrait4h/4h_Z128_38_w1sdcRFP.tif]

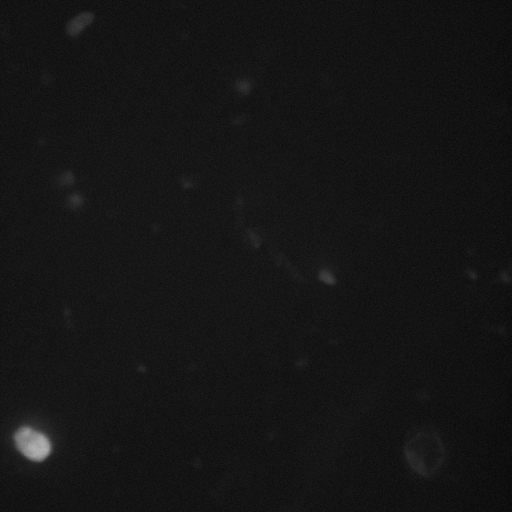

Supplement: S1 File — (ZIP) [file pcbi.1006986.s002.zip › extrait4h/4h_Z128_9_w2sdcGFP.tif]

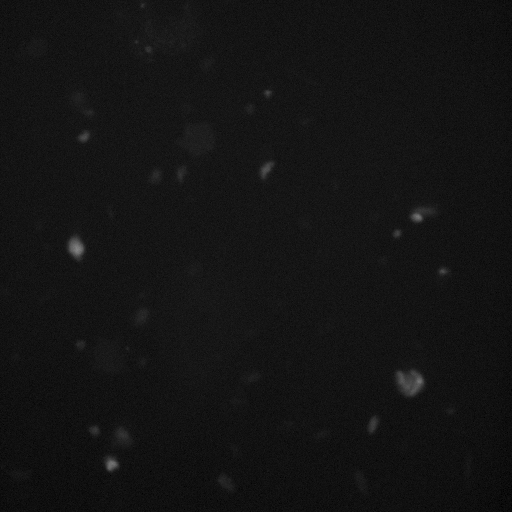

Supplement: S1 File — (ZIP) [file pcbi.1006986.s002.zip › extrait4h/4h_Z128_17_w1sdcRFP.tif]

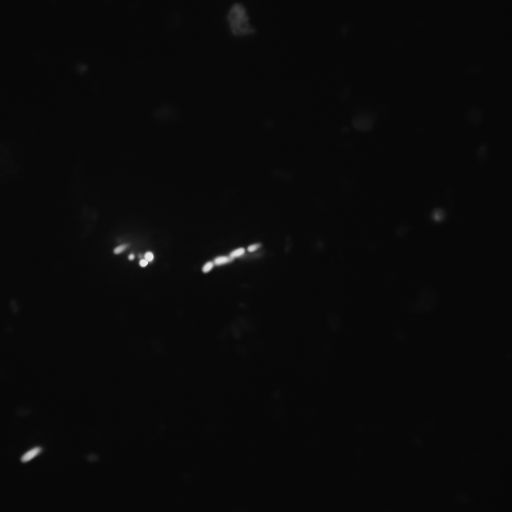

Supplement: S1 File — (ZIP) [file pcbi.1006986.s002.zip › extrait4h/4h_Z129_33_w2sdcGFP.tif]

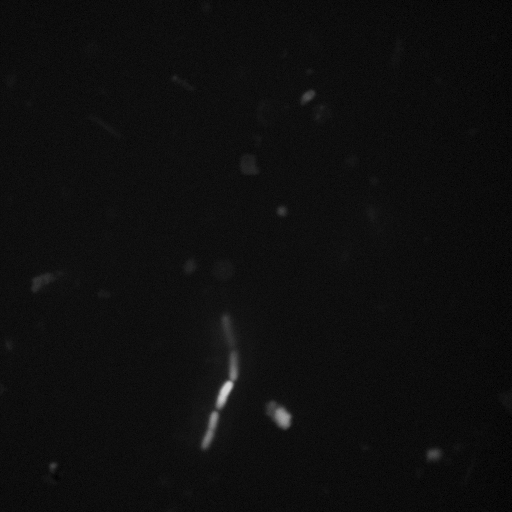

Supplement: S1 File — (ZIP) [file pcbi.1006986.s002.zip › extrait4h/4h_Z128_25_w2sdcGFP.tif]

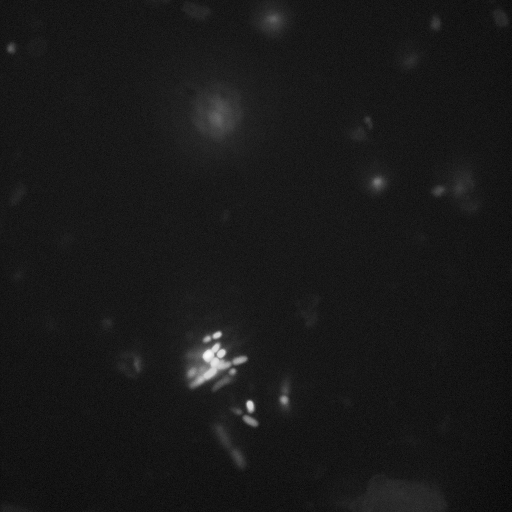

Supplement: S1 File — (ZIP) [file pcbi.1006986.s002.zip › extrait4h/4h_Z129_13_w1sdcRFP.tif]

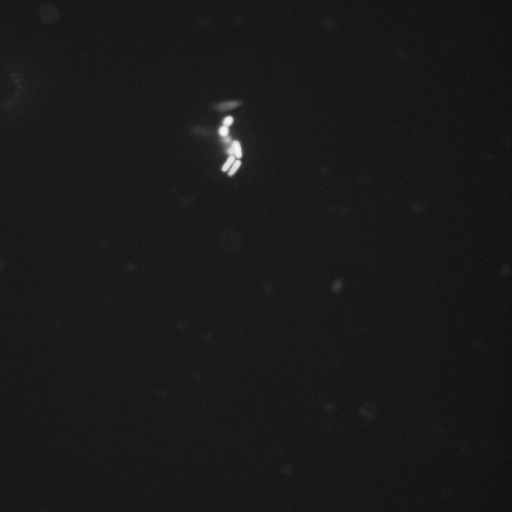

Supplement: S1 File — (ZIP) [file pcbi.1006986.s002.zip › extrait4h/4h_Z129_9_w2sdcGFP.tif]

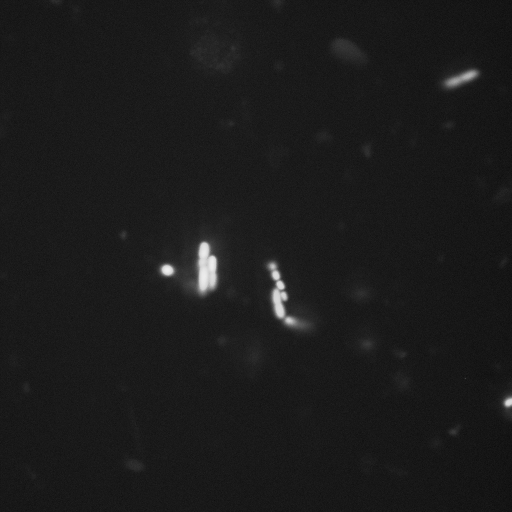

Supplement: S1 File — (ZIP) [file pcbi.1006986.s002.zip › extrait4h/4h_Z129_28_w2sdcGFP.tif]

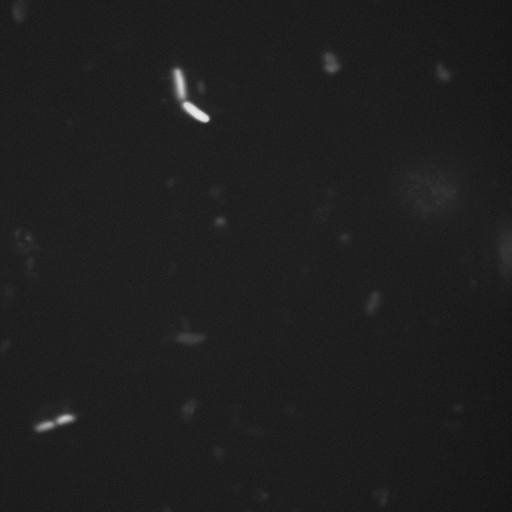

Supplement: S1 File — (ZIP) [file pcbi.1006986.s002.zip › extrait4h/4h_Z129_11_w2sdcGFP.tif]

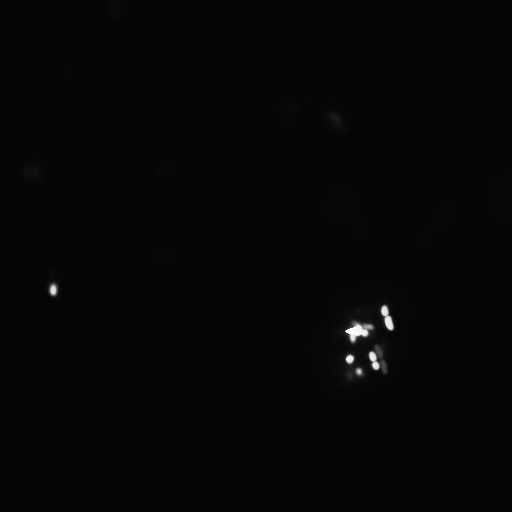

Supplement: S1 File — (ZIP) [file pcbi.1006986.s002.zip › extrait4h/4h_Z129_6_w1sdcRFP.tif]

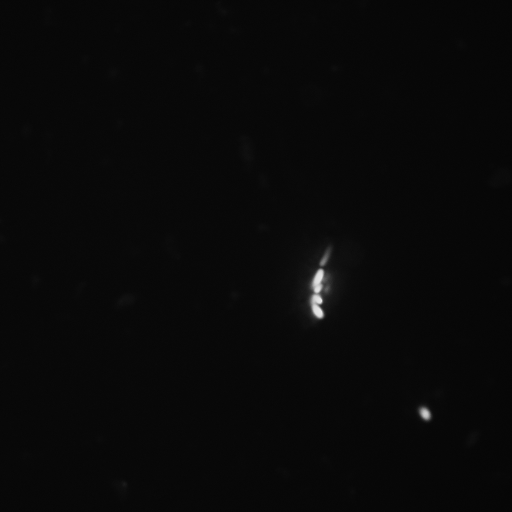

Supplement: S1 File — (ZIP) [file pcbi.1006986.s002.zip › extrait4h/4h_Z129_23_w2sdcGFP.tif]

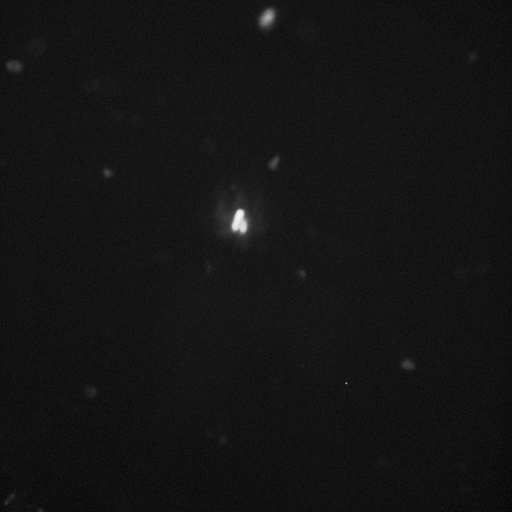

Supplement: S1 File — (ZIP) [file pcbi.1006986.s002.zip › extrait4h/4h_Z128_6_w1sdcRFP.tif]

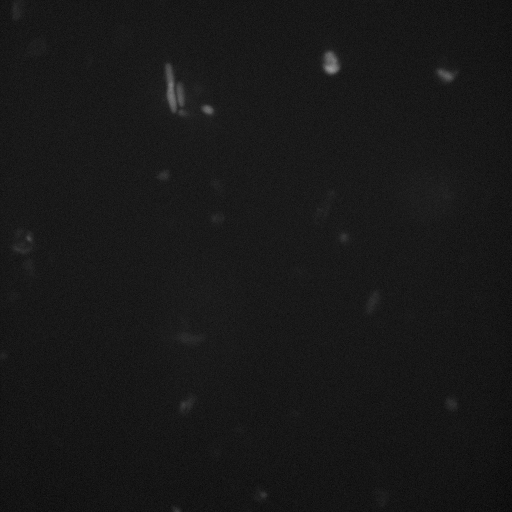

Supplement: S1 File — (ZIP) [file pcbi.1006986.s002.zip › extrait4h/4h_Z129_11_w1sdcRFP.tif]

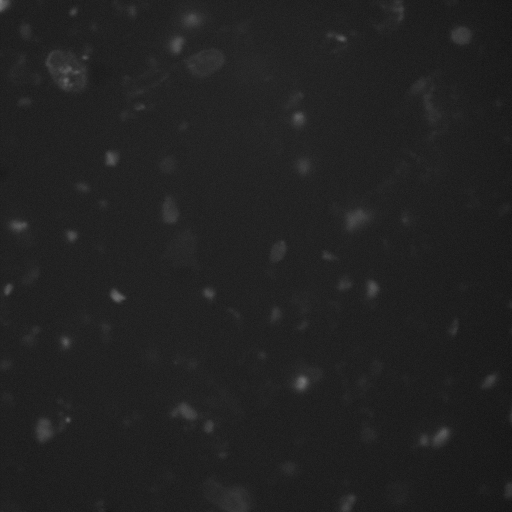

Supplement: S1 File — (ZIP) [file pcbi.1006986.s002.zip › extrait4h/4h_Z125_29_w2sdcGFP.tif]

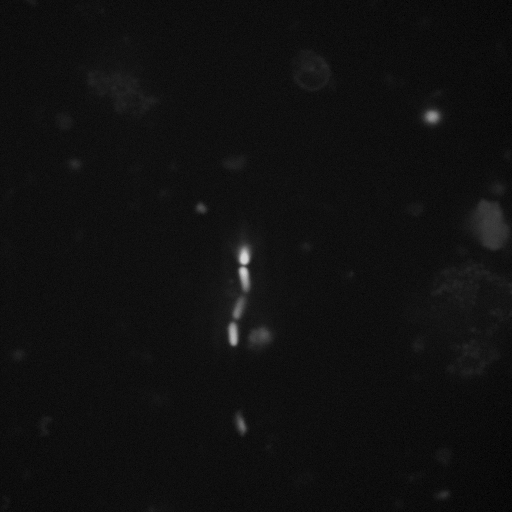

Supplement: S1 File — (ZIP) [file pcbi.1006986.s002.zip › extrait4h/4h_Z128_22_w2sdcGFP.tif]

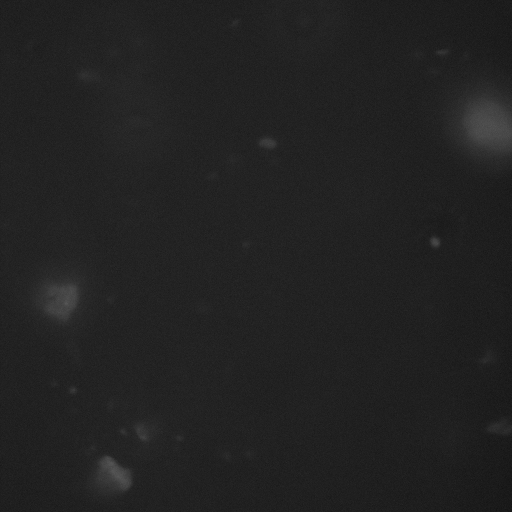

Supplement: S1 File — (ZIP) [file pcbi.1006986.s002.zip › extrait4h/4h_Z125_16_w2sdcGFP.tif]

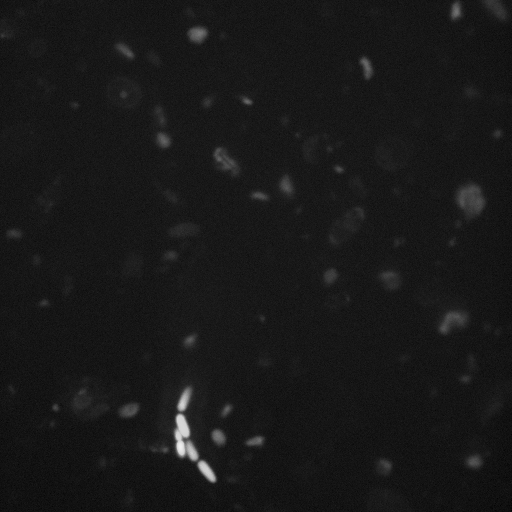

Supplement: S1 File — (ZIP) [file pcbi.1006986.s002.zip › extrait4h/4h_Z125_27_w1sdcRFP.tif]

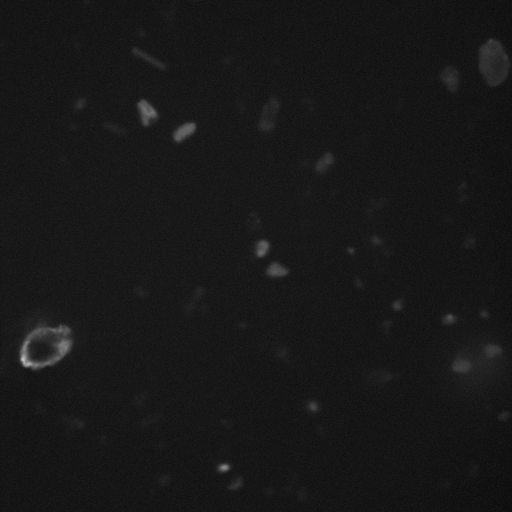

Supplement: S1 File — (ZIP) [file pcbi.1006986.s002.zip › extrait4h/4h_Z128_8_w2sdcGFP.tif]

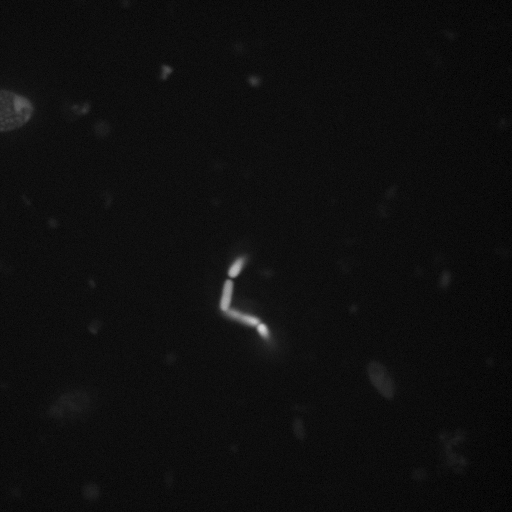

Supplement: S1 File — (ZIP) [file pcbi.1006986.s002.zip › extrait4h/4h_Z128_24_w2sdcGFP.tif]

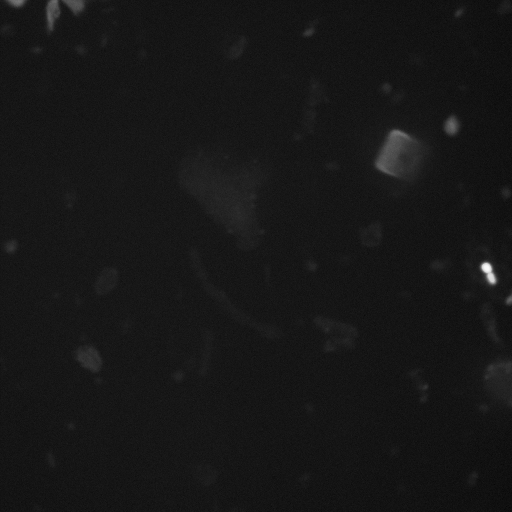

Supplement: S1 File — (ZIP) [file pcbi.1006986.s002.zip › extrait4h/4h_Z128_21_w2sdcGFP.tif]

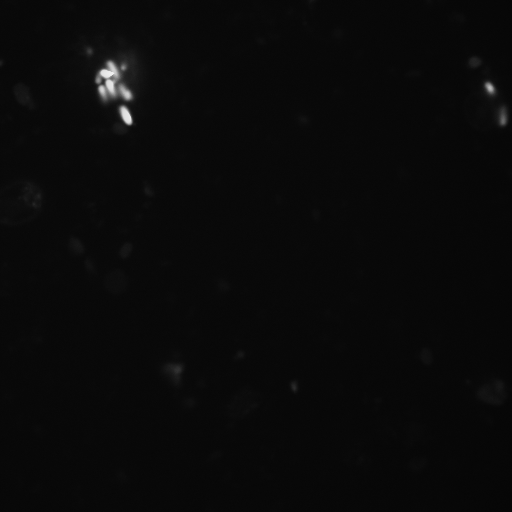

Supplement: S1 File — (ZIP) [file pcbi.1006986.s002.zip › extrait4h/4h_Z129_20_w2sdcGFP.tif]

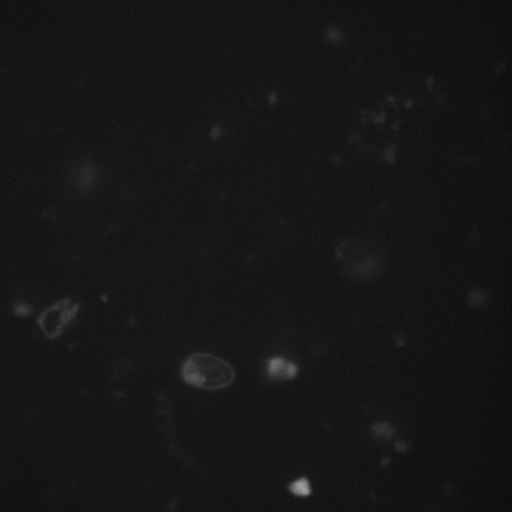

Supplement: S1 File — (ZIP) [file pcbi.1006986.s002.zip › extrait4h/4h_Z128_28_w2sdcGFP.tif]

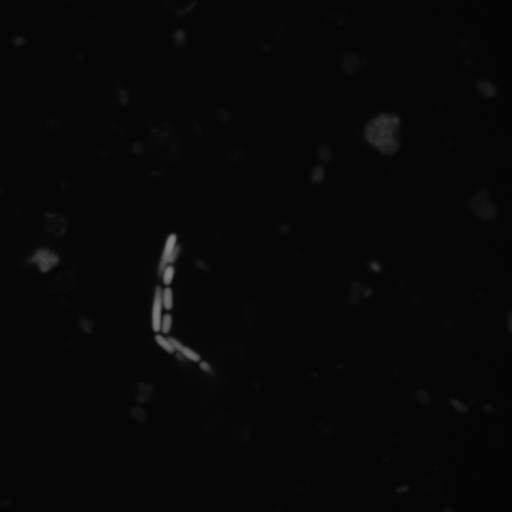

Supplement: S1 File — (ZIP) [file pcbi.1006986.s002.zip › extrait4h/4h_Z125_21_w2sdcGFP.tif]

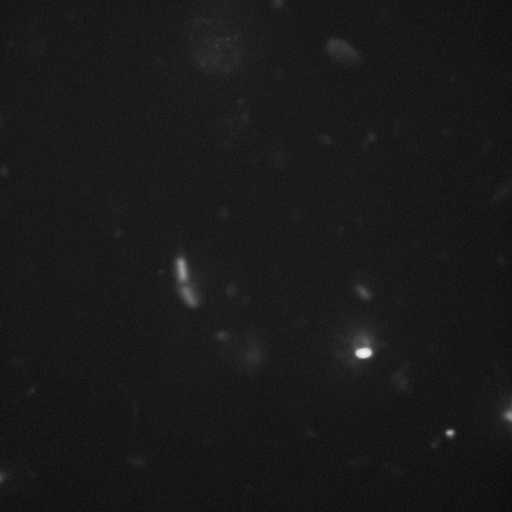

Supplement: S1 File — (ZIP) [file pcbi.1006986.s002.zip › extrait4h/4h_Z129_28_w1sdcRFP.tif]

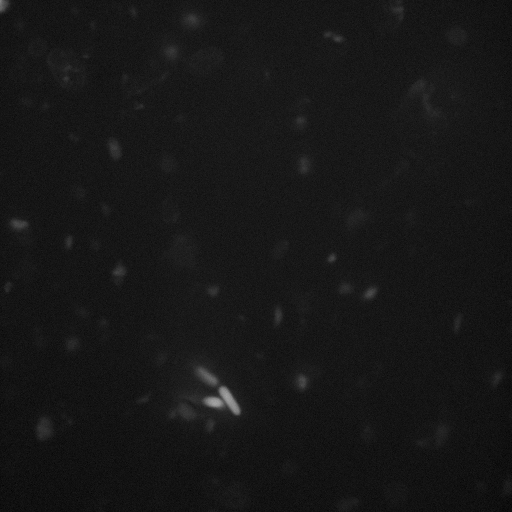

Supplement: S1 File — (ZIP) [file pcbi.1006986.s002.zip › extrait4h/4h_Z125_29_w1sdcRFP.tif]

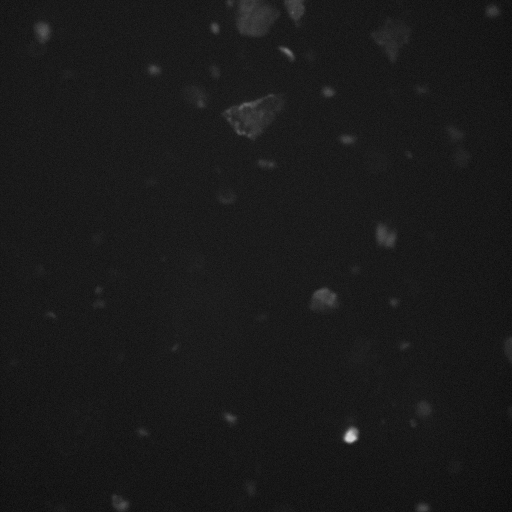

Supplement: S1 File — (ZIP) [file pcbi.1006986.s002.zip › extrait4h/4h_Z125_2_w1sdcRFP.tif]

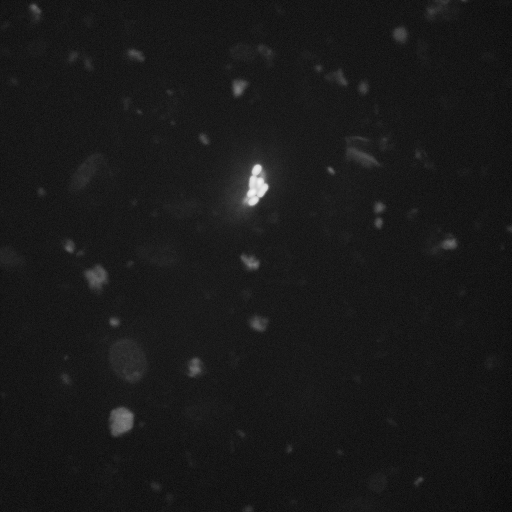

Supplement: S1 File — (ZIP) [file pcbi.1006986.s002.zip › extrait4h/4h_Z125_7_w1sdcRFP.tif]

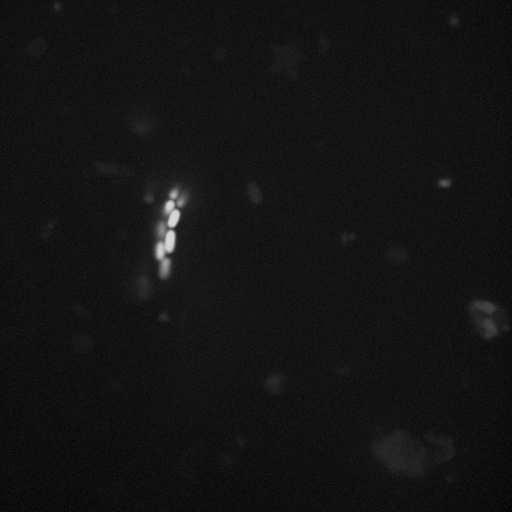

Supplement: S1 File — (ZIP) [file pcbi.1006986.s002.zip › extrait4h/4h_Z128_39_w1sdcRFP.tif]

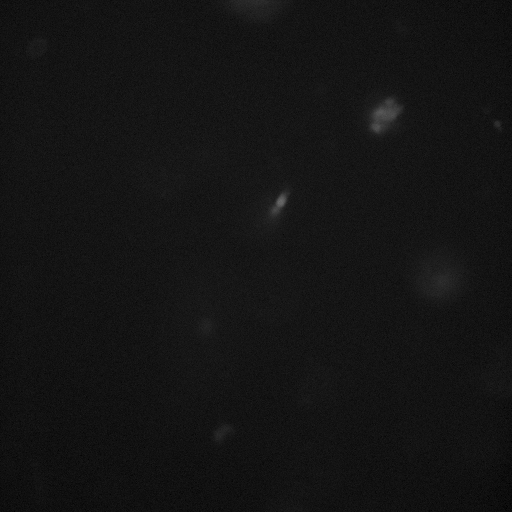

Supplement: S1 File — (ZIP) [file pcbi.1006986.s002.zip › extrait4h/4h_Z128_5_w1sdcRFP.tif]

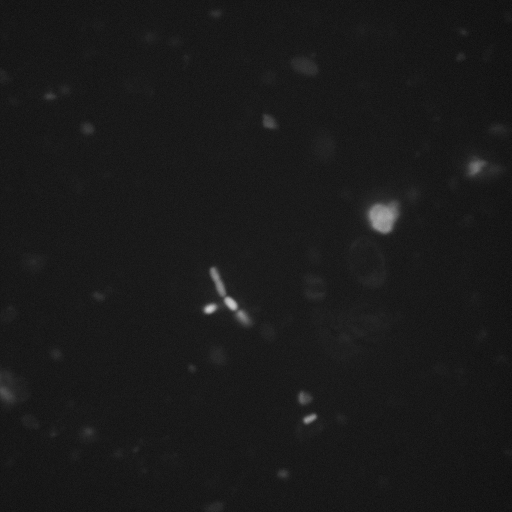

Supplement: S1 File — (ZIP) [file pcbi.1006986.s002.zip › extrait4h/4h_Z129_39_w2sdcGFP.tif]

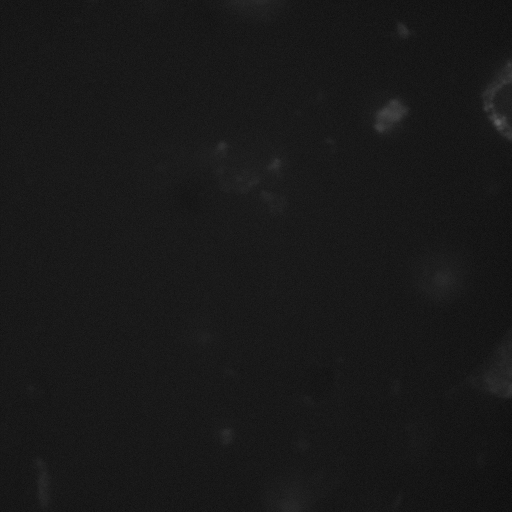

Supplement: S1 File — (ZIP) [file pcbi.1006986.s002.zip › extrait4h/4h_Z128_5_w2sdcGFP.tif]

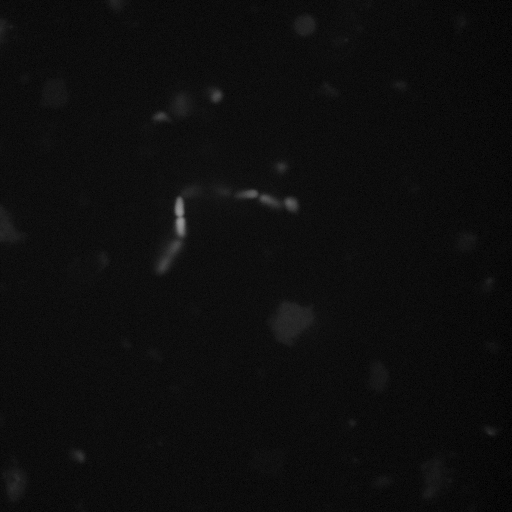

Supplement: S1 File — (ZIP) [file pcbi.1006986.s002.zip › extrait4h/4h_Z128_40_w2sdcGFP.tif]

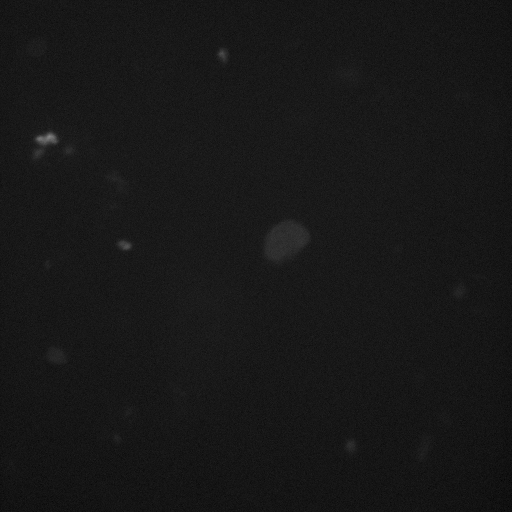

Supplement: S1 File — (ZIP) [file pcbi.1006986.s002.zip › extrait4h/4h_Z128_11_w1sdcRFP.tif]

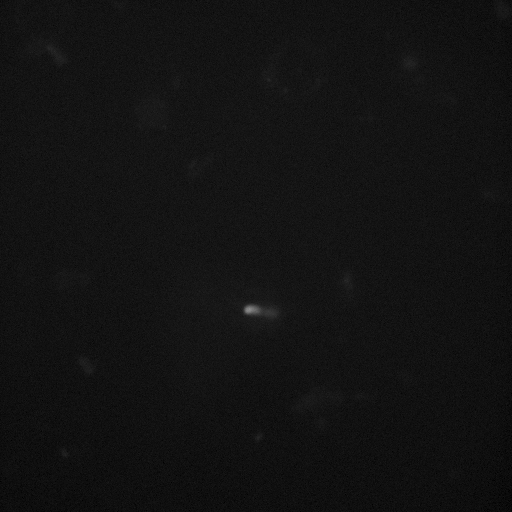

Supplement: S1 File — (ZIP) [file pcbi.1006986.s002.zip › extrait4h/4h_Z128_1_w1sdcRFP.tif]

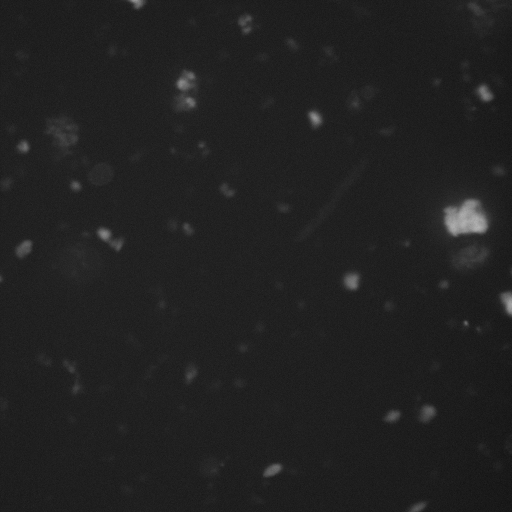

Supplement: S1 File — (ZIP) [file pcbi.1006986.s002.zip › extrait4h/4h_Z125_30_w2sdcGFP.tif]

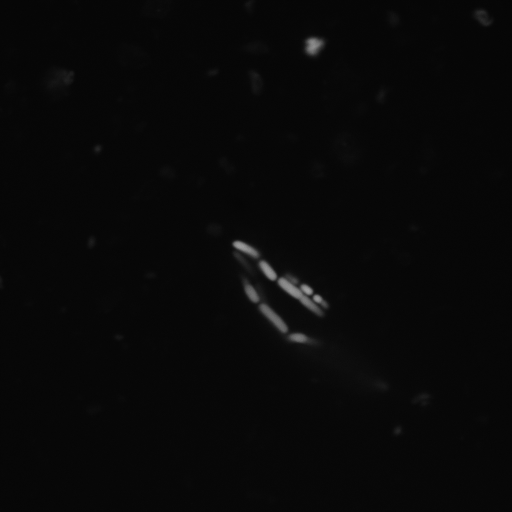

Supplement: S1 File — (ZIP) [file pcbi.1006986.s002.zip › extrait4h/4h_Z125_26_w2sdcGFP.tif]

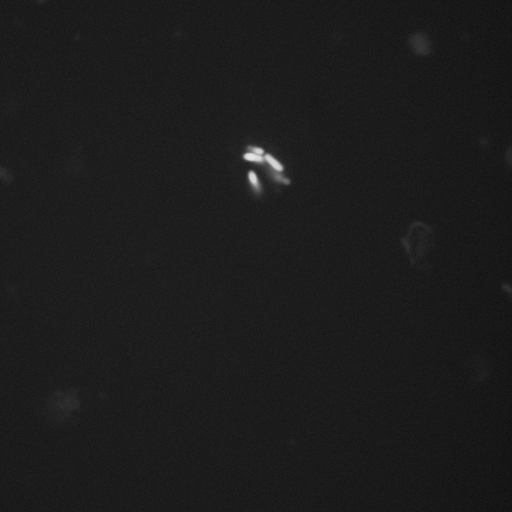

Supplement: S1 File — (ZIP) [file pcbi.1006986.s002.zip › extrait4h/4h_Z129_1_w2sdcGFP.tif]

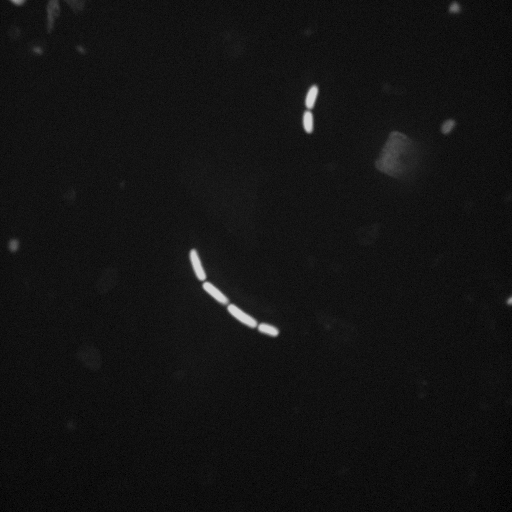

Supplement: S1 File — (ZIP) [file pcbi.1006986.s002.zip › extrait4h/4h_Z128_21_w1sdcRFP.tif]

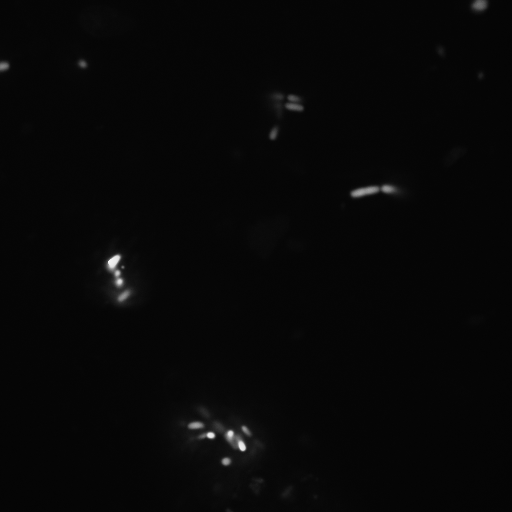

Supplement: S1 File — (ZIP) [file pcbi.1006986.s002.zip › extrait4h/4h_Z129_21_w1sdcRFP.tif]

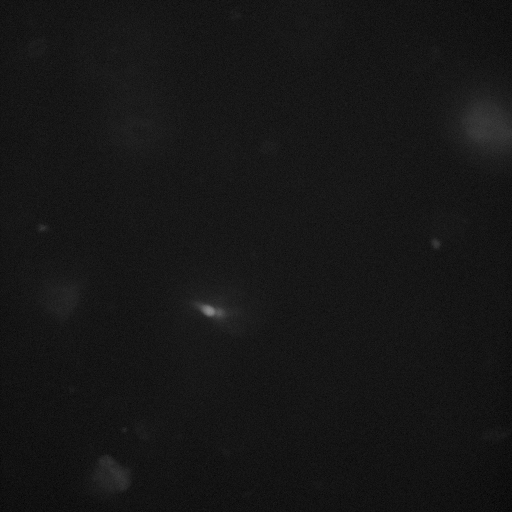

Supplement: S1 File — (ZIP) [file pcbi.1006986.s002.zip › extrait4h/4h_Z125_16_w1sdcRFP.tif]

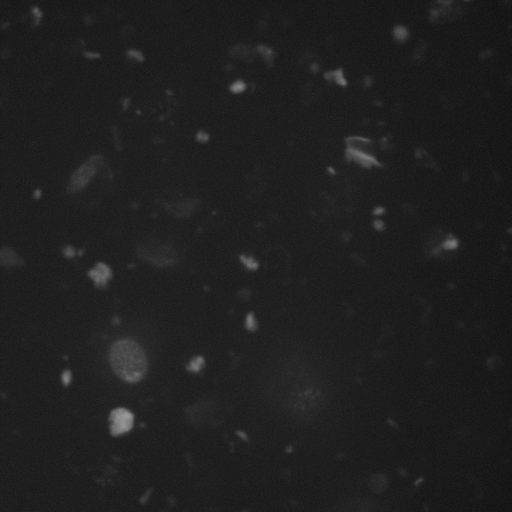

Supplement: S1 File — (ZIP) [file pcbi.1006986.s002.zip › extrait4h/4h_Z125_7_w2sdcGFP.tif]

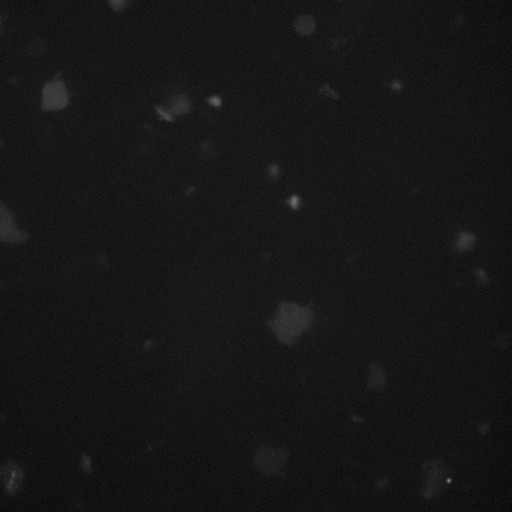

Supplement: S1 File — (ZIP) [file pcbi.1006986.s002.zip › extrait4h/4h_Z128_40_w1sdcRFP.tif]

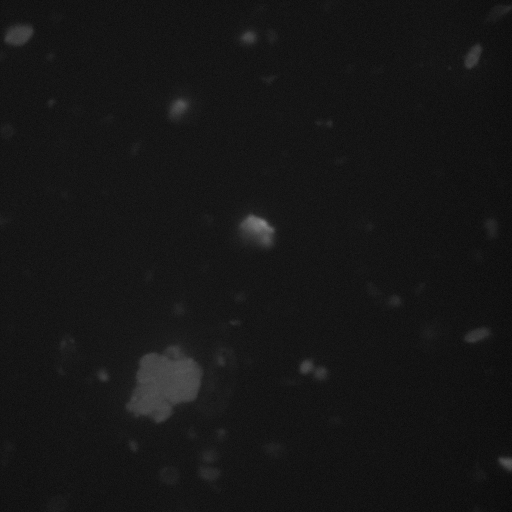

Supplement: S1 File — (ZIP) [file pcbi.1006986.s002.zip › extrait4h/4h_Z128_42_w2sdcGFP.tif]

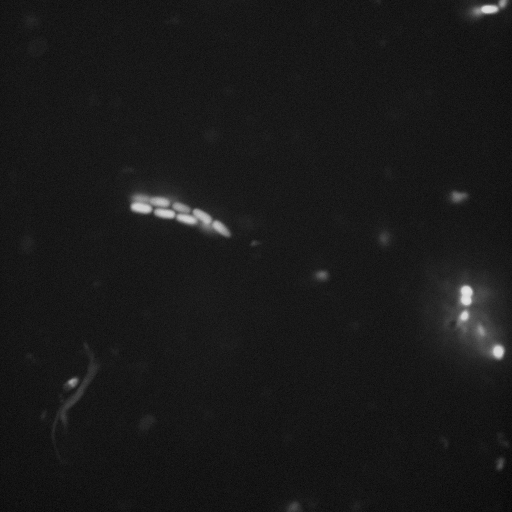

Supplement: S1 File — (ZIP) [file pcbi.1006986.s002.zip › extrait4h/4h_Z129_43_w1sdcRFP.tif]

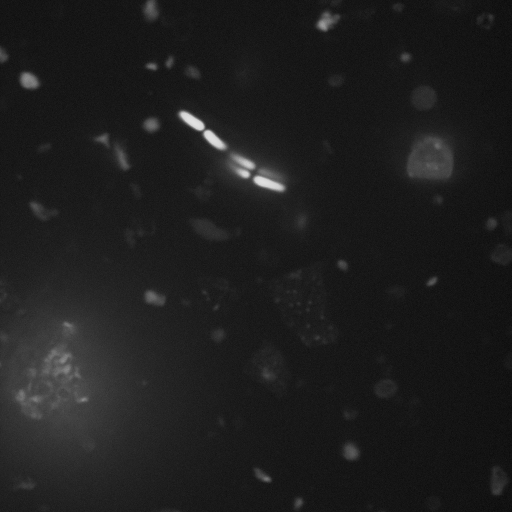

Supplement: S1 File — (ZIP) [file pcbi.1006986.s002.zip › extrait4h/4h_Z125_20_w2sdcGFP.tif]

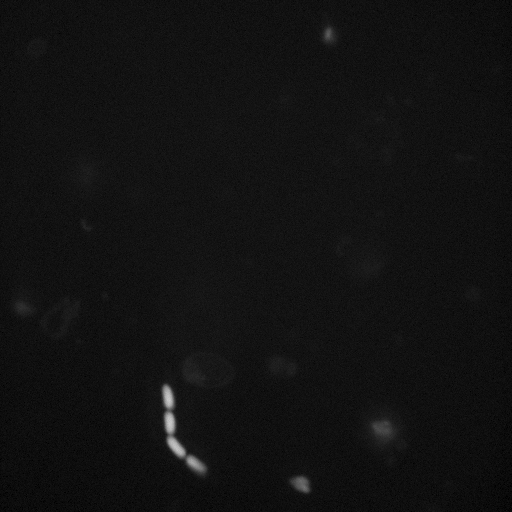

Supplement: S1 File — (ZIP) [file pcbi.1006986.s002.zip › extrait4h/4h_Z128_28_w1sdcRFP.tif]

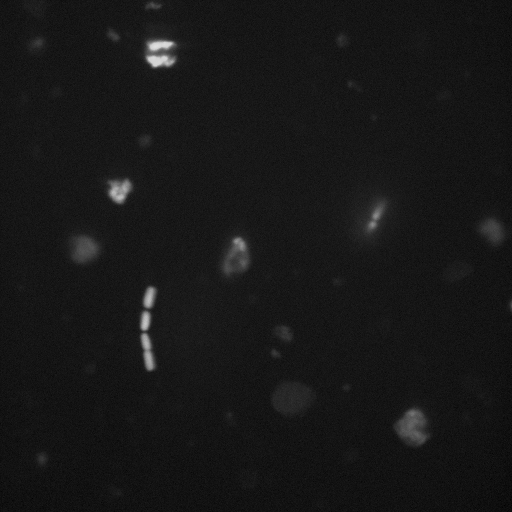

Supplement: S1 File — (ZIP) [file pcbi.1006986.s002.zip › extrait4h/4h_Z129_25_w1sdcRFP.tif]

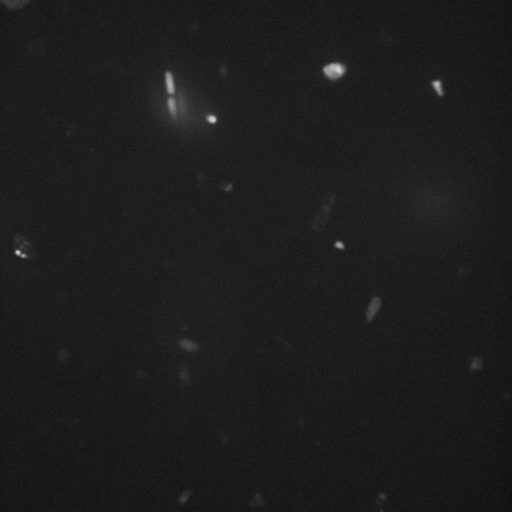

Supplement: S1 File — (ZIP) [file pcbi.1006986.s002.zip › extrait4h/4h_Z129_10_w1sdcRFP.tif]

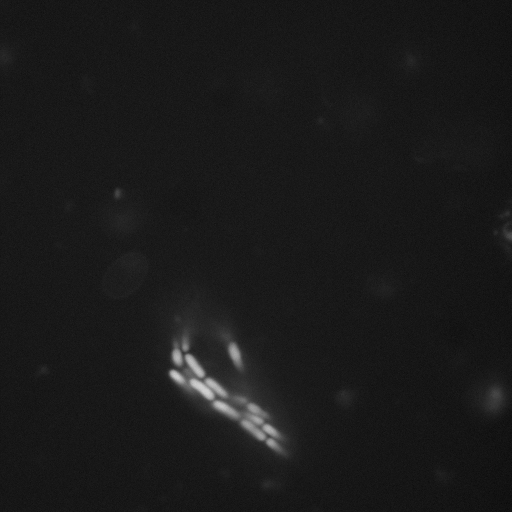

Supplement: S1 File — (ZIP) [file pcbi.1006986.s002.zip › extrait4h/4h_Z125_3_w2sdcGFP.tif]

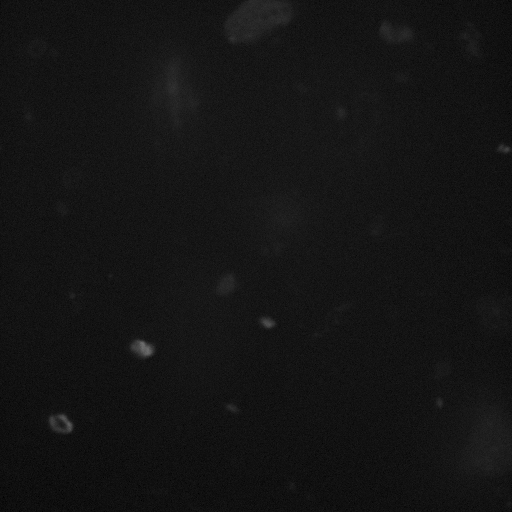

Supplement: S1 File — (ZIP) [file pcbi.1006986.s002.zip › extrait4h/4h_Z128_12_w1sdcRFP.tif]

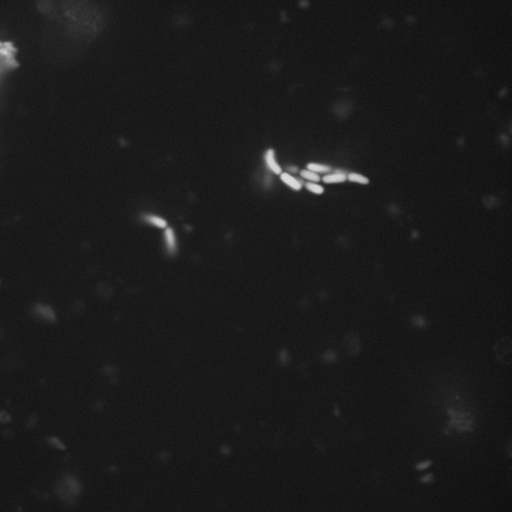

Supplement: S1 File — (ZIP) [file pcbi.1006986.s002.zip › extrait4h/4h_Z125_6_w2sdcGFP.tif]

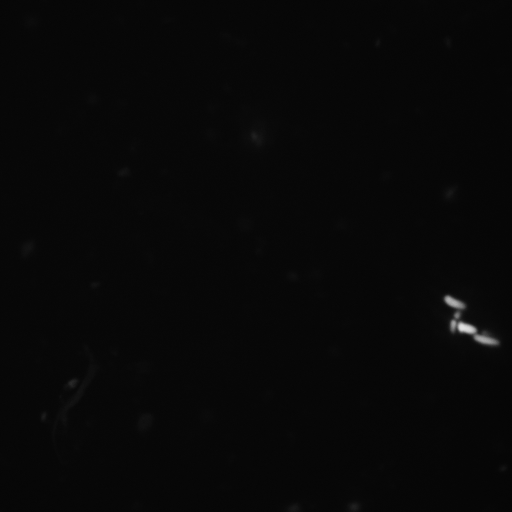

Supplement: S1 File — (ZIP) [file pcbi.1006986.s002.zip › extrait4h/4h_Z129_43_w2sdcGFP.tif]

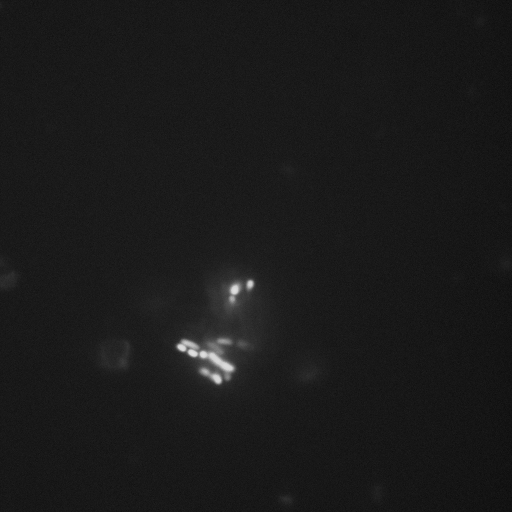

Supplement: S1 File — (ZIP) [file pcbi.1006986.s002.zip › extrait4h/4h_Z129_4_w2sdcGFP.tif]

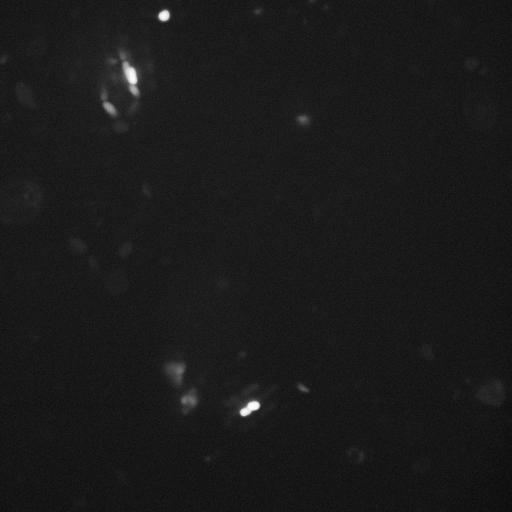

Supplement: S1 File — (ZIP) [file pcbi.1006986.s002.zip › extrait4h/4h_Z129_20_w1sdcRFP.tif]

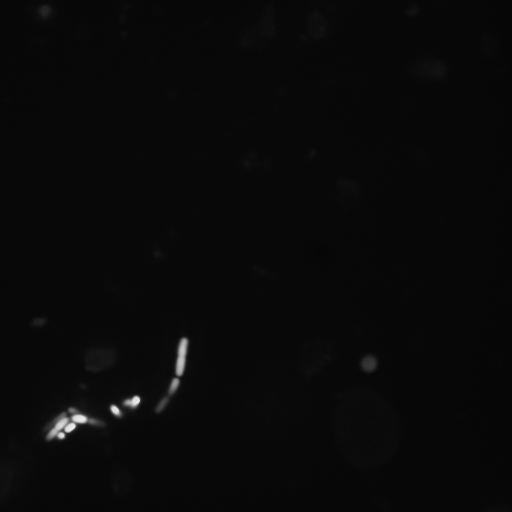

Supplement: S1 File — (ZIP) [file pcbi.1006986.s002.zip › extrait4h/4h_Z129_32_w2sdcGFP.tif]

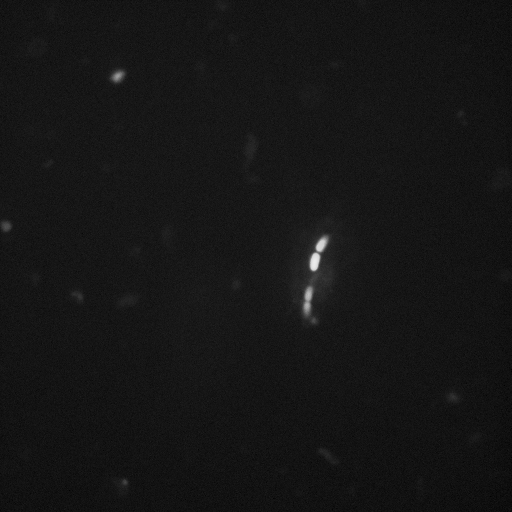

Supplement: S1 File — (ZIP) [file pcbi.1006986.s002.zip › extrait4h/4h_Z129_23_w1sdcRFP.tif]

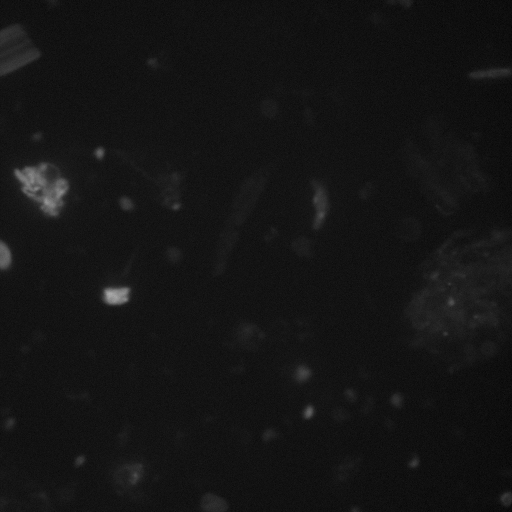

Supplement: S1 File — (ZIP) [file pcbi.1006986.s002.zip › extrait4h/4h_Z128_45_w2sdcGFP.tif]

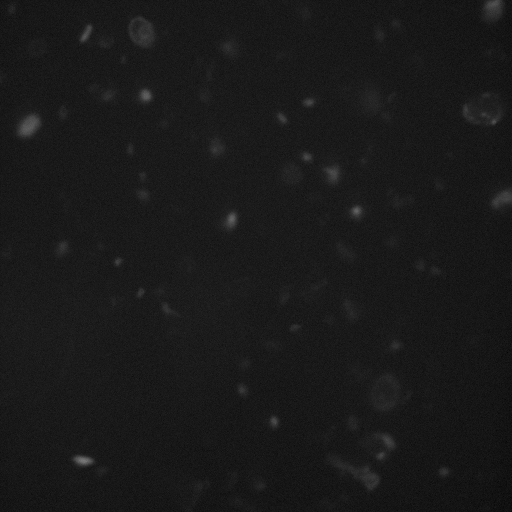

Supplement: S1 File — (ZIP) [file pcbi.1006986.s002.zip › extrait4h/4h_Z125_19_w1sdcRFP.tif]

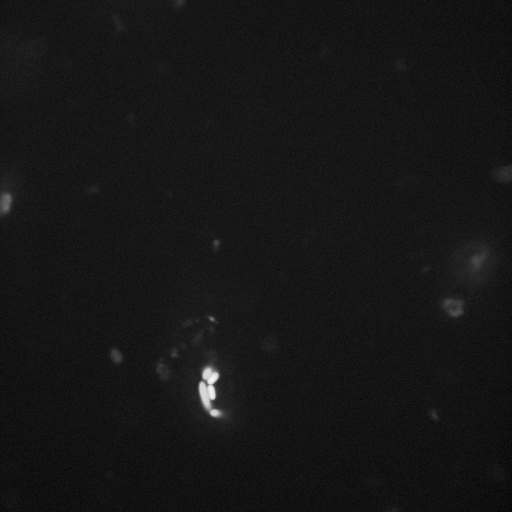

Supplement: S1 File — (ZIP) [file pcbi.1006986.s002.zip › extrait4h/4h_Z129_26_w1sdcRFP.tif]

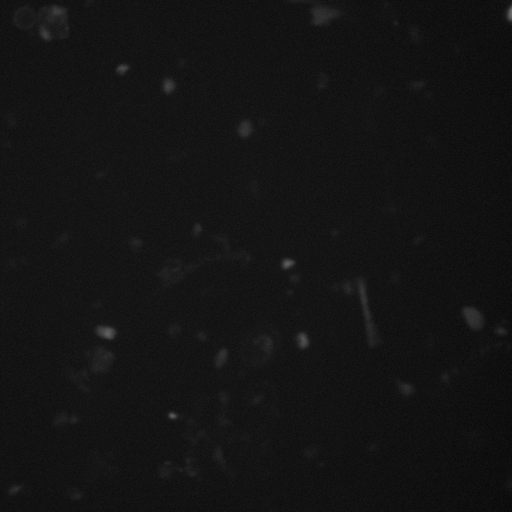

Supplement: S1 File — (ZIP) [file pcbi.1006986.s002.zip › extrait4h/4h_Z128_37_w2sdcGFP.tif]

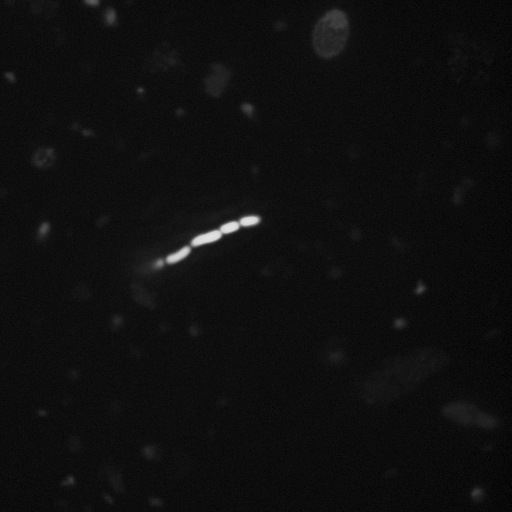

Supplement: S1 File — (ZIP) [file pcbi.1006986.s002.zip › extrait4h/4h_Z128_35_w2sdcGFP.tif]

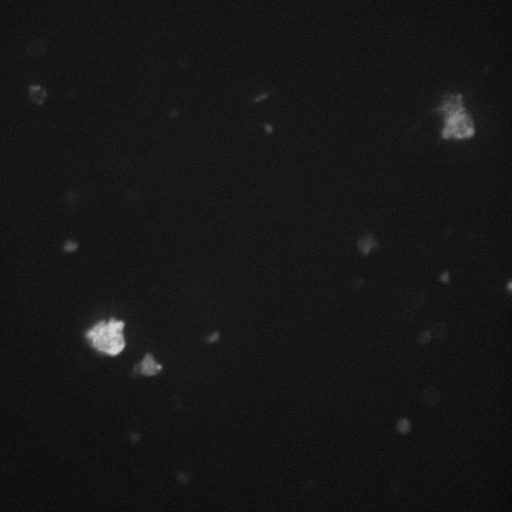

Supplement: S1 File — (ZIP) [file pcbi.1006986.s002.zip › extrait4h/4h_Z128_4_w1sdcRFP.tif]

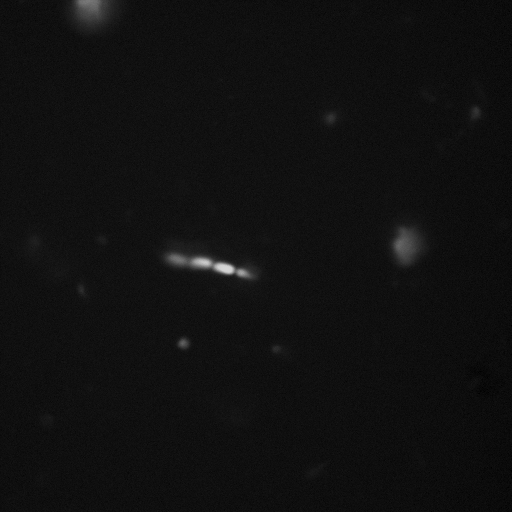

Supplement: S1 File — (ZIP) [file pcbi.1006986.s002.zip › extrait4h/4h_Z128_14_w2sdcGFP.tif]

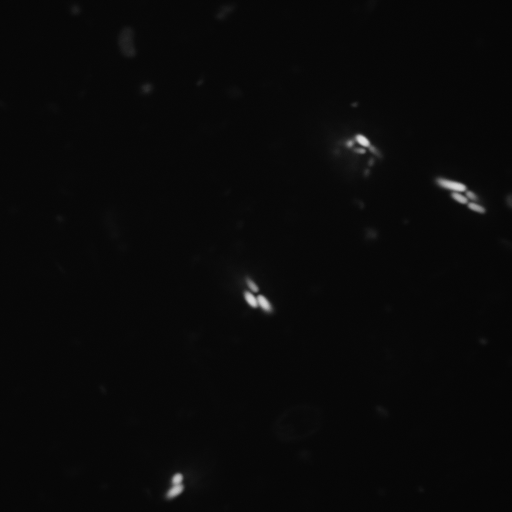

Supplement: S1 File — (ZIP) [file pcbi.1006986.s002.zip › extrait4h/4h_Z129_42_w2sdcGFP.tif]

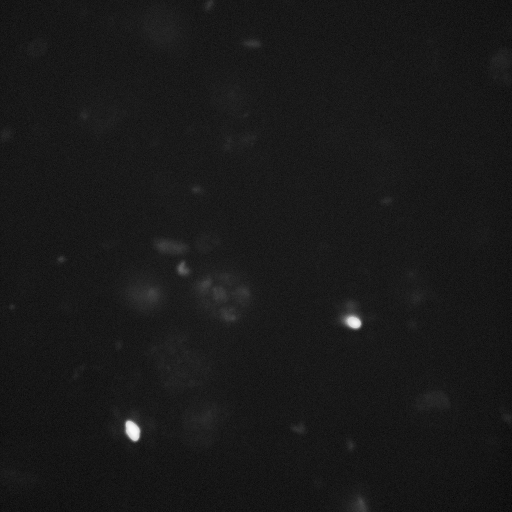

Supplement: S1 File — (ZIP) [file pcbi.1006986.s002.zip › extrait4h/4h_Z125_10_w1sdcRFP.tif]

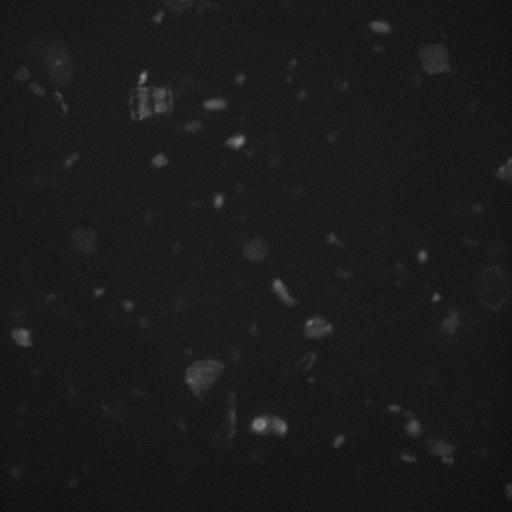

Supplement: S1 File — (ZIP) [file pcbi.1006986.s002.zip › extrait4h/4h_Z125_24_w1sdcRFP.tif]

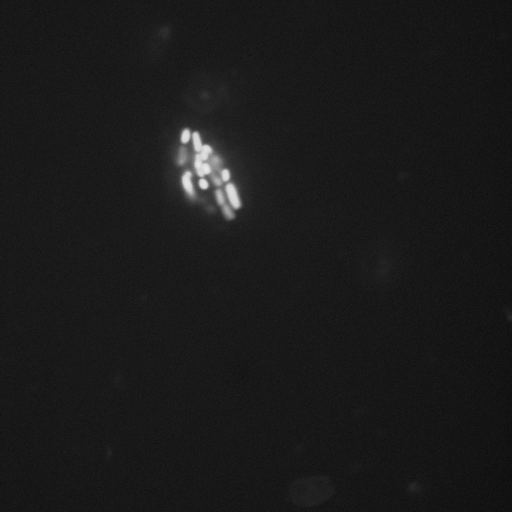

Supplement: S1 File — (ZIP) [file pcbi.1006986.s002.zip › extrait4h/4h_Z129_16_w2sdcGFP.tif]

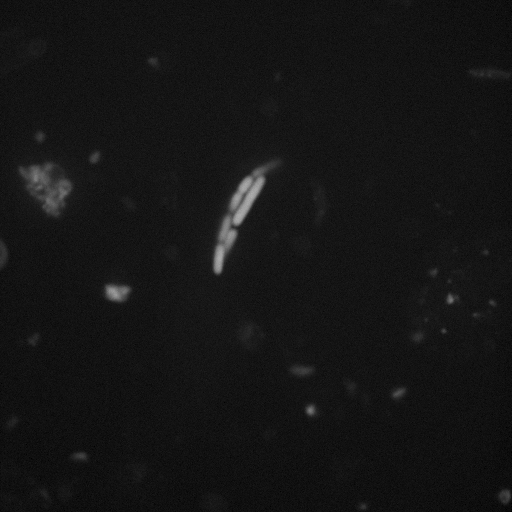

Supplement: S1 File — (ZIP) [file pcbi.1006986.s002.zip › extrait4h/4h_Z128_45_w1sdcRFP.tif]

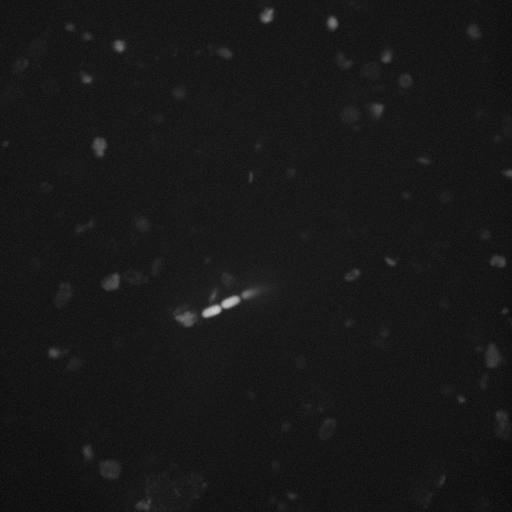

Supplement: S1 File — (ZIP) [file pcbi.1006986.s002.zip › extrait4h/4h_Z125_23_w1sdcRFP.tif]

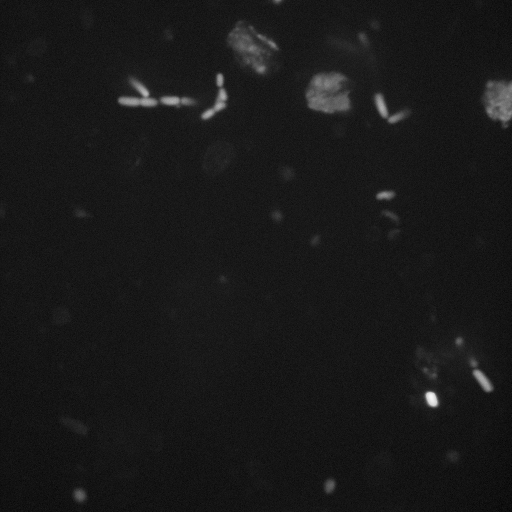

Supplement: S1 File — (ZIP) [file pcbi.1006986.s002.zip › extrait4h/4h_Z129_41_w1sdcRFP.tif]

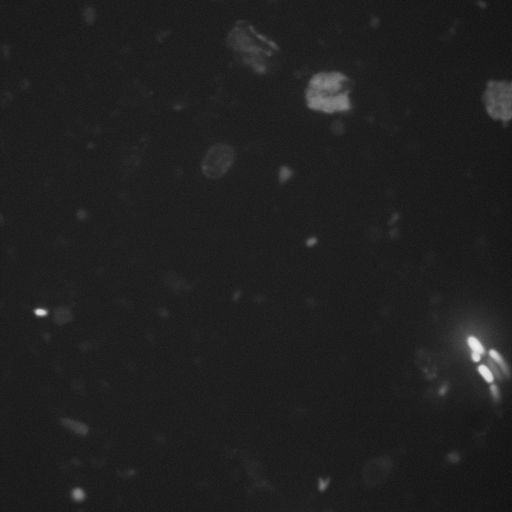

Supplement: S1 File — (ZIP) [file pcbi.1006986.s002.zip › extrait4h/4h_Z129_41_w2sdcGFP.tif]

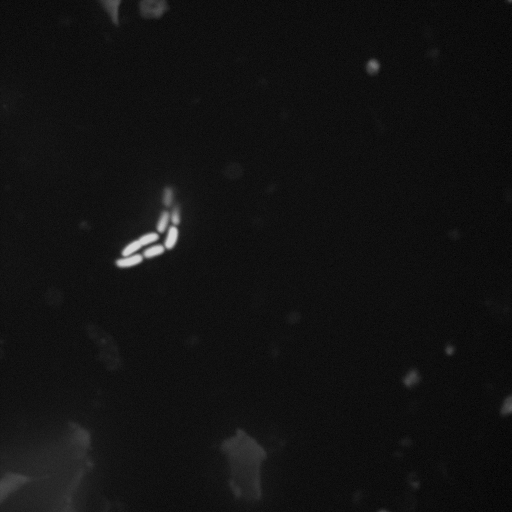

Supplement: S1 File — (ZIP) [file pcbi.1006986.s002.zip › extrait4h/4h_Z128_15_w2sdcGFP.tif]

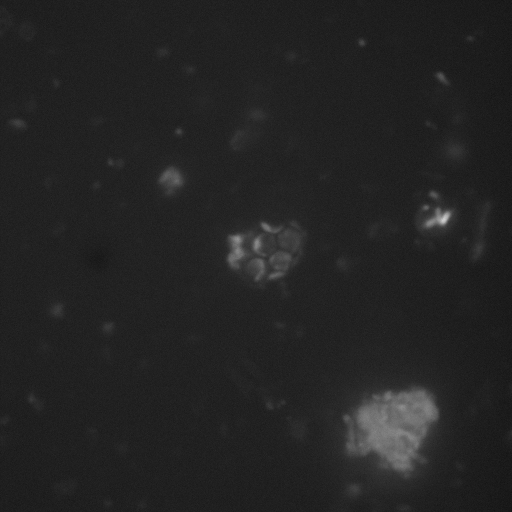

Supplement: S1 File — (ZIP) [file pcbi.1006986.s002.zip › extrait4h/4h_Z125_15_w2sdcGFP.tif]

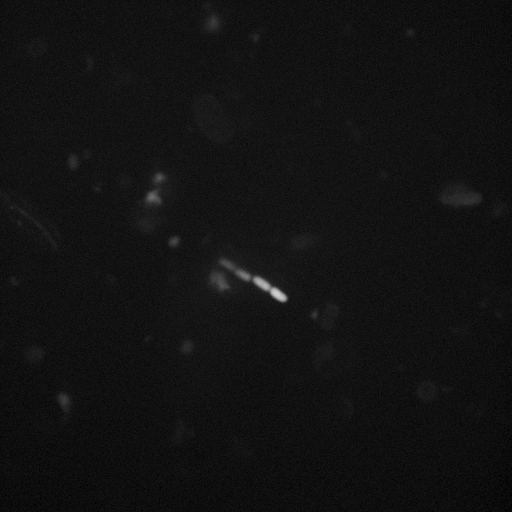

Supplement: S1 File — (ZIP) [file pcbi.1006986.s002.zip › extrait4h/4h_Z128_18_w1sdcRFP.tif]

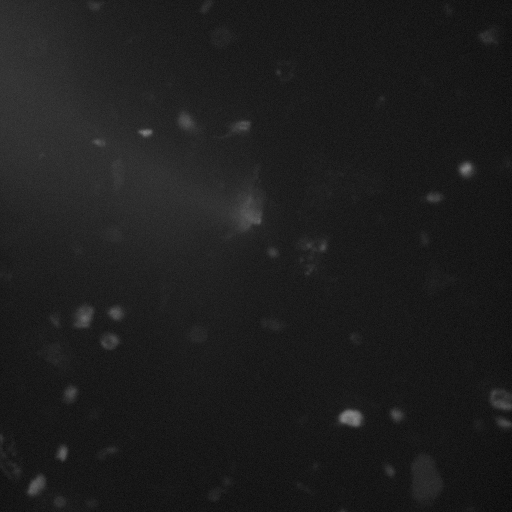

Supplement: S1 File — (ZIP) [file pcbi.1006986.s002.zip › extrait4h/4h_Z125_13_w1sdcRFP.tif]

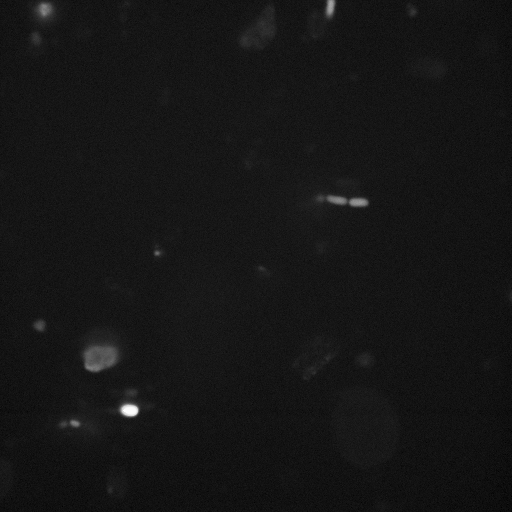

Supplement: S1 File — (ZIP) [file pcbi.1006986.s002.zip › extrait4h/4h_Z129_32_w1sdcRFP.tif]

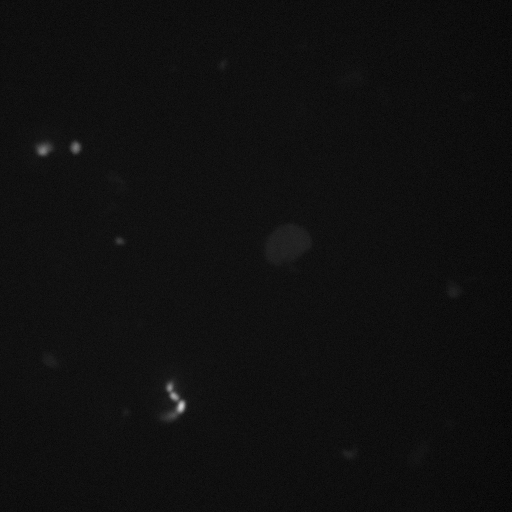

Supplement: S1 File — (ZIP) [file pcbi.1006986.s002.zip › extrait4h/4h_Z128_11_w2sdcGFP.tif]

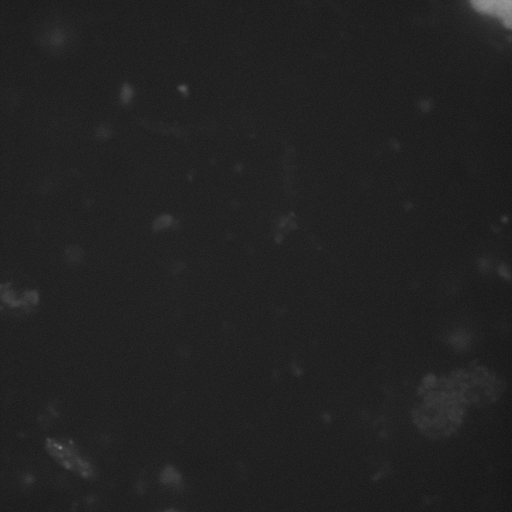

Supplement: S1 File — (ZIP) [file pcbi.1006986.s002.zip › extrait4h/4h_Z125_18_w2sdcGFP.tif]

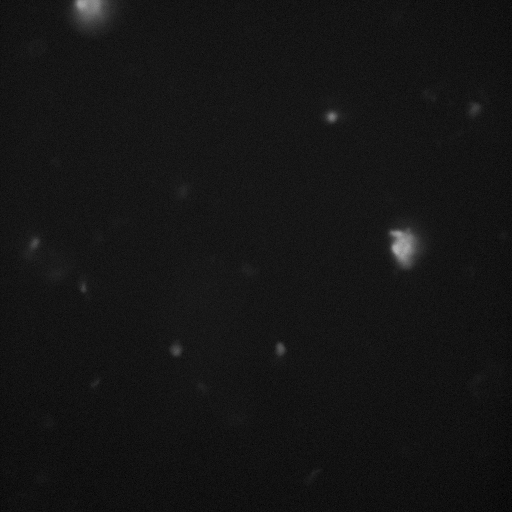

Supplement: S1 File — (ZIP) [file pcbi.1006986.s002.zip › extrait4h/4h_Z128_14_w1sdcRFP.tif]

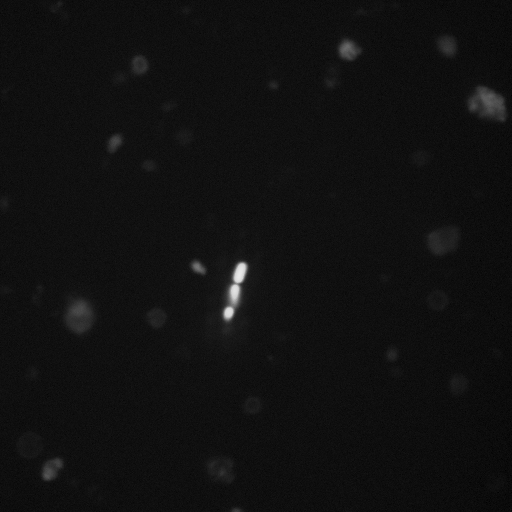

Supplement: S1 File — (ZIP) [file pcbi.1006986.s002.zip › extrait4h/4h_Z128_13_w2sdcGFP.tif]

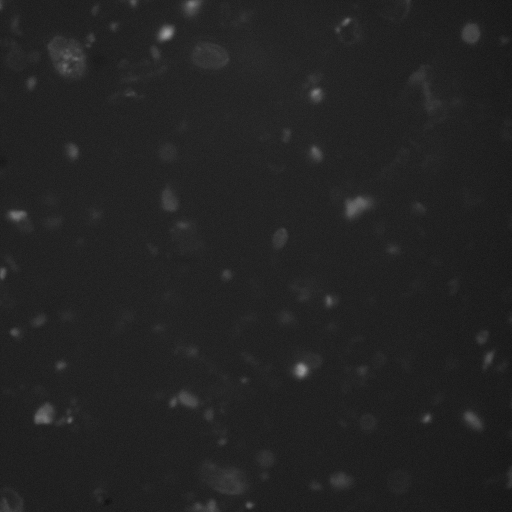

Supplement: S1 File — (ZIP) [file pcbi.1006986.s002.zip › extrait4h/4h_Z125_28_w2sdcGFP.tif]

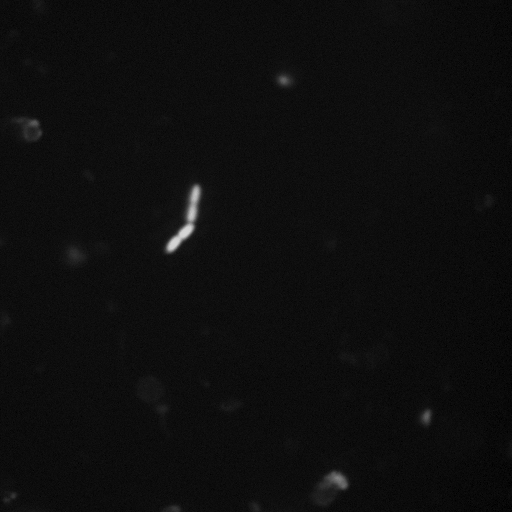

Supplement: S1 File — (ZIP) [file pcbi.1006986.s002.zip › extrait4h/4h_Z128_30_w2sdcGFP.tif]

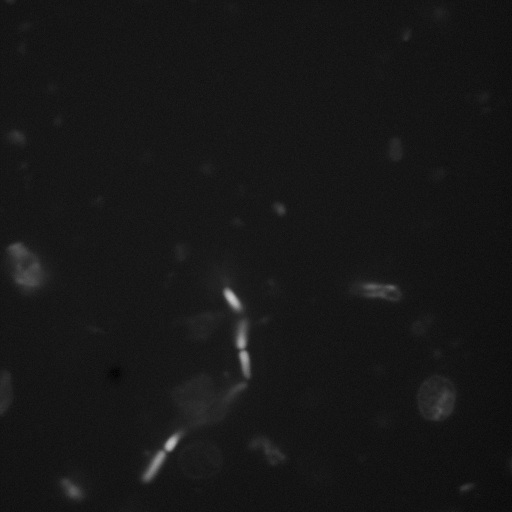

Supplement: S1 File — (ZIP) [file pcbi.1006986.s002.zip › extrait4h/4h_Z125_1_w2sdcGFP.tif]

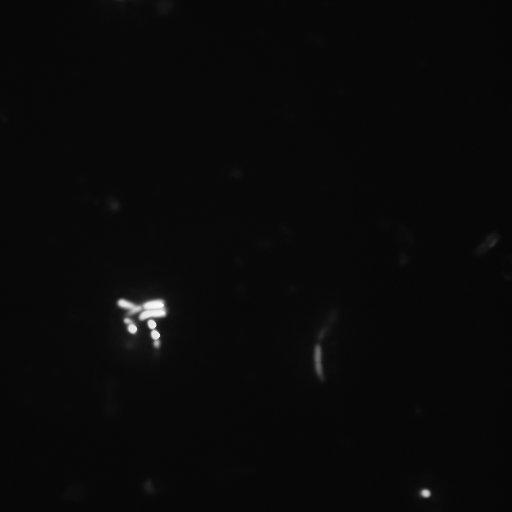

Supplement: S1 File — (ZIP) [file pcbi.1006986.s002.zip › extrait4h/4h_Z129_24_w2sdcGFP.tif]

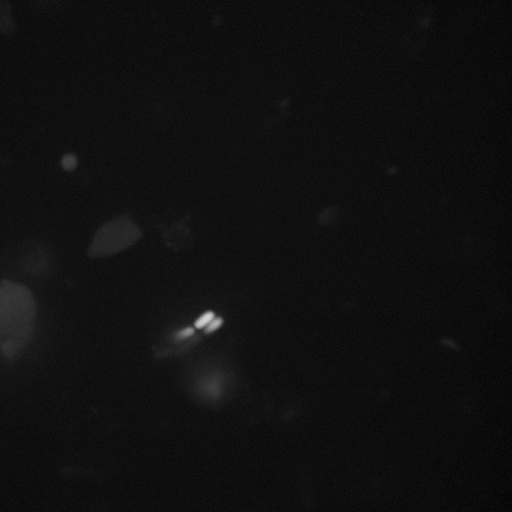

Supplement: S1 File — (ZIP) [file pcbi.1006986.s002.zip › extrait4h/4h_Z128_32_w2sdcGFP.tif]
