# Supplementary figures and images for: Enchained growth and cluster dislocation: A possible mechanism for microbiota homeostasis (part 4 of 10)
Source: PLoS Comput Biol. 2019 May 3;15(5):e1006986. doi: 10.1371/journal.pcbi.1006986 (PMC6519844; doi:10.1371/journal.pcbi.1006986)

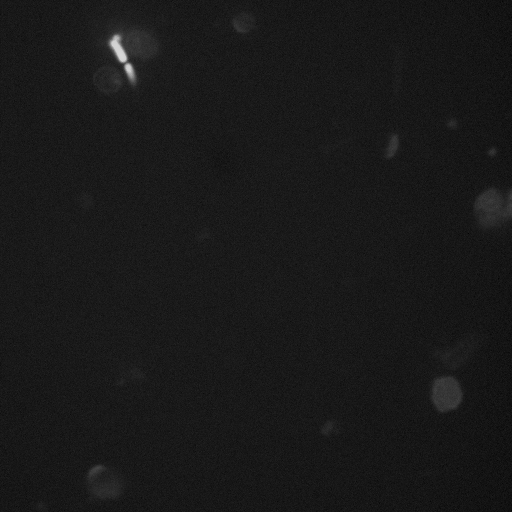

Supplement: S2 File — (ZIP) [file pcbi.1006986.s003.zip › extrait_4hKM16021/4h-Z694_2_w2sdcGFP.tif]

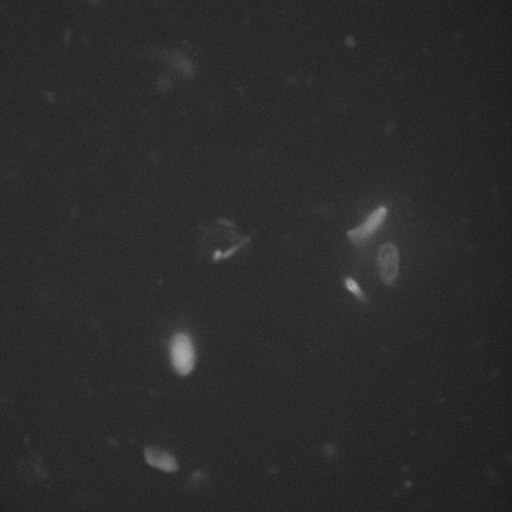

Supplement: S2 File — (ZIP) [file pcbi.1006986.s003.zip › extrait_4hKM16021/4h-Z692_16_w2sdcGFP.tif]

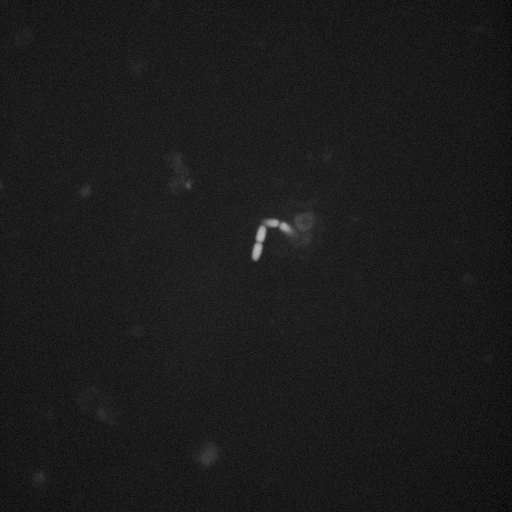

Supplement: S2 File — (ZIP) [file pcbi.1006986.s003.zip › extrait_4hKM16021/4h-Z693_2_w1sdcRFP.tif]

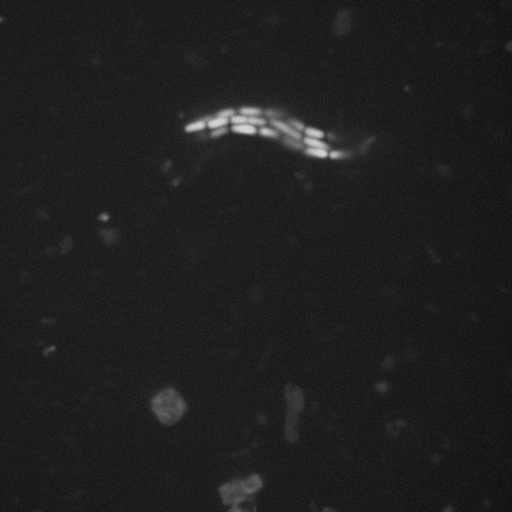

Supplement: S2 File — (ZIP) [file pcbi.1006986.s003.zip › extrait_4hKM16021/4h-Z692_21_w2sdcGFP.tif]

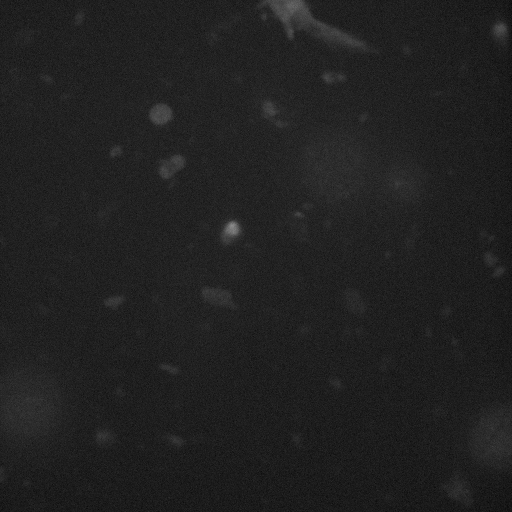

Supplement: S2 File — (ZIP) [file pcbi.1006986.s003.zip › extrait_4hKM16021/4h-Z693_7_w1sdcRFP.tif]

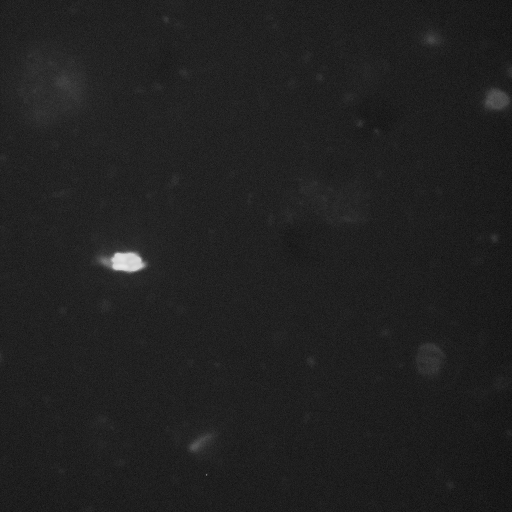

Supplement: S2 File — (ZIP) [file pcbi.1006986.s003.zip › extrait_4hKM16021/4h-Z693_17_w2sdcGFP.tif]

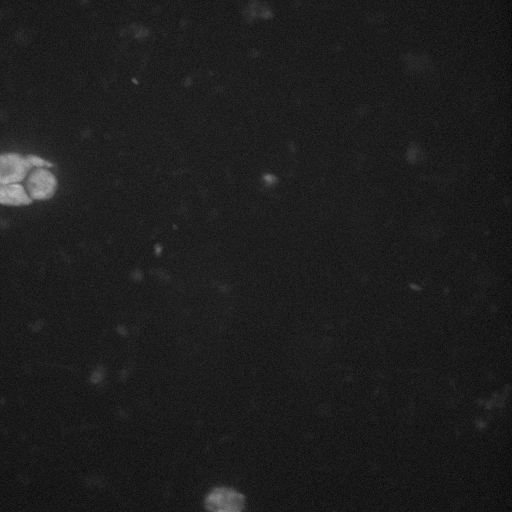

Supplement: S2 File — (ZIP) [file pcbi.1006986.s003.zip › extrait_4hKM16021/4h-Z692_34_w1sdcRFP.tif]

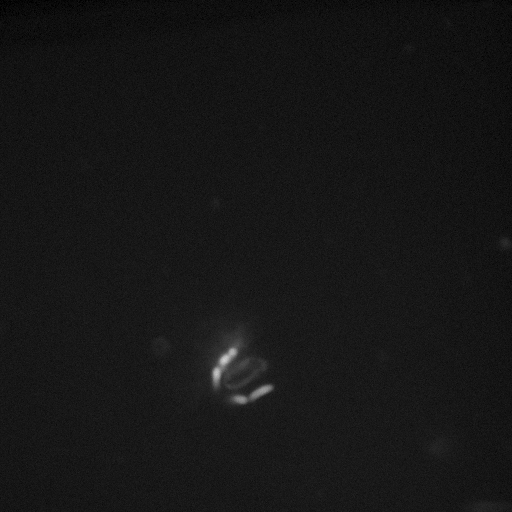

Supplement: S2 File — (ZIP) [file pcbi.1006986.s003.zip › extrait_4hKM16021/4h-Z693_3_w2sdcGFP.tif]

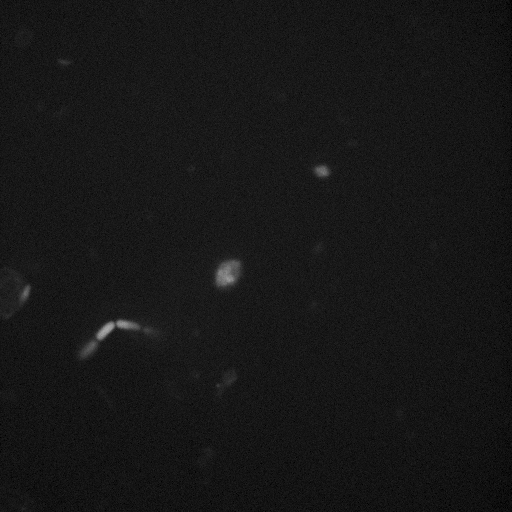

Supplement: S2 File — (ZIP) [file pcbi.1006986.s003.zip › extrait_4hKM16021/4h-Z694_12_w1sdcRFP.tif]

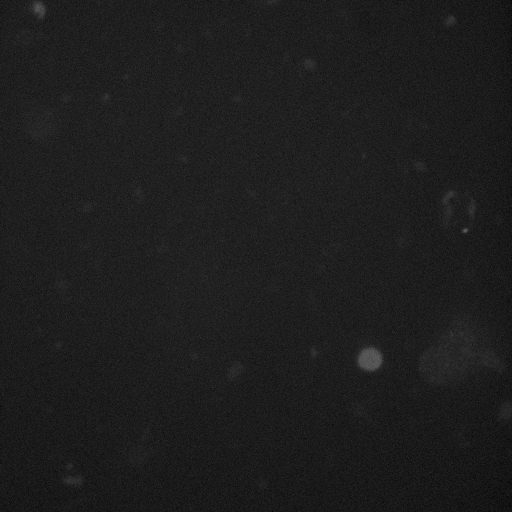

Supplement: S2 File — (ZIP) [file pcbi.1006986.s003.zip › extrait_4hKM16021/4h-Z692_8_w1sdcRFP.tif]

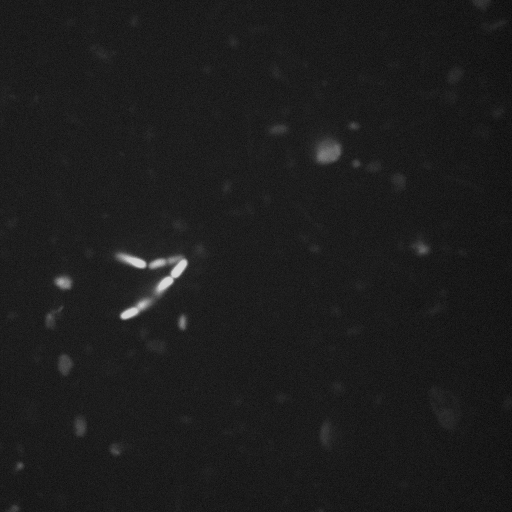

Supplement: S2 File — (ZIP) [file pcbi.1006986.s003.zip › extrait_4hKM16021/4h-Z692_20_w2sdcGFP.tif]

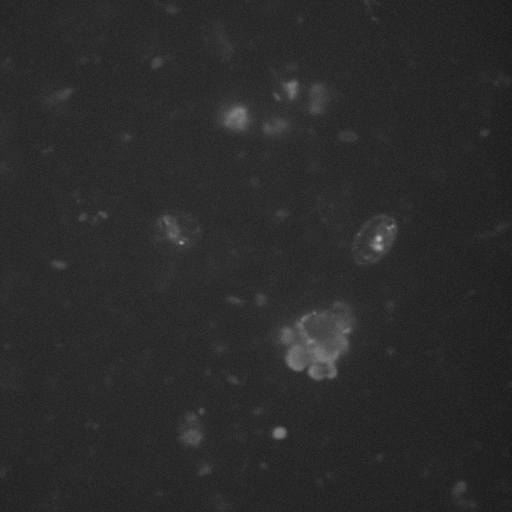

Supplement: S2 File — (ZIP) [file pcbi.1006986.s003.zip › extrait_4hKM16021/4h-Z693_26_w2sdcGFP.tif]

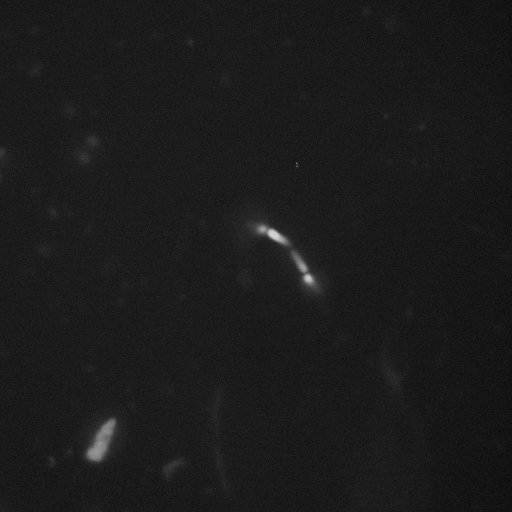

Supplement: S2 File — (ZIP) [file pcbi.1006986.s003.zip › extrait_4hKM16021/4h-Z692_19_w2sdcGFP.tif]

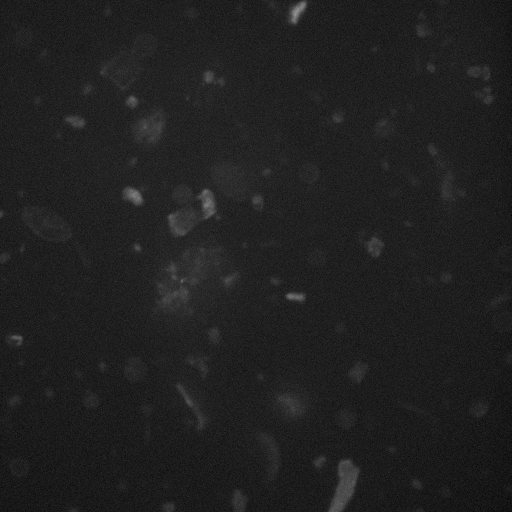

Supplement: S2 File — (ZIP) [file pcbi.1006986.s003.zip › extrait_4hKM16021/4h-Z695_2_w1sdcRFP.tif]

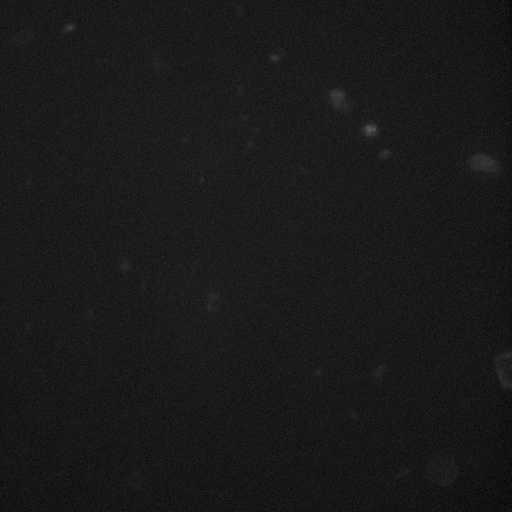

Supplement: S2 File — (ZIP) [file pcbi.1006986.s003.zip › extrait_4hKM16021/4h-Z694_9_w1sdcRFP.tif]

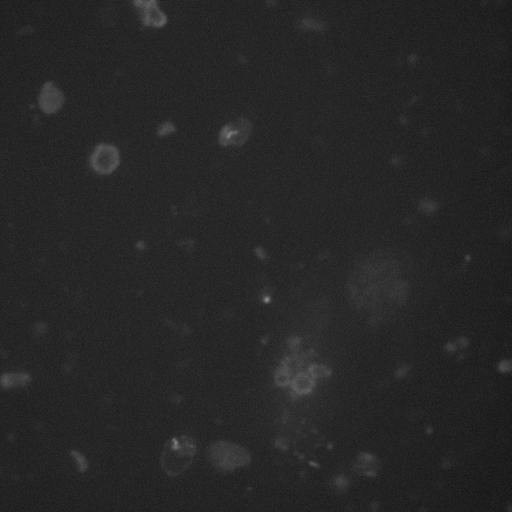

Supplement: S2 File — (ZIP) [file pcbi.1006986.s003.zip › extrait_4hKM16021/4h-Z693_5_w2sdcGFP.tif]

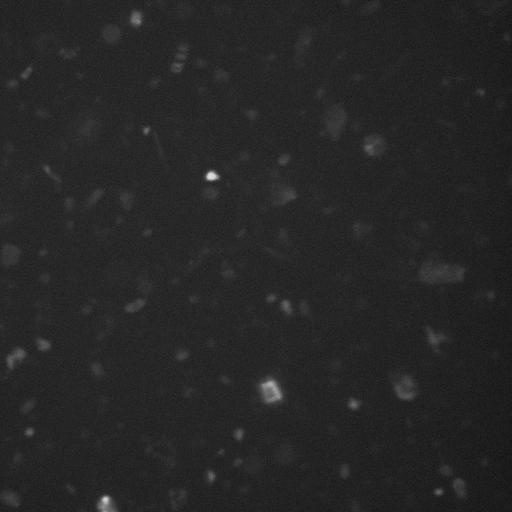

Supplement: S2 File — (ZIP) [file pcbi.1006986.s003.zip › extrait_4hKM16021/4h-Z693_29_w2sdcGFP.tif]

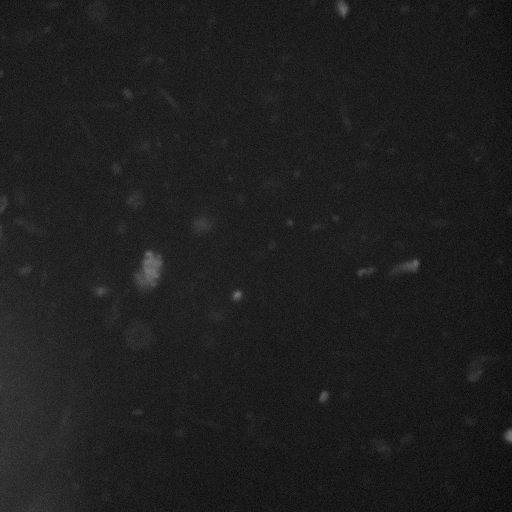

Supplement: S2 File — (ZIP) [file pcbi.1006986.s003.zip › extrait_4hKM16021/4h-Z692_35_w1sdcRFP.tif]

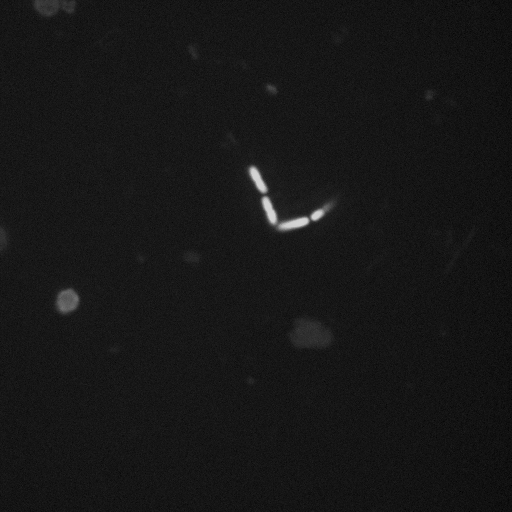

Supplement: S2 File — (ZIP) [file pcbi.1006986.s003.zip › extrait_4hKM16021/4h-Z694_11_w2sdcGFP.tif]

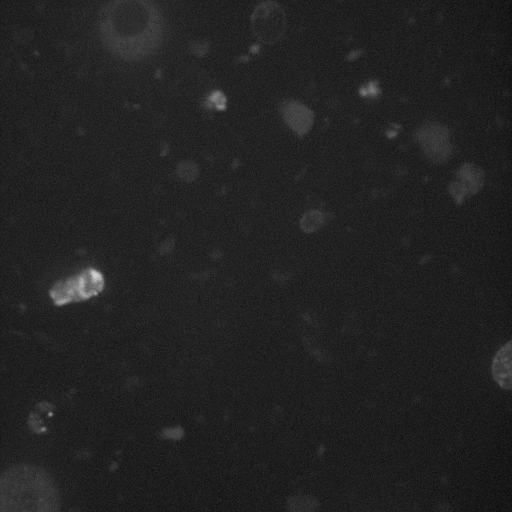

Supplement: S2 File — (ZIP) [file pcbi.1006986.s003.zip › extrait_4hKM16021/4h-Z693_20_w1sdcRFP.tif]

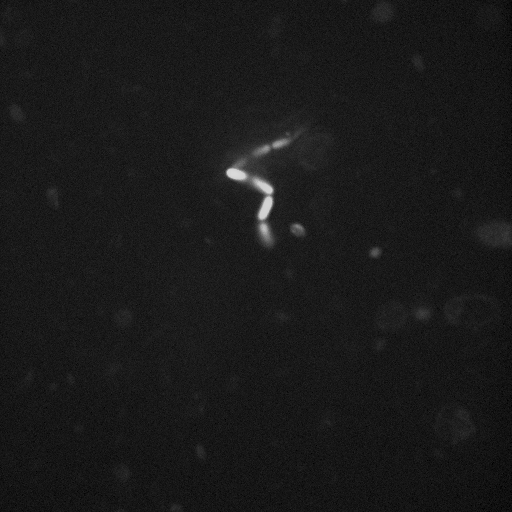

Supplement: S2 File — (ZIP) [file pcbi.1006986.s003.zip › extrait_4hKM16021/4h-Z692_29_w1sdcRFP.tif]

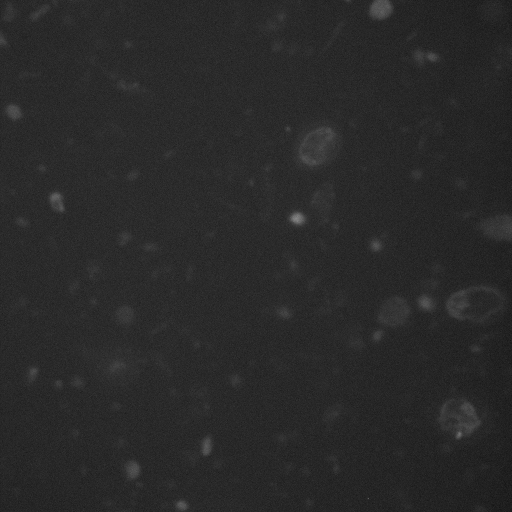

Supplement: S2 File — (ZIP) [file pcbi.1006986.s003.zip › extrait_4hKM16021/4h-Z692_29_w2sdcGFP.tif]

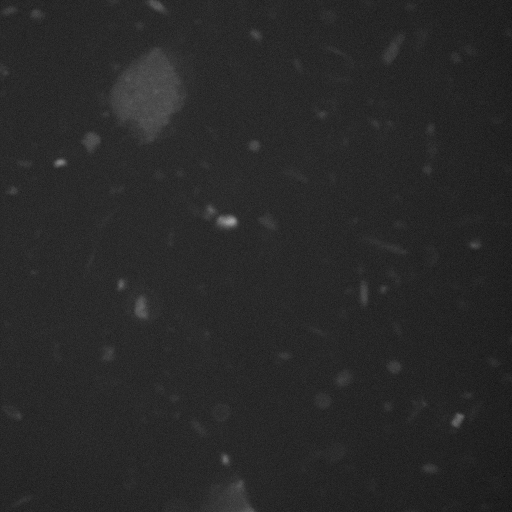

Supplement: S2 File — (ZIP) [file pcbi.1006986.s003.zip › extrait_4hKM16021/4h-Z692_12_w2sdcGFP.tif]

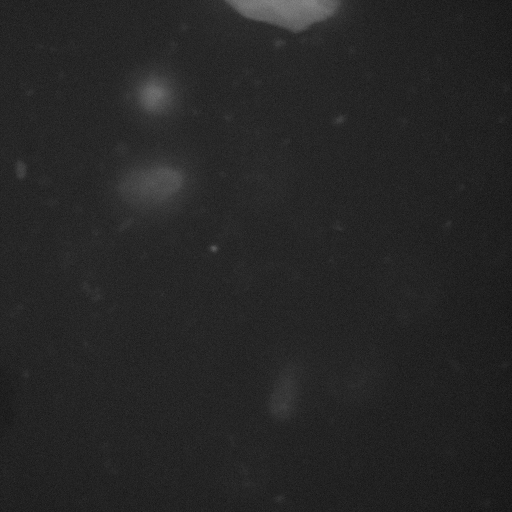

Supplement: S2 File — (ZIP) [file pcbi.1006986.s003.zip › extrait_4hKM16021/4h-Z692_11_w2sdcGFP.tif]

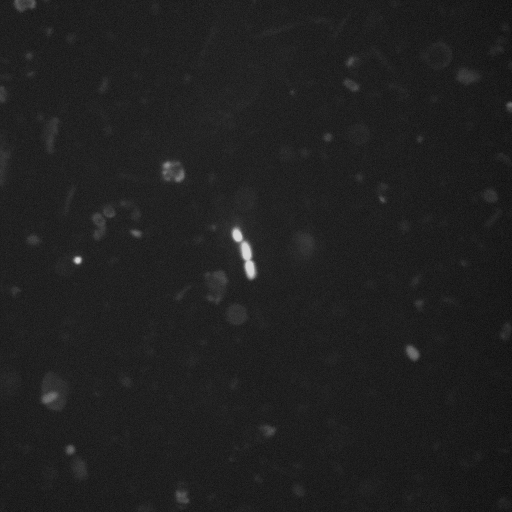

Supplement: S2 File — (ZIP) [file pcbi.1006986.s003.zip › extrait_4hKM16021/4h-Z693_18_w2sdcGFP.tif]

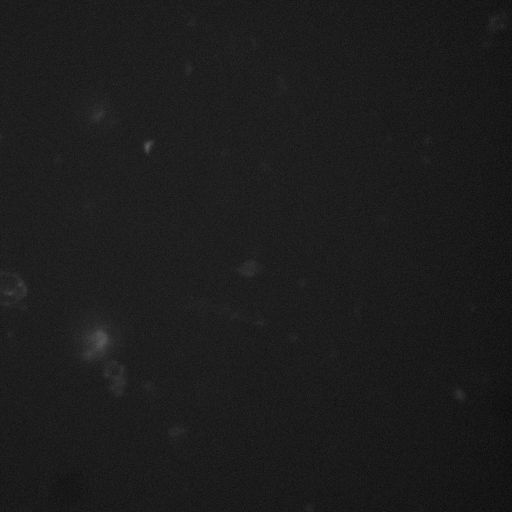

Supplement: S2 File — (ZIP) [file pcbi.1006986.s003.zip › extrait_4hKM16021/4h-Z694_1_w2sdcGFP.tif]

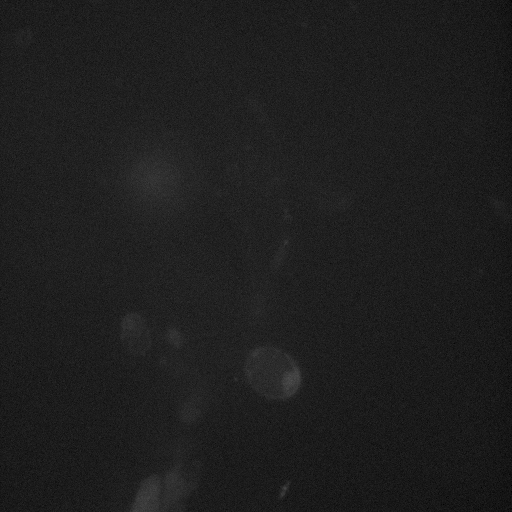

Supplement: S2 File — (ZIP) [file pcbi.1006986.s003.zip › extrait_4hKM16021/4h-Z692_6_w1sdcRFP.tif]

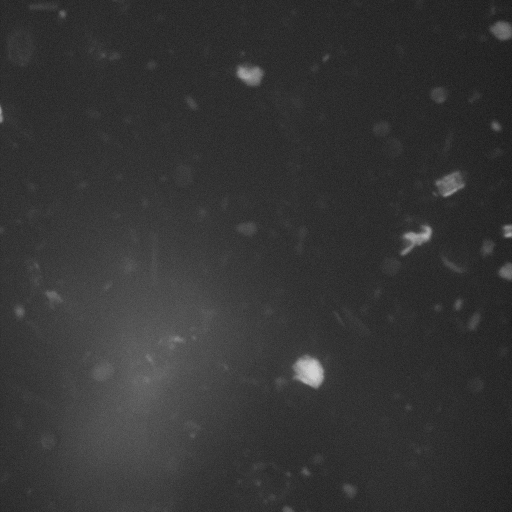

Supplement: S2 File — (ZIP) [file pcbi.1006986.s003.zip › extrait_4hKM16021/4h-Z693_22_w2sdcGFP.tif]

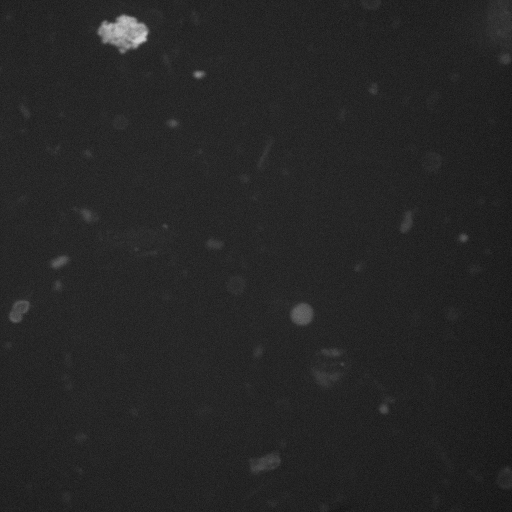

Supplement: S2 File — (ZIP) [file pcbi.1006986.s003.zip › extrait_4hKM16021/4h-Z693_4_w2sdcGFP.tif]

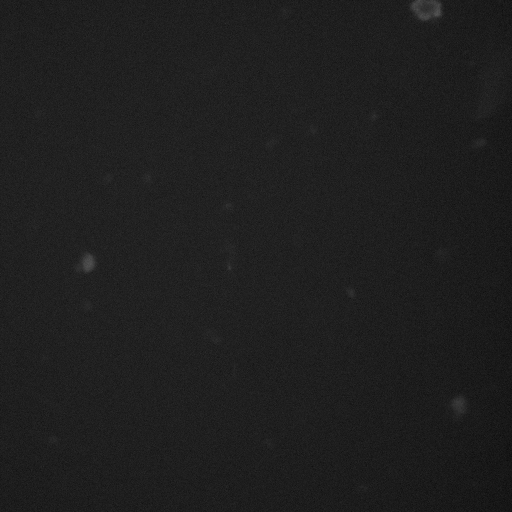

Supplement: S2 File — (ZIP) [file pcbi.1006986.s003.zip › extrait_4hKM16021/4h-Z694_14_w2sdcGFP.tif]

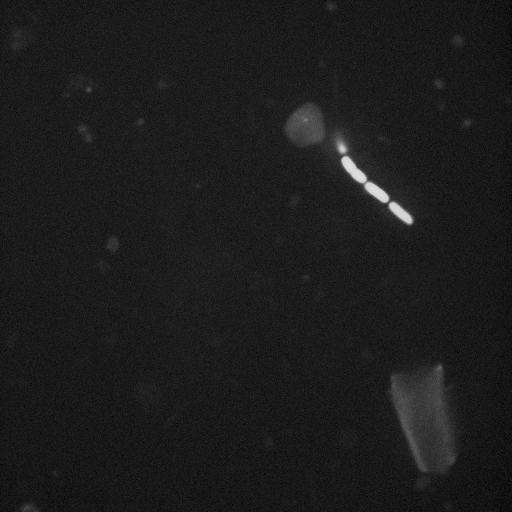

Supplement: S2 File — (ZIP) [file pcbi.1006986.s003.zip › extrait_4hKM16021/4h-Z694_16_w1sdcRFP.tif]

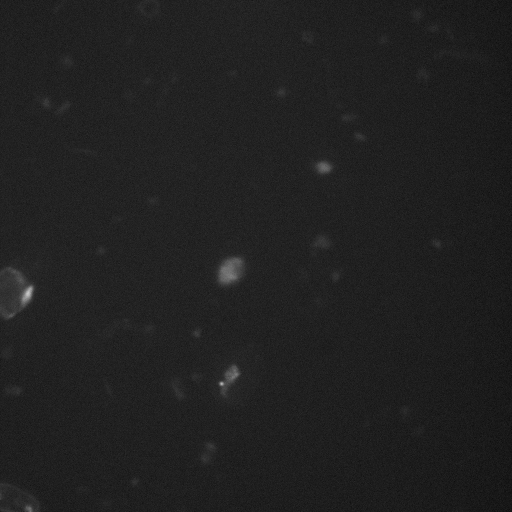

Supplement: S2 File — (ZIP) [file pcbi.1006986.s003.zip › extrait_4hKM16021/4h-Z694_12_w2sdcGFP.tif]

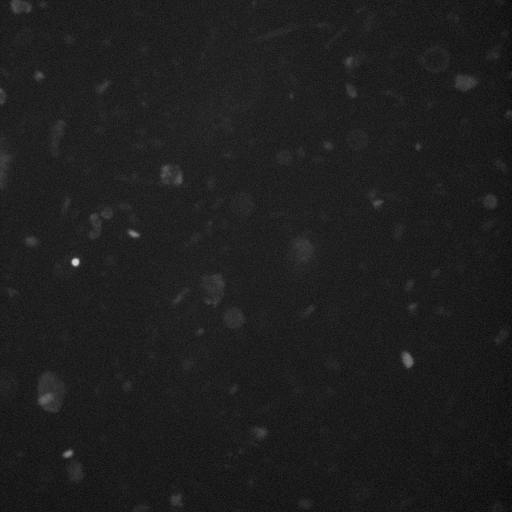

Supplement: S2 File — (ZIP) [file pcbi.1006986.s003.zip › extrait_4hKM16021/4h-Z693_18_w1sdcRFP.tif]

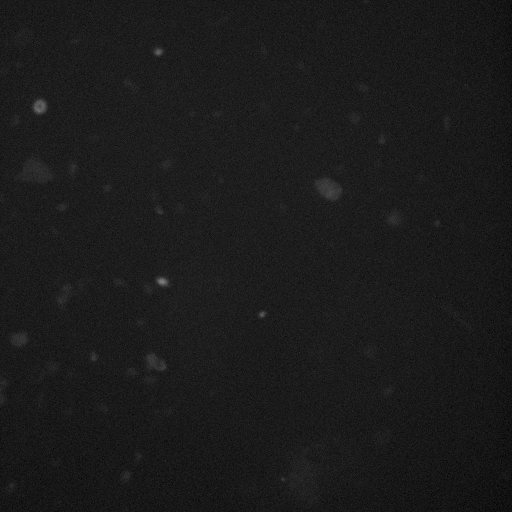

Supplement: S2 File — (ZIP) [file pcbi.1006986.s003.zip › extrait_4hKM16021/4h-Z694_31_w1sdcRFP.tif]

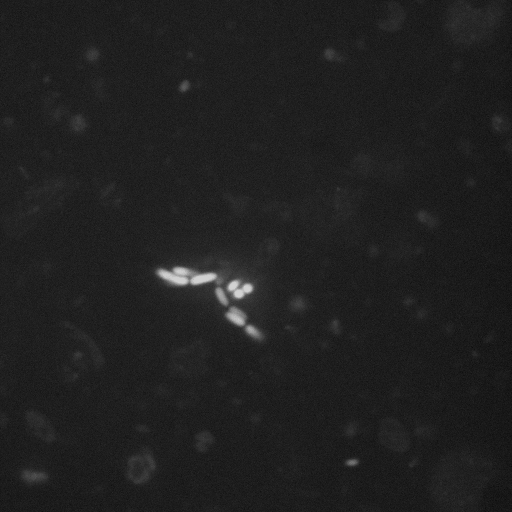

Supplement: S2 File — (ZIP) [file pcbi.1006986.s003.zip › extrait_4hKM16021/4h-Z693_30_w2sdcGFP.tif]

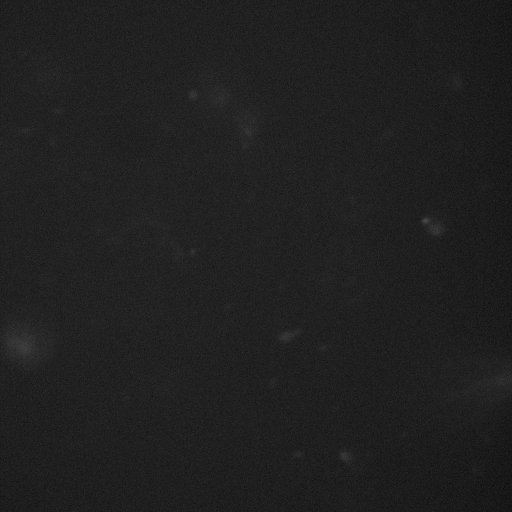

Supplement: S2 File — (ZIP) [file pcbi.1006986.s003.zip › extrait_4hKM16021/4h-Z694_23_w2sdcGFP.tif]

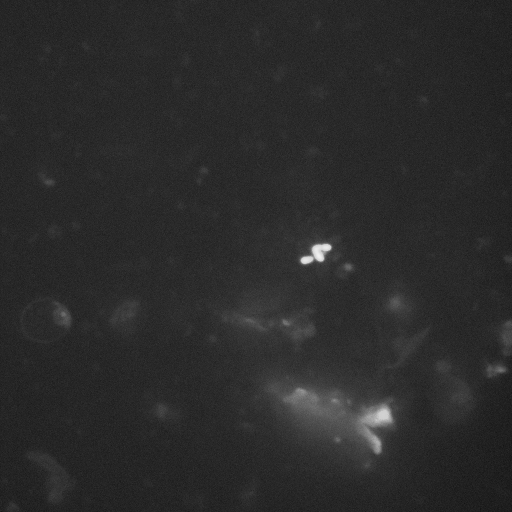

Supplement: S2 File — (ZIP) [file pcbi.1006986.s003.zip › extrait_4hKM16021/4h-Z693_25_w2sdcGFP.tif]

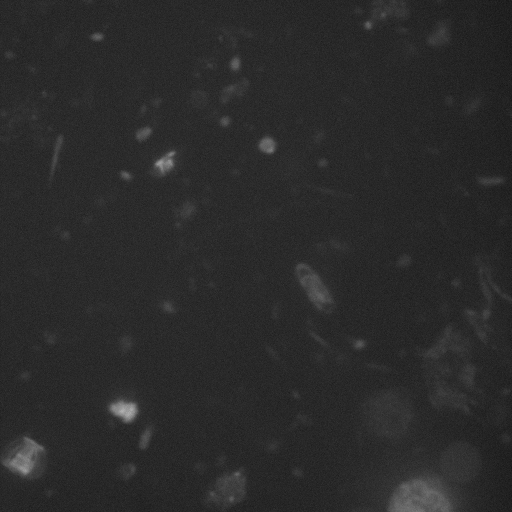

Supplement: S2 File — (ZIP) [file pcbi.1006986.s003.zip › extrait_4hKM16021/4h-Z692_13_w2sdcGFP.tif]

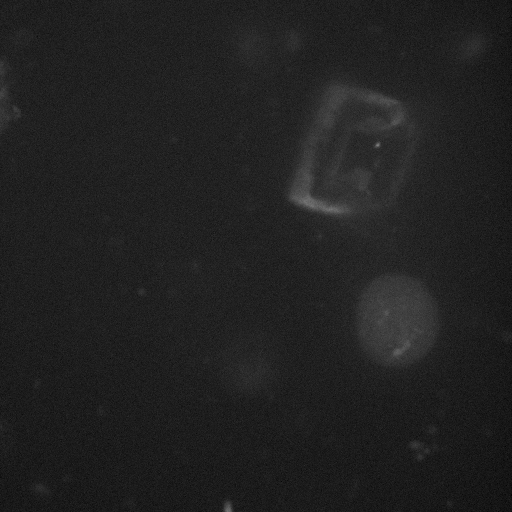

Supplement: S2 File — (ZIP) [file pcbi.1006986.s003.zip › extrait_4hKM16021/4h-Z692_4_w1sdcRFP.tif]

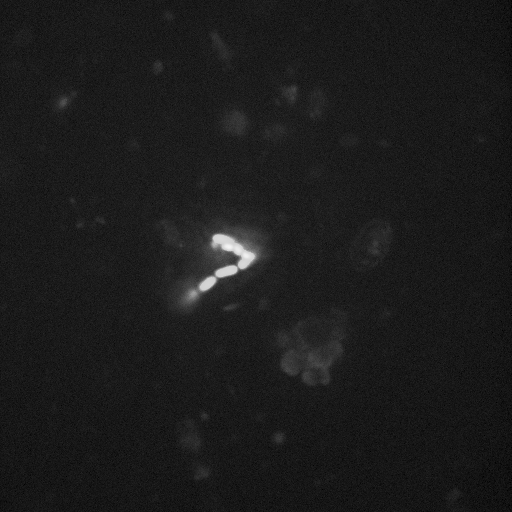

Supplement: S2 File — (ZIP) [file pcbi.1006986.s003.zip › extrait_4hKM16021/4h-Z693_26_w1sdcRFP.tif]

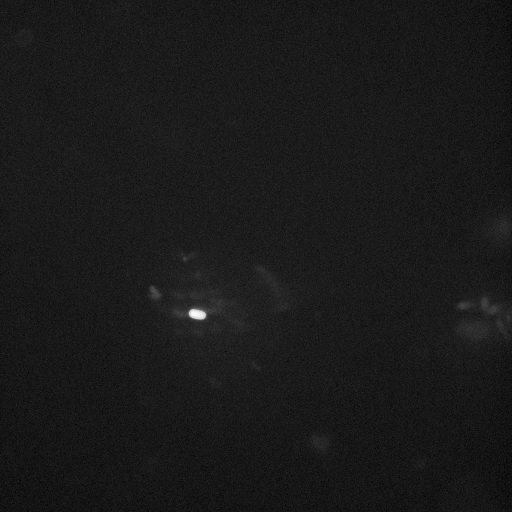

Supplement: S2 File — (ZIP) [file pcbi.1006986.s003.zip › extrait_4hKM16021/4h-Z692_2_w1sdcRFP.tif]

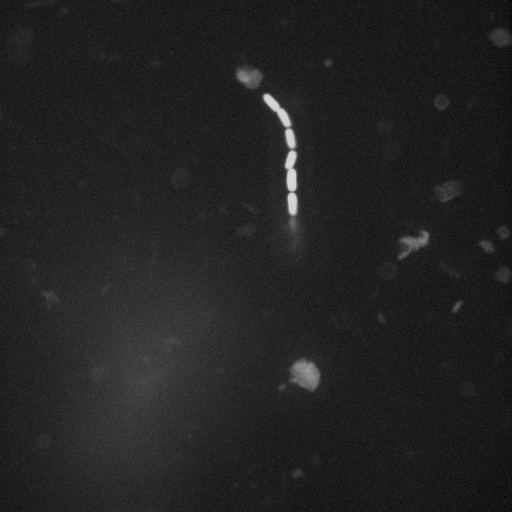

Supplement: S2 File — (ZIP) [file pcbi.1006986.s003.zip › extrait_4hKM16021/4h-Z693_22_w1sdcRFP.tif]

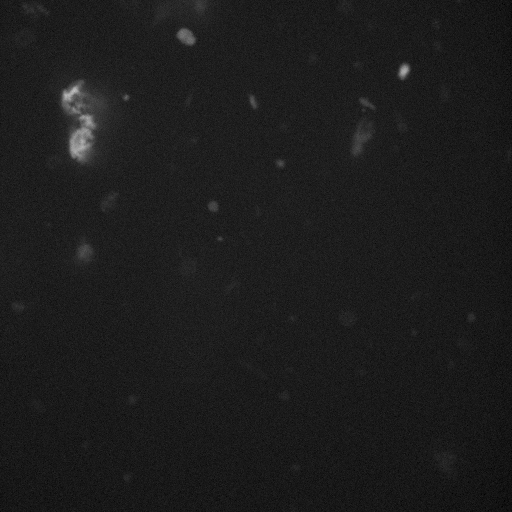

Supplement: S2 File — (ZIP) [file pcbi.1006986.s003.zip › extrait_4hKM16021/4h-Z694_17_w1sdcRFP.tif]

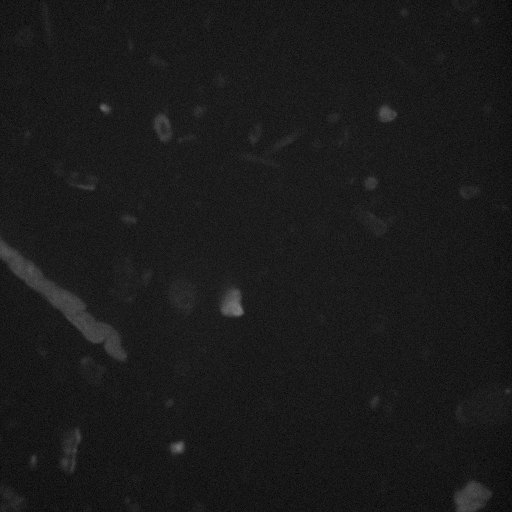

Supplement: S2 File — (ZIP) [file pcbi.1006986.s003.zip › extrait_4hKM16021/4h-Z693_13_w1sdcRFP.tif]

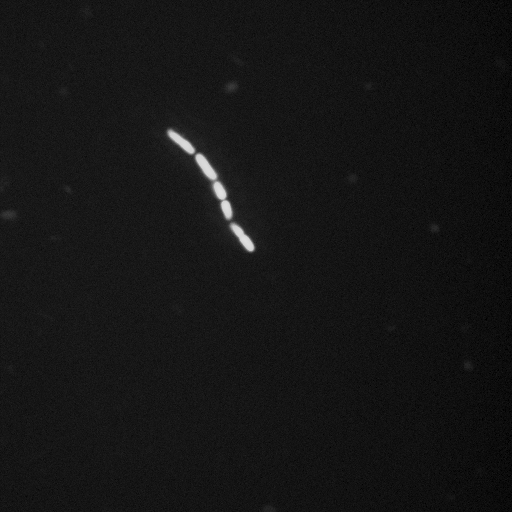

Supplement: S2 File — (ZIP) [file pcbi.1006986.s003.zip › extrait_4hKM16021/4h-Z694_27_w2sdcGFP.tif]

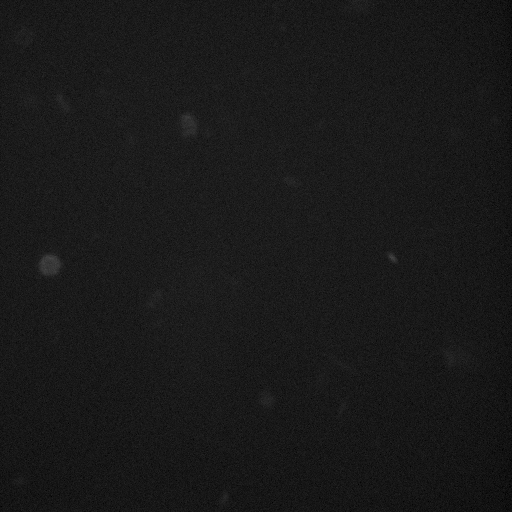

Supplement: S2 File — (ZIP) [file pcbi.1006986.s003.zip › extrait_4hKM16021/4h-Z694_10_w1sdcRFP.tif]

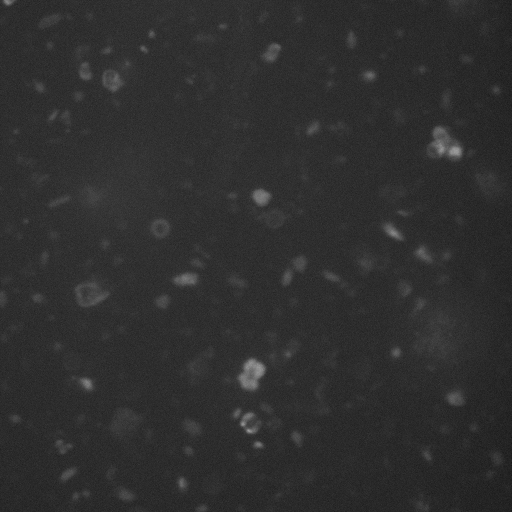

Supplement: S2 File — (ZIP) [file pcbi.1006986.s003.zip › extrait_4hKM16021/4h-Z693_16_w2sdcGFP.tif]

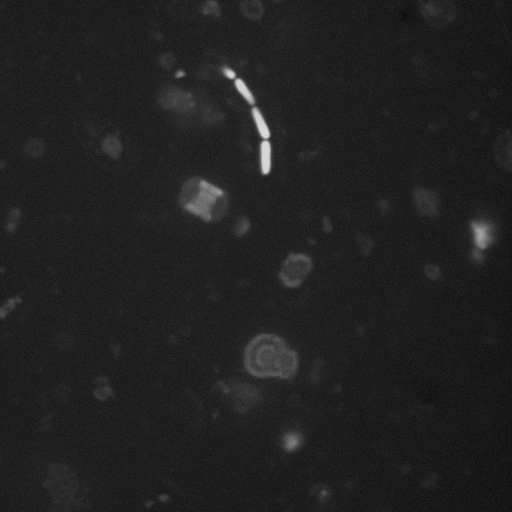

Supplement: S2 File — (ZIP) [file pcbi.1006986.s003.zip › extrait_4hKM16021/4h-Z693_27_w2sdcGFP.tif]

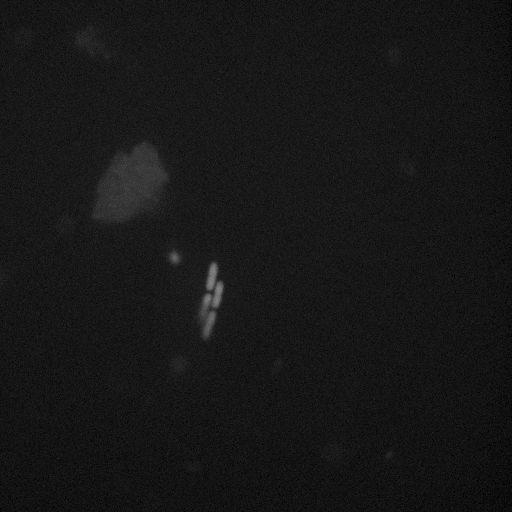

Supplement: S2 File — (ZIP) [file pcbi.1006986.s003.zip › extrait_4hKM16021/4h-Z694_7_w1sdcRFP.tif]

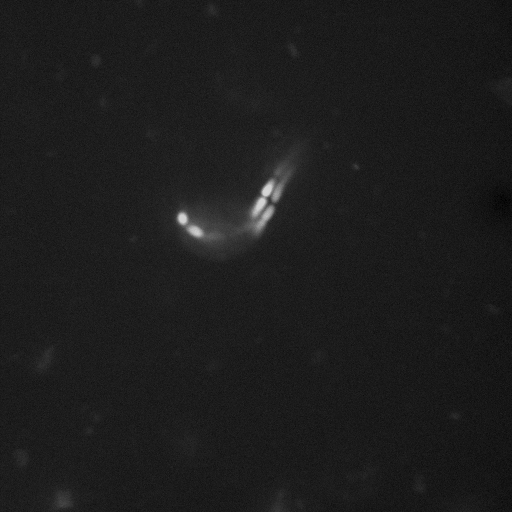

Supplement: S2 File — (ZIP) [file pcbi.1006986.s003.zip › extrait_4hKM16021/4h-Z692_31_w2sdcGFP.tif]

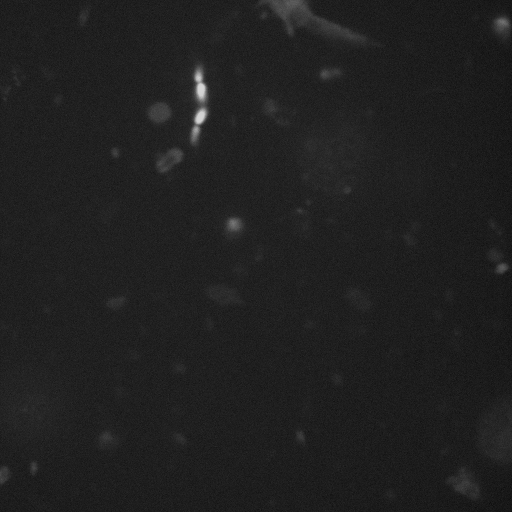

Supplement: S2 File — (ZIP) [file pcbi.1006986.s003.zip › extrait_4hKM16021/4h-Z693_7_w2sdcGFP.tif]

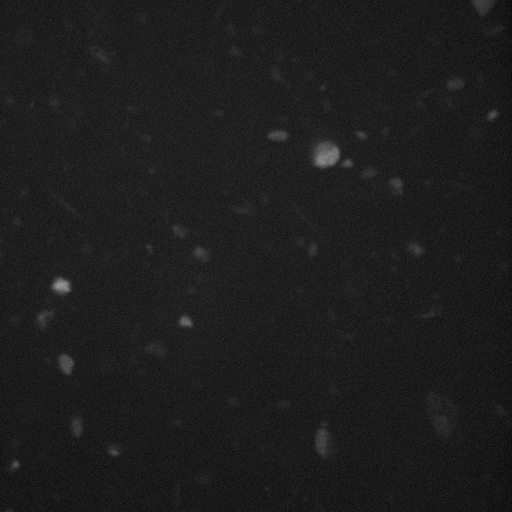

Supplement: S2 File — (ZIP) [file pcbi.1006986.s003.zip › extrait_4hKM16021/4h-Z692_20_w1sdcRFP.tif]

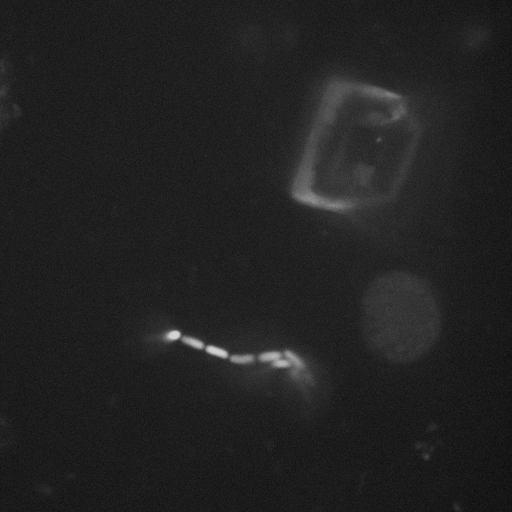

Supplement: S2 File — (ZIP) [file pcbi.1006986.s003.zip › extrait_4hKM16021/4h-Z692_4_w2sdcGFP.tif]

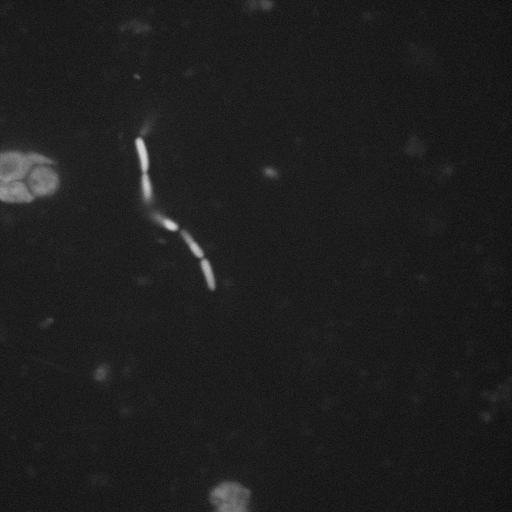

Supplement: S2 File — (ZIP) [file pcbi.1006986.s003.zip › extrait_4hKM16021/4h-Z692_34_w2sdcGFP.tif]

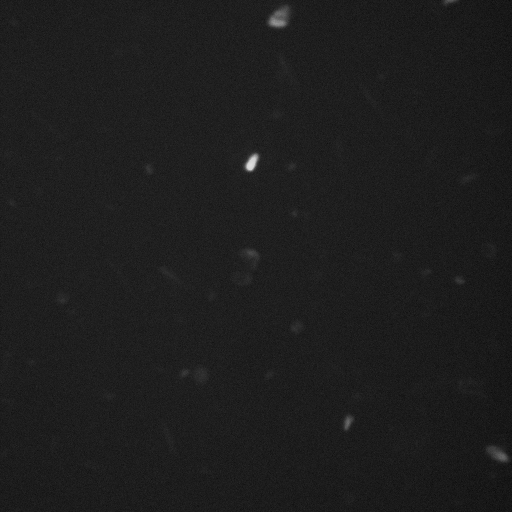

Supplement: S2 File — (ZIP) [file pcbi.1006986.s003.zip › extrait_4hKM16021/4h-Z692_3_w2sdcGFP.tif]

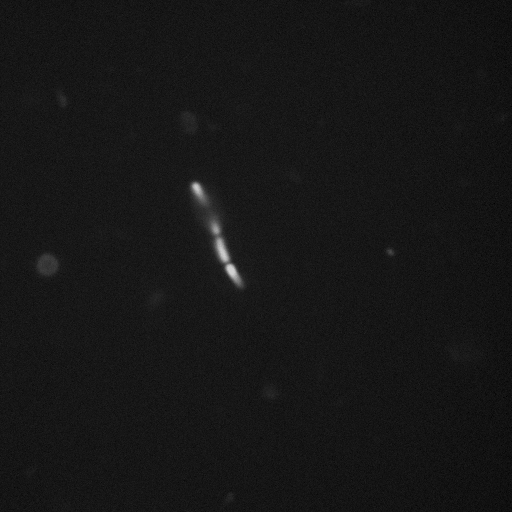

Supplement: S2 File — (ZIP) [file pcbi.1006986.s003.zip › extrait_4hKM16021/4h-Z694_10_w2sdcGFP.tif]

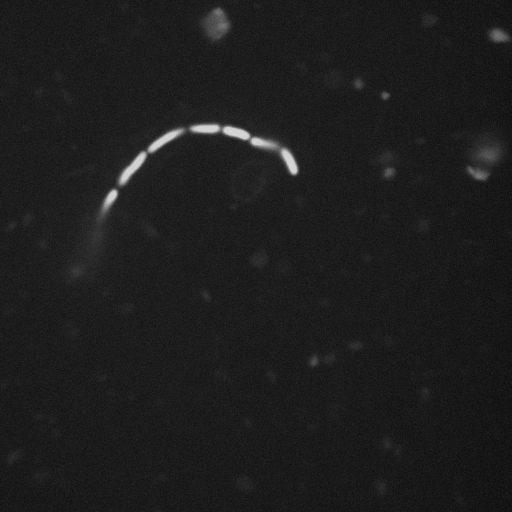

Supplement: S2 File — (ZIP) [file pcbi.1006986.s003.zip › extrait_4hKM16021/4h-Z692_22_w2sdcGFP.tif]

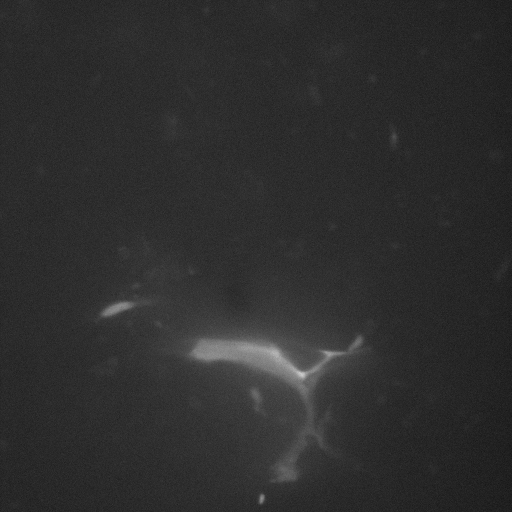

Supplement: S2 File — (ZIP) [file pcbi.1006986.s003.zip › extrait_4hKM16021/4h-Z692_14_w2sdcGFP.tif]

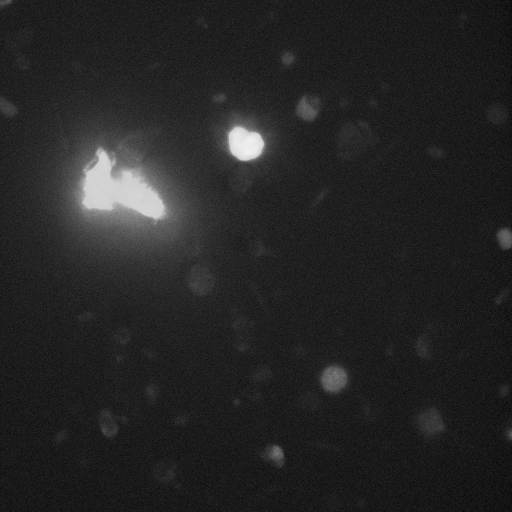

Supplement: S2 File — (ZIP) [file pcbi.1006986.s003.zip › extrait_4hKM16021/4h-Z695_1_w1sdcRFP.tif]

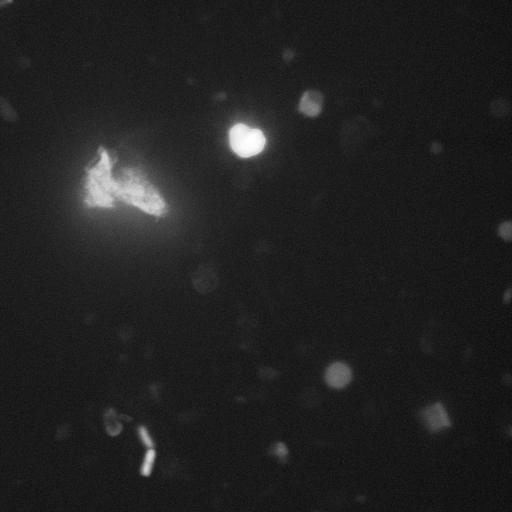

Supplement: S2 File — (ZIP) [file pcbi.1006986.s003.zip › extrait_4hKM16021/4h-Z695_1_w2sdcGFP.tif]

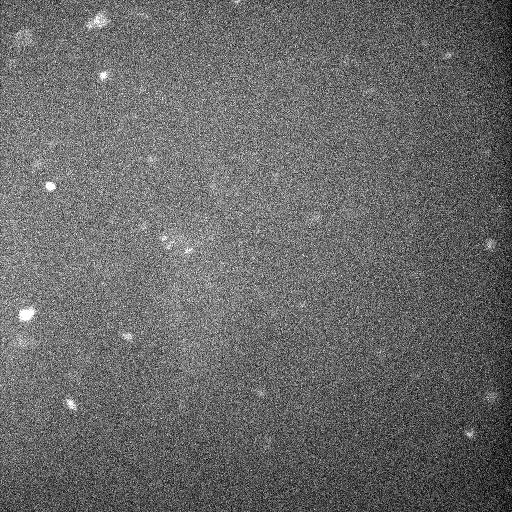

Supplement: S2 File — (ZIP) [file pcbi.1006986.s003.zip › extrait_4hKM16021/4h-Z693_1_w1sdcRFP.tif]

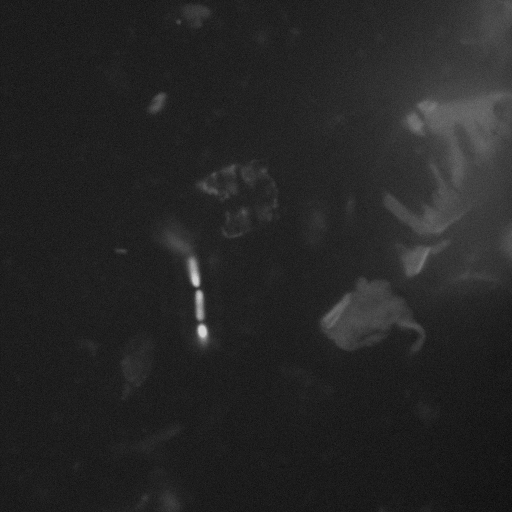

Supplement: S2 File — (ZIP) [file pcbi.1006986.s003.zip › extrait_4hKM16021/4h-Z692_9_w2sdcGFP.tif]

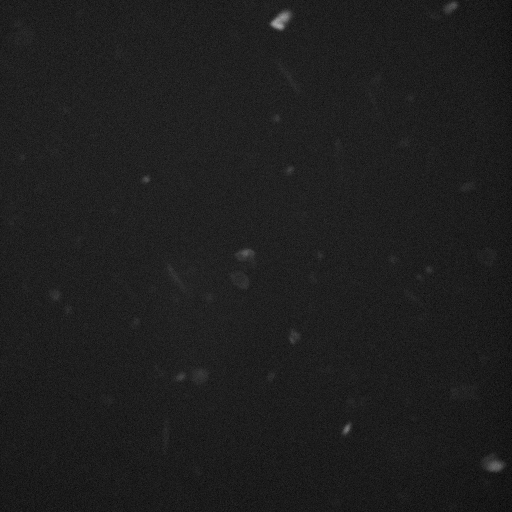

Supplement: S2 File — (ZIP) [file pcbi.1006986.s003.zip › extrait_4hKM16021/4h-Z692_3_w1sdcRFP.tif]

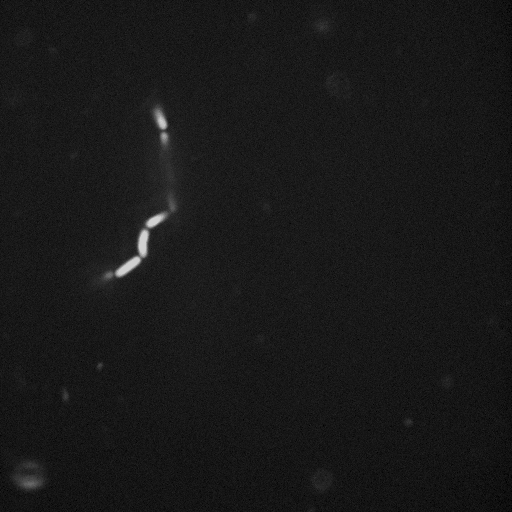

Supplement: S2 File — (ZIP) [file pcbi.1006986.s003.zip › extrait_4hKM16021/4h-Z694_22_w1sdcRFP.tif]

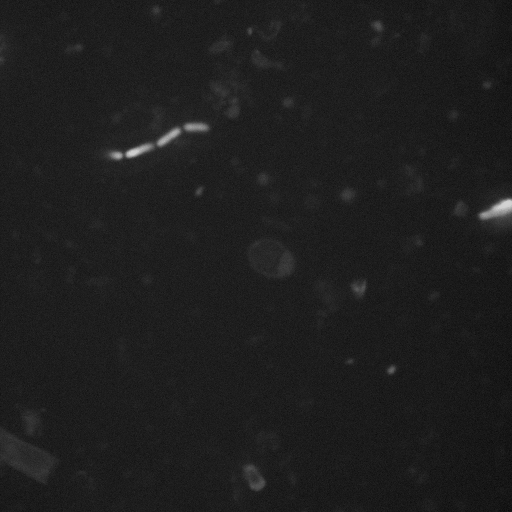

Supplement: S2 File — (ZIP) [file pcbi.1006986.s003.zip › extrait_4hKM16021/4h-Z693_19_w2sdcGFP.tif]

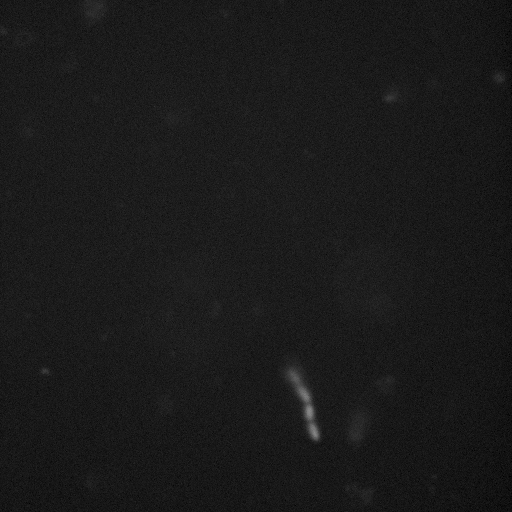

Supplement: S2 File — (ZIP) [file pcbi.1006986.s003.zip › extrait_4hKM16021/4h-Z692_15_w1sdcRFP.tif]

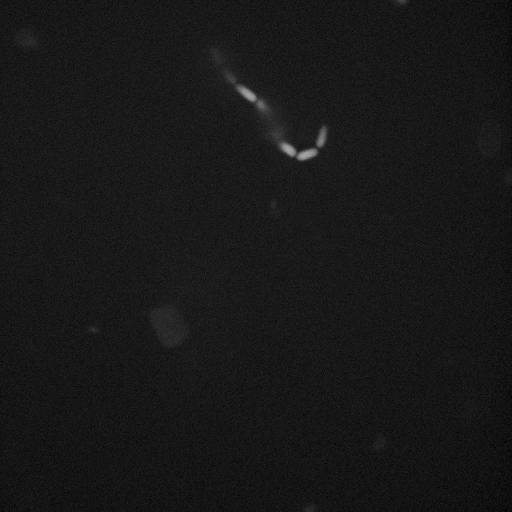

Supplement: S2 File — (ZIP) [file pcbi.1006986.s003.zip › extrait_4hKM16021/4h-Z694_29_w1sdcRFP.tif]

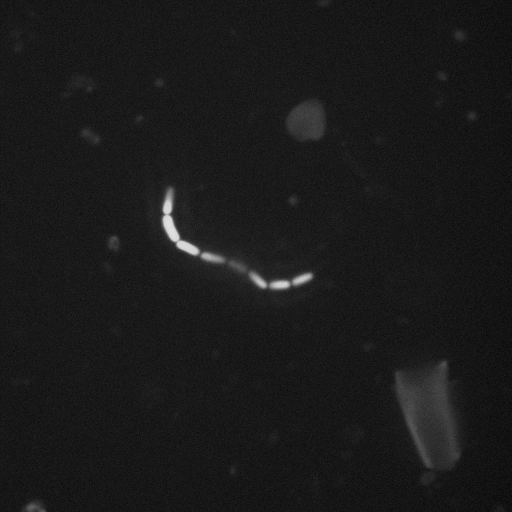

Supplement: S2 File — (ZIP) [file pcbi.1006986.s003.zip › extrait_4hKM16021/4h-Z694_16_w2sdcGFP.tif]

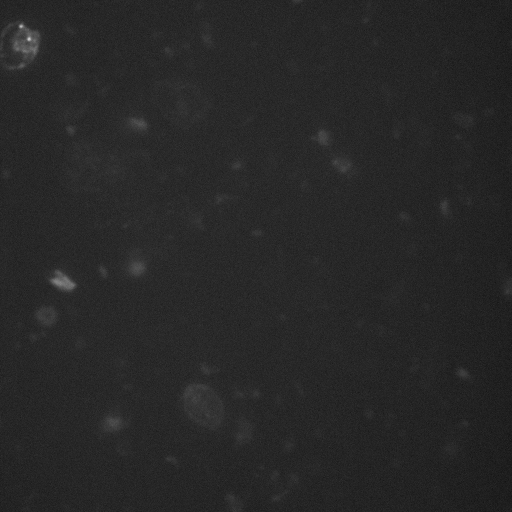

Supplement: S2 File — (ZIP) [file pcbi.1006986.s003.zip › extrait_4hKM16021/4h-Z693_12_w2sdcGFP.tif]

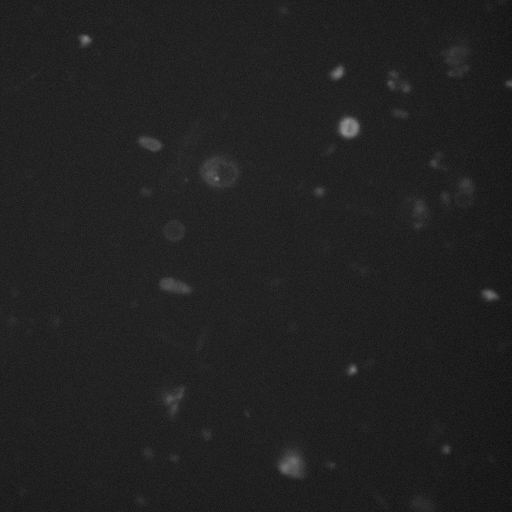

Supplement: S2 File — (ZIP) [file pcbi.1006986.s003.zip › extrait_4hKM16021/4h-Z694_18_w2sdcGFP.tif]

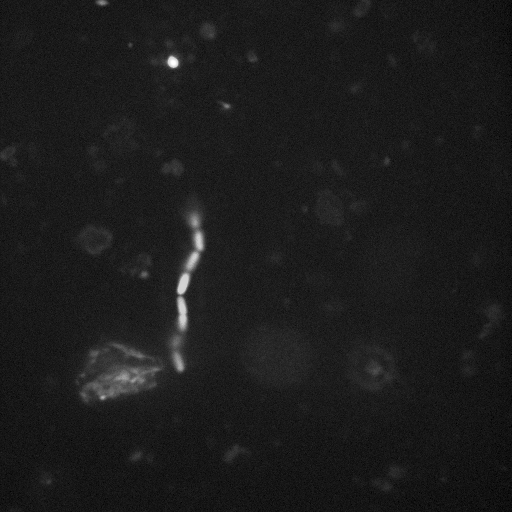

Supplement: S2 File — (ZIP) [file pcbi.1006986.s003.zip › extrait_4hKM16021/4h-Z693_23_w1sdcRFP.tif]

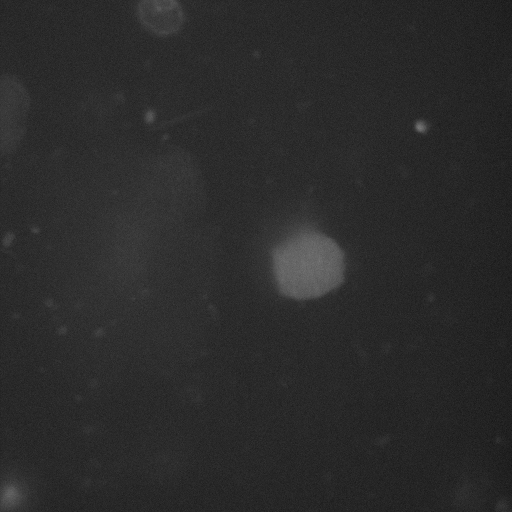

Supplement: S2 File — (ZIP) [file pcbi.1006986.s003.zip › extrait_4hKM16021/4h-Z692_23_w2sdcGFP.tif]

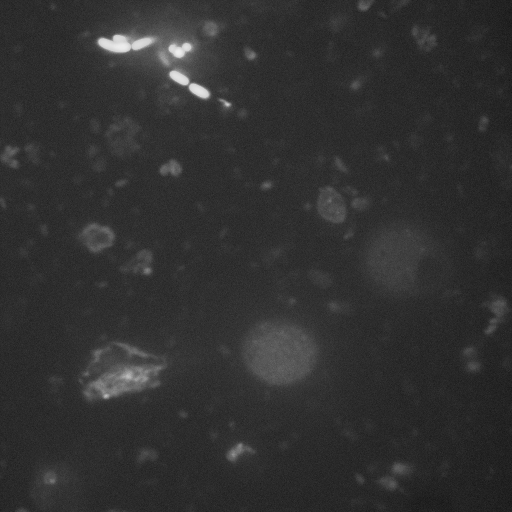

Supplement: S2 File — (ZIP) [file pcbi.1006986.s003.zip › extrait_4hKM16021/4h-Z693_23_w2sdcGFP.tif]

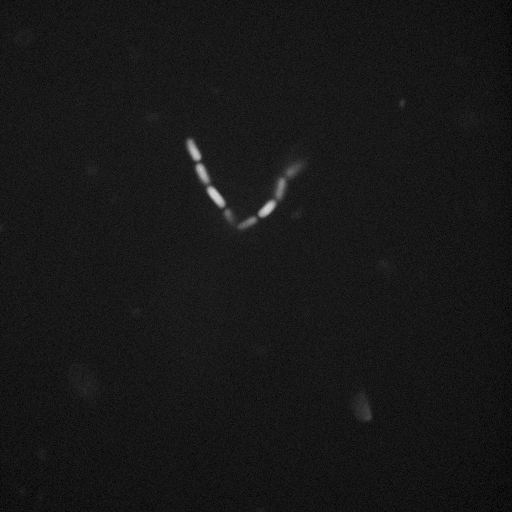

Supplement: S2 File — (ZIP) [file pcbi.1006986.s003.zip › extrait_4hKM16021/4h-Z694_8_w1sdcRFP.tif]

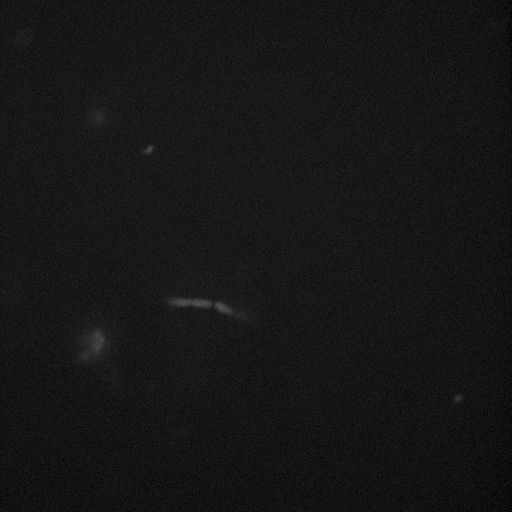

Supplement: S2 File — (ZIP) [file pcbi.1006986.s003.zip › extrait_4hKM16021/4h-Z694_1_w1sdcRFP.tif]

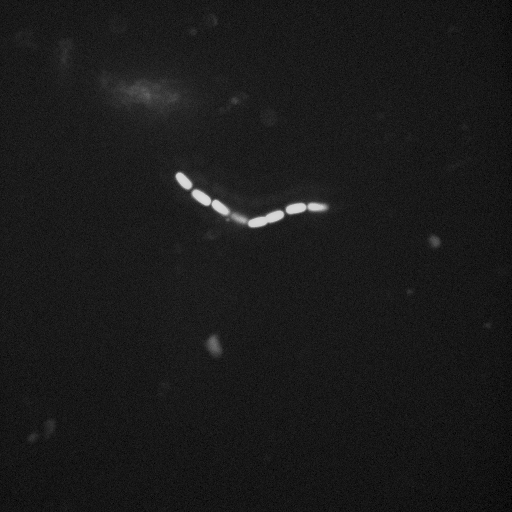

Supplement: S2 File — (ZIP) [file pcbi.1006986.s003.zip › extrait_4hKM16021/4h-Z694_28_w1sdcRFP.tif]

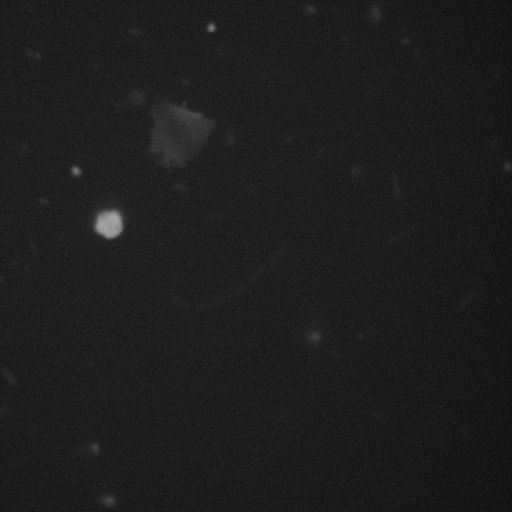

Supplement: S2 File — (ZIP) [file pcbi.1006986.s003.zip › extrait_4hKM16021/4h-Z694_13_w2sdcGFP.tif]

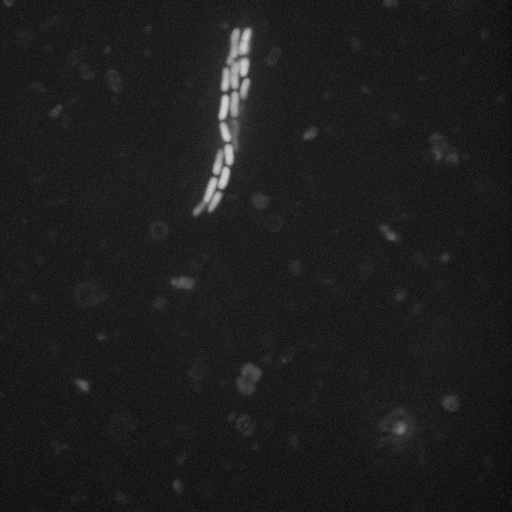

Supplement: S2 File — (ZIP) [file pcbi.1006986.s003.zip › extrait_4hKM16021/4h-Z693_16_w1sdcRFP.tif]

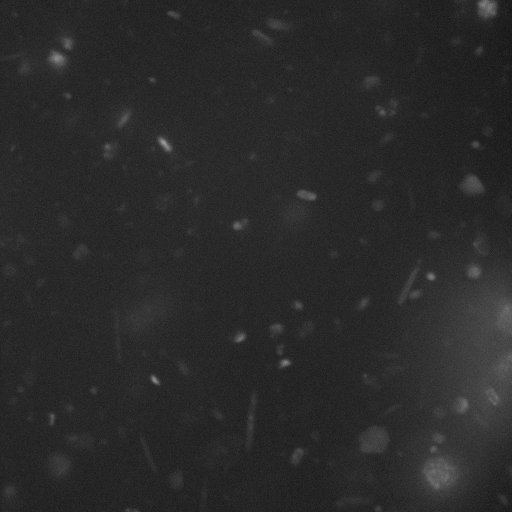

Supplement: S2 File — (ZIP) [file pcbi.1006986.s003.zip › extrait_4hKM16021/4h-Z695_3_w2sdcGFP.tif]

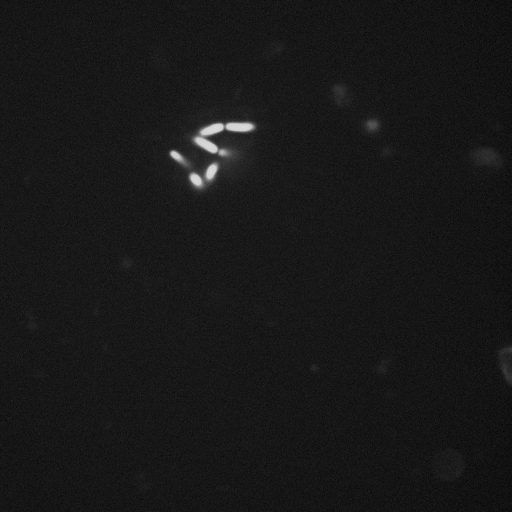

Supplement: S2 File — (ZIP) [file pcbi.1006986.s003.zip › extrait_4hKM16021/4h-Z694_9_w2sdcGFP.tif]

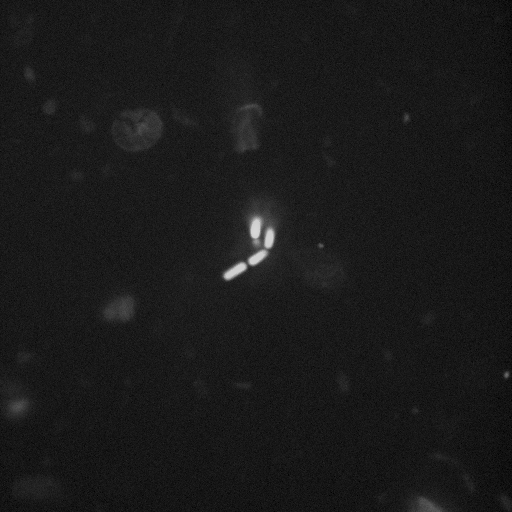

Supplement: S2 File — (ZIP) [file pcbi.1006986.s003.zip › extrait_4hKM16021/4h-Z692_24_w1sdcRFP.tif]

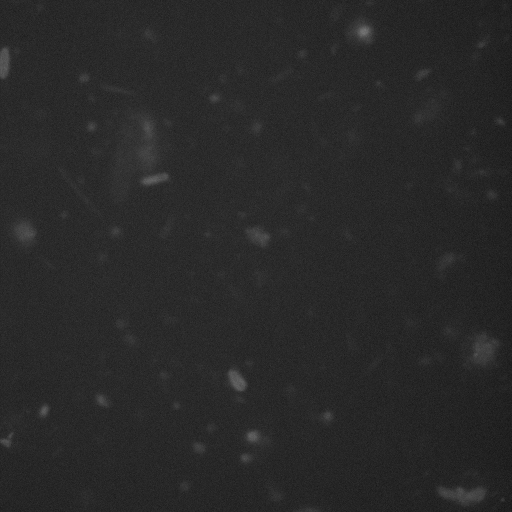

Supplement: S2 File — (ZIP) [file pcbi.1006986.s003.zip › extrait_4hKM16021/4h-Z692_26_w2sdcGFP.tif]

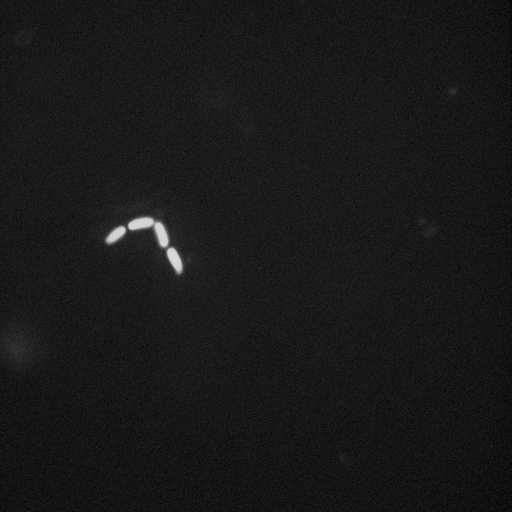

Supplement: S2 File — (ZIP) [file pcbi.1006986.s003.zip › extrait_4hKM16021/4h-Z694_23_w1sdcRFP.tif]

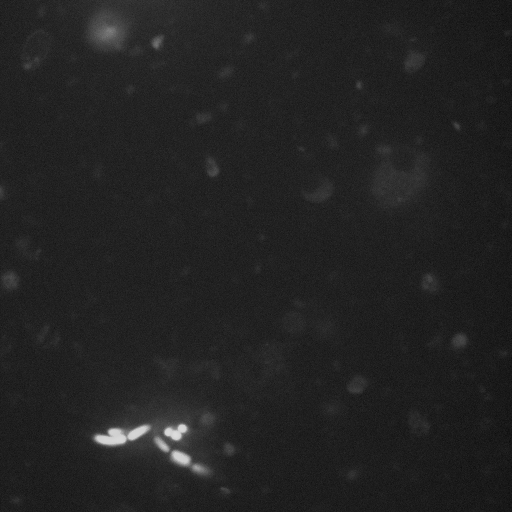

Supplement: S2 File — (ZIP) [file pcbi.1006986.s003.zip › extrait_4hKM16021/4h-Z693_24_w2sdcGFP.tif]

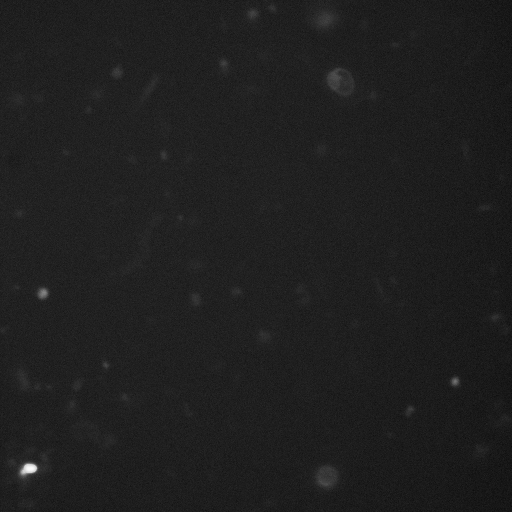

Supplement: S2 File — (ZIP) [file pcbi.1006986.s003.zip › extrait_4hKM16021/4h-Z694_22_w2sdcGFP.tif]

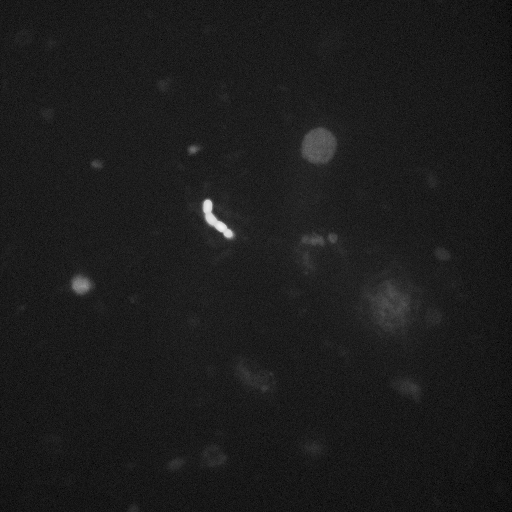

Supplement: S2 File — (ZIP) [file pcbi.1006986.s003.zip › extrait_4hKM16021/4h-Z694_20_w1sdcRFP.tif]

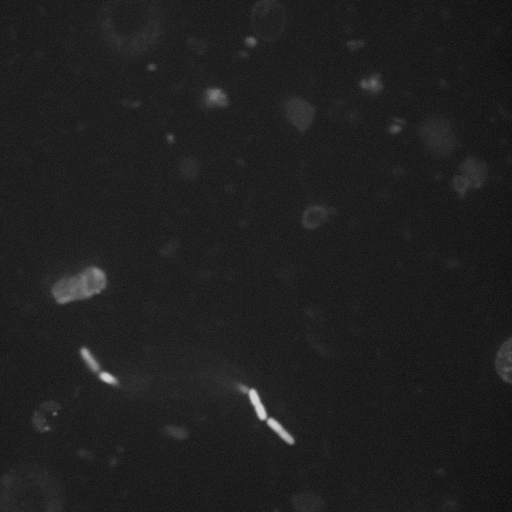

Supplement: S2 File — (ZIP) [file pcbi.1006986.s003.zip › extrait_4hKM16021/4h-Z693_20_w2sdcGFP.tif]

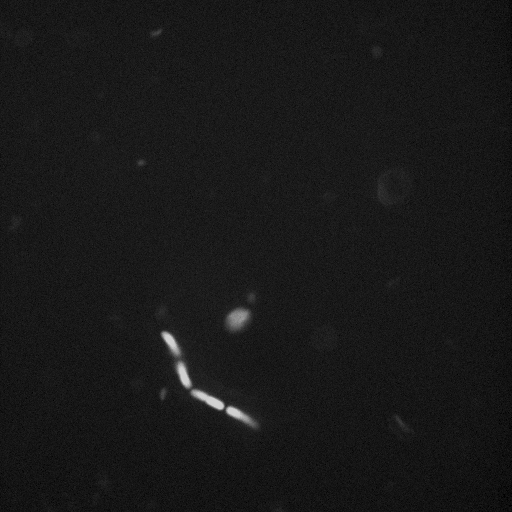

Supplement: S2 File — (ZIP) [file pcbi.1006986.s003.zip › extrait_4hKM16021/4h-Z694_24_w1sdcRFP.tif]

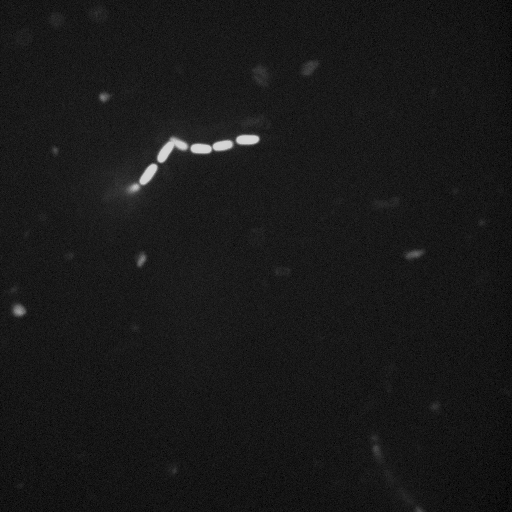

Supplement: S2 File — (ZIP) [file pcbi.1006986.s003.zip › extrait_4hKM16021/4h-Z694_30_w1sdcRFP.tif]

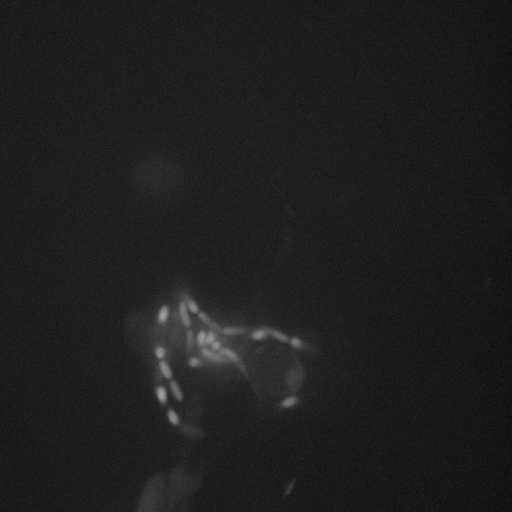

Supplement: S2 File — (ZIP) [file pcbi.1006986.s003.zip › extrait_4hKM16021/4h-Z692_6_w2sdcGFP.tif]

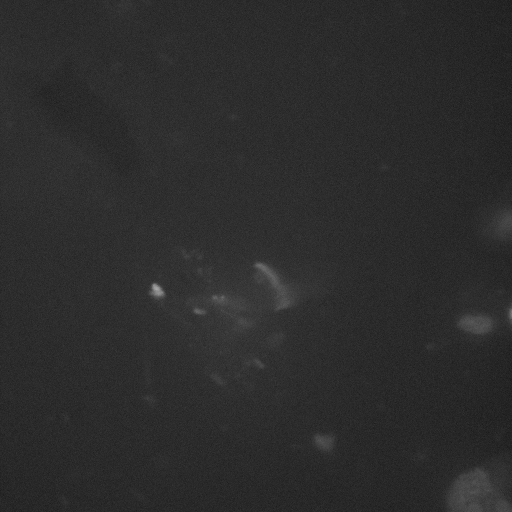

Supplement: S2 File — (ZIP) [file pcbi.1006986.s003.zip › extrait_4hKM16021/4h-Z692_2_w2sdcGFP.tif]

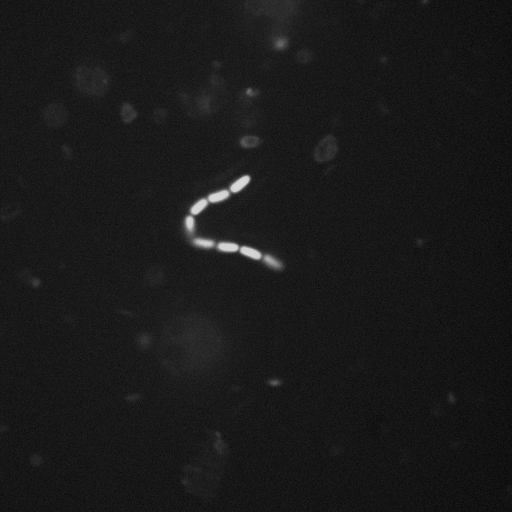

Supplement: S2 File — (ZIP) [file pcbi.1006986.s003.zip › extrait_4hKM16021/4h-Z694_21_w2sdcGFP.tif]

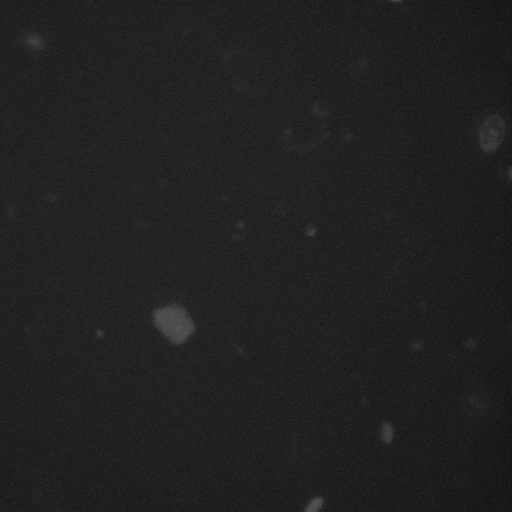

Supplement: S2 File — (ZIP) [file pcbi.1006986.s003.zip › extrait_4hKM16021/4h-Z694_29_w2sdcGFP.tif]

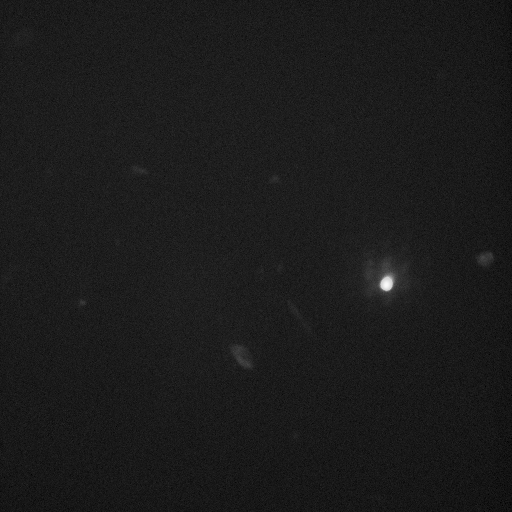

Supplement: S2 File — (ZIP) [file pcbi.1006986.s003.zip › extrait_4hKM16021/4h-Z694_4_w1sdcRFP.tif]

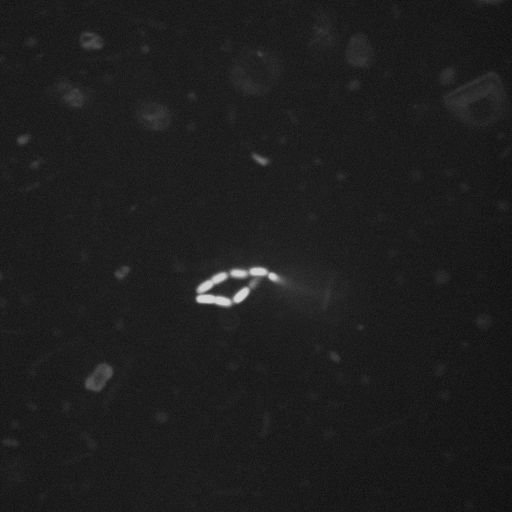

Supplement: S2 File — (ZIP) [file pcbi.1006986.s003.zip › extrait_4hKM16021/4h-Z692_33_w2sdcGFP.tif]

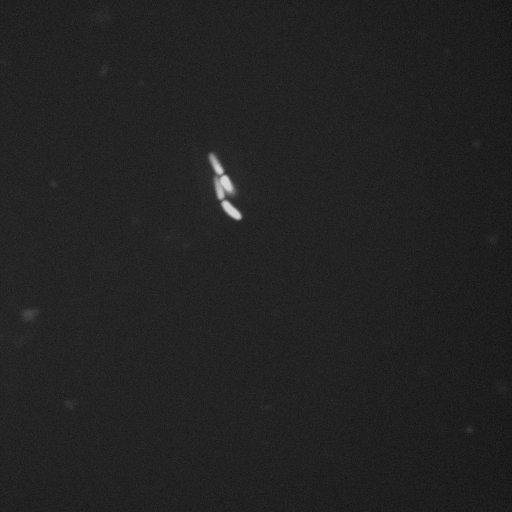

Supplement: S2 File — (ZIP) [file pcbi.1006986.s003.zip › extrait_4hKM16021/4h-Z693_1_w2sdcGFP.tif]

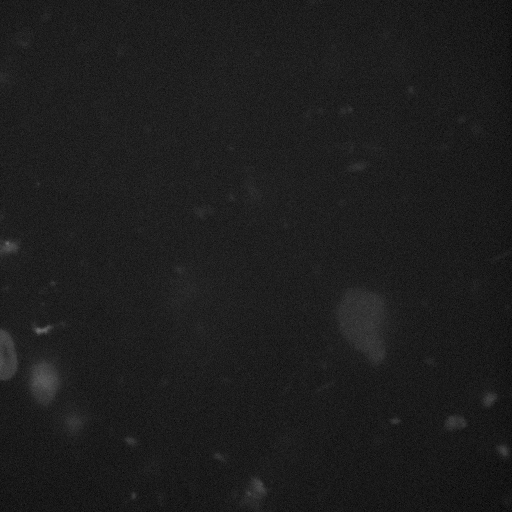

Supplement: S2 File — (ZIP) [file pcbi.1006986.s003.zip › extrait_4hKM16021/4h-Z692_18_w1sdcRFP.tif]

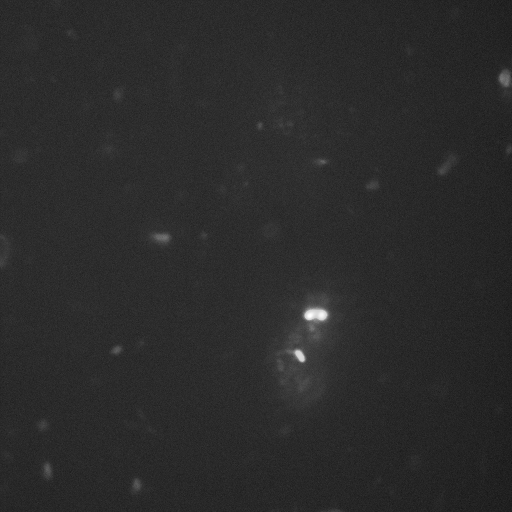

Supplement: S2 File — (ZIP) [file pcbi.1006986.s003.zip › extrait_4hKM16021/4h-Z692_1_w2sdcGFP.tif]

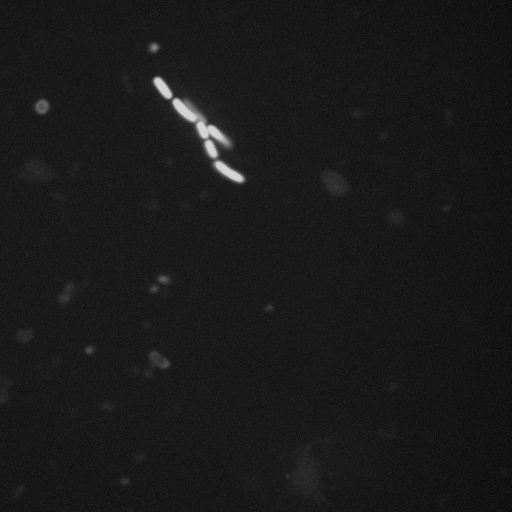

Supplement: S2 File — (ZIP) [file pcbi.1006986.s003.zip › extrait_4hKM16021/4h-Z694_31_w2sdcGFP.tif]

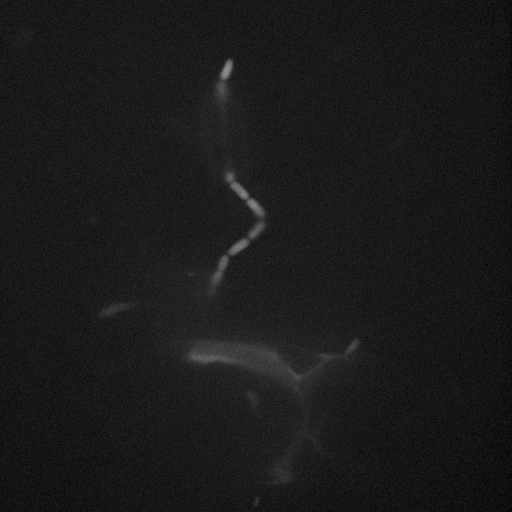

Supplement: S2 File — (ZIP) [file pcbi.1006986.s003.zip › extrait_4hKM16021/4h-Z692_14_w1sdcRFP.tif]
