# Supplementary figures and images for: Enchained growth and cluster dislocation: A possible mechanism for microbiota homeostasis (part 3 of 10)
Source: PLoS Comput Biol. 2019 May 3;15(5):e1006986. doi: 10.1371/journal.pcbi.1006986 (PMC6519844; doi:10.1371/journal.pcbi.1006986)

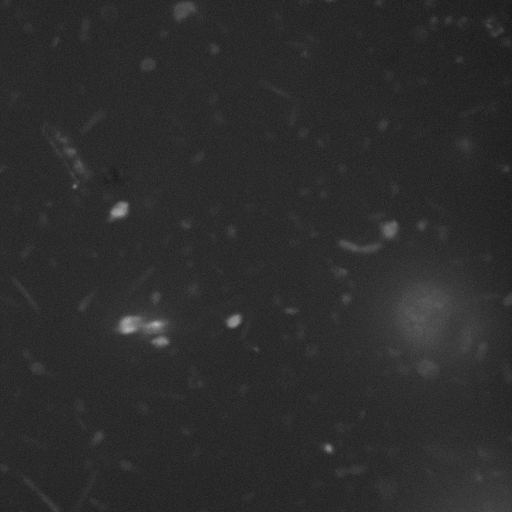

Supplement: S2 File — (ZIP) [file pcbi.1006986.s003.zip › extrait_4hKM16021/4h-Z692_28_w2sdcGFP.tif]

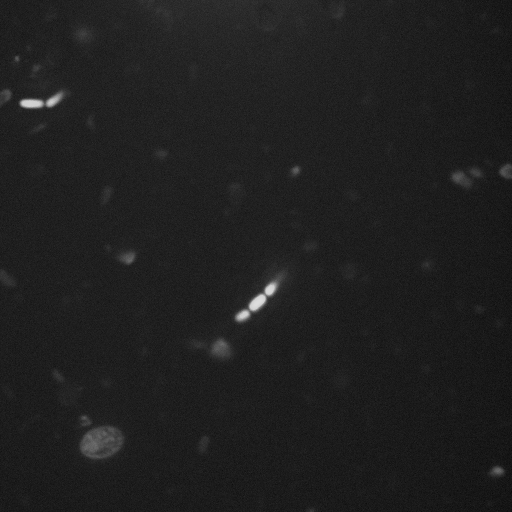

Supplement: S2 File — (ZIP) [file pcbi.1006986.s003.zip › extrait_4hKM16021/4h-Z693_6_w2sdcGFP.tif]

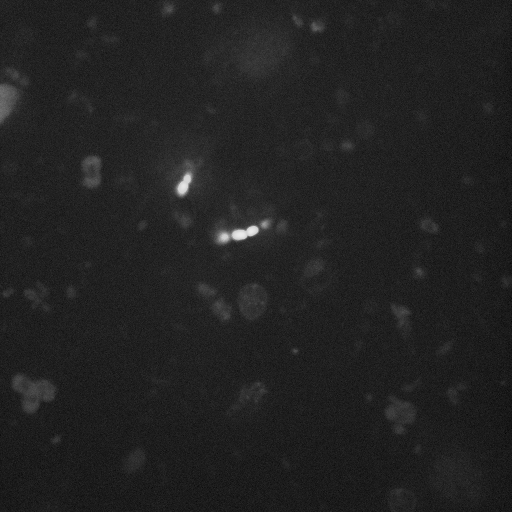

Supplement: S2 File — (ZIP) [file pcbi.1006986.s003.zip › extrait_4hKM16021/4h-Z693_31_w1sdcRFP.tif]

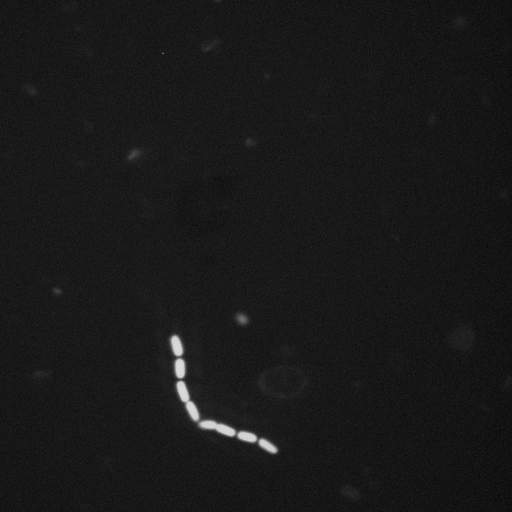

Supplement: S2 File — (ZIP) [file pcbi.1006986.s003.zip › extrait_4hKM16021/4h-Z694_26_w2sdcGFP.tif]

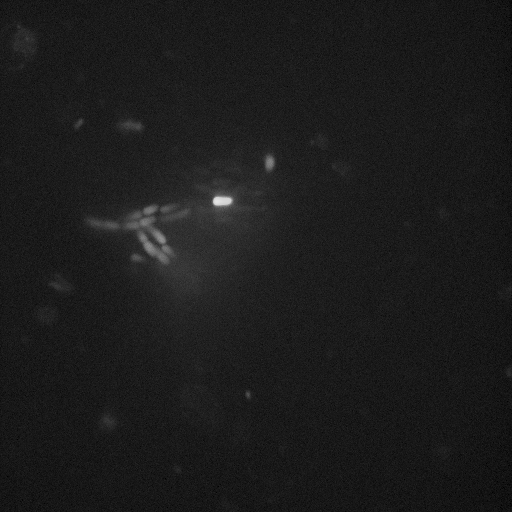

Supplement: S2 File — (ZIP) [file pcbi.1006986.s003.zip › extrait_4hKM16021/4h-Z693_12_w1sdcRFP.tif]

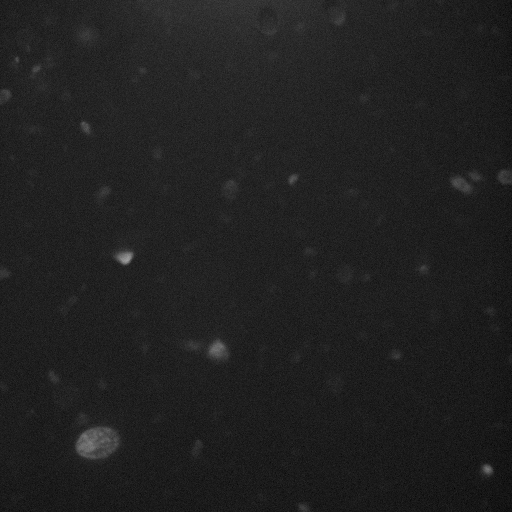

Supplement: S2 File — (ZIP) [file pcbi.1006986.s003.zip › extrait_4hKM16021/4h-Z693_6_w1sdcRFP.tif]

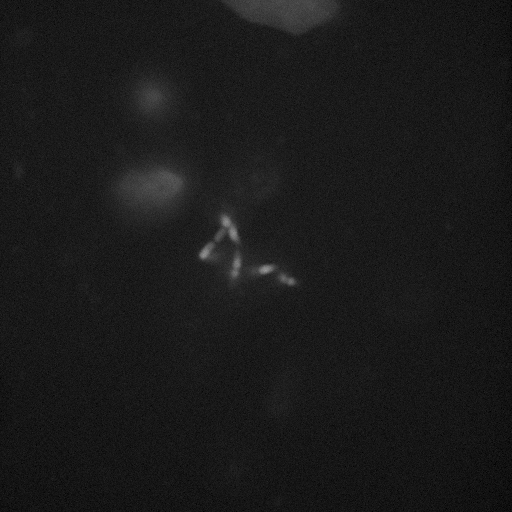

Supplement: S2 File — (ZIP) [file pcbi.1006986.s003.zip › extrait_4hKM16021/4h-Z692_11_w1sdcRFP.tif]

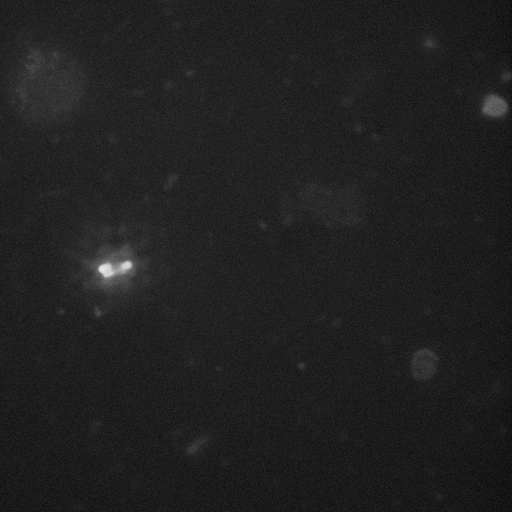

Supplement: S2 File — (ZIP) [file pcbi.1006986.s003.zip › extrait_4hKM16021/4h-Z693_17_w1sdcRFP.tif]

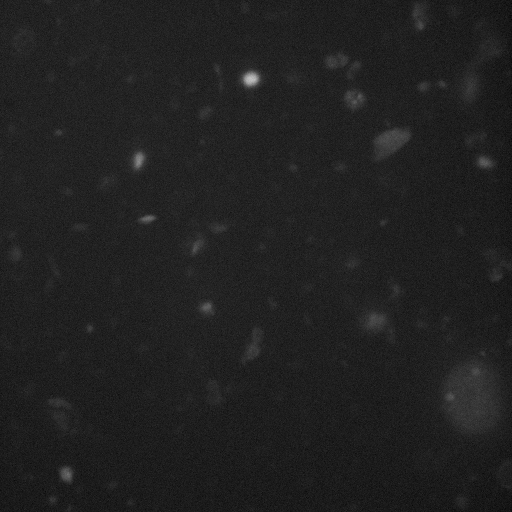

Supplement: S2 File — (ZIP) [file pcbi.1006986.s003.zip › extrait_4hKM16021/4h-Z693_21_w1sdcRFP.tif]

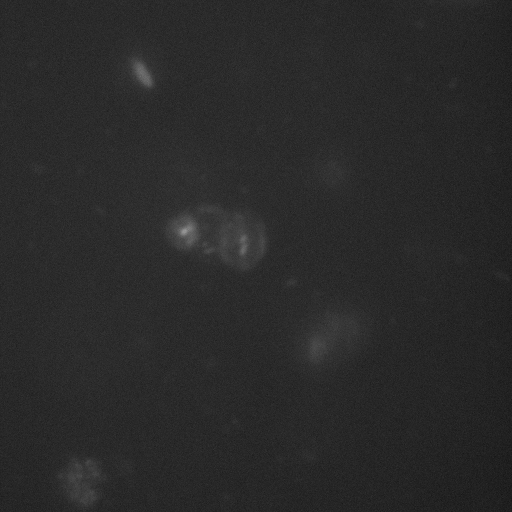

Supplement: S2 File — (ZIP) [file pcbi.1006986.s003.zip › extrait_4hKM16021/4h-Z692_5_w2sdcGFP.tif]

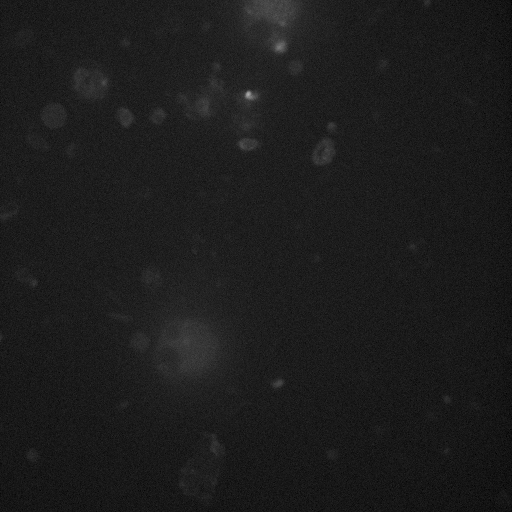

Supplement: S2 File — (ZIP) [file pcbi.1006986.s003.zip › extrait_4hKM16021/4h-Z694_21_w1sdcRFP.tif]

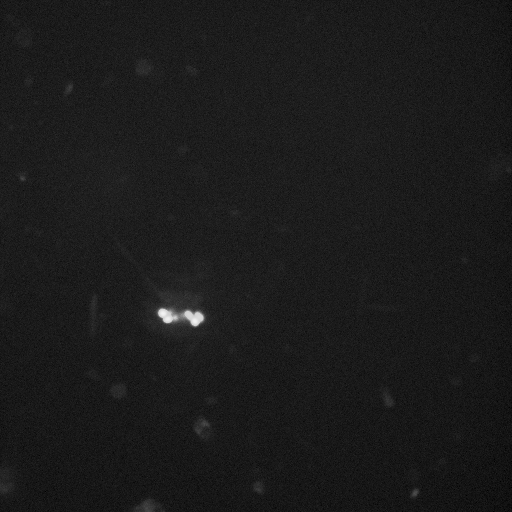

Supplement: S2 File — (ZIP) [file pcbi.1006986.s003.zip › extrait_4hKM16021/4h-Z693_8_w1sdcRFP.tif]

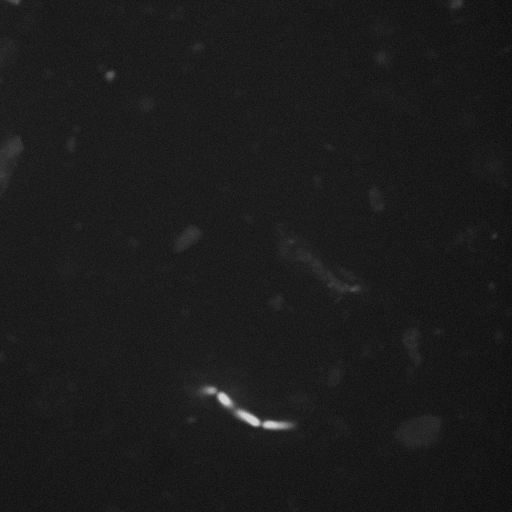

Supplement: S2 File — (ZIP) [file pcbi.1006986.s003.zip › extrait_4hKM16021/4h-Z692_32_w2sdcGFP.tif]

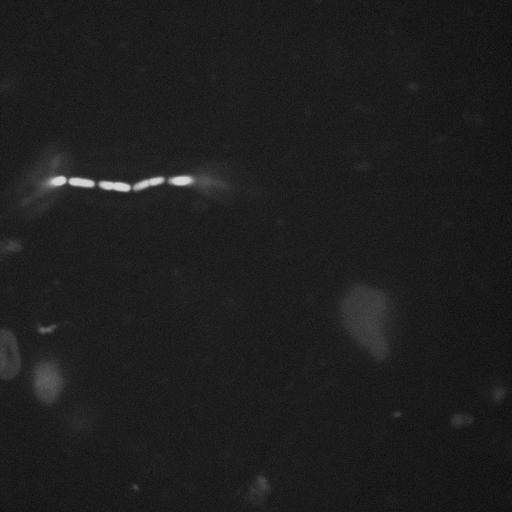

Supplement: S2 File — (ZIP) [file pcbi.1006986.s003.zip › extrait_4hKM16021/4h-Z692_18_w2sdcGFP.tif]

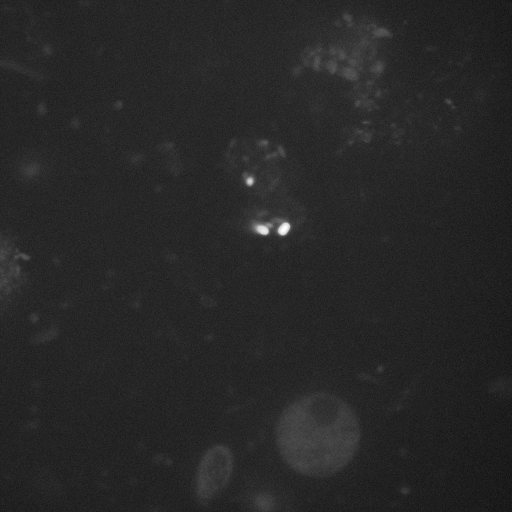

Supplement: S2 File — (ZIP) [file pcbi.1006986.s003.zip › extrait_4hKM16021/4h-Z692_10_w2sdcGFP.tif]

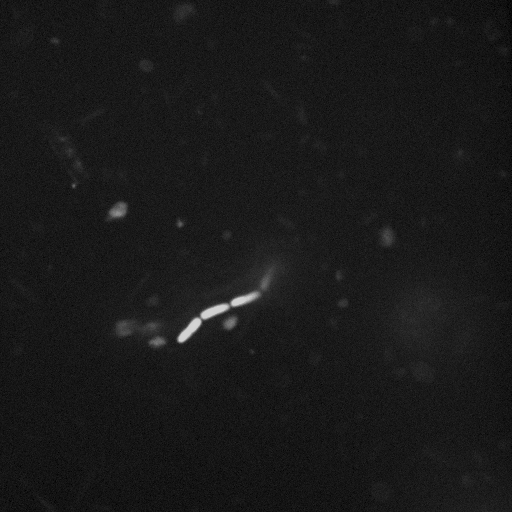

Supplement: S2 File — (ZIP) [file pcbi.1006986.s003.zip › extrait_4hKM16021/4h-Z692_28_w1sdcRFP.tif]

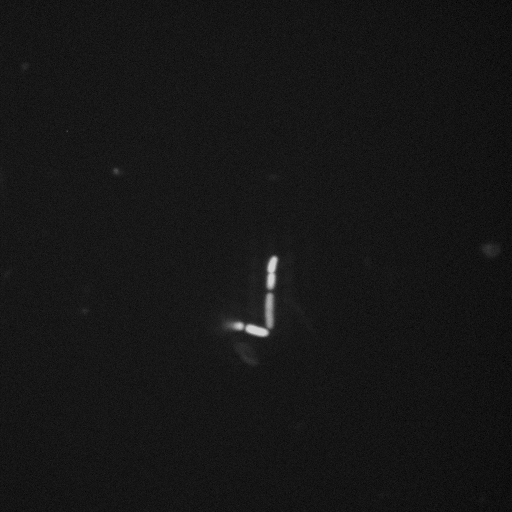

Supplement: S2 File — (ZIP) [file pcbi.1006986.s003.zip › extrait_4hKM16021/4h-Z694_4_w2sdcGFP.tif]

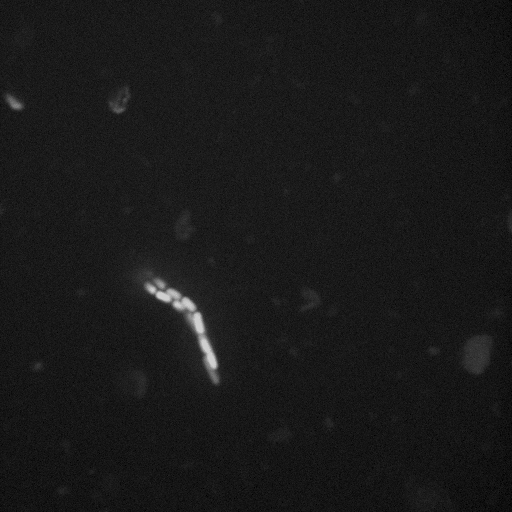

Supplement: S2 File — (ZIP) [file pcbi.1006986.s003.zip › extrait_4hKM16021/4h-Z692_25_w1sdcRFP.tif]

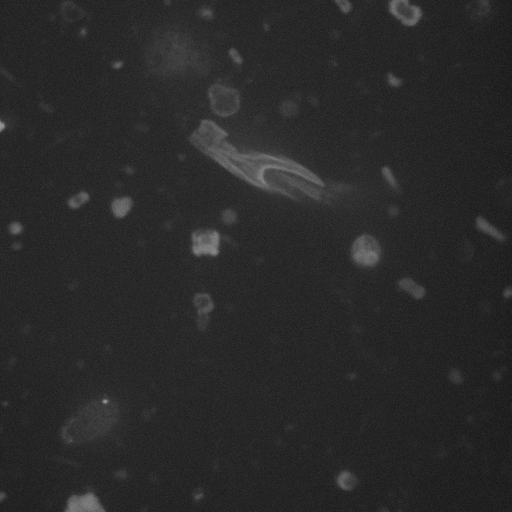

Supplement: S2 File — (ZIP) [file pcbi.1006986.s003.zip › extrait_4hKM16021/4h-Z693_11_w2sdcGFP.tif]

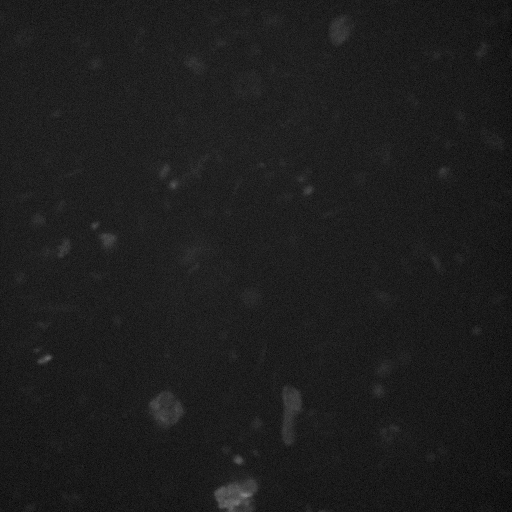

Supplement: S2 File — (ZIP) [file pcbi.1006986.s003.zip › extrait_4hKM16021/4h-Z692_21_w1sdcRFP.tif]

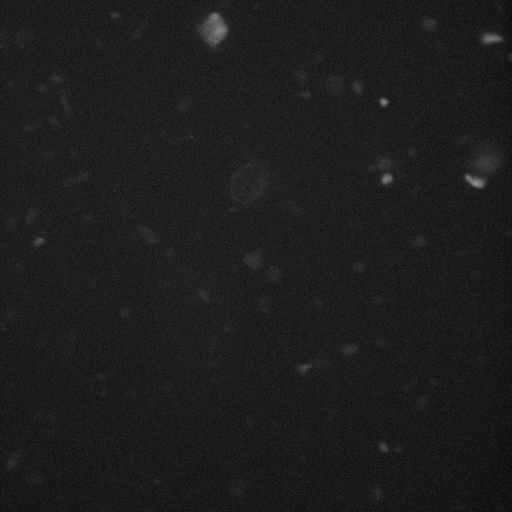

Supplement: S2 File — (ZIP) [file pcbi.1006986.s003.zip › extrait_4hKM16021/4h-Z692_22_w1sdcRFP.tif]

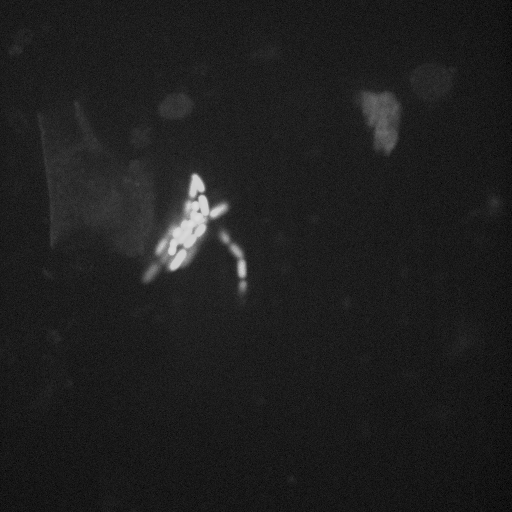

Supplement: S2 File — (ZIP) [file pcbi.1006986.s003.zip › extrait_4hKM16021/4h-Z693_9_w1sdcRFP.tif]

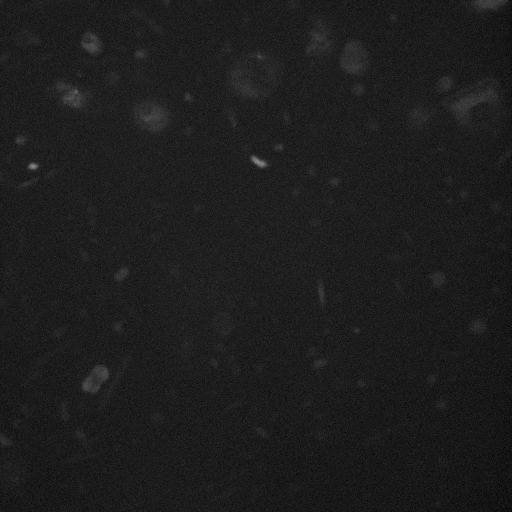

Supplement: S2 File — (ZIP) [file pcbi.1006986.s003.zip › extrait_4hKM16021/4h-Z692_33_w1sdcRFP.tif]

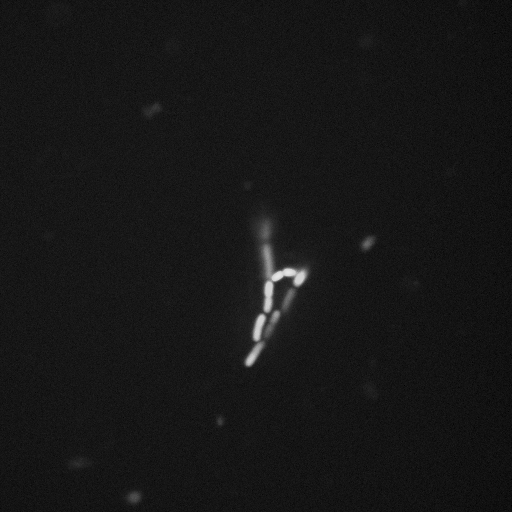

Supplement: S2 File — (ZIP) [file pcbi.1006986.s003.zip › extrait_4hKM16021/4h-Z694_15_w2sdcGFP.tif]

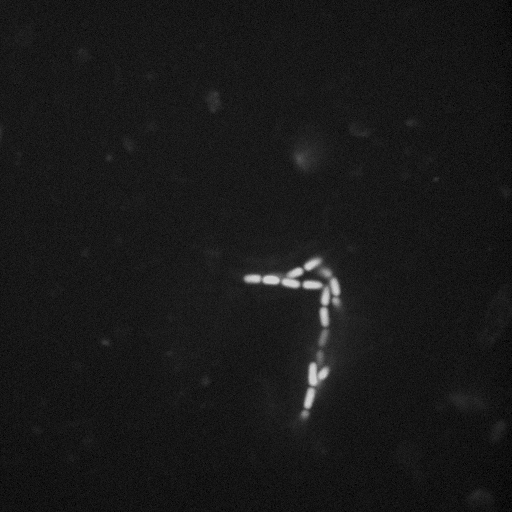

Supplement: S2 File — (ZIP) [file pcbi.1006986.s003.zip › extrait_4hKM16021/4h-Z692_30_w1sdcRFP.tif]

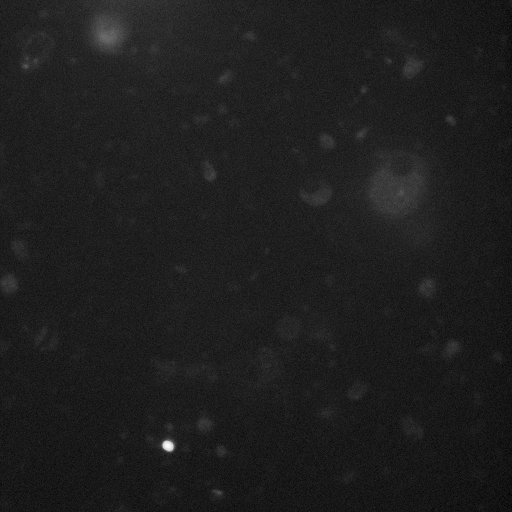

Supplement: S2 File — (ZIP) [file pcbi.1006986.s003.zip › extrait_4hKM16021/4h-Z693_24_w1sdcRFP.tif]

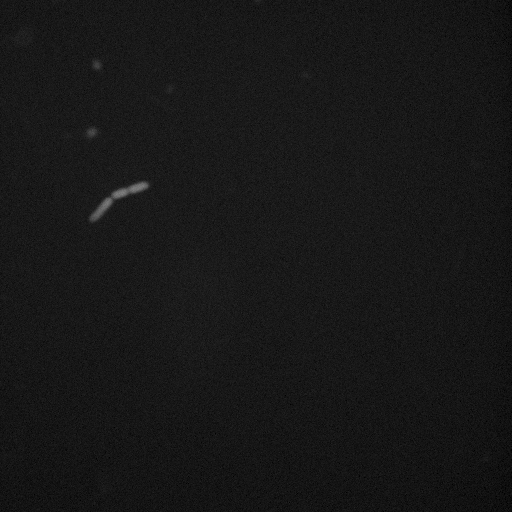

Supplement: S2 File — (ZIP) [file pcbi.1006986.s003.zip › extrait_4hKM16021/4h-Z694_5_w1sdcRFP.tif]

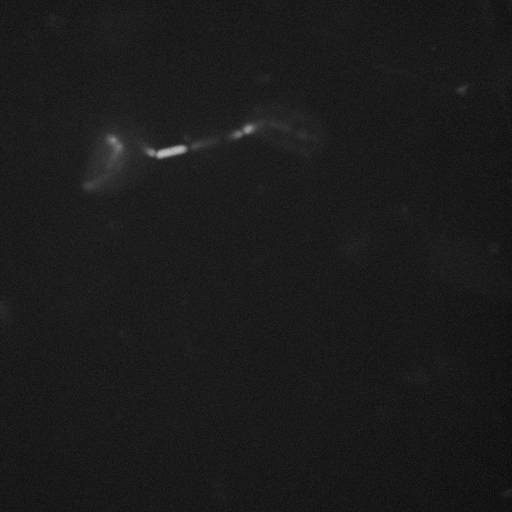

Supplement: S2 File — (ZIP) [file pcbi.1006986.s003.zip › extrait_4hKM16021/4h-Z692_7_w2sdcGFP.tif]

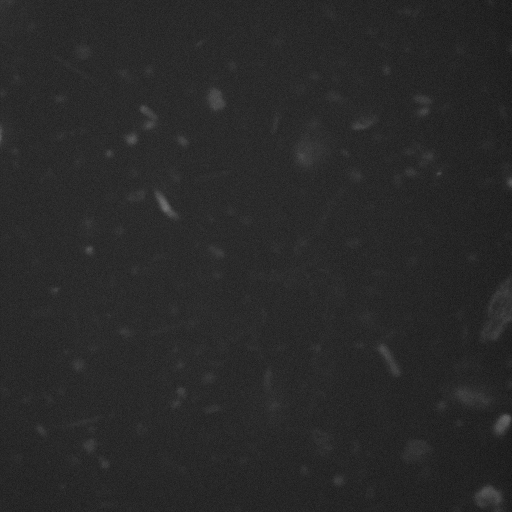

Supplement: S2 File — (ZIP) [file pcbi.1006986.s003.zip › extrait_4hKM16021/4h-Z692_30_w2sdcGFP.tif]

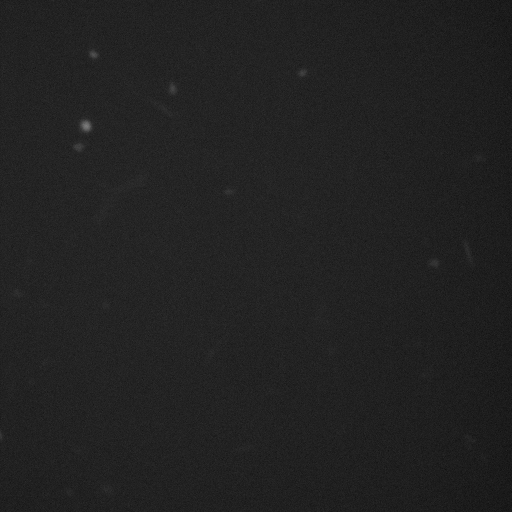

Supplement: S2 File — (ZIP) [file pcbi.1006986.s003.zip › extrait_4hKM16021/4h-Z694_5_w2sdcGFP.tif]

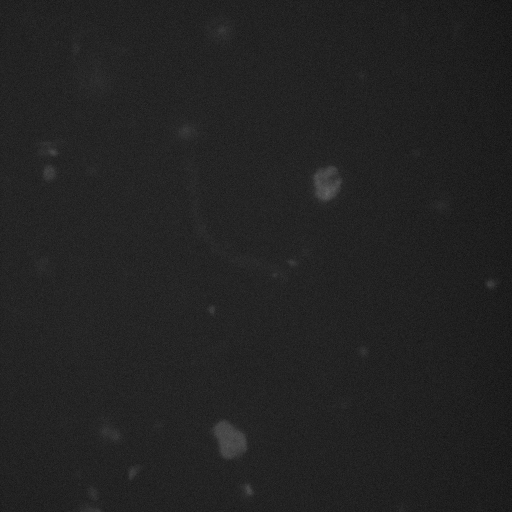

Supplement: S2 File — (ZIP) [file pcbi.1006986.s003.zip › extrait_4hKM16021/4h-Z694_6_w2sdcGFP.tif]

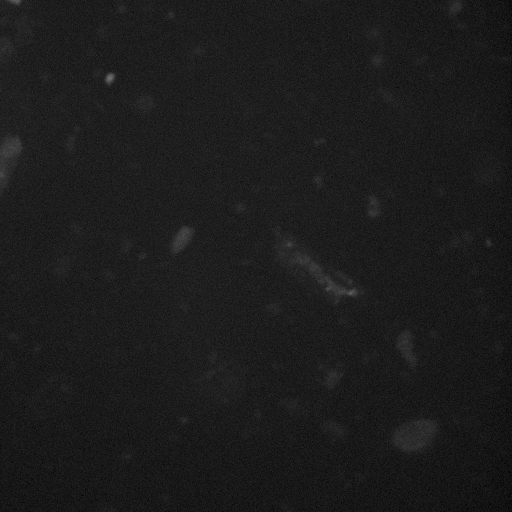

Supplement: S2 File — (ZIP) [file pcbi.1006986.s003.zip › extrait_4hKM16021/4h-Z692_32_w1sdcRFP.tif]

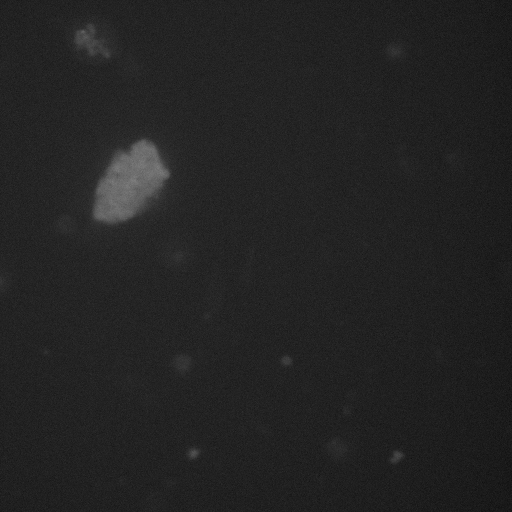

Supplement: S2 File — (ZIP) [file pcbi.1006986.s003.zip › extrait_4hKM16021/4h-Z694_7_w2sdcGFP.tif]

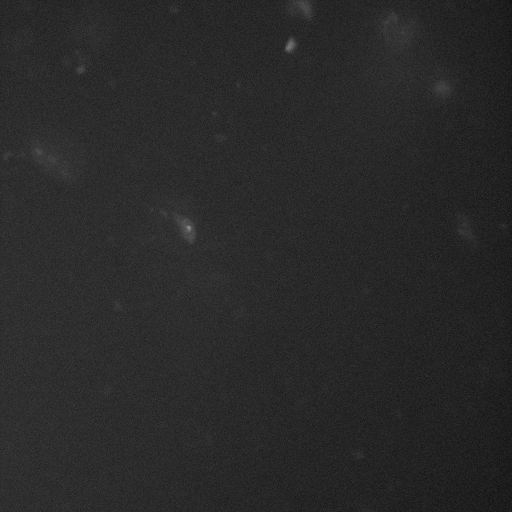

Supplement: S2 File — (ZIP) [file pcbi.1006986.s003.zip › extrait_4hKM16021/4h-Z693_15_w1sdcRFP.tif]

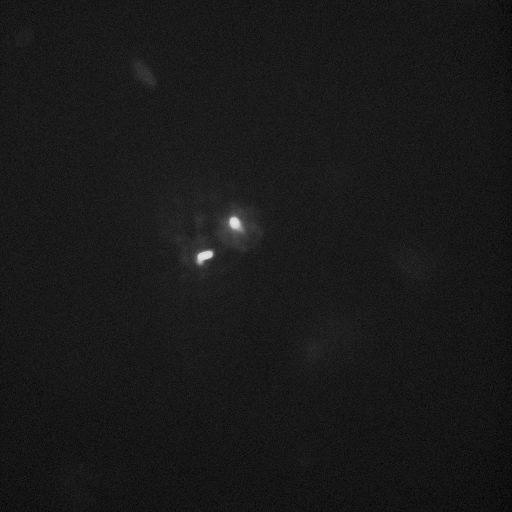

Supplement: S2 File — (ZIP) [file pcbi.1006986.s003.zip › extrait_4hKM16021/4h-Z692_5_w1sdcRFP.tif]

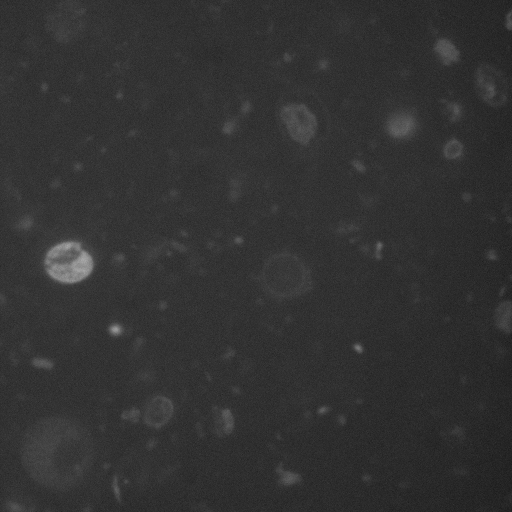

Supplement: S2 File — (ZIP) [file pcbi.1006986.s003.zip › extrait_4hKM16021/4h-Z693_28_w2sdcGFP.tif]

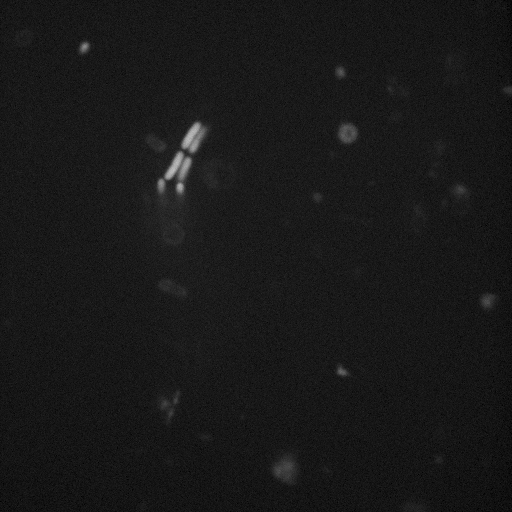

Supplement: S2 File — (ZIP) [file pcbi.1006986.s003.zip › extrait_4hKM16021/4h-Z694_18_w1sdcRFP.tif]

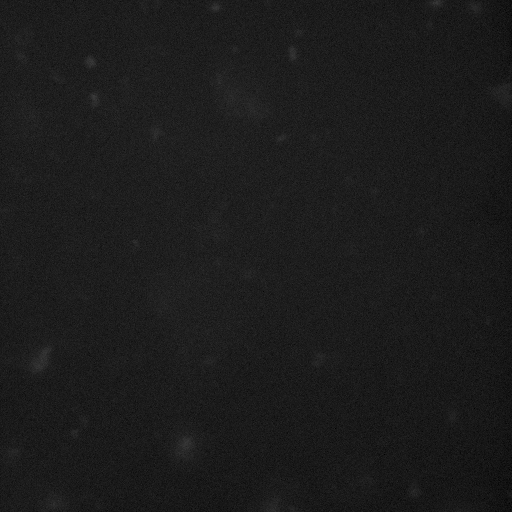

Supplement: S2 File — (ZIP) [file pcbi.1006986.s003.zip › extrait_4hKM16021/4h-Z692_31_w1sdcRFP.tif]

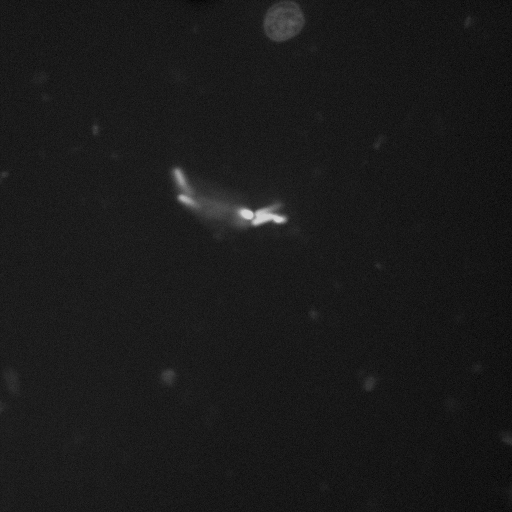

Supplement: S2 File — (ZIP) [file pcbi.1006986.s003.zip › extrait_4hKM16021/4h-Z694_19_w2sdcGFP.tif]

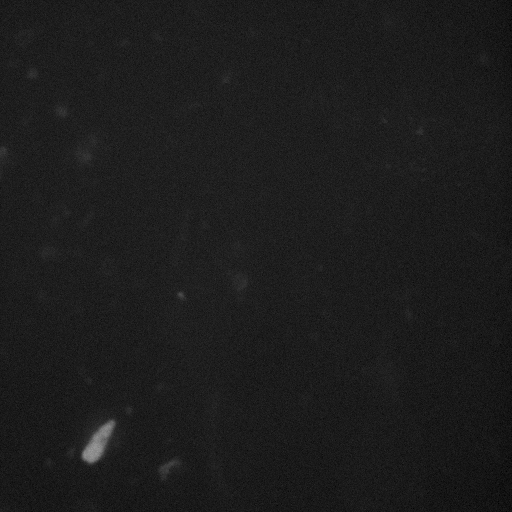

Supplement: S2 File — (ZIP) [file pcbi.1006986.s003.zip › extrait_4hKM16021/4h-Z692_19_w1sdcRFP.tif]

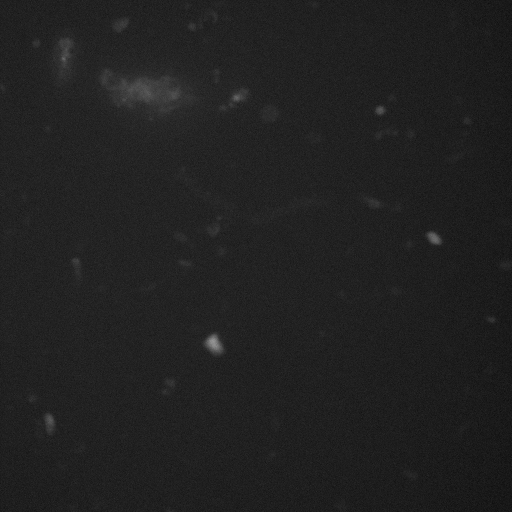

Supplement: S2 File — (ZIP) [file pcbi.1006986.s003.zip › extrait_4hKM16021/4h-Z694_28_w2sdcGFP.tif]

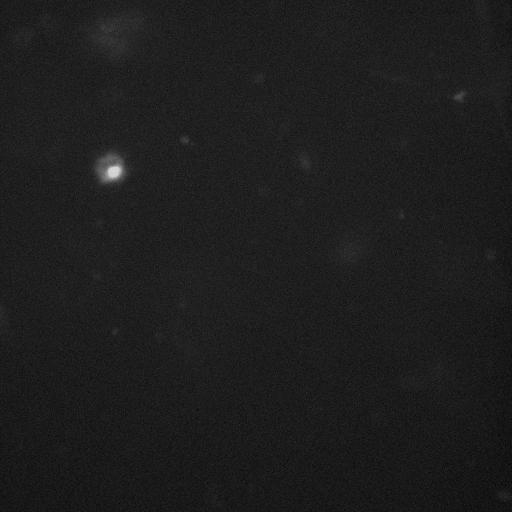

Supplement: S2 File — (ZIP) [file pcbi.1006986.s003.zip › extrait_4hKM16021/4h-Z692_7_w1sdcRFP.tif]

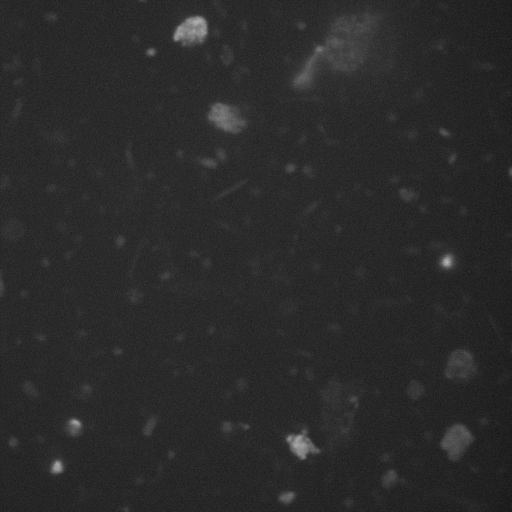

Supplement: S2 File — (ZIP) [file pcbi.1006986.s003.zip › extrait_4hKM16021/4h-Z692_27_w2sdcGFP.tif]

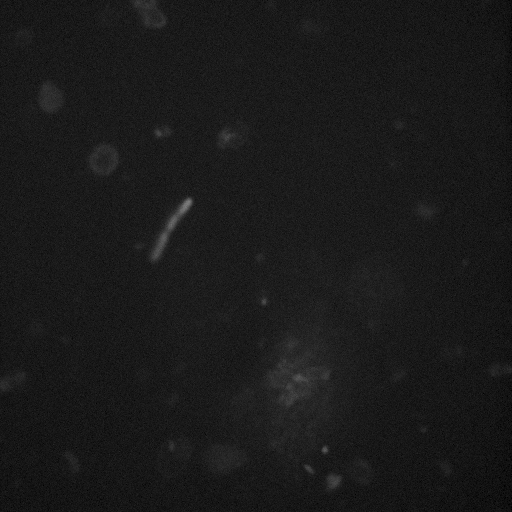

Supplement: S2 File — (ZIP) [file pcbi.1006986.s003.zip › extrait_4hKM16021/4h-Z693_5_w1sdcRFP.tif]

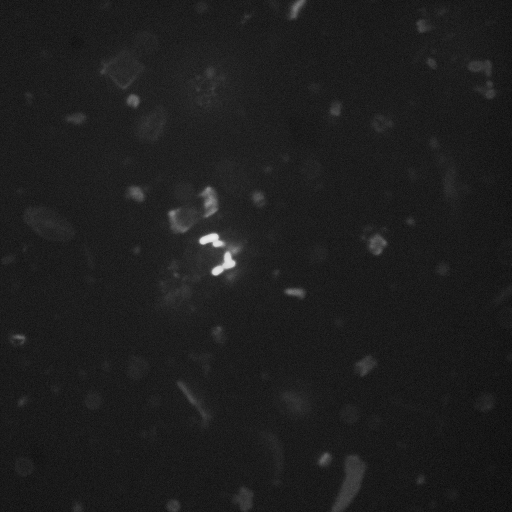

Supplement: S2 File — (ZIP) [file pcbi.1006986.s003.zip › extrait_4hKM16021/4h-Z695_2_w2sdcGFP.tif]

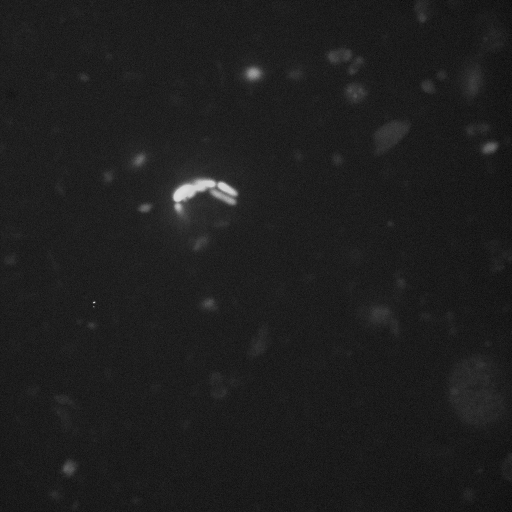

Supplement: S2 File — (ZIP) [file pcbi.1006986.s003.zip › extrait_4hKM16021/4h-Z693_21_w2sdcGFP.tif]

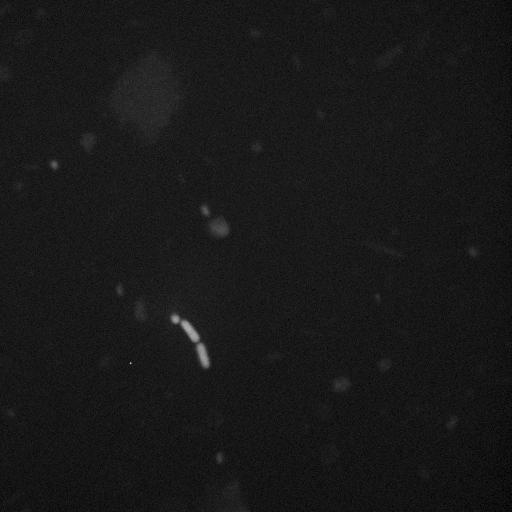

Supplement: S2 File — (ZIP) [file pcbi.1006986.s003.zip › extrait_4hKM16021/4h-Z692_12_w1sdcRFP.tif]

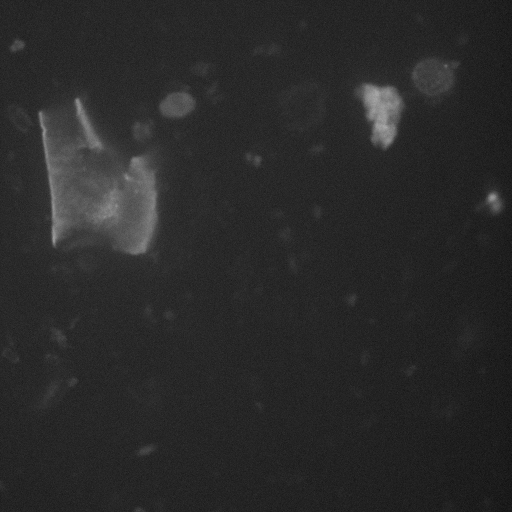

Supplement: S2 File — (ZIP) [file pcbi.1006986.s003.zip › extrait_4hKM16021/4h-Z693_9_w2sdcGFP.tif]

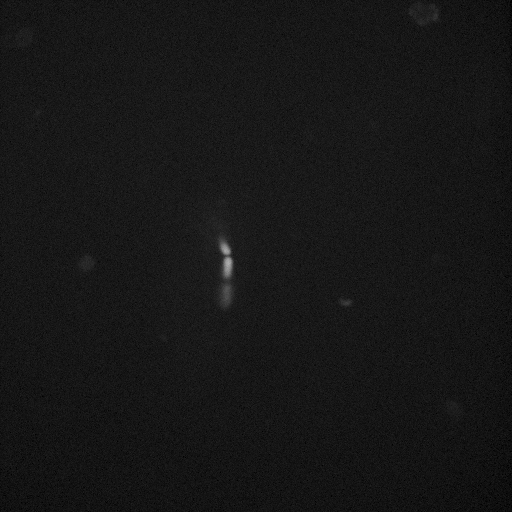

Supplement: S2 File — (ZIP) [file pcbi.1006986.s003.zip › extrait_4hKM16021/4h-Z694_14_w1sdcRFP.tif]

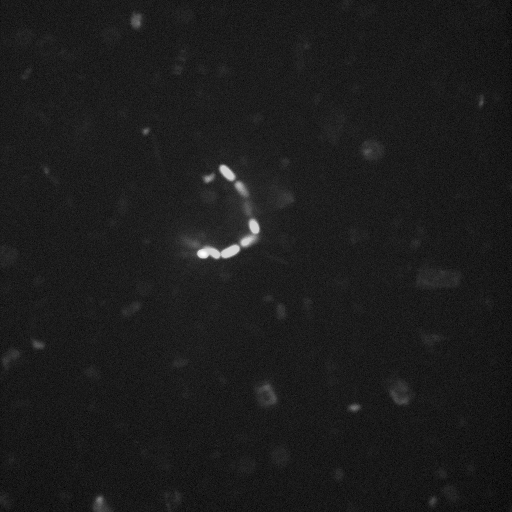

Supplement: S2 File — (ZIP) [file pcbi.1006986.s003.zip › extrait_4hKM16021/4h-Z693_29_w1sdcRFP.tif]

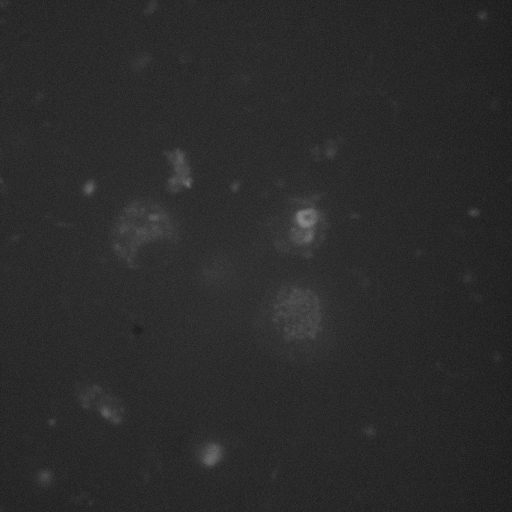

Supplement: S2 File — (ZIP) [file pcbi.1006986.s003.zip › extrait_4hKM16021/4h-Z693_2_w2sdcGFP.tif]

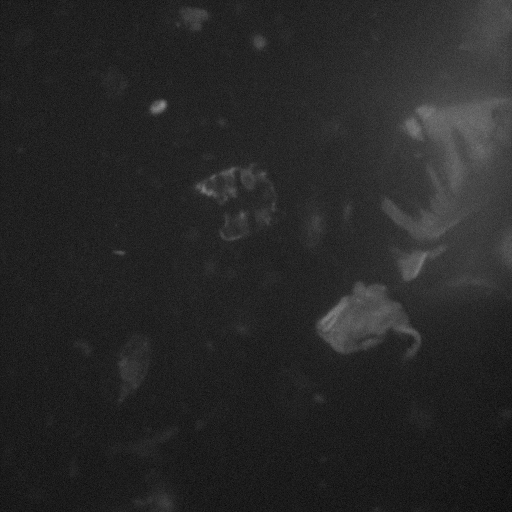

Supplement: S2 File — (ZIP) [file pcbi.1006986.s003.zip › extrait_4hKM16021/4h-Z692_9_w1sdcRFP.tif]

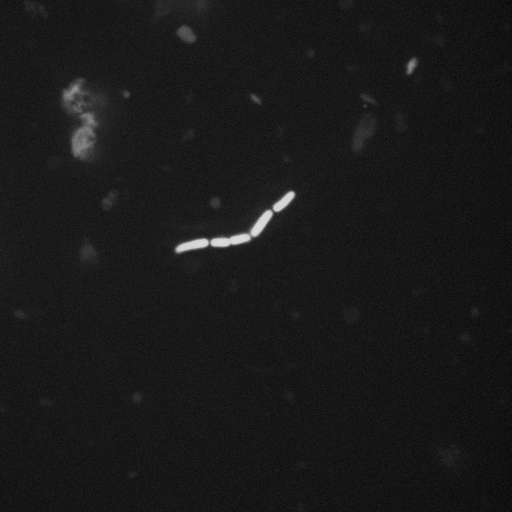

Supplement: S2 File — (ZIP) [file pcbi.1006986.s003.zip › extrait_4hKM16021/4h-Z694_17_w2sdcGFP.tif]

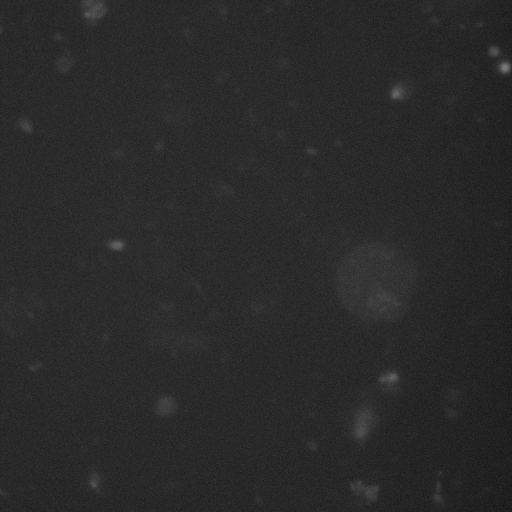

Supplement: S2 File — (ZIP) [file pcbi.1006986.s003.zip › extrait_4hKM16021/4h-Z692_15_w2sdcGFP.tif]

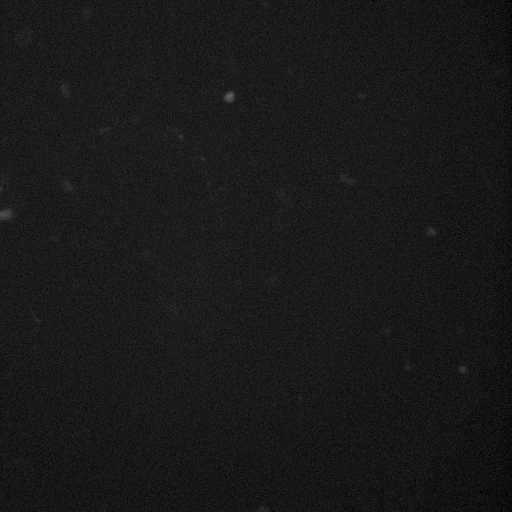

Supplement: S2 File — (ZIP) [file pcbi.1006986.s003.zip › extrait_4hKM16021/4h-Z694_27_w1sdcRFP.tif]

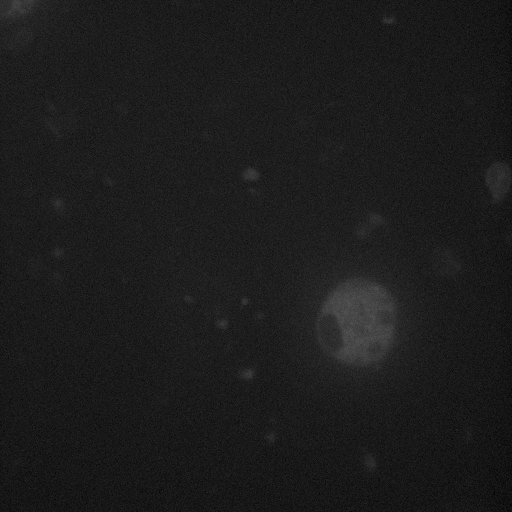

Supplement: S2 File — (ZIP) [file pcbi.1006986.s003.zip › extrait_4hKM16021/4h-Z694_25_w1sdcRFP.tif]

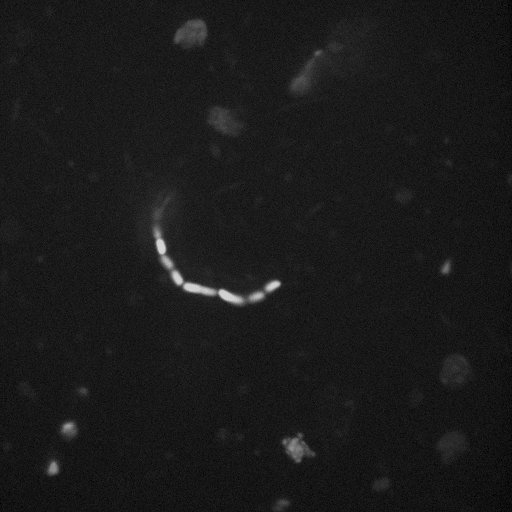

Supplement: S2 File — (ZIP) [file pcbi.1006986.s003.zip › extrait_4hKM16021/4h-Z692_27_w1sdcRFP.tif]

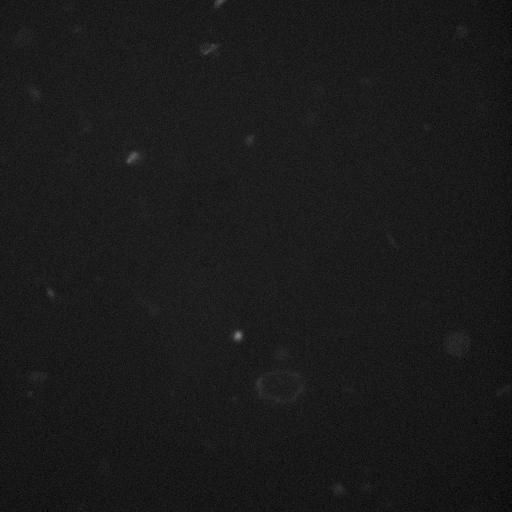

Supplement: S2 File — (ZIP) [file pcbi.1006986.s003.zip › extrait_4hKM16021/4h-Z694_26_w1sdcRFP.tif]

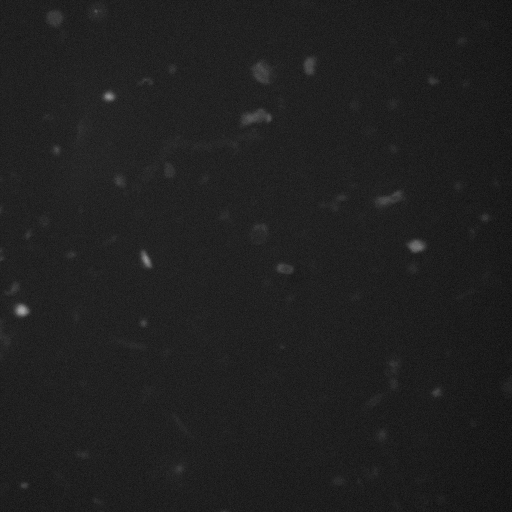

Supplement: S2 File — (ZIP) [file pcbi.1006986.s003.zip › extrait_4hKM16021/4h-Z694_30_w2sdcGFP.tif]

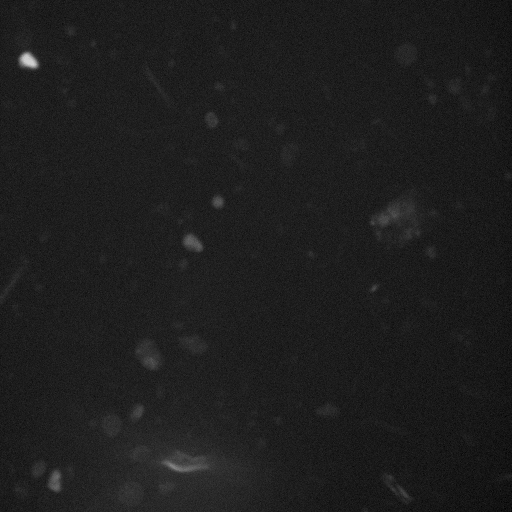

Supplement: S2 File — (ZIP) [file pcbi.1006986.s003.zip › extrait_4hKM16021/4h-Z692_17_w1sdcRFP.tif]

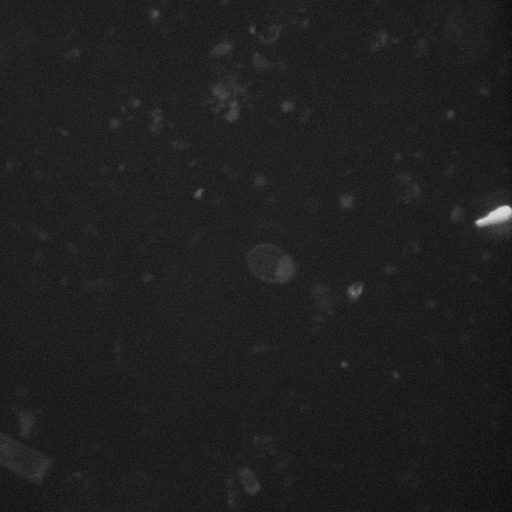

Supplement: S2 File — (ZIP) [file pcbi.1006986.s003.zip › extrait_4hKM16021/4h-Z693_19_w1sdcRFP.tif]

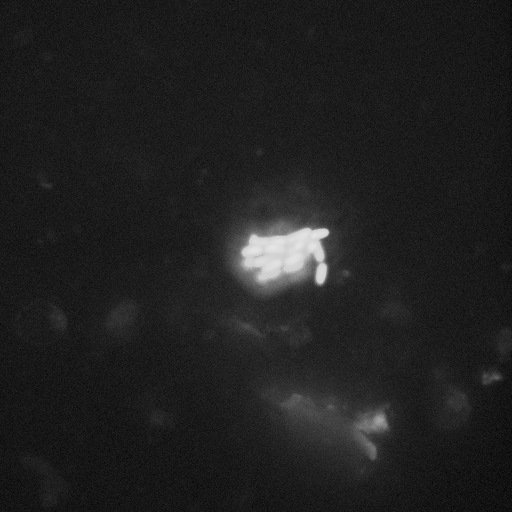

Supplement: S2 File — (ZIP) [file pcbi.1006986.s003.zip › extrait_4hKM16021/4h-Z693_25_w1sdcRFP.tif]

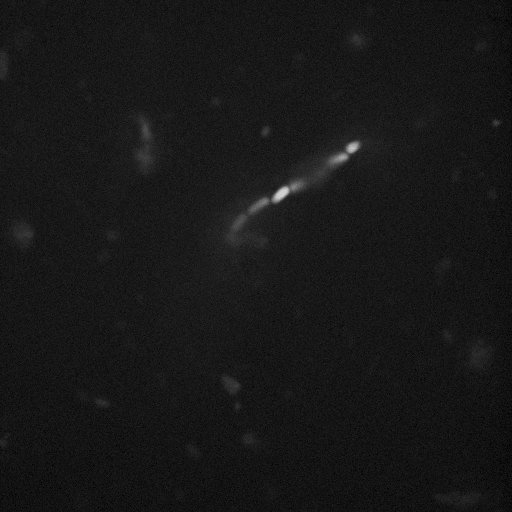

Supplement: S2 File — (ZIP) [file pcbi.1006986.s003.zip › extrait_4hKM16021/4h-Z692_26_w1sdcRFP.tif]

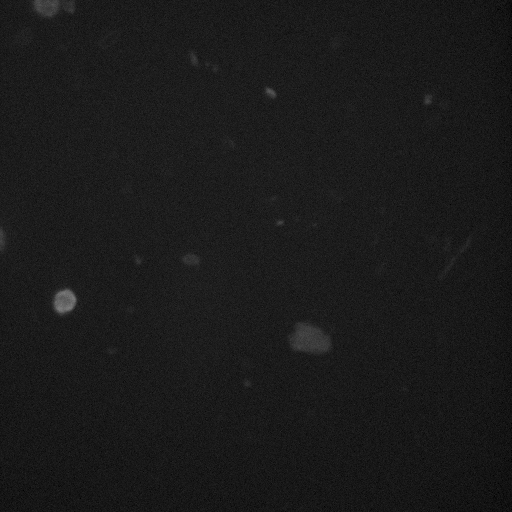

Supplement: S2 File — (ZIP) [file pcbi.1006986.s003.zip › extrait_4hKM16021/4h-Z694_11_w1sdcRFP.tif]

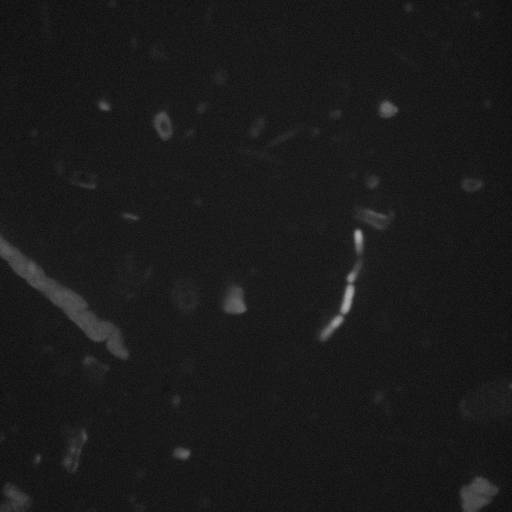

Supplement: S2 File — (ZIP) [file pcbi.1006986.s003.zip › extrait_4hKM16021/4h-Z693_13_w2sdcGFP.tif]

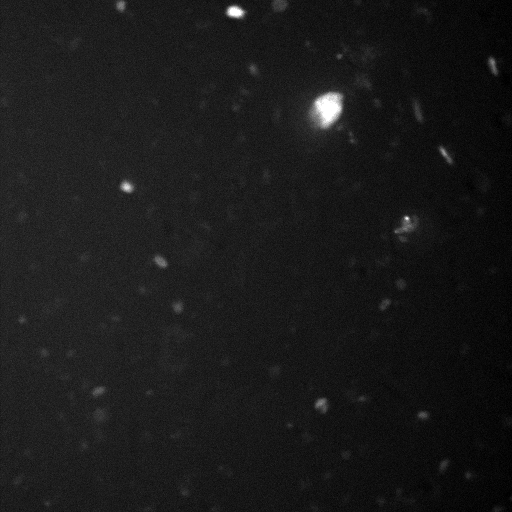

Supplement: S2 File — (ZIP) [file pcbi.1006986.s003.zip › extrait_4hKM16021/4h-Z693_14_w2sdcGFP.tif]

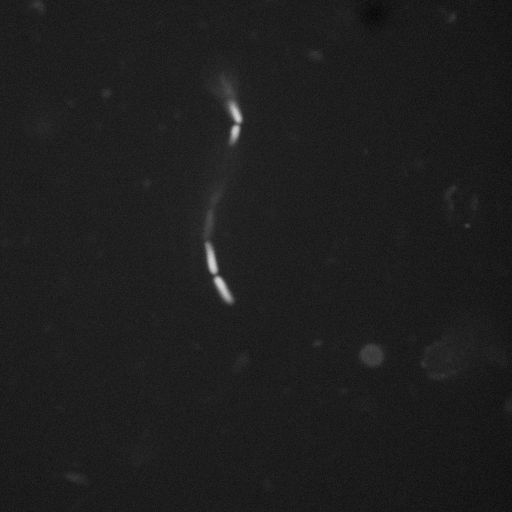

Supplement: S2 File — (ZIP) [file pcbi.1006986.s003.zip › extrait_4hKM16021/4h-Z692_8_w2sdcGFP.tif]

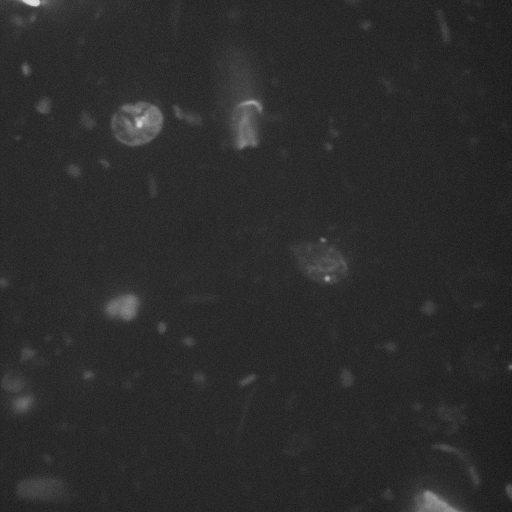

Supplement: S2 File — (ZIP) [file pcbi.1006986.s003.zip › extrait_4hKM16021/4h-Z692_24_w2sdcGFP.tif]

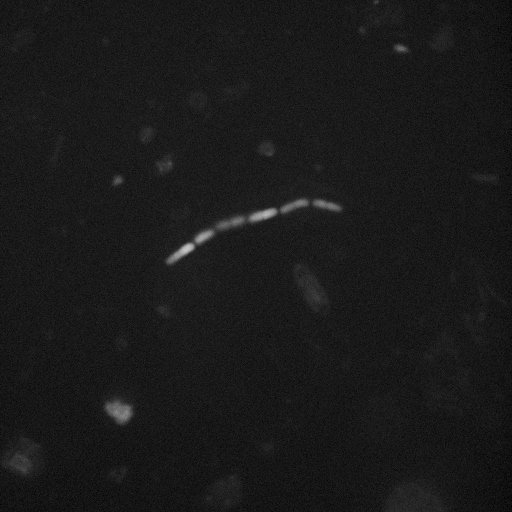

Supplement: S2 File — (ZIP) [file pcbi.1006986.s003.zip › extrait_4hKM16021/4h-Z692_13_w1sdcRFP.tif]

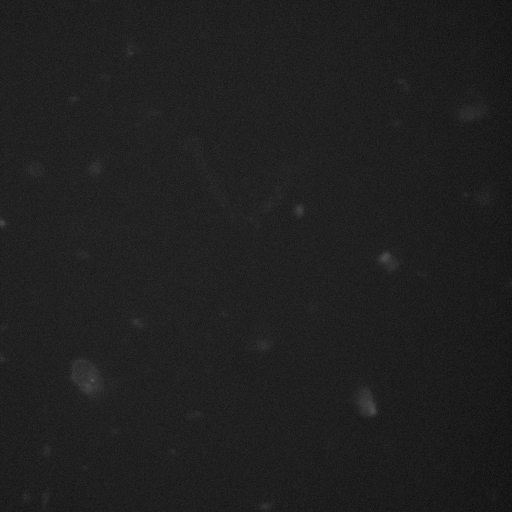

Supplement: S2 File — (ZIP) [file pcbi.1006986.s003.zip › extrait_4hKM16021/4h-Z694_8_w2sdcGFP.tif]

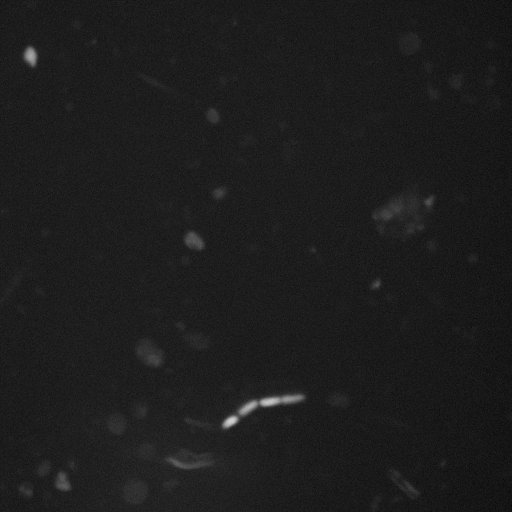

Supplement: S2 File — (ZIP) [file pcbi.1006986.s003.zip › extrait_4hKM16021/4h-Z692_17_w2sdcGFP.tif]

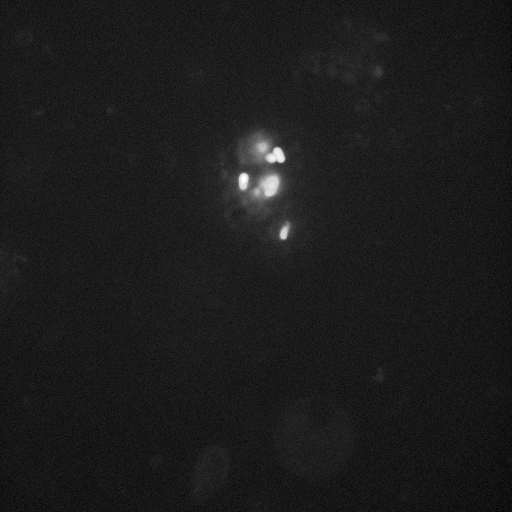

Supplement: S2 File — (ZIP) [file pcbi.1006986.s003.zip › extrait_4hKM16021/4h-Z692_10_w1sdcRFP.tif]

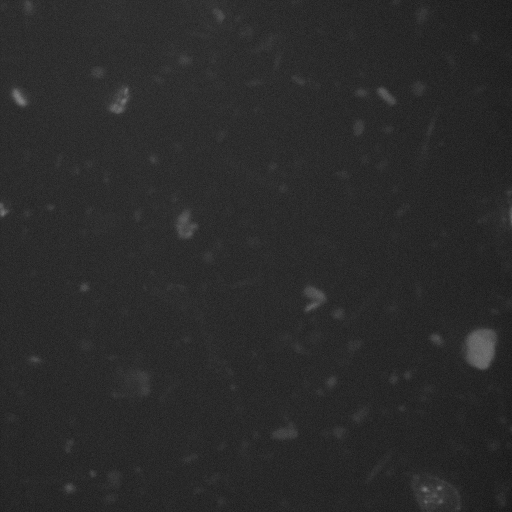

Supplement: S2 File — (ZIP) [file pcbi.1006986.s003.zip › extrait_4hKM16021/4h-Z692_25_w2sdcGFP.tif]

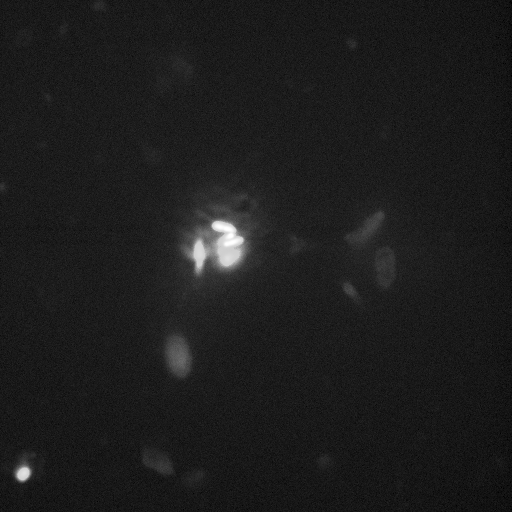

Supplement: S2 File — (ZIP) [file pcbi.1006986.s003.zip › extrait_4hKM16021/4h-Z692_16_w1sdcRFP.tif]

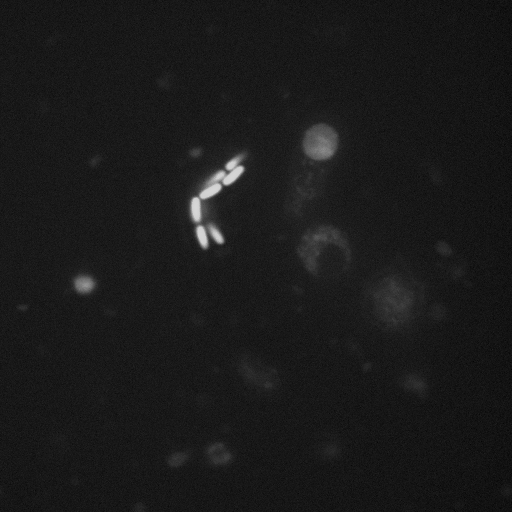

Supplement: S2 File — (ZIP) [file pcbi.1006986.s003.zip › extrait_4hKM16021/4h-Z694_20_w2sdcGFP.tif]

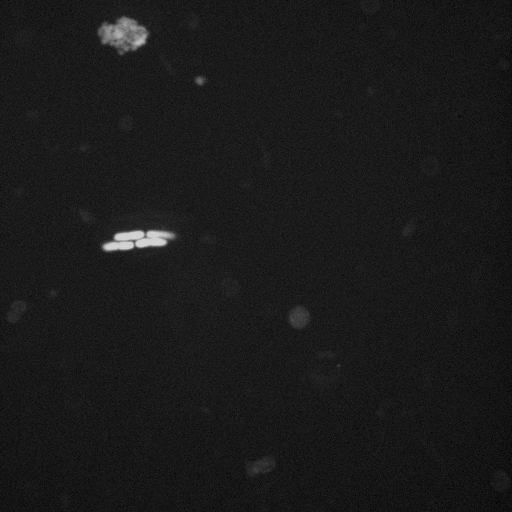

Supplement: S2 File — (ZIP) [file pcbi.1006986.s003.zip › extrait_4hKM16021/4h-Z693_4_w1sdcRFP.tif]

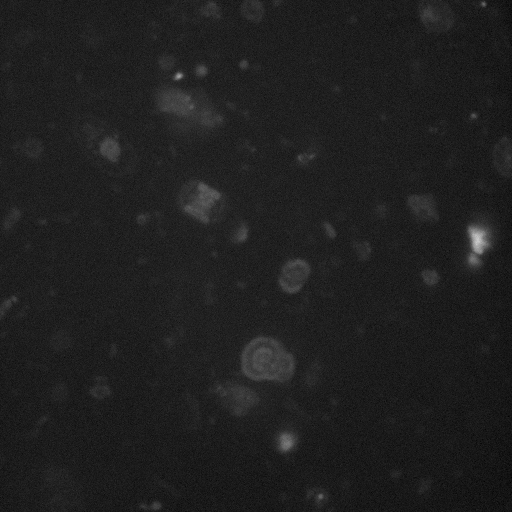

Supplement: S2 File — (ZIP) [file pcbi.1006986.s003.zip › extrait_4hKM16021/4h-Z693_27_w1sdcRFP.tif]

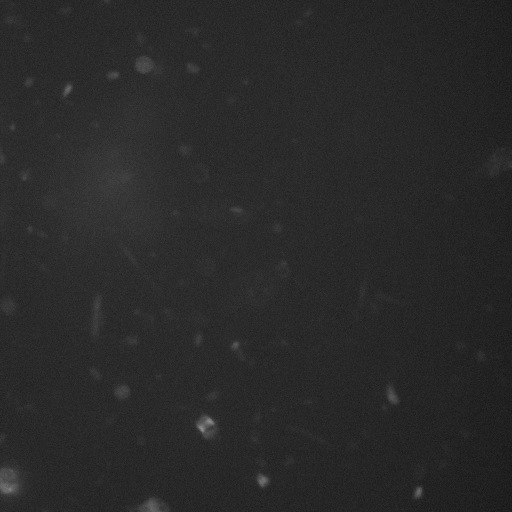

Supplement: S2 File — (ZIP) [file pcbi.1006986.s003.zip › extrait_4hKM16021/4h-Z693_8_w2sdcGFP.tif]

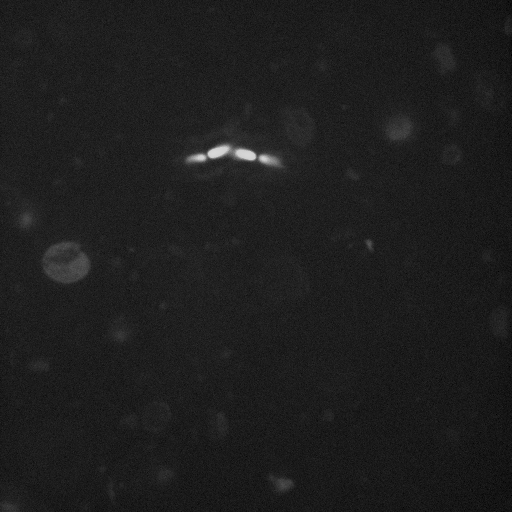

Supplement: S2 File — (ZIP) [file pcbi.1006986.s003.zip › extrait_4hKM16021/4h-Z693_28_w1sdcRFP.tif]

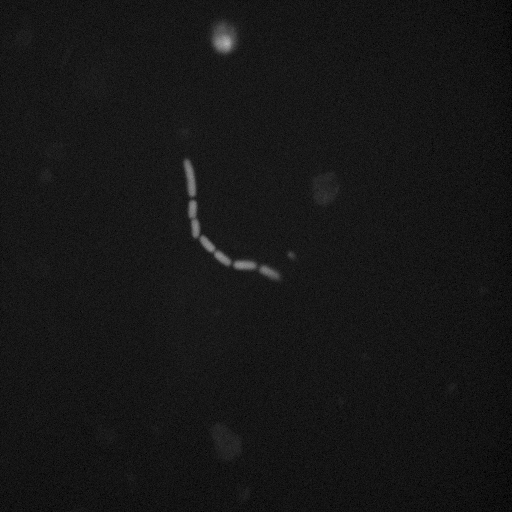

Supplement: S2 File — (ZIP) [file pcbi.1006986.s003.zip › extrait_4hKM16021/4h-Z694_6_w1sdcRFP.tif]

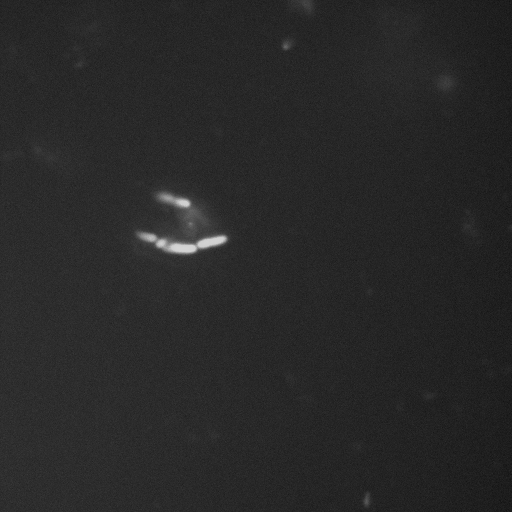

Supplement: S2 File — (ZIP) [file pcbi.1006986.s003.zip › extrait_4hKM16021/4h-Z693_15_w2sdcGFP.tif]

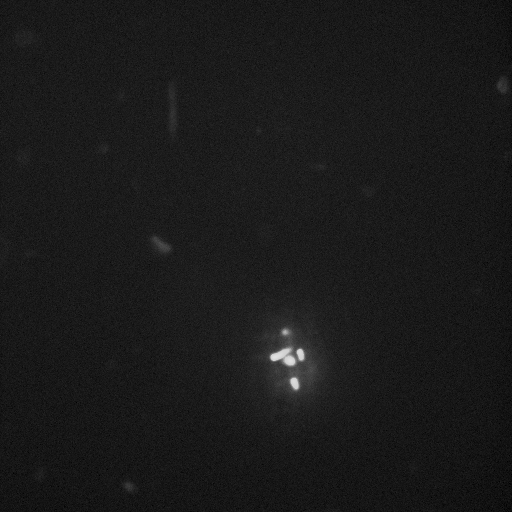

Supplement: S2 File — (ZIP) [file pcbi.1006986.s003.zip › extrait_4hKM16021/4h-Z692_1_w1sdcRFP.tif]

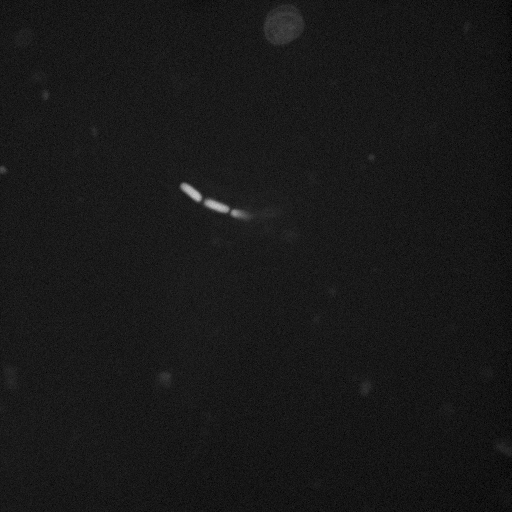

Supplement: S2 File — (ZIP) [file pcbi.1006986.s003.zip › extrait_4hKM16021/4h-Z694_19_w1sdcRFP.tif]

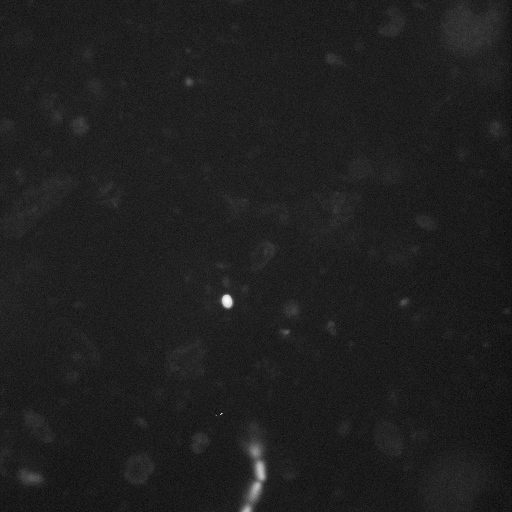

Supplement: S2 File — (ZIP) [file pcbi.1006986.s003.zip › extrait_4hKM16021/4h-Z693_30_w1sdcRFP.tif]

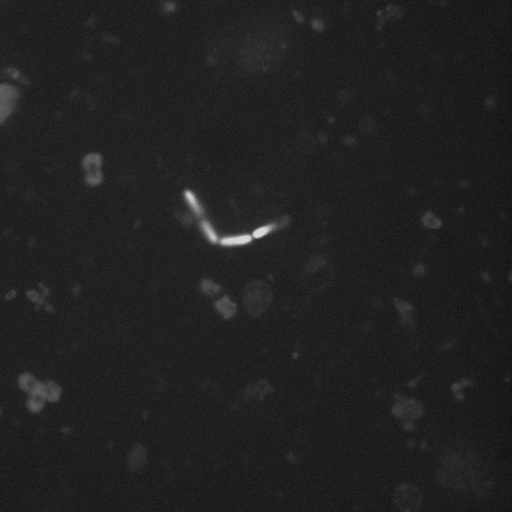

Supplement: S2 File — (ZIP) [file pcbi.1006986.s003.zip › extrait_4hKM16021/4h-Z693_31_w2sdcGFP.tif]

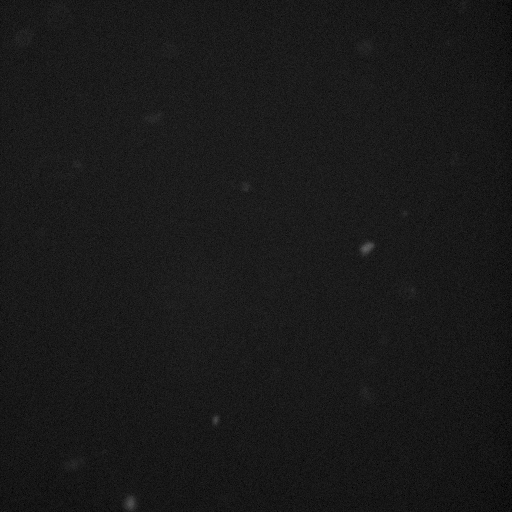

Supplement: S2 File — (ZIP) [file pcbi.1006986.s003.zip › extrait_4hKM16021/4h-Z694_15_w1sdcRFP.tif]

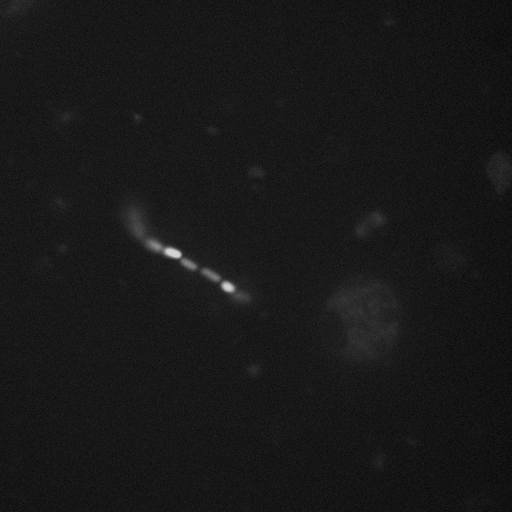

Supplement: S2 File — (ZIP) [file pcbi.1006986.s003.zip › extrait_4hKM16021/4h-Z694_25_w2sdcGFP.tif]

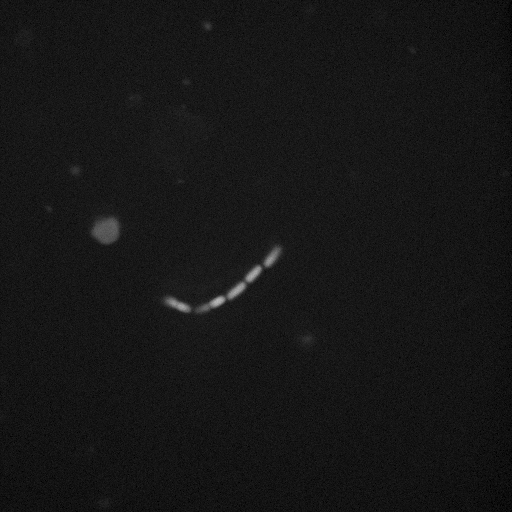

Supplement: S2 File — (ZIP) [file pcbi.1006986.s003.zip › extrait_4hKM16021/4h-Z694_13_w1sdcRFP.tif]

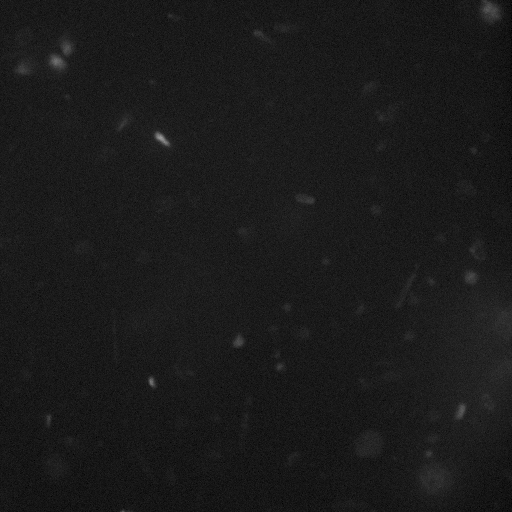

Supplement: S2 File — (ZIP) [file pcbi.1006986.s003.zip › extrait_4hKM16021/4h-Z695_3_w1sdcRFP.tif]

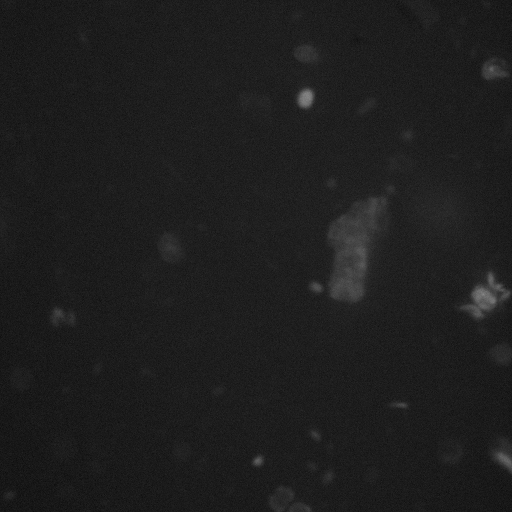

Supplement: S2 File — (ZIP) [file pcbi.1006986.s003.zip › extrait_4hKM16021/4h-Z693_10_w2sdcGFP.tif]

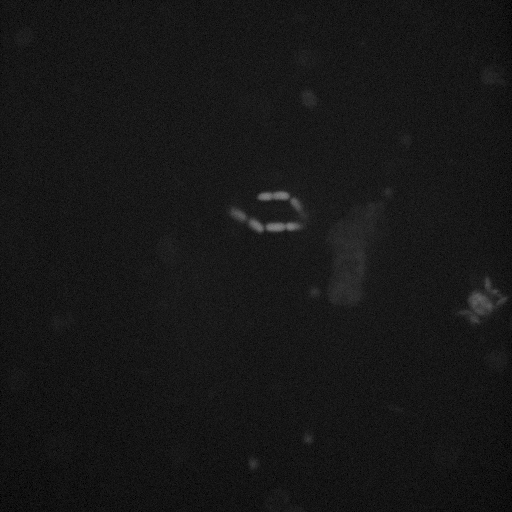

Supplement: S2 File — (ZIP) [file pcbi.1006986.s003.zip › extrait_4hKM16021/4h-Z693_10_w1sdcRFP.tif]

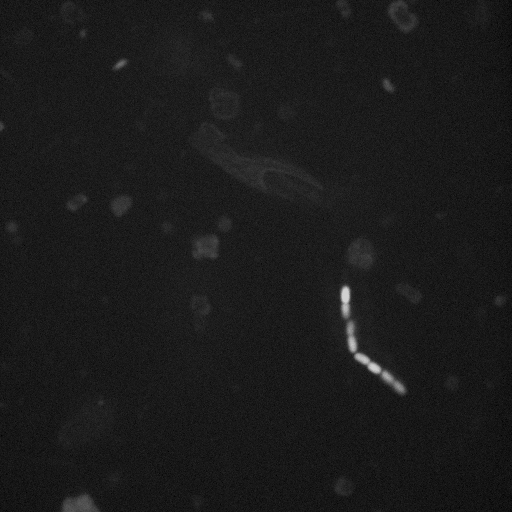

Supplement: S2 File — (ZIP) [file pcbi.1006986.s003.zip › extrait_4hKM16021/4h-Z693_11_w1sdcRFP.tif]

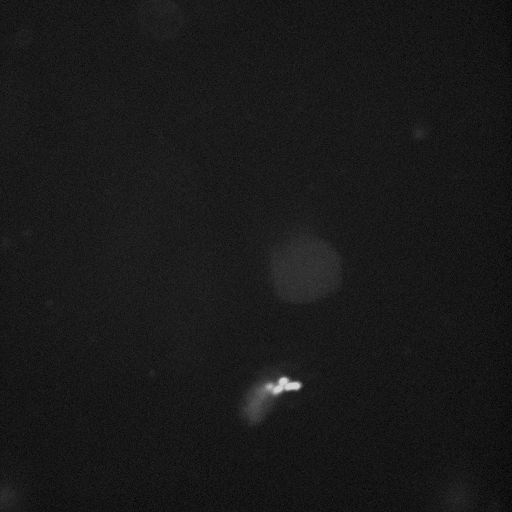

Supplement: S2 File — (ZIP) [file pcbi.1006986.s003.zip › extrait_4hKM16021/4h-Z692_23_w1sdcRFP.tif]

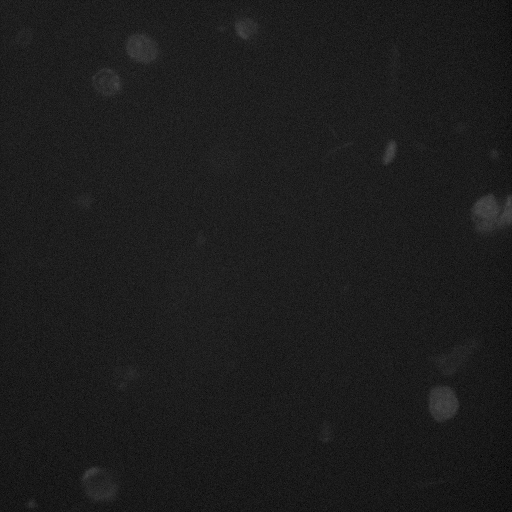

Supplement: S2 File — (ZIP) [file pcbi.1006986.s003.zip › extrait_4hKM16021/4h-Z694_2_w1sdcRFP.tif]

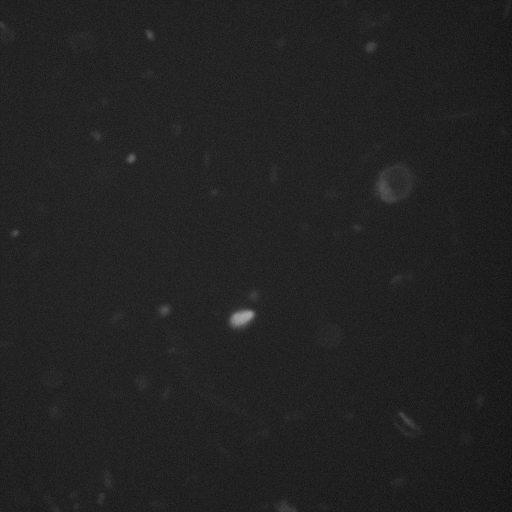

Supplement: S2 File — (ZIP) [file pcbi.1006986.s003.zip › extrait_4hKM16021/4h-Z694_24_w2sdcGFP.tif]

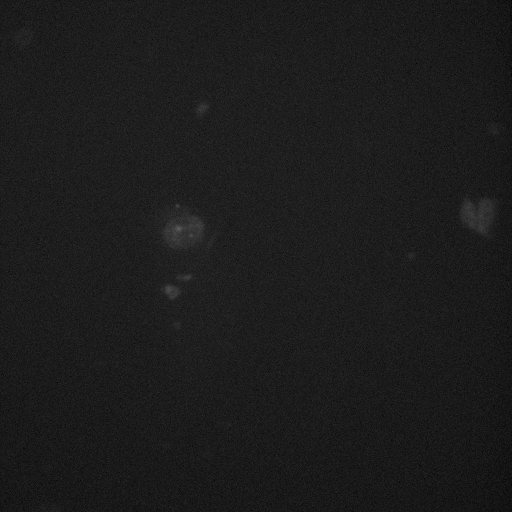

Supplement: S2 File — (ZIP) [file pcbi.1006986.s003.zip › extrait_4hKM16021/4h-Z694_3_w1sdcRFP.tif]

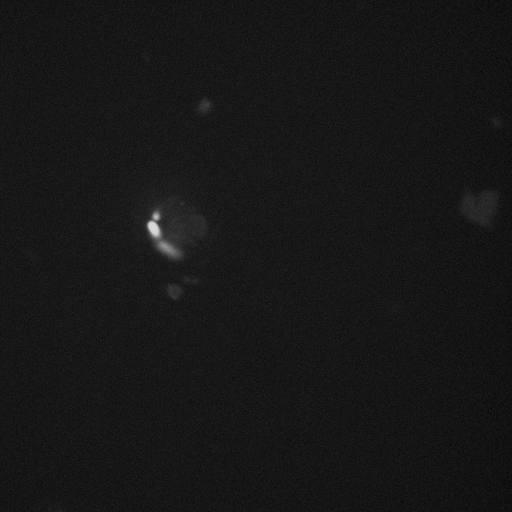

Supplement: S2 File — (ZIP) [file pcbi.1006986.s003.zip › extrait_4hKM16021/4h-Z694_3_w2sdcGFP.tif]

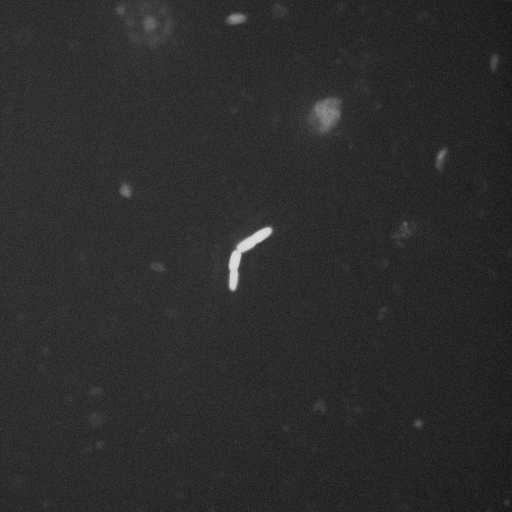

Supplement: S2 File — (ZIP) [file pcbi.1006986.s003.zip › extrait_4hKM16021/4h-Z693_14_w1sdcRFP.tif]

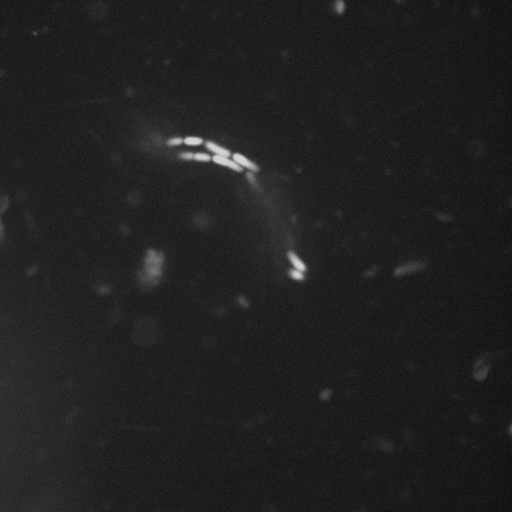

Supplement: S2 File — (ZIP) [file pcbi.1006986.s003.zip › extrait_4hKM16021/4h-Z692_35_w2sdcGFP.tif]

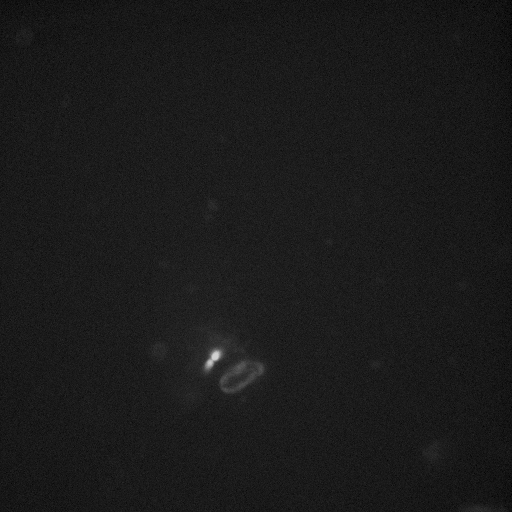

Supplement: S2 File — (ZIP) [file pcbi.1006986.s003.zip › extrait_4hKM16021/4h-Z693_3_w1sdcRFP.tif]
