# Supplementary figures and images for: Enchained growth and cluster dislocation: A possible mechanism for microbiota homeostasis (part 8 of 10)
Source: PLoS Comput Biol. 2019 May 3;15(5):e1006986. doi: 10.1371/journal.pcbi.1006986 (PMC6519844; doi:10.1371/journal.pcbi.1006986)

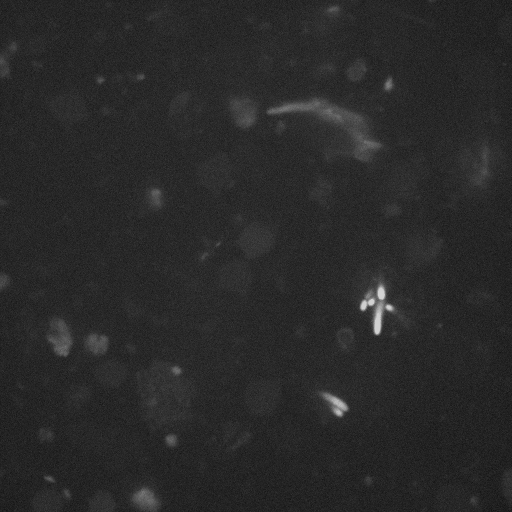

Supplement: S4 File — (ZIP) [file pcbi.1006986.s005.zip › extraitseq4h/KM16_013_4h_32_w1sdcRFP.tif]

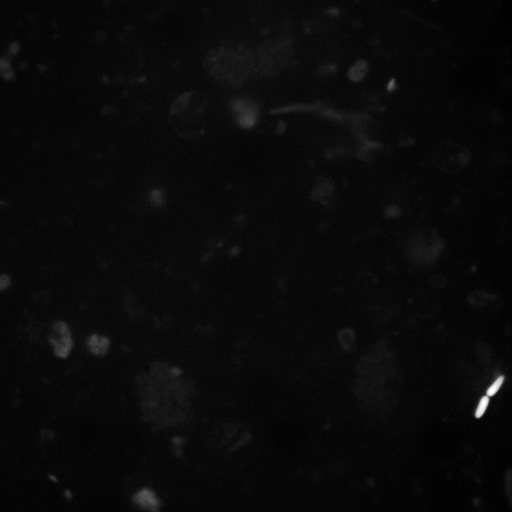

Supplement: S4 File — (ZIP) [file pcbi.1006986.s005.zip › extraitseq4h/KM16_013_4h_32_w2sdcGFP.tif]

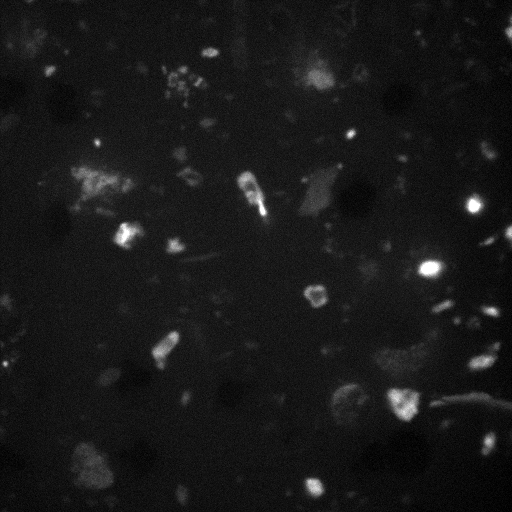

Supplement: S4 File — (ZIP) [file pcbi.1006986.s005.zip › extraitseq4h/KM16_013_4h_8_w2sdcGFP.tif]

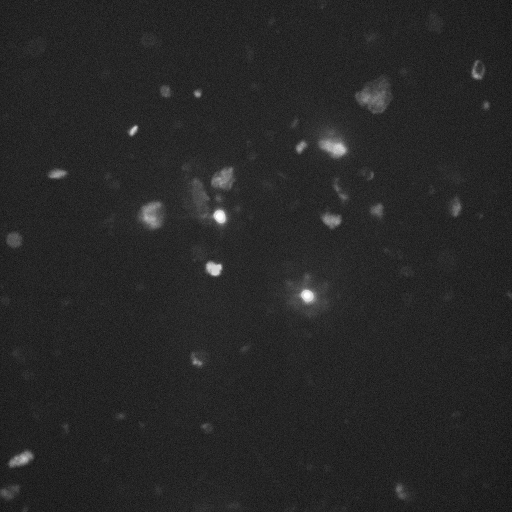

Supplement: S4 File — (ZIP) [file pcbi.1006986.s005.zip › extraitseq4h/KM16_016_4h_Cm_CCCP_21_w1sdcRFP.tif]

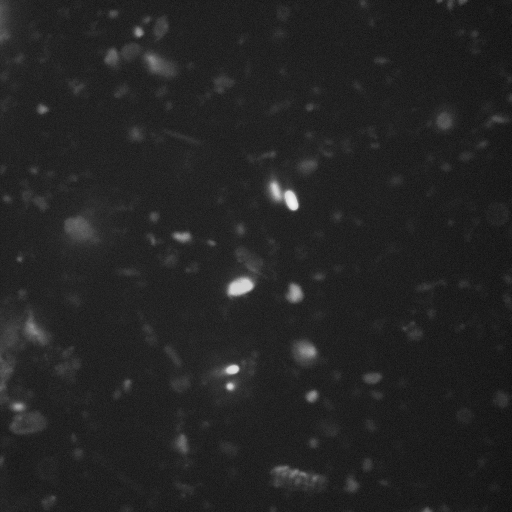

Supplement: S4 File — (ZIP) [file pcbi.1006986.s005.zip › extraitseq4h/KM16_016_4h_PBS_24_w2sdcGFP.tif]

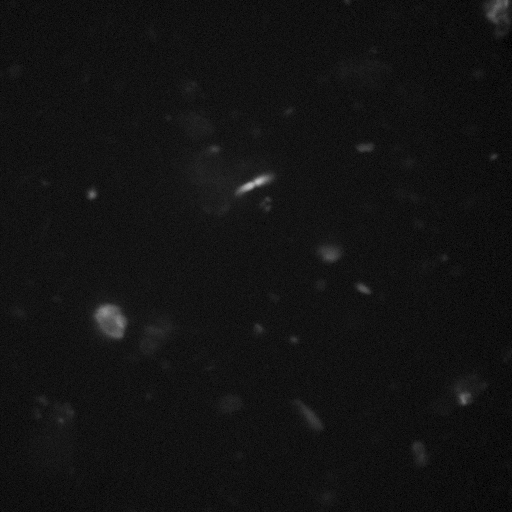

Supplement: S4 File — (ZIP) [file pcbi.1006986.s005.zip › extraitseq4h/KM16_016_4h_Cm_CCCP_14_w2sdcGFP.tif]

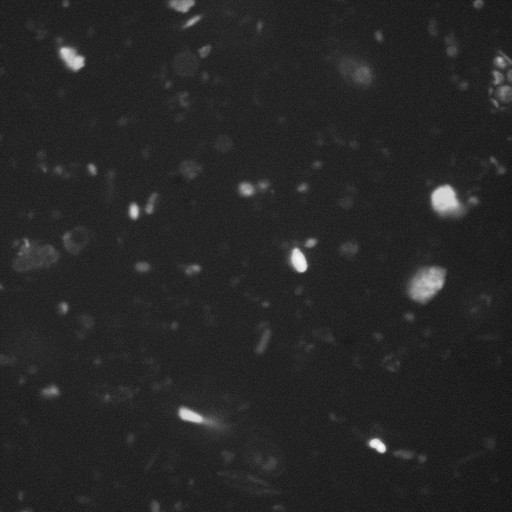

Supplement: S4 File — (ZIP) [file pcbi.1006986.s005.zip › extraitseq4h/KM16_016_4h_PBS_18_w2sdcGFP.tif]

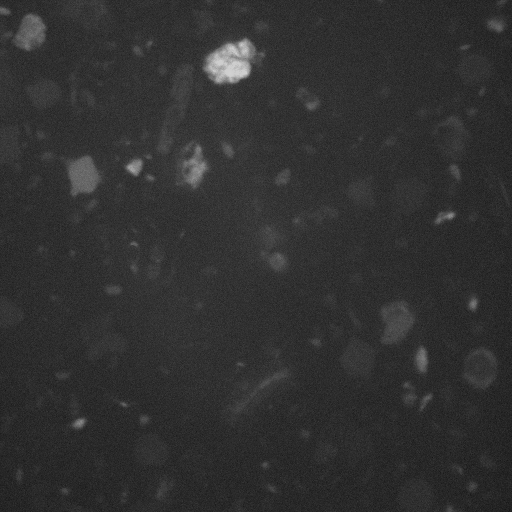

Supplement: S4 File — (ZIP) [file pcbi.1006986.s005.zip › extraitseq4h/KM16_013_4h_28_w1sdcRFP.tif]

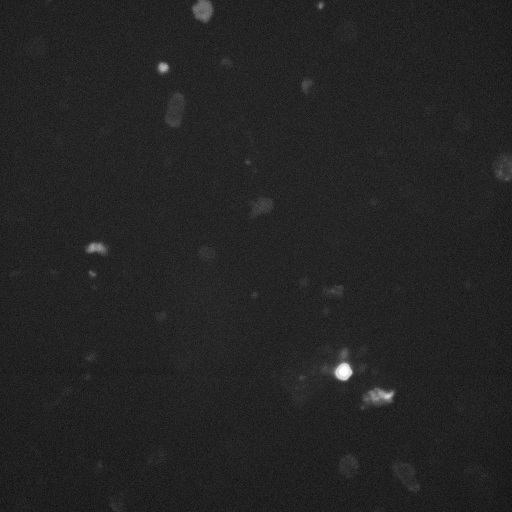

Supplement: S4 File — (ZIP) [file pcbi.1006986.s005.zip › extraitseq4h/KM16_016_4h_Cm_CCCP_6_w1sdcRFP.tif]

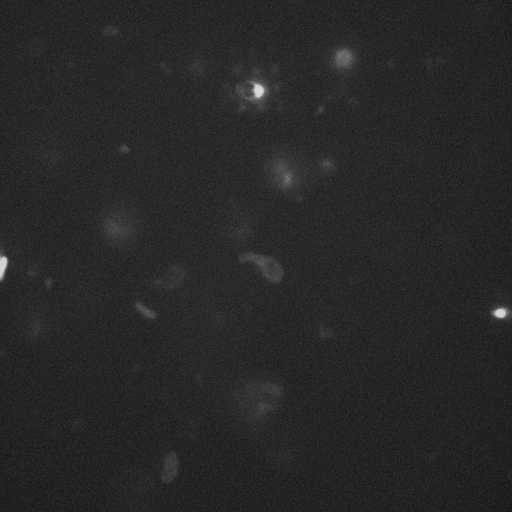

Supplement: S4 File — (ZIP) [file pcbi.1006986.s005.zip › extraitseq4h/KM16_016_4h_PBS_9_w1sdcRFP.tif]

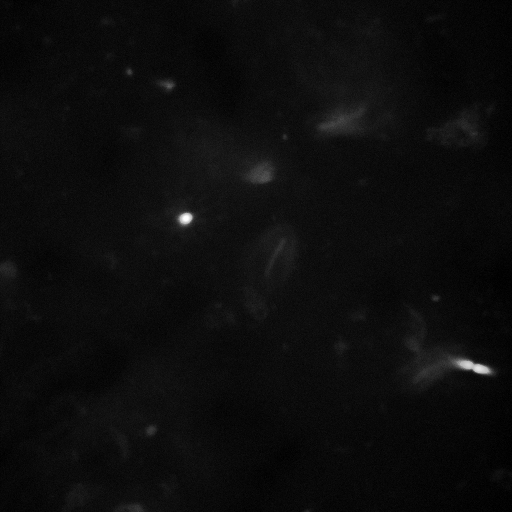

Supplement: S4 File — (ZIP) [file pcbi.1006986.s005.zip › extraitseq4h/KM16_013_4h_33_w2sdcGFP.tif]

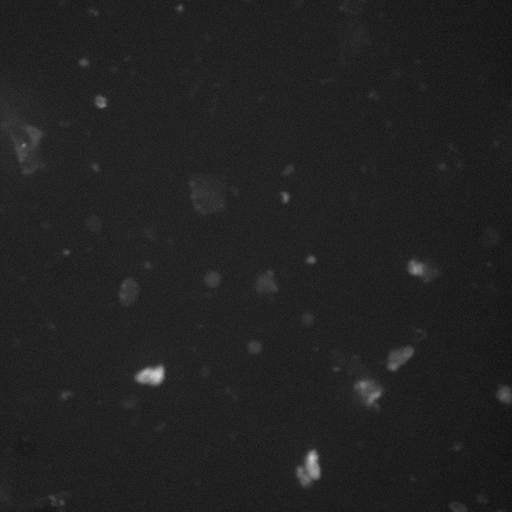

Supplement: S4 File — (ZIP) [file pcbi.1006986.s005.zip › extraitseq4h/KM16_016_4h_PBS_6_w2sdcGFP.tif]

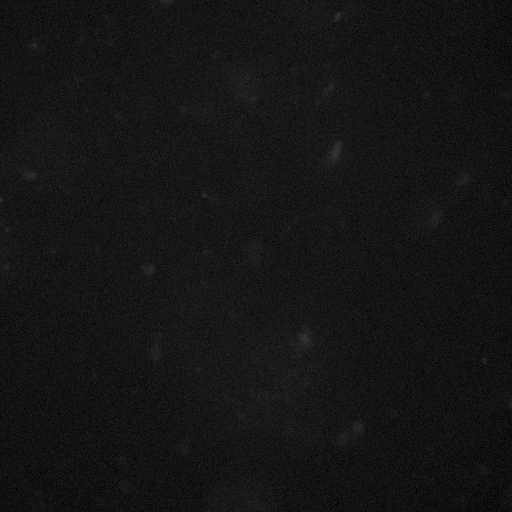

Supplement: S4 File — (ZIP) [file pcbi.1006986.s005.zip › extraitseq4h/KM16_013_4h_1_w1sdcRFP.tif]

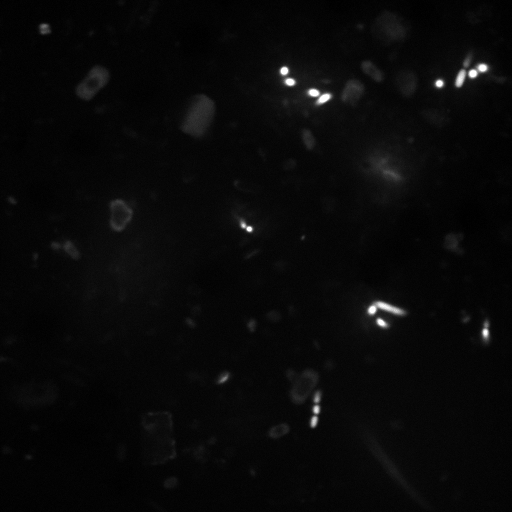

Supplement: S4 File — (ZIP) [file pcbi.1006986.s005.zip › extraitseq4h/KM16_013_4h_21_w2sdcGFP.tif]

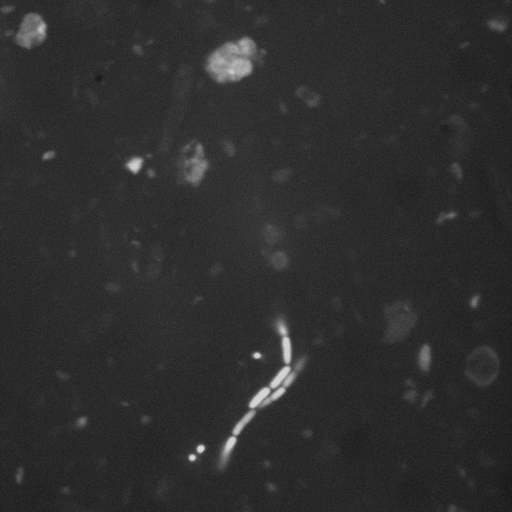

Supplement: S4 File — (ZIP) [file pcbi.1006986.s005.zip › extraitseq4h/KM16_013_4h_28_w2sdcGFP.tif]

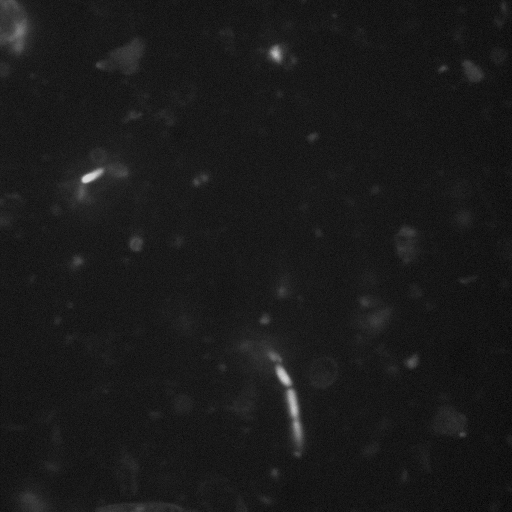

Supplement: S4 File — (ZIP) [file pcbi.1006986.s005.zip › extraitseq4h/KM16_016_4h_PBS_20_w2sdcGFP.tif]

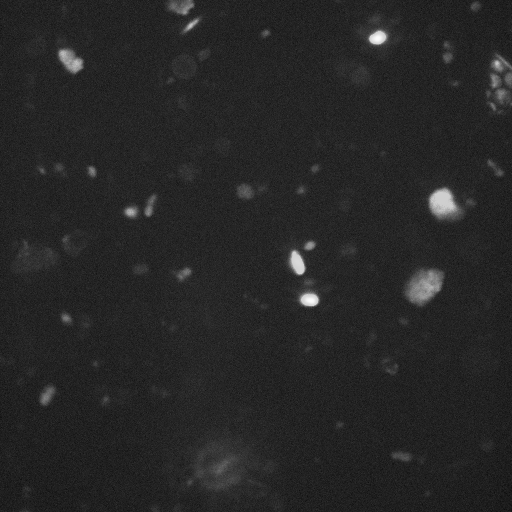

Supplement: S4 File — (ZIP) [file pcbi.1006986.s005.zip › extraitseq4h/KM16_016_4h_PBS_18_w1sdcRFP.tif]

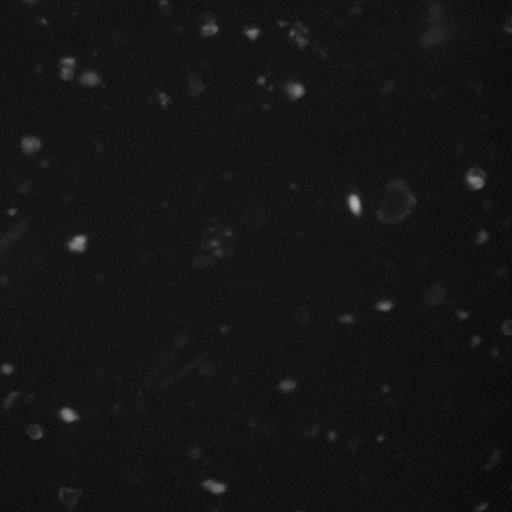

Supplement: S4 File — (ZIP) [file pcbi.1006986.s005.zip › extraitseq4h/KM16_016_4h_Cm_CCCP_25_w2sdcGFP.tif]

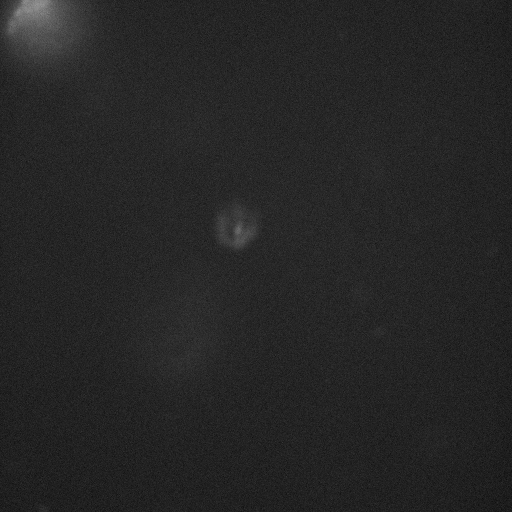

Supplement: S4 File — (ZIP) [file pcbi.1006986.s005.zip › extraitseq4h/KM16_016_4h_PBS_13_w1sdcRFP.tif]

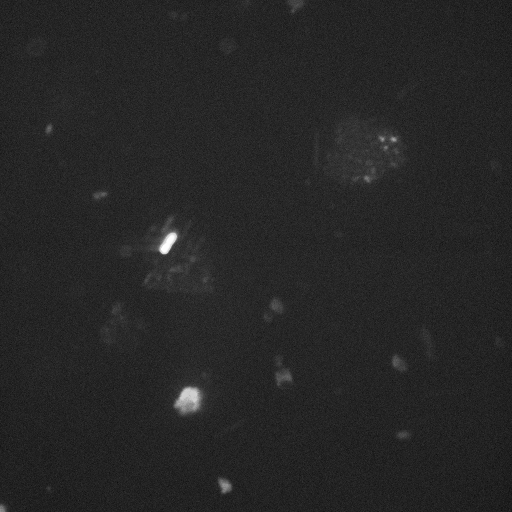

Supplement: S4 File — (ZIP) [file pcbi.1006986.s005.zip › extraitseq4h/KM16_016_4h_Cm_CCCP_2_w1sdcRFP.tif]

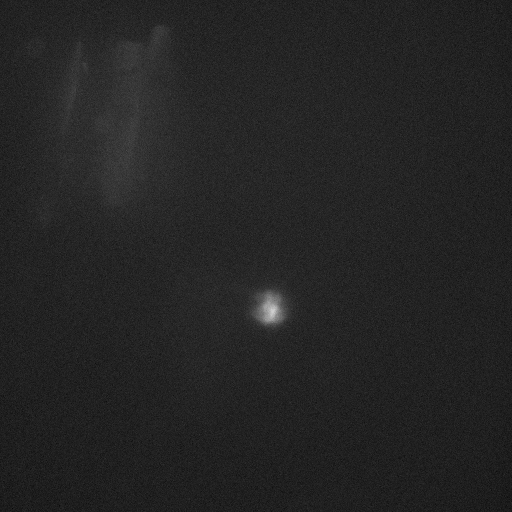

Supplement: S4 File — (ZIP) [file pcbi.1006986.s005.zip › extraitseq4h/KM16_016_4h_Cm_CCCP_11_w1sdcRFP.tif]

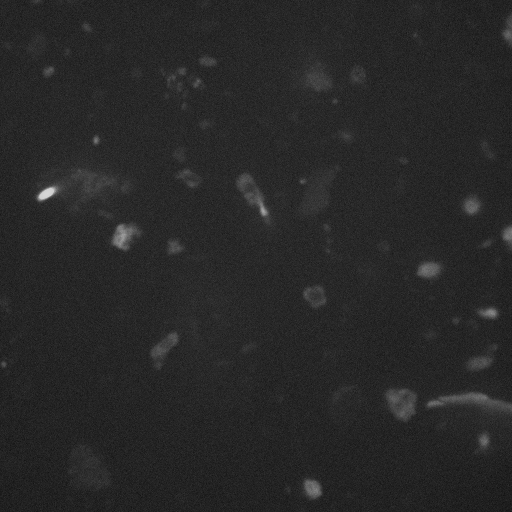

Supplement: S4 File — (ZIP) [file pcbi.1006986.s005.zip › extraitseq4h/KM16_013_4h_8_w1sdcRFP.tif]

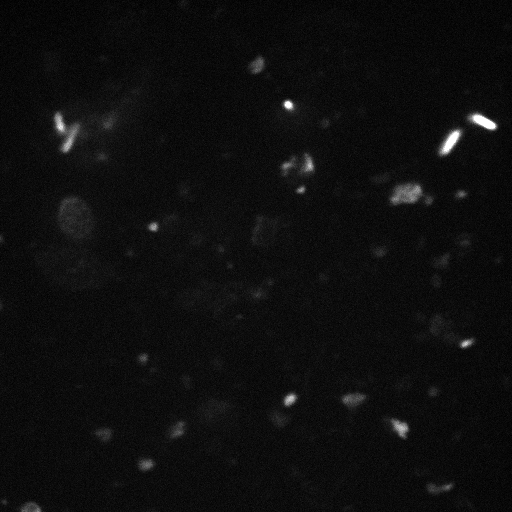

Supplement: S4 File — (ZIP) [file pcbi.1006986.s005.zip › extraitseq4h/KM16_016_4h_PBS_26_w2sdcGFP.tif]

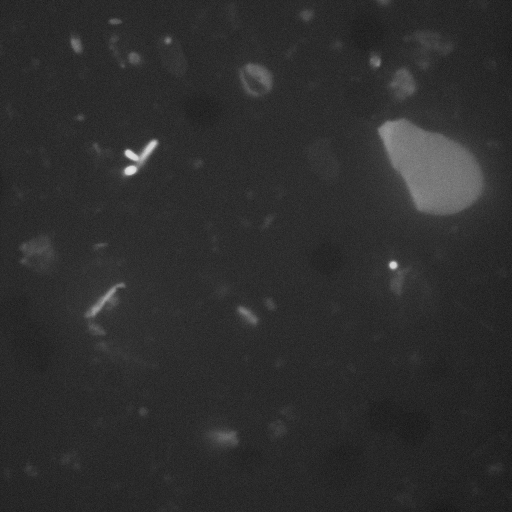

Supplement: S4 File — (ZIP) [file pcbi.1006986.s005.zip › extraitseq4h/KM16_013_4h_4_w2sdcGFP.tif]

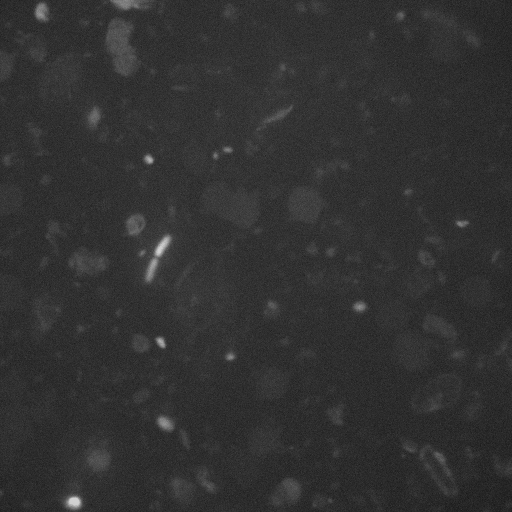

Supplement: S4 File — (ZIP) [file pcbi.1006986.s005.zip › extraitseq4h/KM16_013_4h_29_w1sdcRFP.tif]

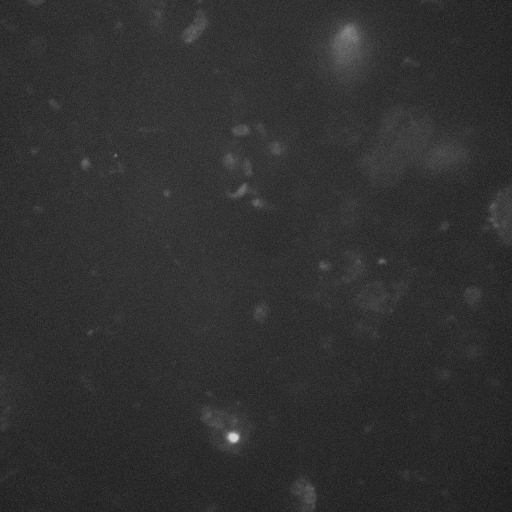

Supplement: S4 File — (ZIP) [file pcbi.1006986.s005.zip › extraitseq4h/KM16_013_4h_14_w1sdcRFP.tif]

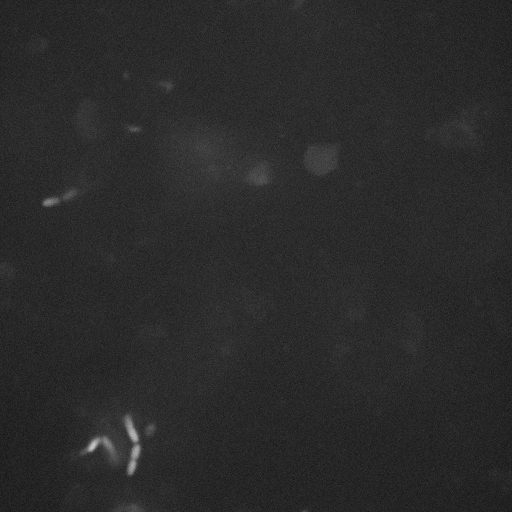

Supplement: S4 File — (ZIP) [file pcbi.1006986.s005.zip › extraitseq4h/KM16_013_4h_33_w1sdcRFP.tif]

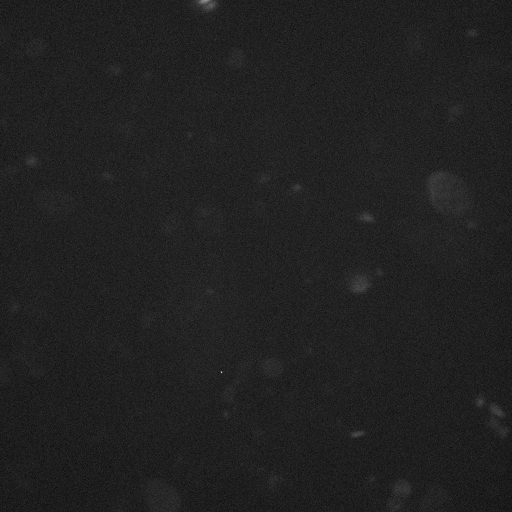

Supplement: S4 File — (ZIP) [file pcbi.1006986.s005.zip › extraitseq4h/KM16_016_4h_PBS_25_w1sdcRFP.tif]

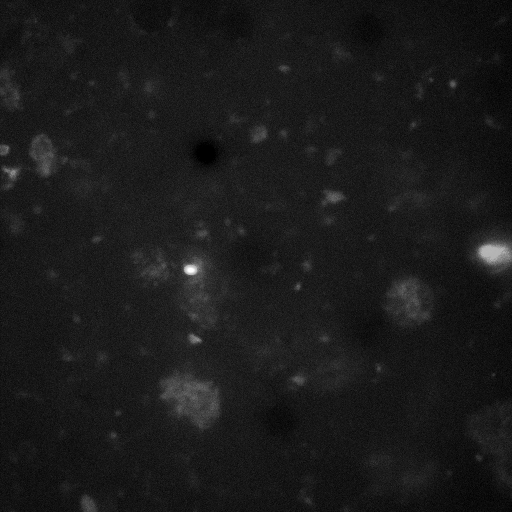

Supplement: S4 File — (ZIP) [file pcbi.1006986.s005.zip › extraitseq4h/KM16_013_4h_13_w2sdcGFP.tif]

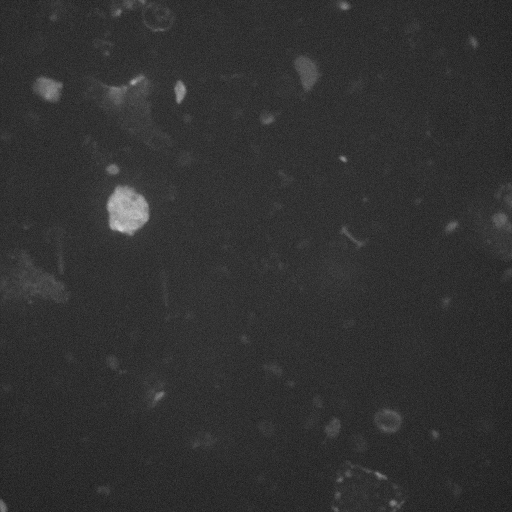

Supplement: S4 File — (ZIP) [file pcbi.1006986.s005.zip › extraitseq4h/KM16_013_4h_3_w1sdcRFP.tif]

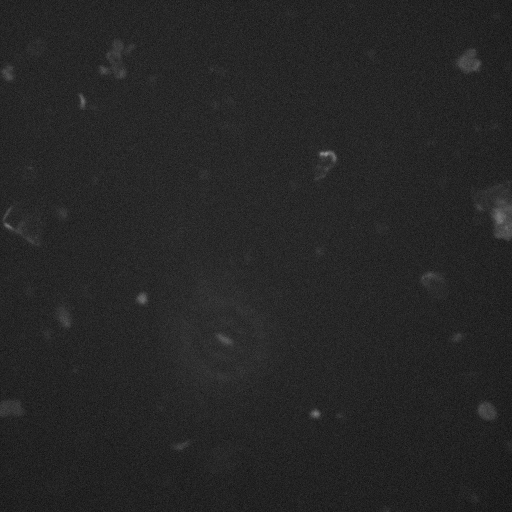

Supplement: S4 File — (ZIP) [file pcbi.1006986.s005.zip › extraitseq4h/KM16_016_4h_Cm_CCCP_8_w1sdcRFP.tif]

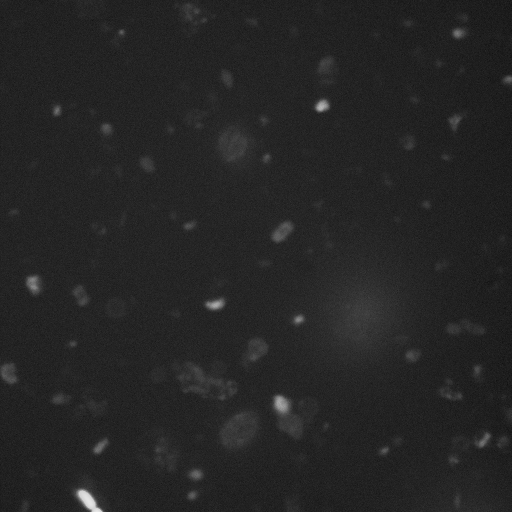

Supplement: S4 File — (ZIP) [file pcbi.1006986.s005.zip › extraitseq4h/KM16_016_4h_Cm_CCCP_12_w2sdcGFP.tif]

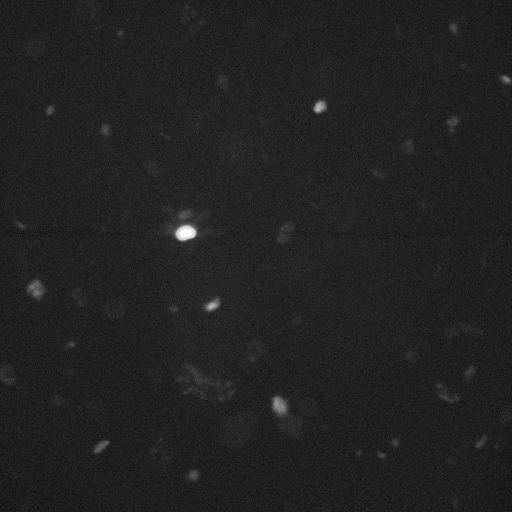

Supplement: S4 File — (ZIP) [file pcbi.1006986.s005.zip › extraitseq4h/KM16_016_4h_Cm_CCCP_12_w1sdcRFP.tif]

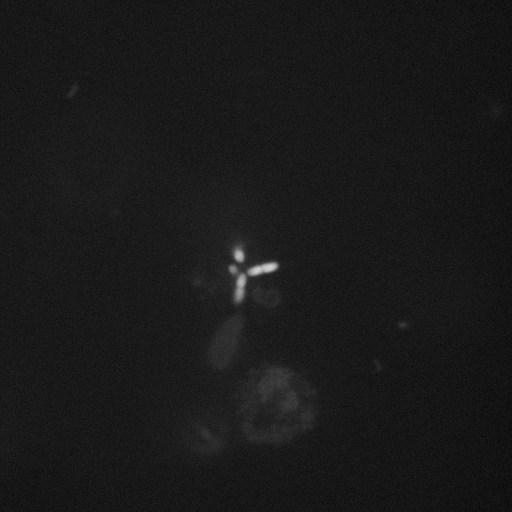

Supplement: S4 File — (ZIP) [file pcbi.1006986.s005.zip › extraitseq4h/KM16_016_4h_Cm_CCCP_4_w2sdcGFP.tif]

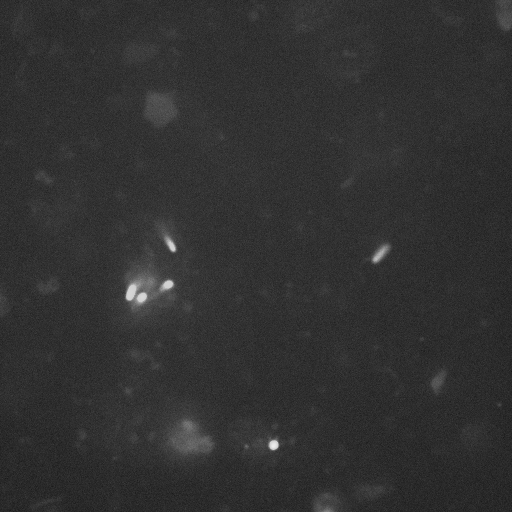

Supplement: S4 File — (ZIP) [file pcbi.1006986.s005.zip › extraitseq4h/KM16_013_4h_19_w1sdcRFP.tif]

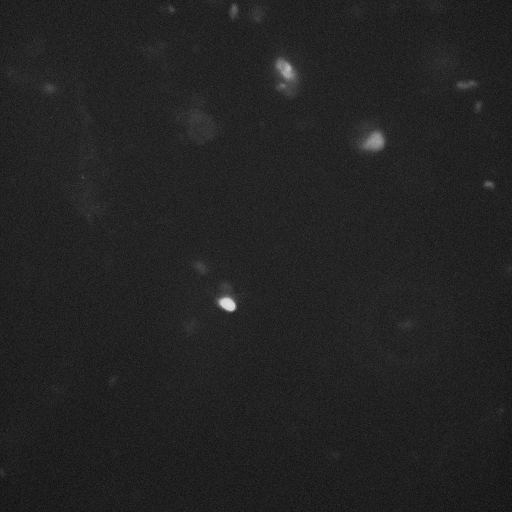

Supplement: S4 File — (ZIP) [file pcbi.1006986.s005.zip › extraitseq4h/KM16_016_4h_Cm_CCCP_10_w1sdcRFP.tif]

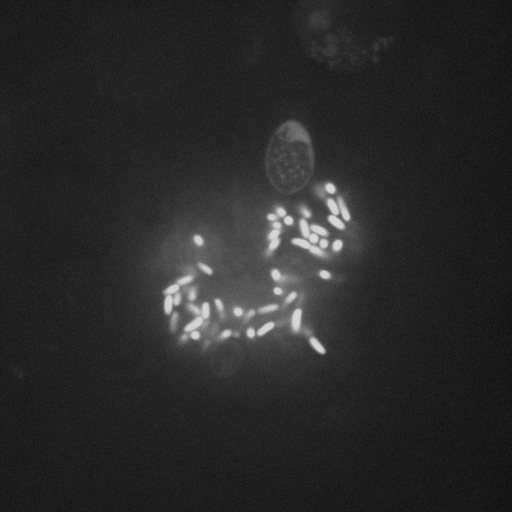

Supplement: S4 File — (ZIP) [file pcbi.1006986.s005.zip › extraitseq4h/KM16_016_4h_PBS_15_w2sdcGFP.tif]

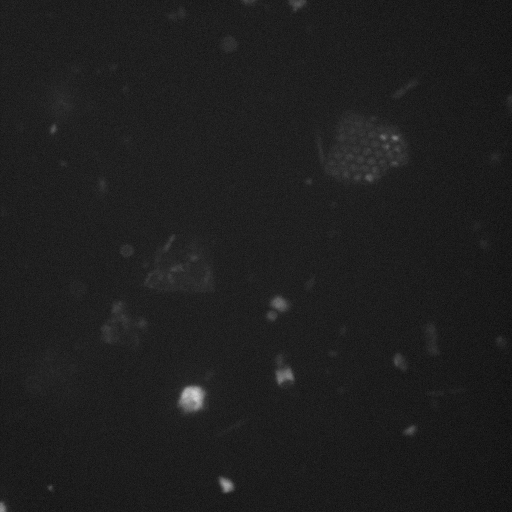

Supplement: S4 File — (ZIP) [file pcbi.1006986.s005.zip › extraitseq4h/KM16_016_4h_Cm_CCCP_2_w2sdcGFP.tif]

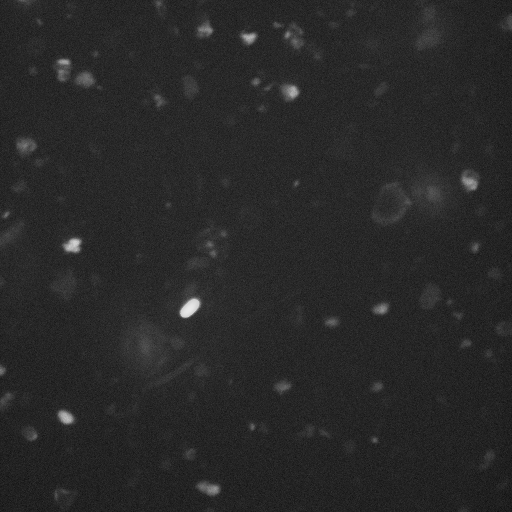

Supplement: S4 File — (ZIP) [file pcbi.1006986.s005.zip › extraitseq4h/KM16_016_4h_Cm_CCCP_25_w1sdcRFP.tif]

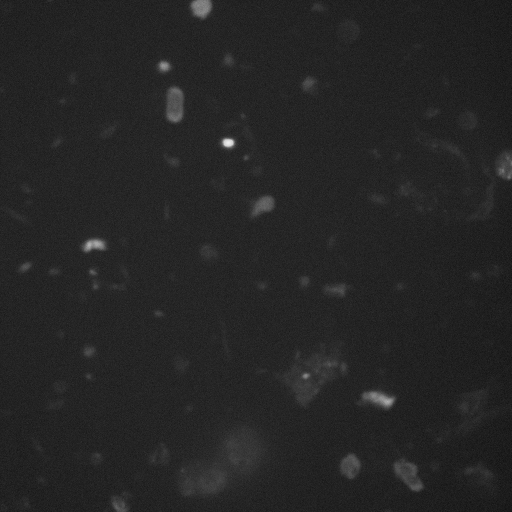

Supplement: S4 File — (ZIP) [file pcbi.1006986.s005.zip › extraitseq4h/KM16_016_4h_Cm_CCCP_6_w2sdcGFP.tif]

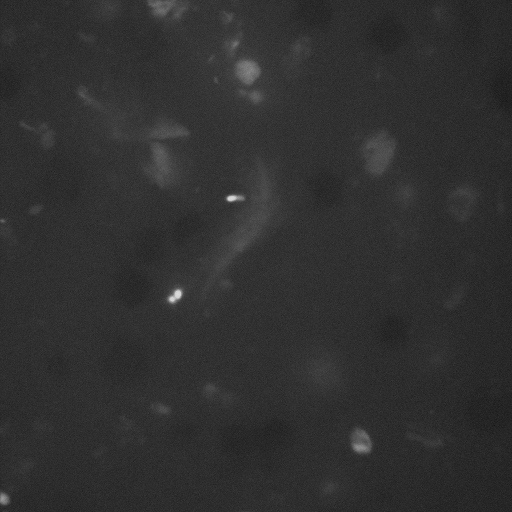

Supplement: S4 File — (ZIP) [file pcbi.1006986.s005.zip › extraitseq4h/KM16_013_4h_11_w2sdcGFP.tif]

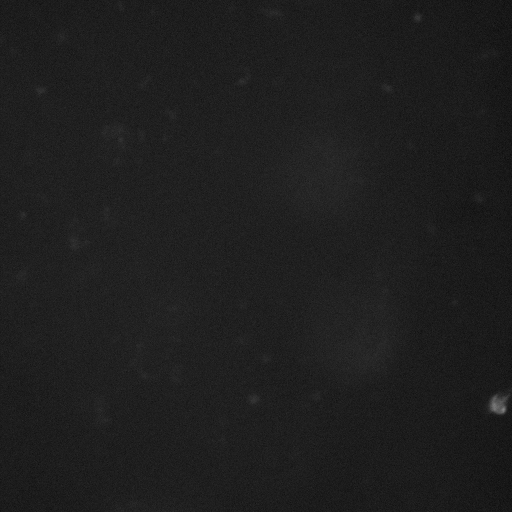

Supplement: S4 File — (ZIP) [file pcbi.1006986.s005.zip › extraitseq4h/KM16_016_4h_Cm_CCCP_16_w2sdcGFP.tif]

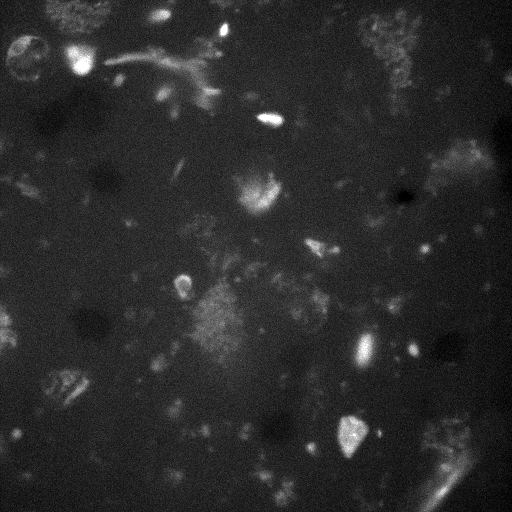

Supplement: S4 File — (ZIP) [file pcbi.1006986.s005.zip › extraitseq4h/KM16_013_4h_9_w2sdcGFP.tif]

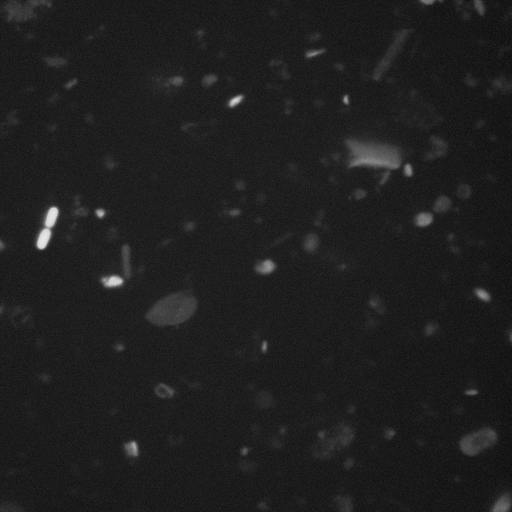

Supplement: S4 File — (ZIP) [file pcbi.1006986.s005.zip › extraitseq4h/KM16_016_4h_Cm_CCCP_15_w2sdcGFP.tif]

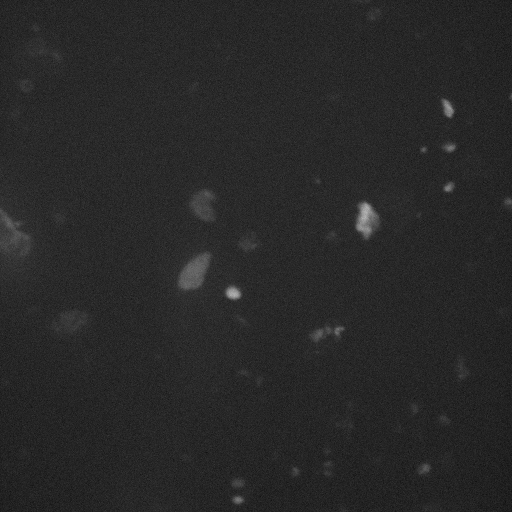

Supplement: S4 File — (ZIP) [file pcbi.1006986.s005.zip › extraitseq4h/KM16_016_4h_Cm_CCCP_5_w1sdcRFP.tif]

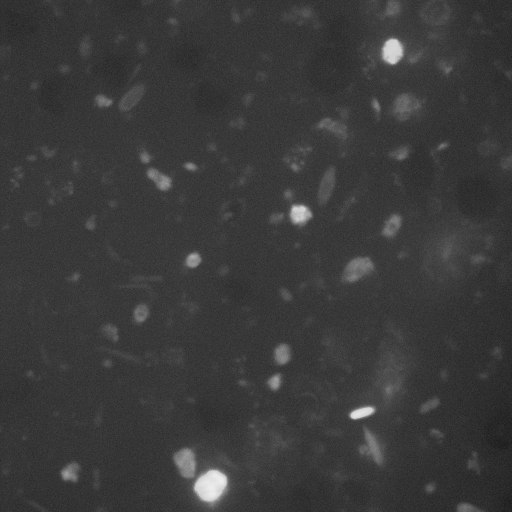

Supplement: S4 File — (ZIP) [file pcbi.1006986.s005.zip › extraitseq4h/KM16_013_4h_12_w2sdcGFP.tif]

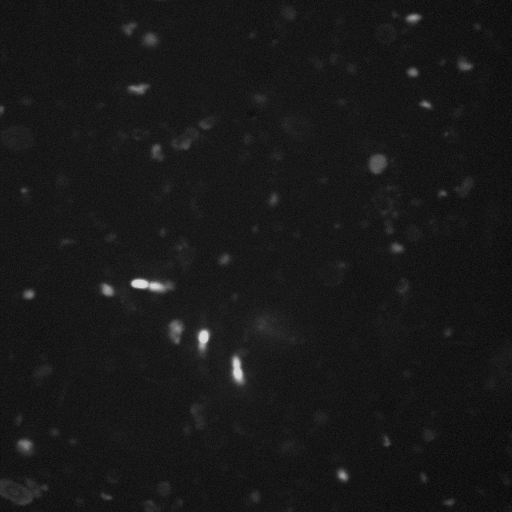

Supplement: S4 File — (ZIP) [file pcbi.1006986.s005.zip › extraitseq4h/KM16_016_4h_PBS_22_w2sdcGFP.tif]

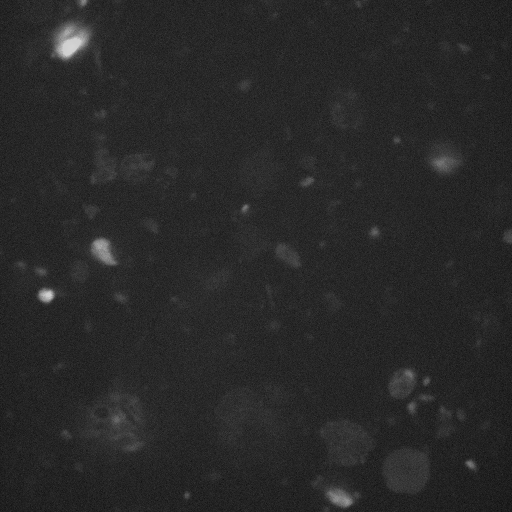

Supplement: S4 File — (ZIP) [file pcbi.1006986.s005.zip › extraitseq4h/KM16_016_4h_PBS_21_w1sdcRFP.tif]

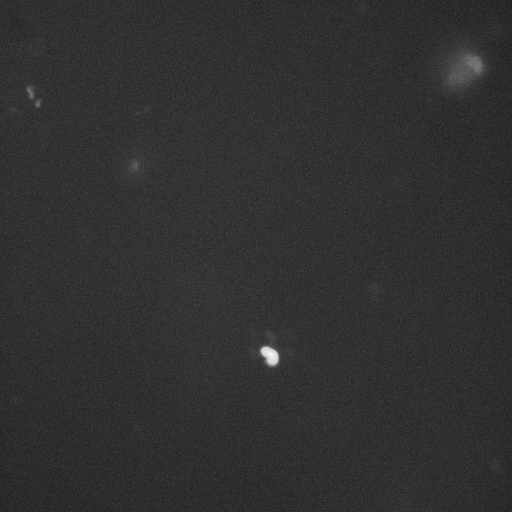

Supplement: S4 File — (ZIP) [file pcbi.1006986.s005.zip › extraitseq4h/KM16_016_4h_PBS_7_w1sdcRFP.tif]

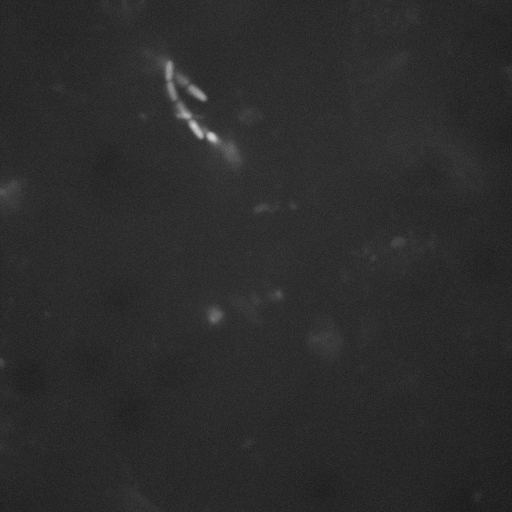

Supplement: S4 File — (ZIP) [file pcbi.1006986.s005.zip › extraitseq4h/KM16_013_4h_17_w2sdcGFP.tif]

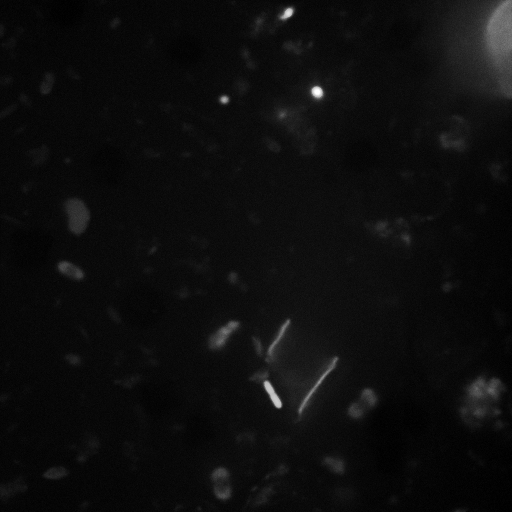

Supplement: S4 File — (ZIP) [file pcbi.1006986.s005.zip › extraitseq4h/KM16_013_4h_6_w2sdcGFP.tif]

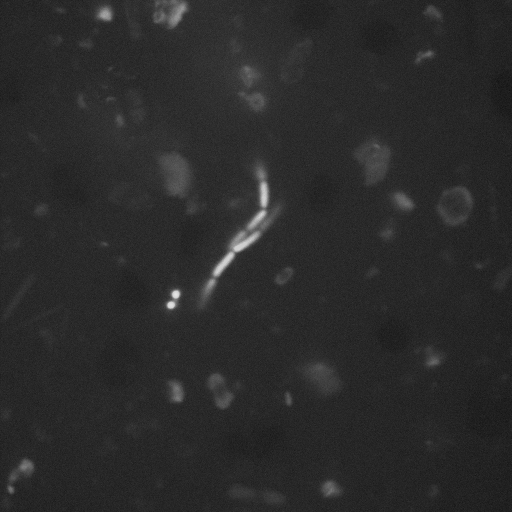

Supplement: S4 File — (ZIP) [file pcbi.1006986.s005.zip › extraitseq4h/KM16_013_4h_10_w2sdcGFP.tif]

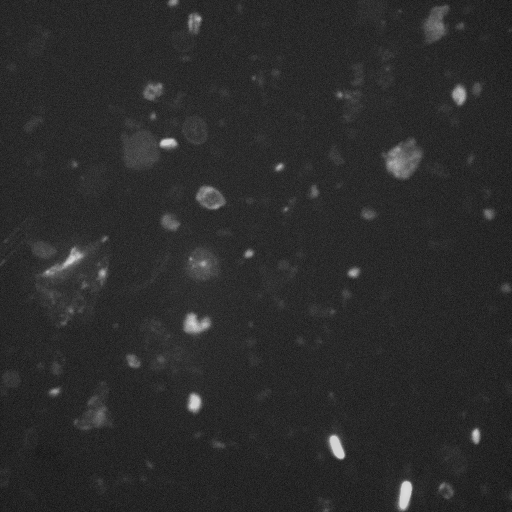

Supplement: S4 File — (ZIP) [file pcbi.1006986.s005.zip › extraitseq4h/KM16_016_4h_Cm_CCCP_24_w1sdcRFP.tif]

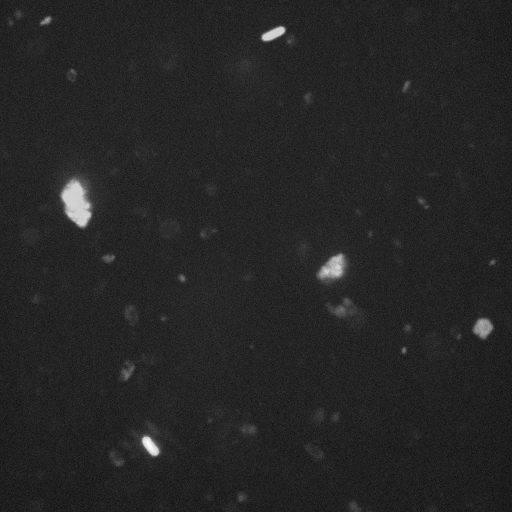

Supplement: S4 File — (ZIP) [file pcbi.1006986.s005.zip › extraitseq4h/KM16_016_4h_PBS_23_w1sdcRFP.tif]

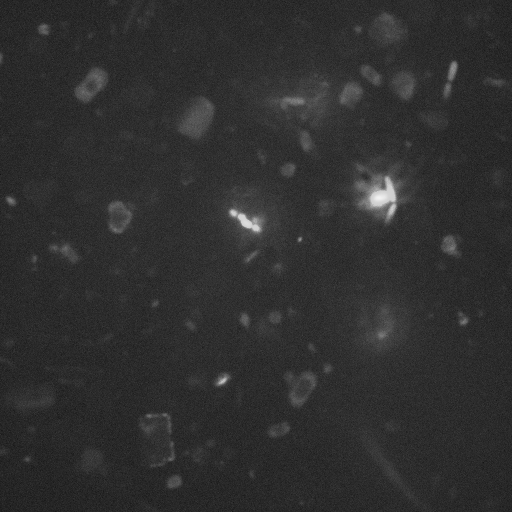

Supplement: S4 File — (ZIP) [file pcbi.1006986.s005.zip › extraitseq4h/KM16_013_4h_21_w1sdcRFP.tif]

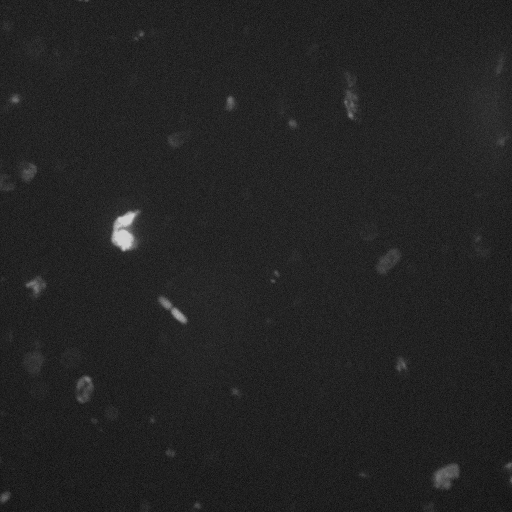

Supplement: S4 File — (ZIP) [file pcbi.1006986.s005.zip › extraitseq4h/KM16_016_4h_Cm_CCCP_7_w1sdcRFP.tif]

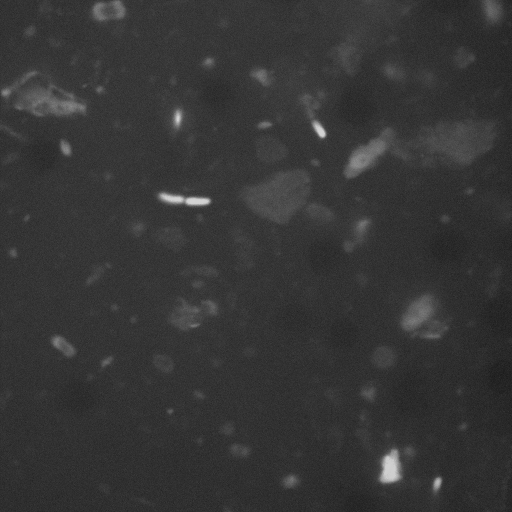

Supplement: S4 File — (ZIP) [file pcbi.1006986.s005.zip › extraitseq4h/KM16_013_4h_24_w2sdcGFP.tif]

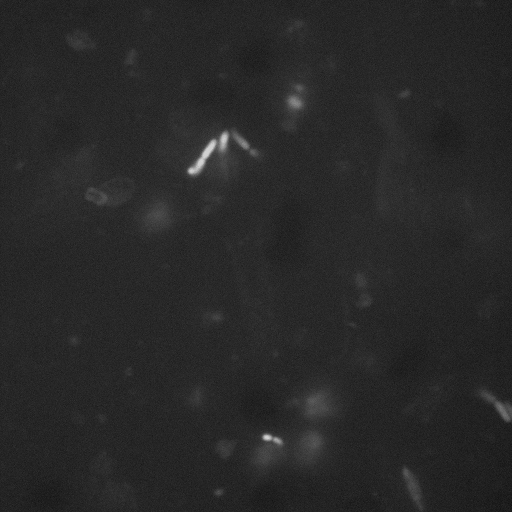

Supplement: S4 File — (ZIP) [file pcbi.1006986.s005.zip › extraitseq4h/KM16_013_4h_15_w2sdcGFP.tif]

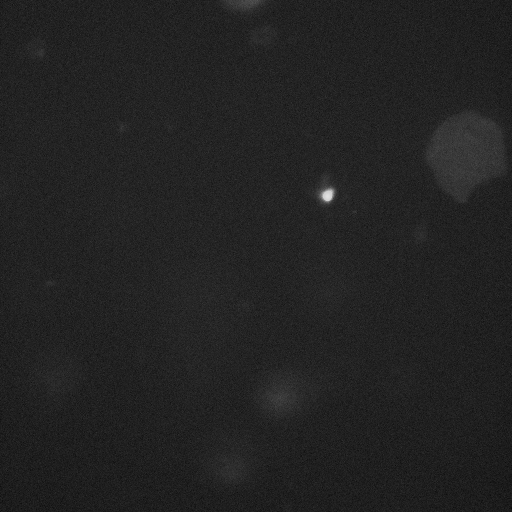

Supplement: S4 File — (ZIP) [file pcbi.1006986.s005.zip › extraitseq4h/KM16_016_4h_PBS_5_w1sdcRFP.tif]

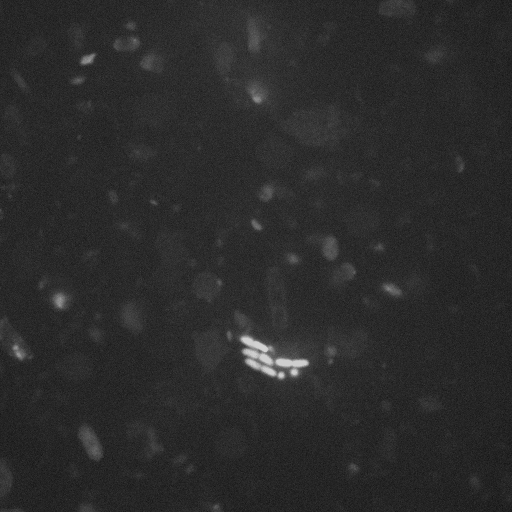

Supplement: S4 File — (ZIP) [file pcbi.1006986.s005.zip › extraitseq4h/KM16_013_4h_30_w1sdcRFP.tif]

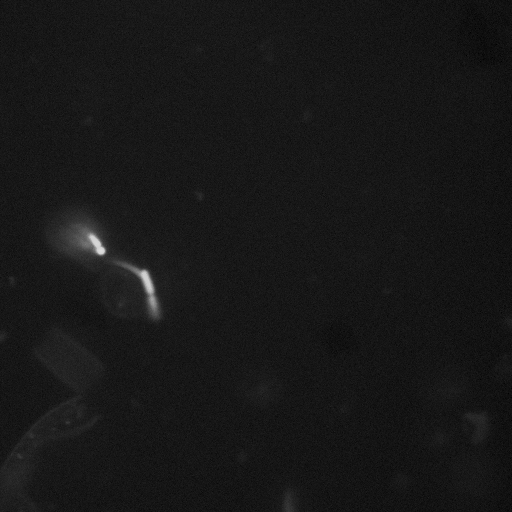

Supplement: S4 File — (ZIP) [file pcbi.1006986.s005.zip › extraitseq4h/KM16_016_4h_PBS_3_w2sdcGFP.tif]

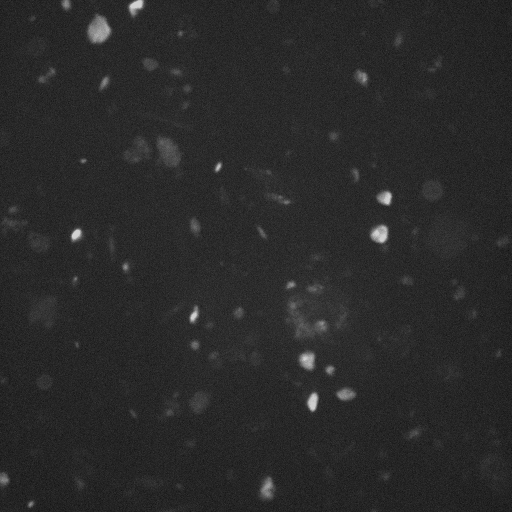

Supplement: S4 File — (ZIP) [file pcbi.1006986.s005.zip › extraitseq4h/KM16_016_4h_Cm_CCCP_23_w1sdcRFP.tif]

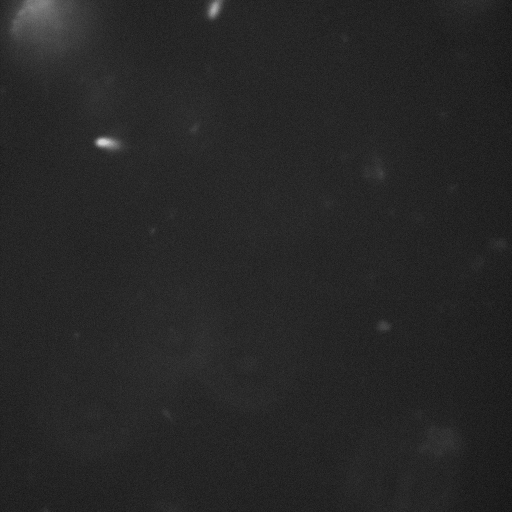

Supplement: S4 File — (ZIP) [file pcbi.1006986.s005.zip › extraitseq4h/KM16_016_4h_PBS_13_w2sdcGFP.tif]

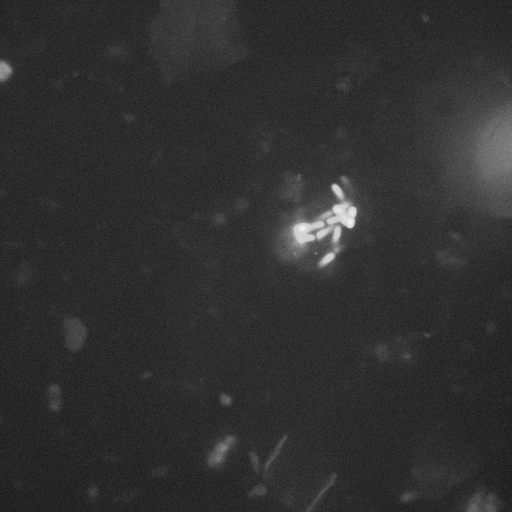

Supplement: S4 File — (ZIP) [file pcbi.1006986.s005.zip › extraitseq4h/KM16_013_4h_5_w1sdcRFP.tif]

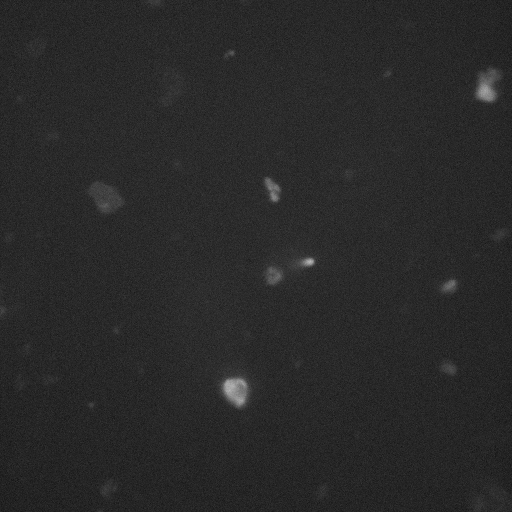

Supplement: S4 File — (ZIP) [file pcbi.1006986.s005.zip › extraitseq4h/KM16_016_4h_Cm_CCCP_3_w1sdcRFP.tif]

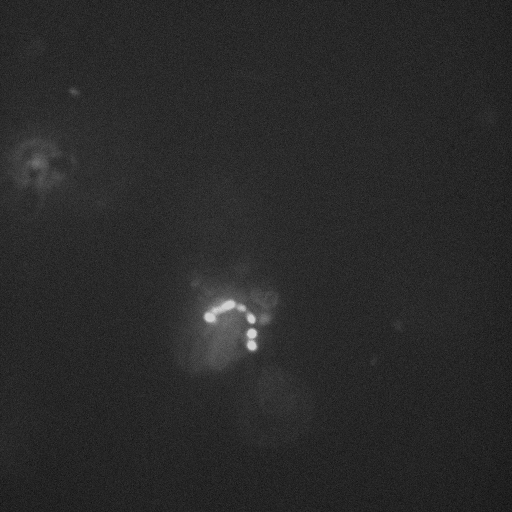

Supplement: S4 File — (ZIP) [file pcbi.1006986.s005.zip › extraitseq4h/KM16_016_4h_Cm_CCCP_4_w1sdcRFP.tif]

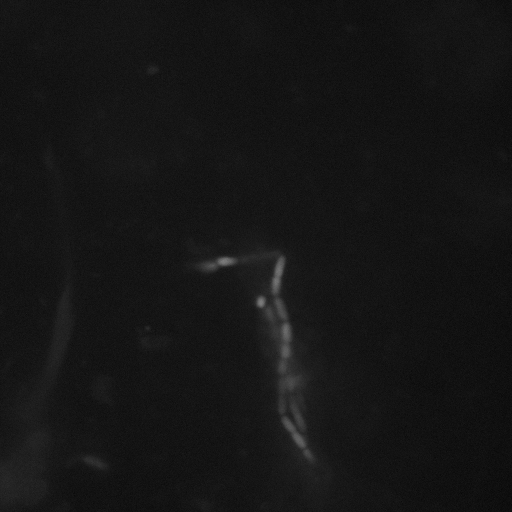

Supplement: S5 File — (ZIP) [file pcbi.1006986.s006.zip › extraitseq5h/KM16_014_5h_30_w2sdcGFP.tif]

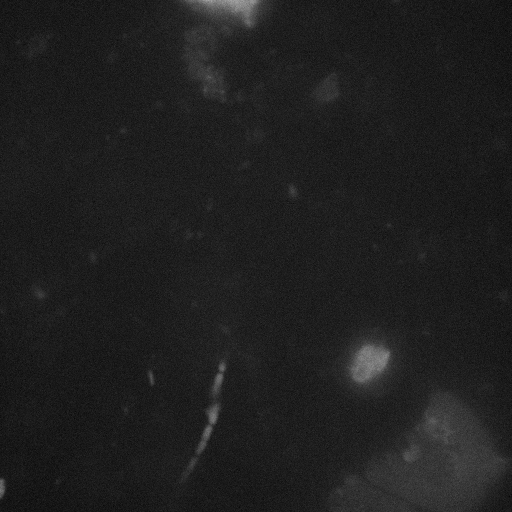

Supplement: S5 File — (ZIP) [file pcbi.1006986.s006.zip › extraitseq5h/KM16_014_5h_6_w1sdcRFP.tif]

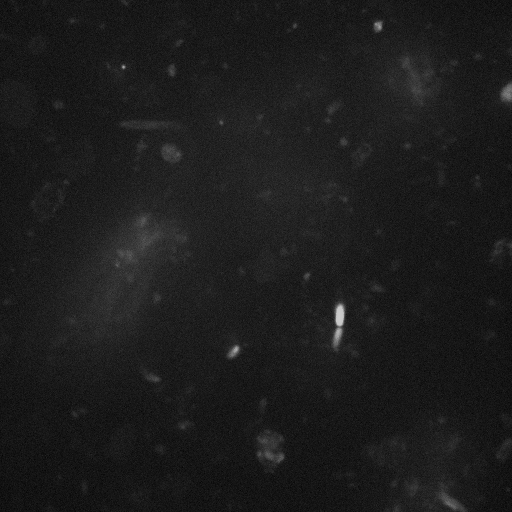

Supplement: S5 File — (ZIP) [file pcbi.1006986.s006.zip › extraitseq5h/KM16_013_5h_13_w1sdcRFP.tif]

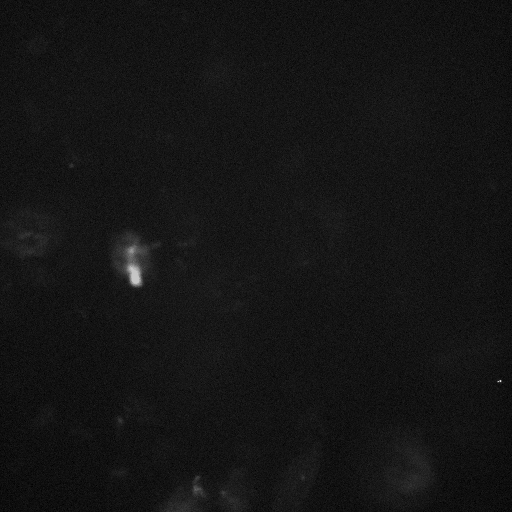

Supplement: S5 File — (ZIP) [file pcbi.1006986.s006.zip › extraitseq5h/KM16_013_5h_27_w1sdcRFP.tif]

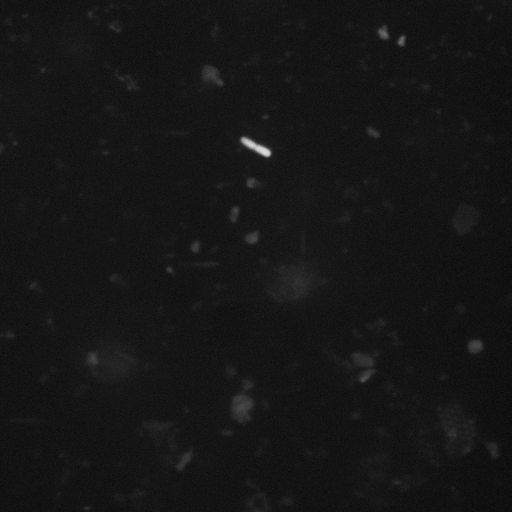

Supplement: S5 File — (ZIP) [file pcbi.1006986.s006.zip › extraitseq5h/KM16_013_5h_14_w2sdcGFP.tif]

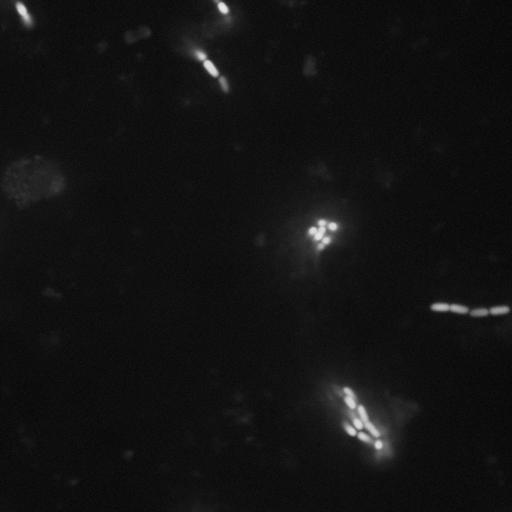

Supplement: S5 File — (ZIP) [file pcbi.1006986.s006.zip › extraitseq5h/KM16_013_5h_33_w2sdcGFP.tif]

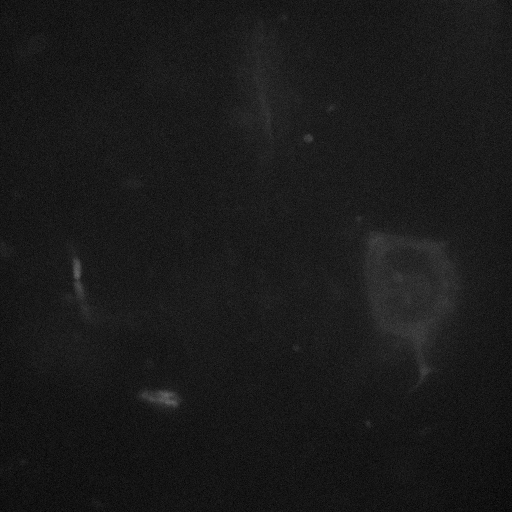

Supplement: S5 File — (ZIP) [file pcbi.1006986.s006.zip › extraitseq5h/KM16_014_5h_29_w1sdcRFP.tif]

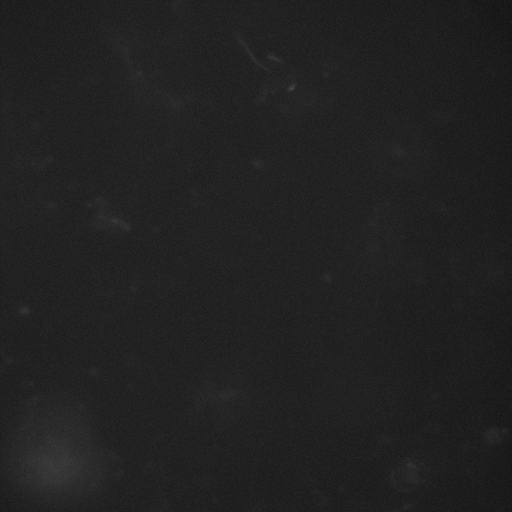

Supplement: S5 File — (ZIP) [file pcbi.1006986.s006.zip › extraitseq5h/KM16_014_5h_24_w2sdcGFP.tif]

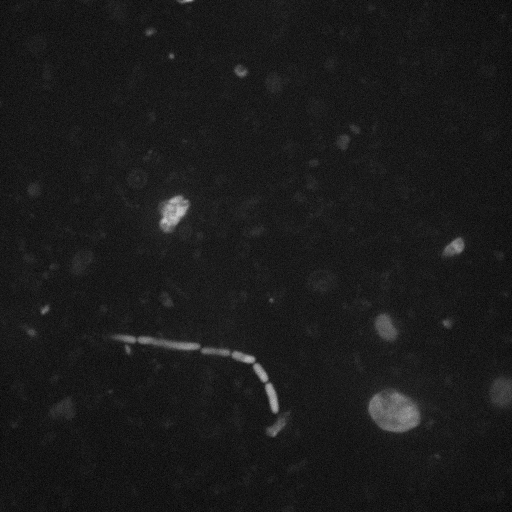

Supplement: S5 File — (ZIP) [file pcbi.1006986.s006.zip › extraitseq5h/KM16_014_5h_17_w1sdcRFP.tif]

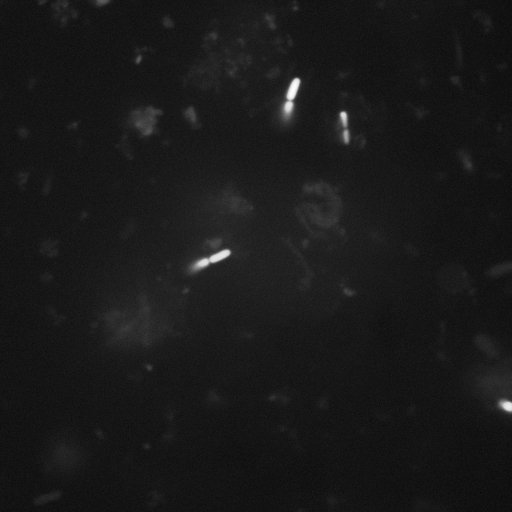

Supplement: S5 File — (ZIP) [file pcbi.1006986.s006.zip › extraitseq5h/KM16_013_5h_31_w2sdcGFP.tif]

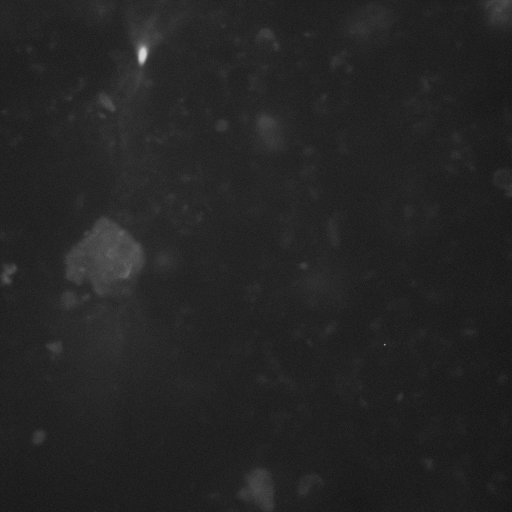

Supplement: S5 File — (ZIP) [file pcbi.1006986.s006.zip › extraitseq5h/KM16_014_5h_34_w2sdcGFP.tif]

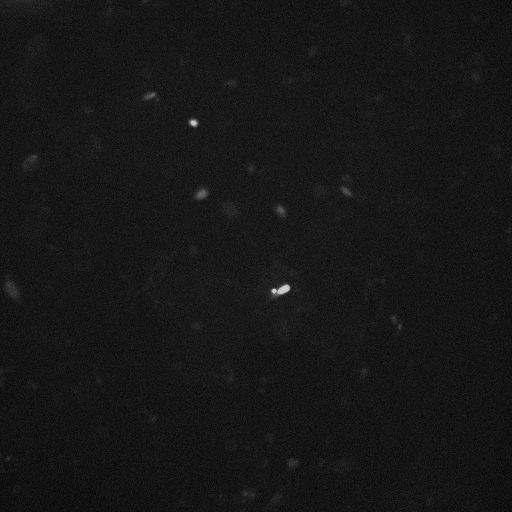

Supplement: S5 File — (ZIP) [file pcbi.1006986.s006.zip › extraitseq5h/KM16_014_5h_1_w1sdcRFP.tif]

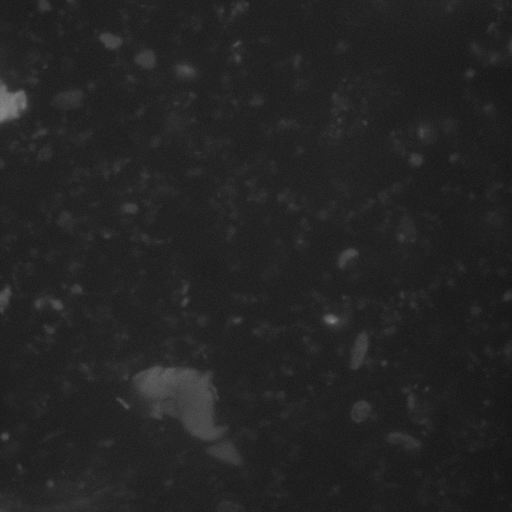

Supplement: S5 File — (ZIP) [file pcbi.1006986.s006.zip › extraitseq5h/KM16_014_5h_32_w2sdcGFP.tif]

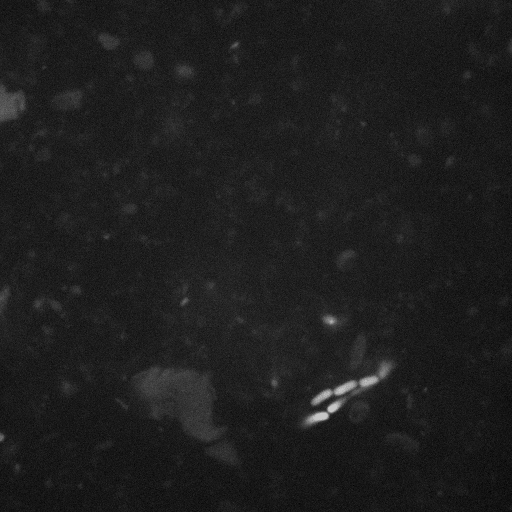

Supplement: S5 File — (ZIP) [file pcbi.1006986.s006.zip › extraitseq5h/KM16_014_5h_32_w1sdcRFP.tif]

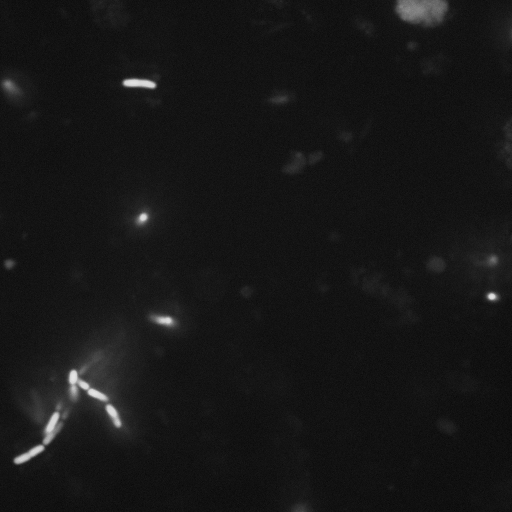

Supplement: S5 File — (ZIP) [file pcbi.1006986.s006.zip › extraitseq5h/KM16_013_5h_23_w2sdcGFP.tif]

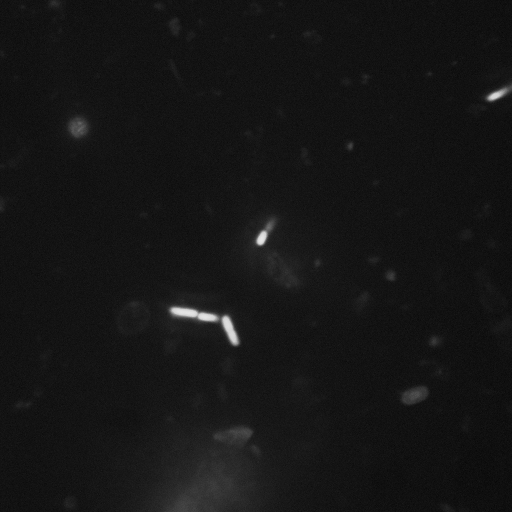

Supplement: S5 File — (ZIP) [file pcbi.1006986.s006.zip › extraitseq5h/KM16_013_5h_4_w2sdcGFP.tif]

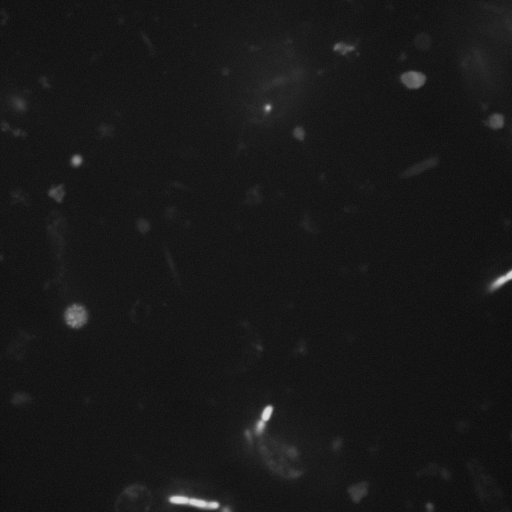

Supplement: S5 File — (ZIP) [file pcbi.1006986.s006.zip › extraitseq5h/KM16_013_5h_3_w2sdcGFP.tif]

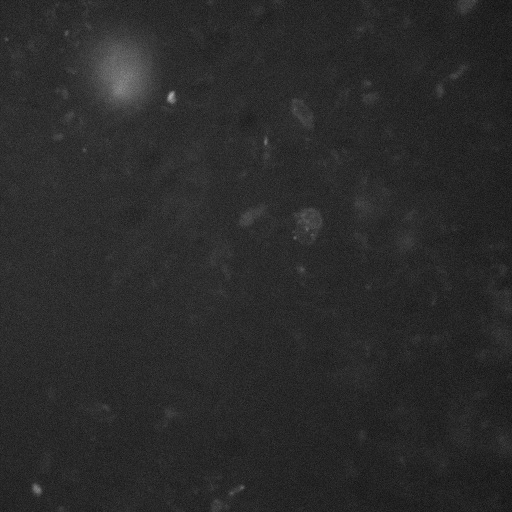

Supplement: S5 File — (ZIP) [file pcbi.1006986.s006.zip › extraitseq5h/KM16_014_5h_20_w1sdcRFP.tif]

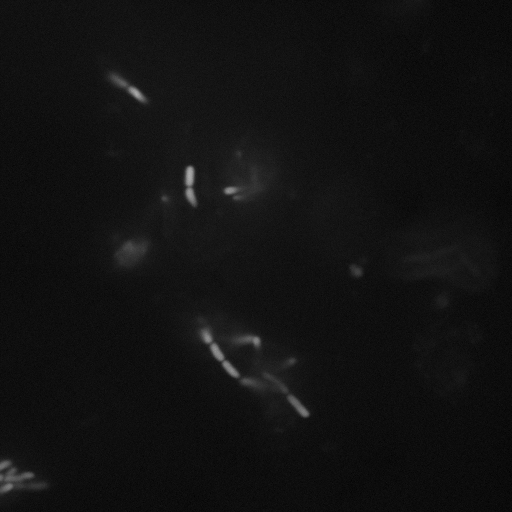

Supplement: S5 File — (ZIP) [file pcbi.1006986.s006.zip › extraitseq5h/KM16_013_5h_8_w2sdcGFP.tif]

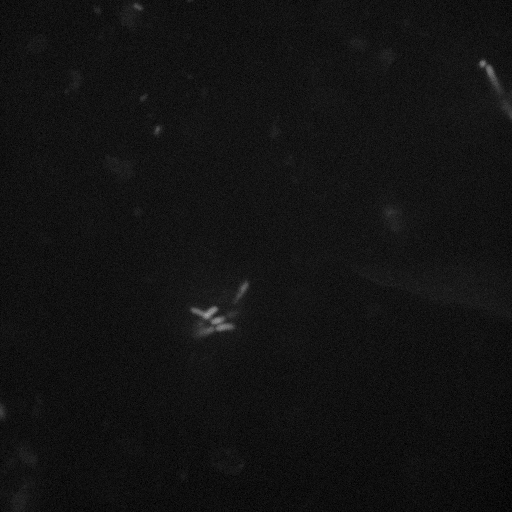

Supplement: S5 File — (ZIP) [file pcbi.1006986.s006.zip › extraitseq5h/KM16_013_5h_22_w1sdcRFP.tif]

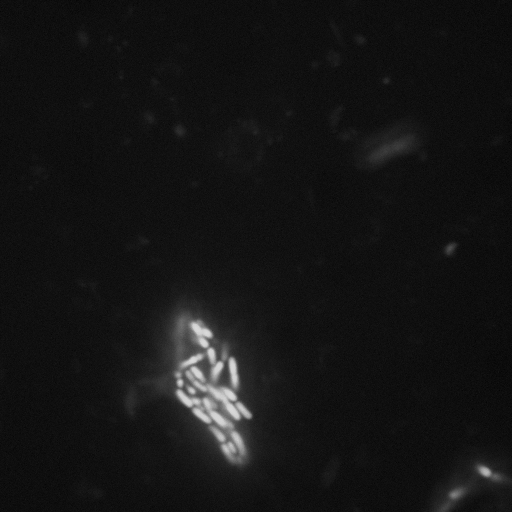

Supplement: S5 File — (ZIP) [file pcbi.1006986.s006.zip › extraitseq5h/KM16_013_5h_32_w2sdcGFP.tif]

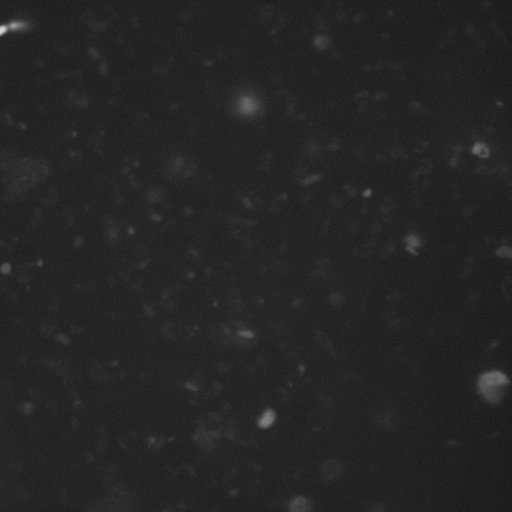

Supplement: S5 File — (ZIP) [file pcbi.1006986.s006.zip › extraitseq5h/KM16_014_5h_19_w2sdcGFP.tif]

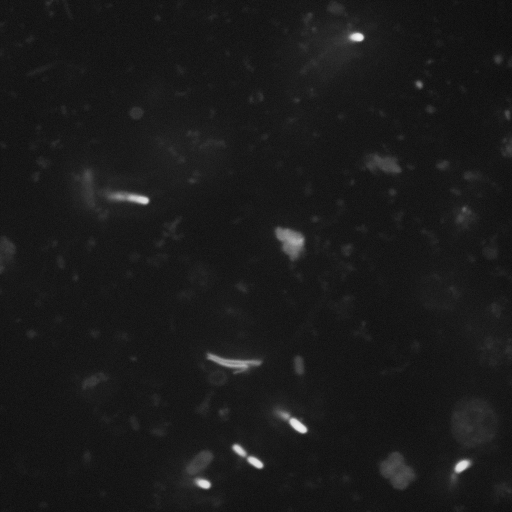

Supplement: S5 File — (ZIP) [file pcbi.1006986.s006.zip › extraitseq5h/KM16_013_5h_40_w2sdcGFP.tif]

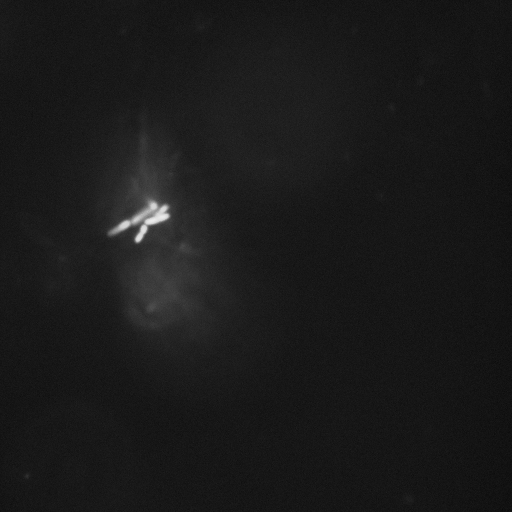

Supplement: S5 File — (ZIP) [file pcbi.1006986.s006.zip › extraitseq5h/KM16_013_5h_15_w2sdcGFP.tif]

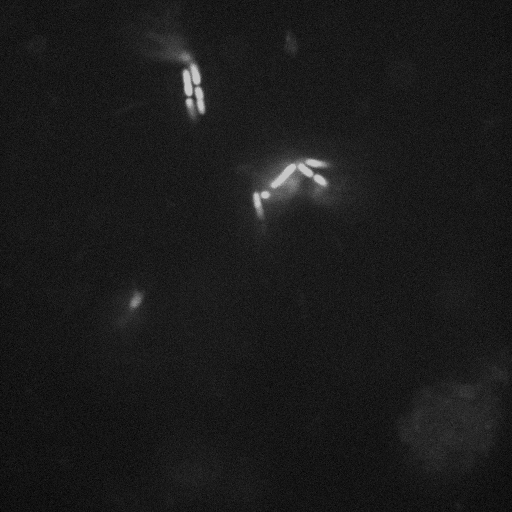

Supplement: S5 File — (ZIP) [file pcbi.1006986.s006.zip › extraitseq5h/KM16_013_5h_36_w1sdcRFP.tif]

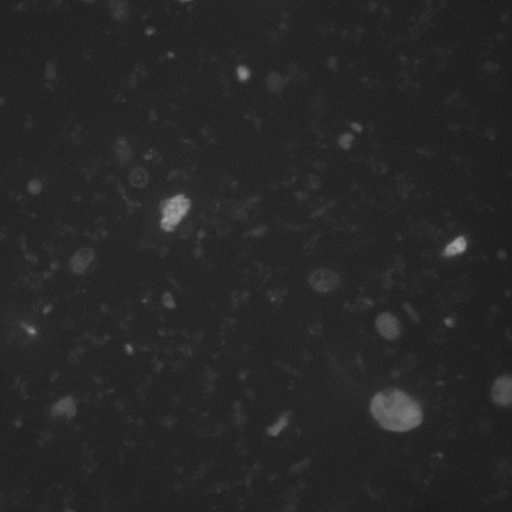

Supplement: S5 File — (ZIP) [file pcbi.1006986.s006.zip › extraitseq5h/KM16_014_5h_17_w2sdcGFP.tif]

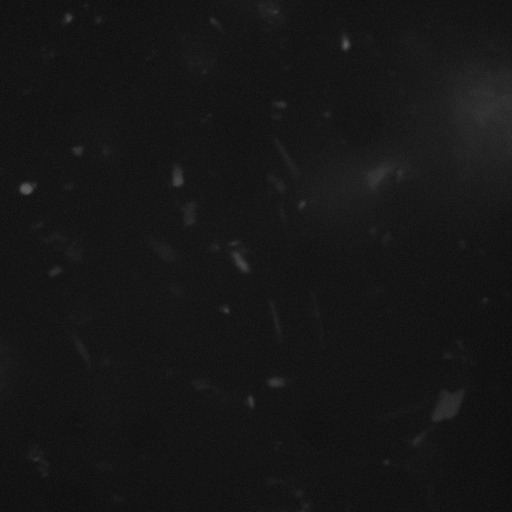

Supplement: S5 File — (ZIP) [file pcbi.1006986.s006.zip › extraitseq5h/KM16_013_5h_2_w2sdcGFP.tif]

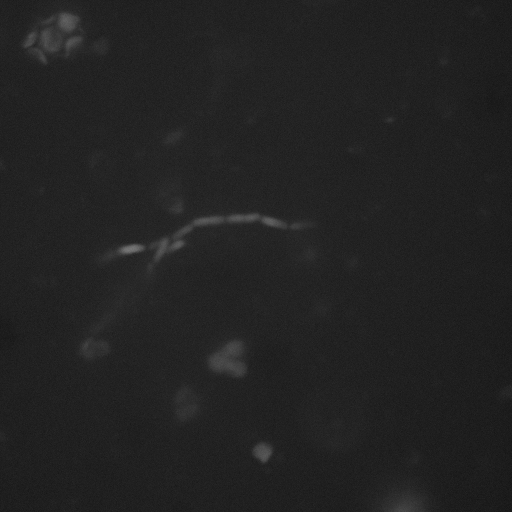

Supplement: S5 File — (ZIP) [file pcbi.1006986.s006.zip › extraitseq5h/KM16_014_5h_2_w2sdcGFP.tif]

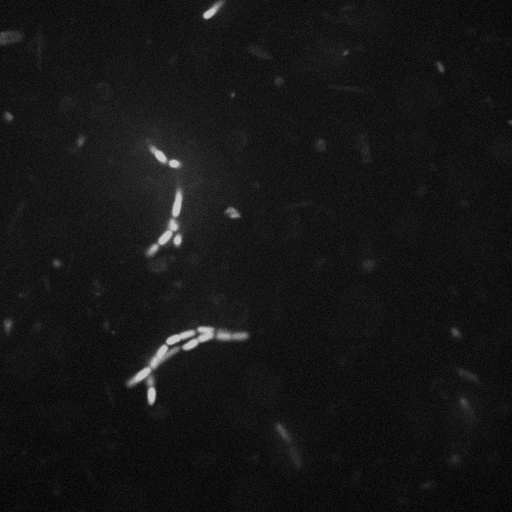

Supplement: S5 File — (ZIP) [file pcbi.1006986.s006.zip › extraitseq5h/KM16_013_5h_30_w1sdcRFP.tif]

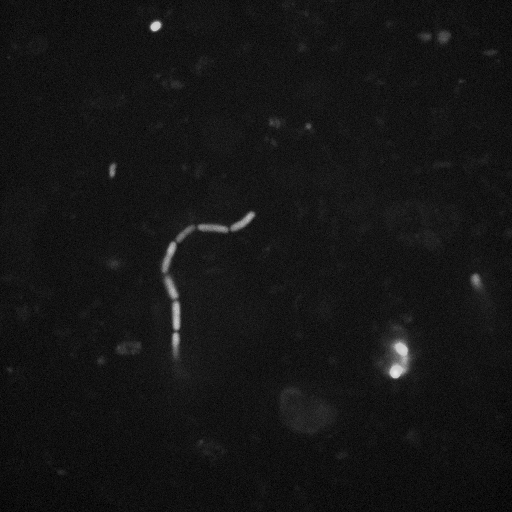

Supplement: S5 File — (ZIP) [file pcbi.1006986.s006.zip › extraitseq5h/KM16_013_5h_29_w1sdcRFP.tif]

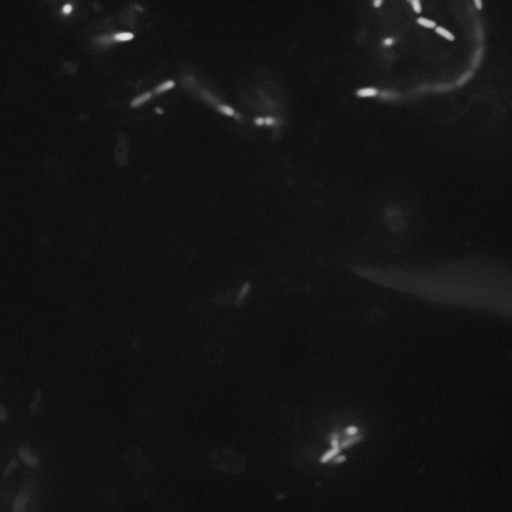

Supplement: S5 File — (ZIP) [file pcbi.1006986.s006.zip › extraitseq5h/KM16_013_5h_21_w2sdcGFP.tif]

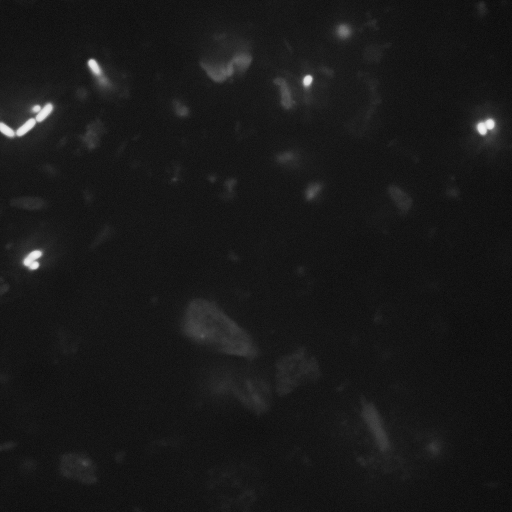

Supplement: S5 File — (ZIP) [file pcbi.1006986.s006.zip › extraitseq5h/KM16_013_5h_19_w2sdcGFP.tif]

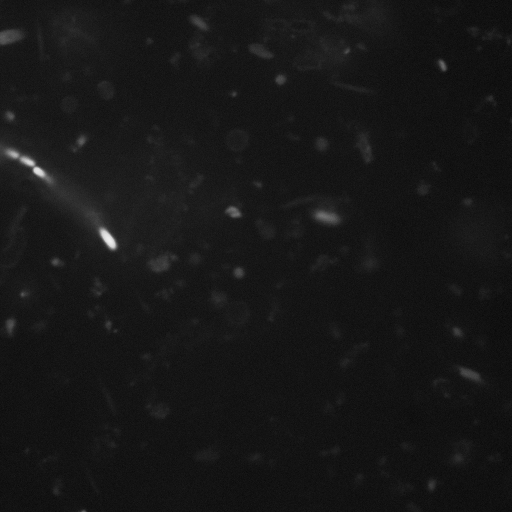

Supplement: S5 File — (ZIP) [file pcbi.1006986.s006.zip › extraitseq5h/KM16_013_5h_30_w2sdcGFP.tif]

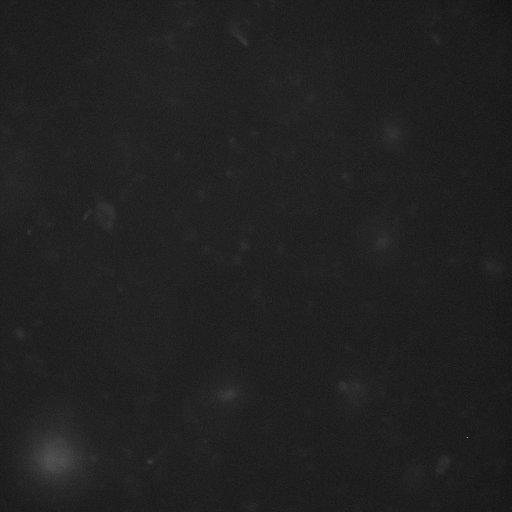

Supplement: S5 File — (ZIP) [file pcbi.1006986.s006.zip › extraitseq5h/KM16_014_5h_25_w2sdcGFP.tif]
