# Supplementary figures and images for: Enchained growth and cluster dislocation: A possible mechanism for microbiota homeostasis (part 7 of 10)
Source: PLoS Comput Biol. 2019 May 3;15(5):e1006986. doi: 10.1371/journal.pcbi.1006986 (PMC6519844; doi:10.1371/journal.pcbi.1006986)

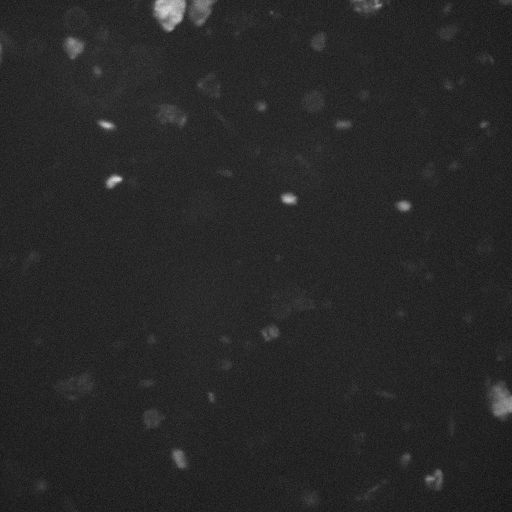

Supplement: S4 File — (ZIP) [file pcbi.1006986.s005.zip › extraitseq4h/KM16_016_4h_PBS_10_w1sdcRFP.tif]

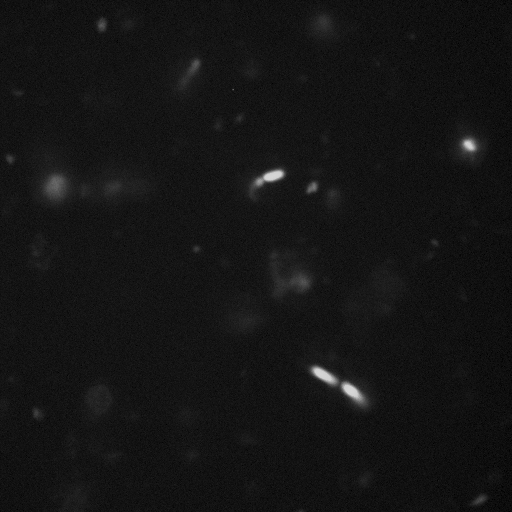

Supplement: S4 File — (ZIP) [file pcbi.1006986.s005.zip › extraitseq4h/KM16_016_4h_Cm_CCCP_20_w2sdcGFP.tif]

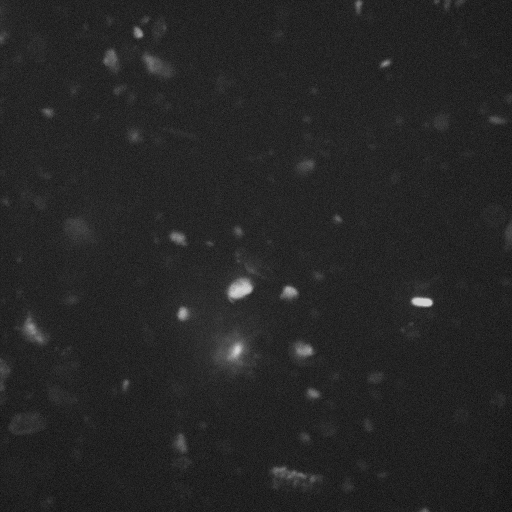

Supplement: S4 File — (ZIP) [file pcbi.1006986.s005.zip › extraitseq4h/KM16_016_4h_PBS_24_w1sdcRFP.tif]

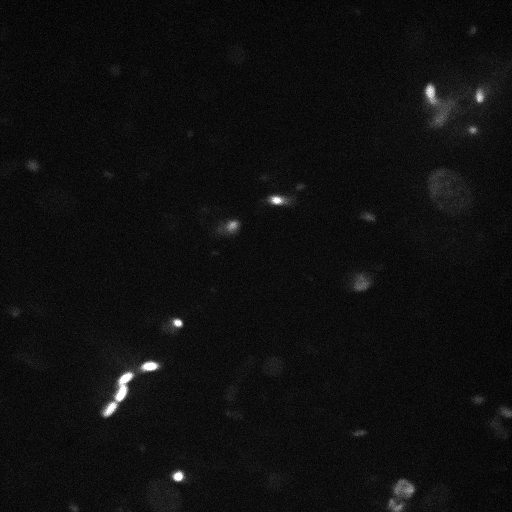

Supplement: S4 File — (ZIP) [file pcbi.1006986.s005.zip › extraitseq4h/KM16_016_4h_PBS_25_w2sdcGFP.tif]

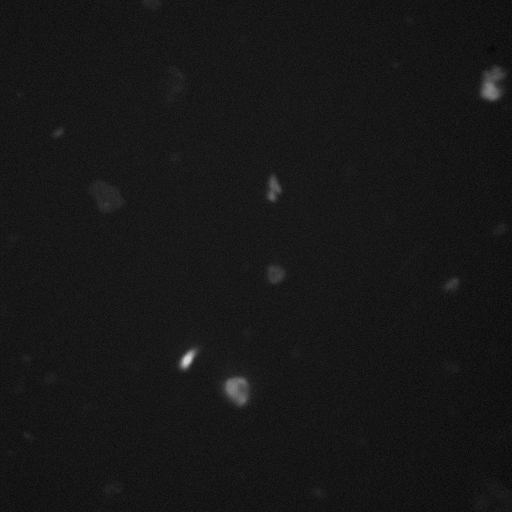

Supplement: S4 File — (ZIP) [file pcbi.1006986.s005.zip › extraitseq4h/KM16_016_4h_Cm_CCCP_3_w2sdcGFP.tif]

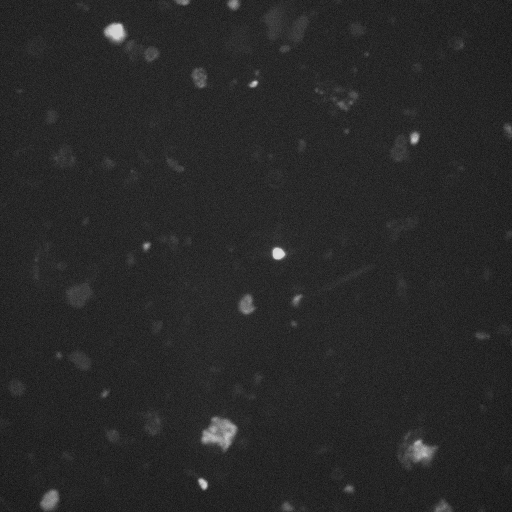

Supplement: S4 File — (ZIP) [file pcbi.1006986.s005.zip › extraitseq4h/KM16_016_4h_Cm_CCCP_19_w1sdcRFP.tif]

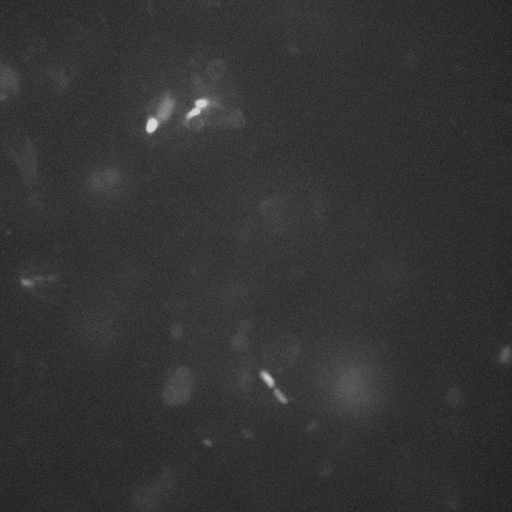

Supplement: S4 File — (ZIP) [file pcbi.1006986.s005.zip › extraitseq4h/KM16_013_4h_34_w1sdcRFP.tif]

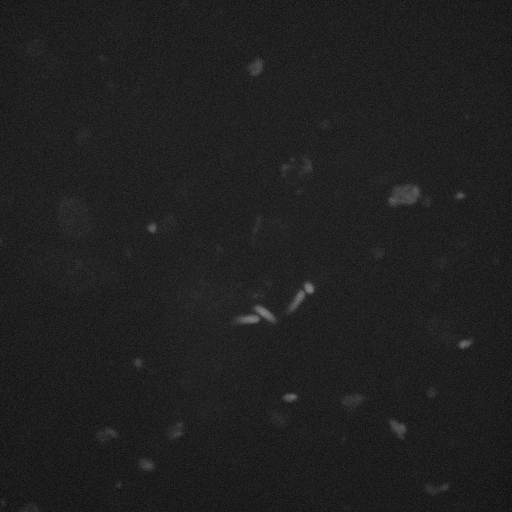

Supplement: S4 File — (ZIP) [file pcbi.1006986.s005.zip › extraitseq4h/KM16_016_4h_PBS_26_w1sdcRFP.tif]

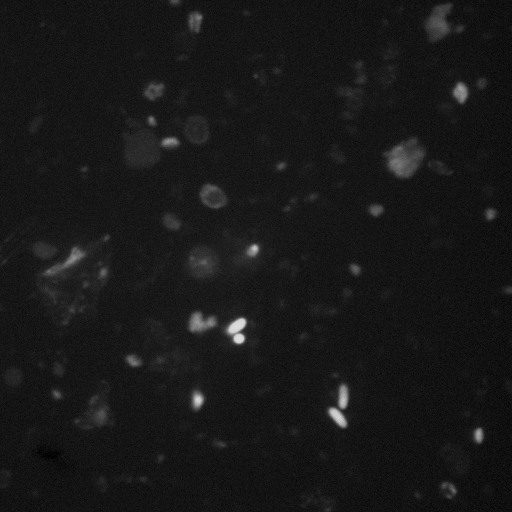

Supplement: S4 File — (ZIP) [file pcbi.1006986.s005.zip › extraitseq4h/KM16_016_4h_Cm_CCCP_24_w2sdcGFP.tif]

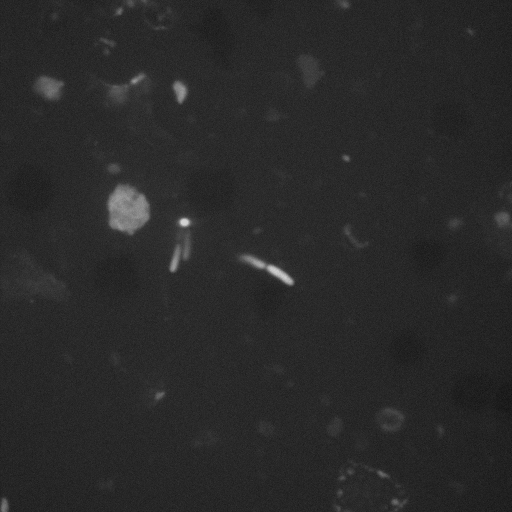

Supplement: S4 File — (ZIP) [file pcbi.1006986.s005.zip › extraitseq4h/KM16_013_4h_3_w2sdcGFP.tif]

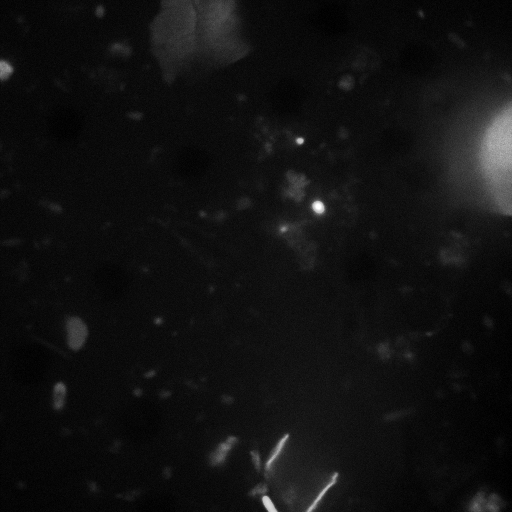

Supplement: S4 File — (ZIP) [file pcbi.1006986.s005.zip › extraitseq4h/KM16_013_4h_5_w2sdcGFP.tif]

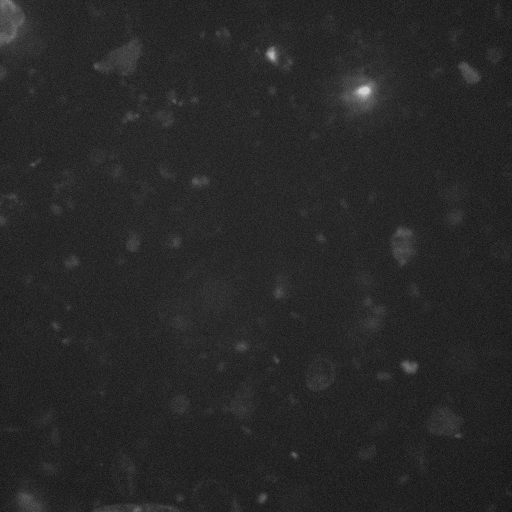

Supplement: S4 File — (ZIP) [file pcbi.1006986.s005.zip › extraitseq4h/KM16_016_4h_PBS_20_w1sdcRFP.tif]

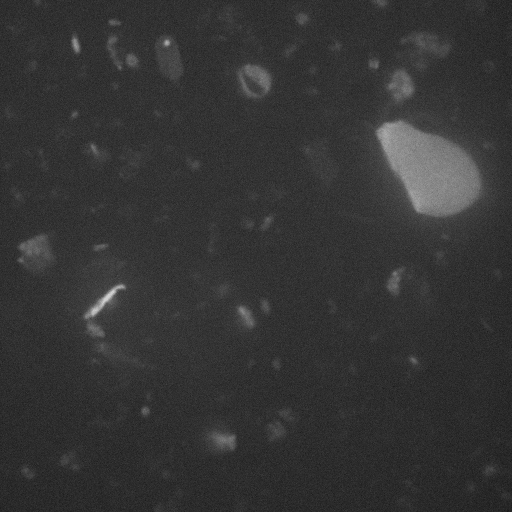

Supplement: S4 File — (ZIP) [file pcbi.1006986.s005.zip › extraitseq4h/KM16_013_4h_4_w1sdcRFP.tif]

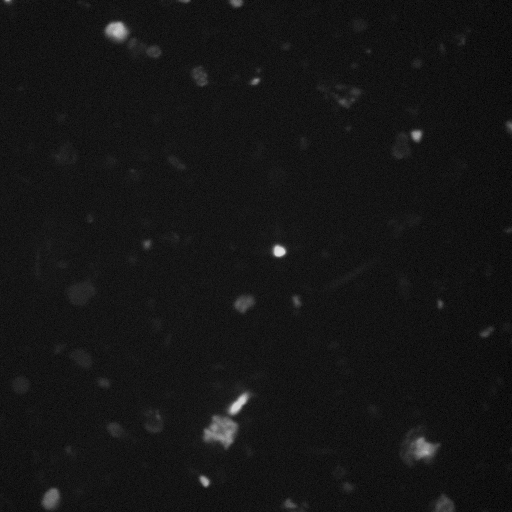

Supplement: S4 File — (ZIP) [file pcbi.1006986.s005.zip › extraitseq4h/KM16_016_4h_Cm_CCCP_19_w2sdcGFP.tif]

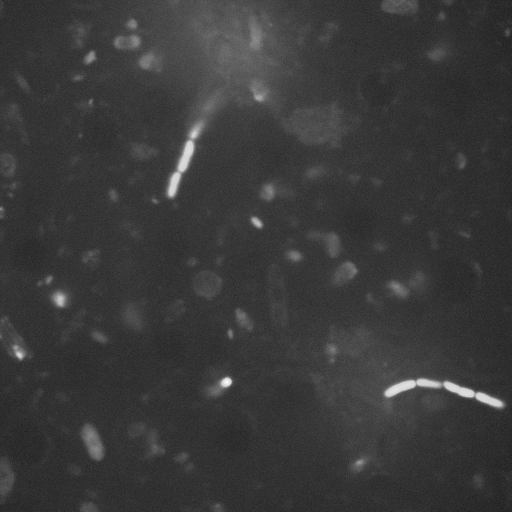

Supplement: S4 File — (ZIP) [file pcbi.1006986.s005.zip › extraitseq4h/KM16_013_4h_30_w2sdcGFP.tif]

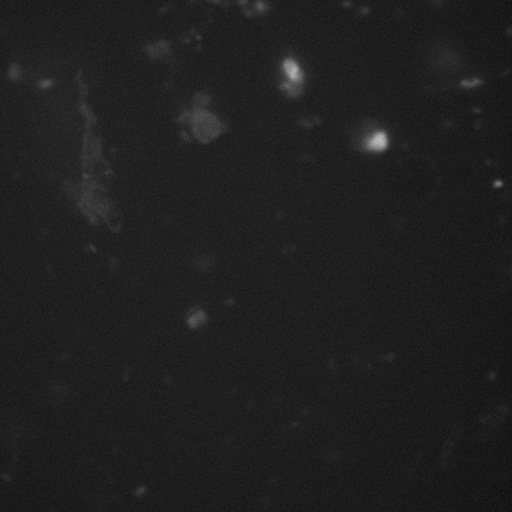

Supplement: S4 File — (ZIP) [file pcbi.1006986.s005.zip › extraitseq4h/KM16_016_4h_Cm_CCCP_10_w2sdcGFP.tif]

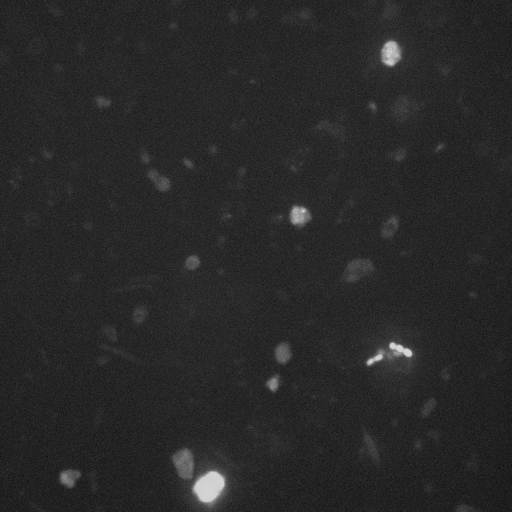

Supplement: S4 File — (ZIP) [file pcbi.1006986.s005.zip › extraitseq4h/KM16_013_4h_12_w1sdcRFP.tif]

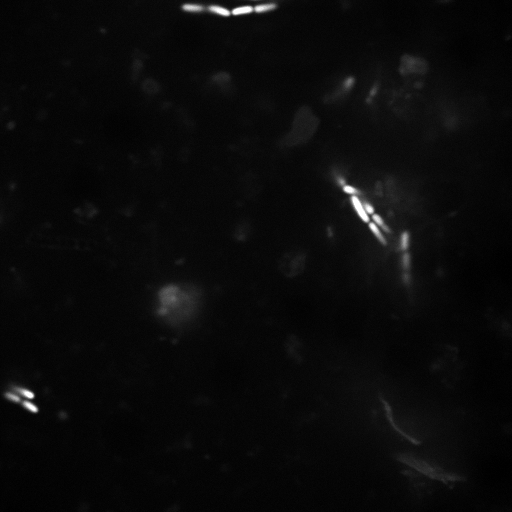

Supplement: S4 File — (ZIP) [file pcbi.1006986.s005.zip › extraitseq4h/KM16_013_4h_22_w2sdcGFP.tif]

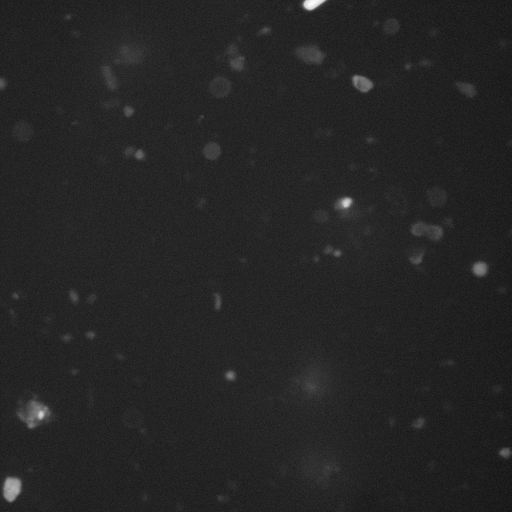

Supplement: S4 File — (ZIP) [file pcbi.1006986.s005.zip › extraitseq4h/KM16_016_4h_PBS_12_w2sdcGFP.tif]

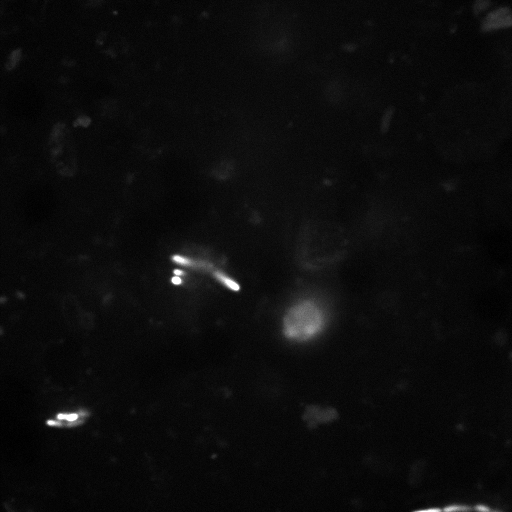

Supplement: S4 File — (ZIP) [file pcbi.1006986.s005.zip › extraitseq4h/KM16_013_4h_26_w2sdcGFP.tif]

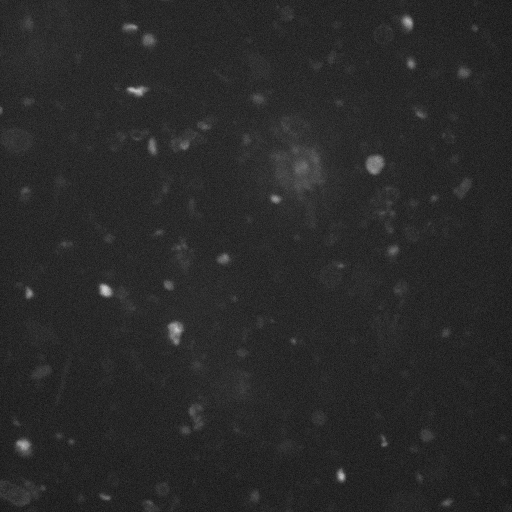

Supplement: S4 File — (ZIP) [file pcbi.1006986.s005.zip › extraitseq4h/KM16_016_4h_PBS_22_w1sdcRFP.tif]

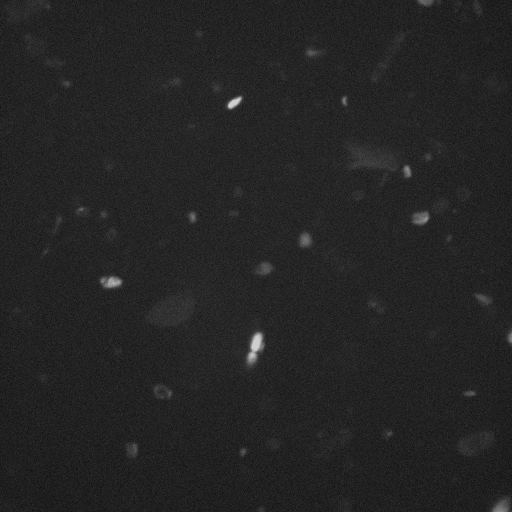

Supplement: S4 File — (ZIP) [file pcbi.1006986.s005.zip › extraitseq4h/KM16_016_4h_Cm_CCCP_15_w1sdcRFP.tif]

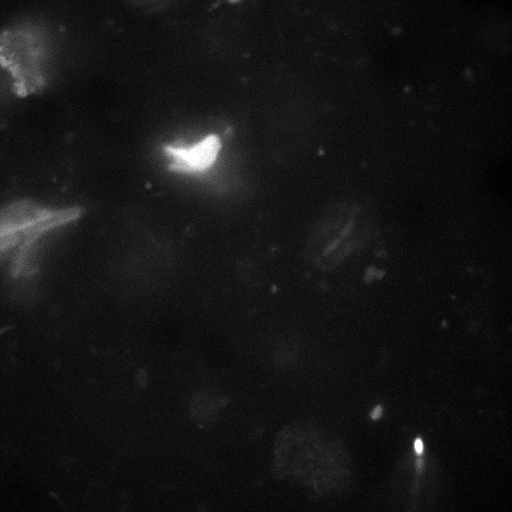

Supplement: S4 File — (ZIP) [file pcbi.1006986.s005.zip › extraitseq4h/KM16_013_4h_25_w2sdcGFP.tif]

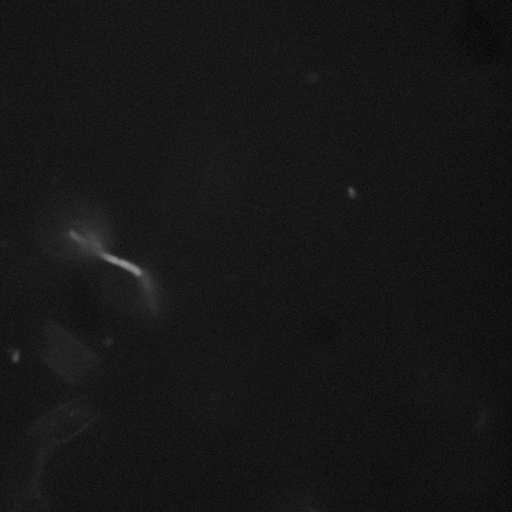

Supplement: S4 File — (ZIP) [file pcbi.1006986.s005.zip › extraitseq4h/KM16_016_4h_PBS_4_w2sdcGFP.tif]

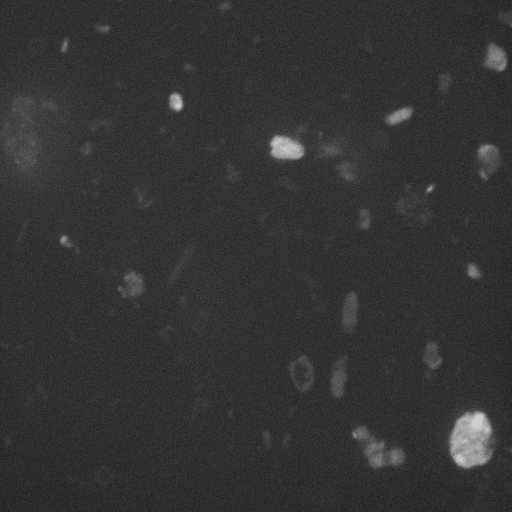

Supplement: S4 File — (ZIP) [file pcbi.1006986.s005.zip › extraitseq4h/KM16_013_4h_2_w1sdcRFP.tif]

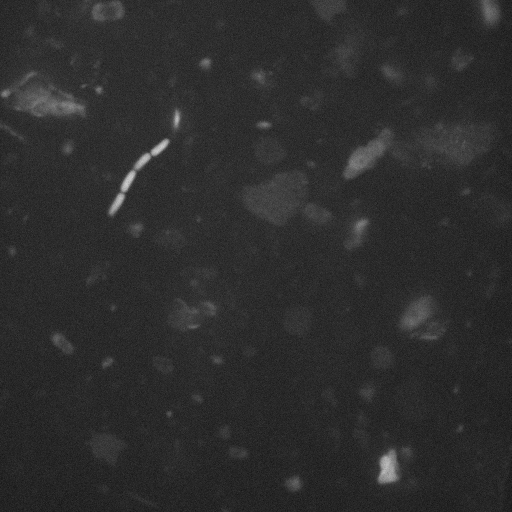

Supplement: S4 File — (ZIP) [file pcbi.1006986.s005.zip › extraitseq4h/KM16_013_4h_24_w1sdcRFP.tif]

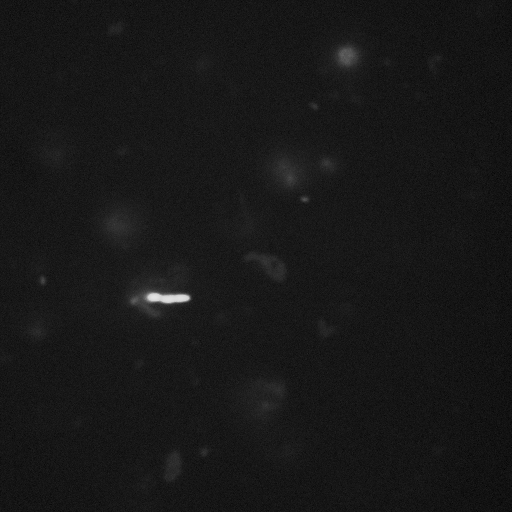

Supplement: S4 File — (ZIP) [file pcbi.1006986.s005.zip › extraitseq4h/KM16_016_4h_PBS_9_w2sdcGFP.tif]

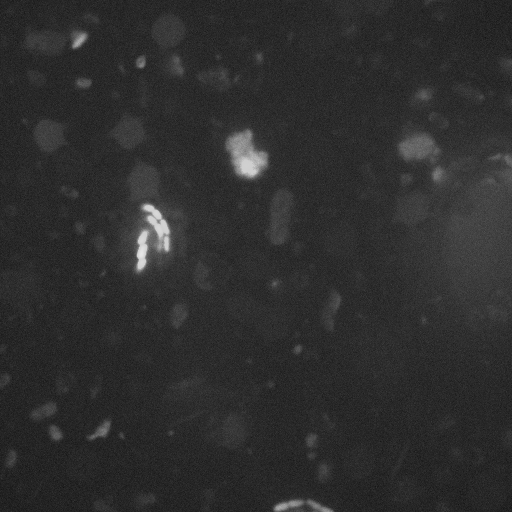

Supplement: S4 File — (ZIP) [file pcbi.1006986.s005.zip › extraitseq4h/KM16_013_4h_31_w1sdcRFP.tif]

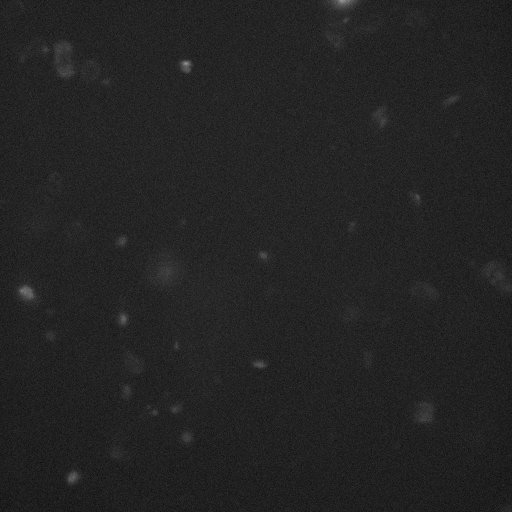

Supplement: S4 File — (ZIP) [file pcbi.1006986.s005.zip › extraitseq4h/KM16_016_4h_PBS_8_w1sdcRFP.tif]

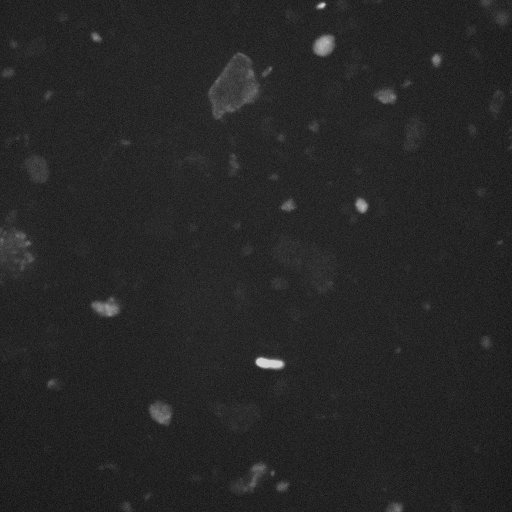

Supplement: S4 File — (ZIP) [file pcbi.1006986.s005.zip › extraitseq4h/KM16_016_4h_Cm_CCCP_18_w1sdcRFP.tif]

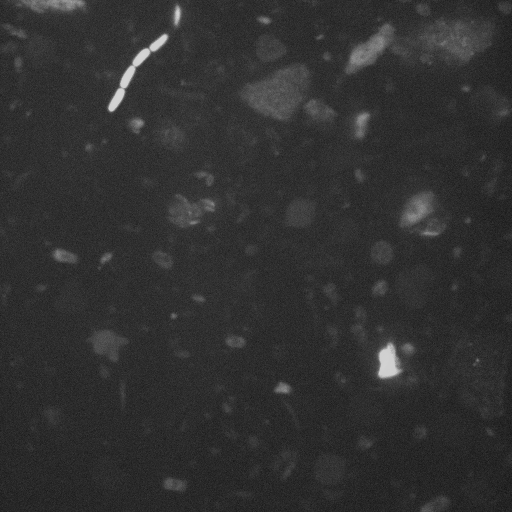

Supplement: S4 File — (ZIP) [file pcbi.1006986.s005.zip › extraitseq4h/KM16_013_4h_23_w1sdcRFP.tif]

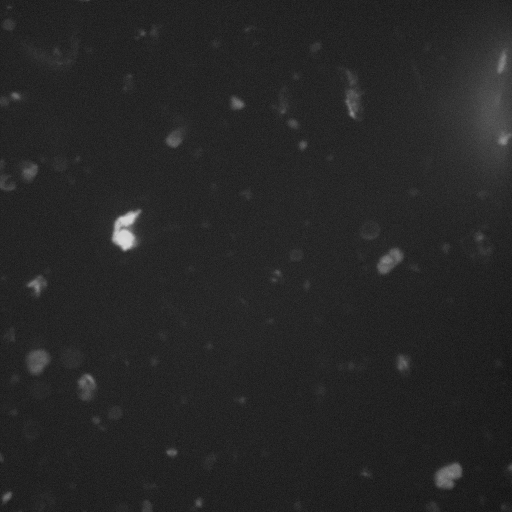

Supplement: S4 File — (ZIP) [file pcbi.1006986.s005.zip › extraitseq4h/KM16_016_4h_Cm_CCCP_7_w2sdcGFP.tif]

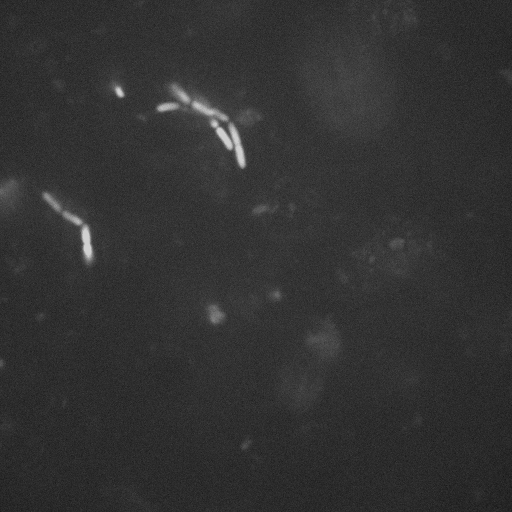

Supplement: S4 File — (ZIP) [file pcbi.1006986.s005.zip › extraitseq4h/KM16_013_4h_17_w1sdcRFP.tif]

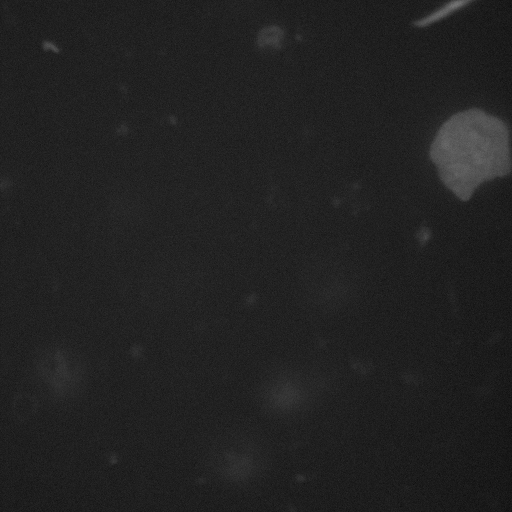

Supplement: S4 File — (ZIP) [file pcbi.1006986.s005.zip › extraitseq4h/KM16_016_4h_PBS_5_w2sdcGFP.tif]

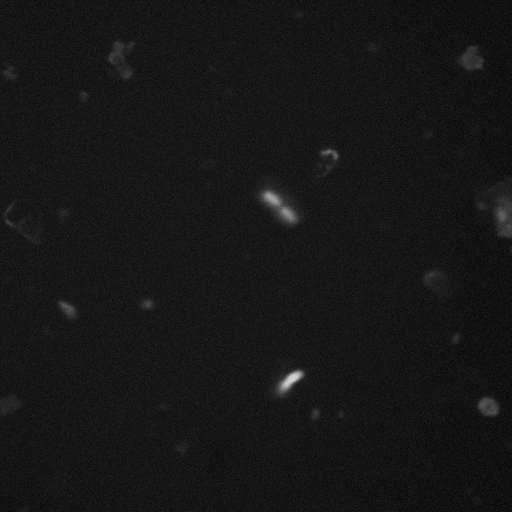

Supplement: S4 File — (ZIP) [file pcbi.1006986.s005.zip › extraitseq4h/KM16_016_4h_Cm_CCCP_8_w2sdcGFP.tif]

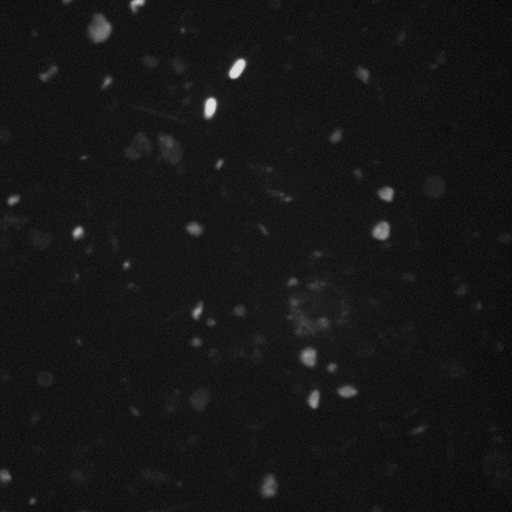

Supplement: S4 File — (ZIP) [file pcbi.1006986.s005.zip › extraitseq4h/KM16_016_4h_Cm_CCCP_23_w2sdcGFP.tif]

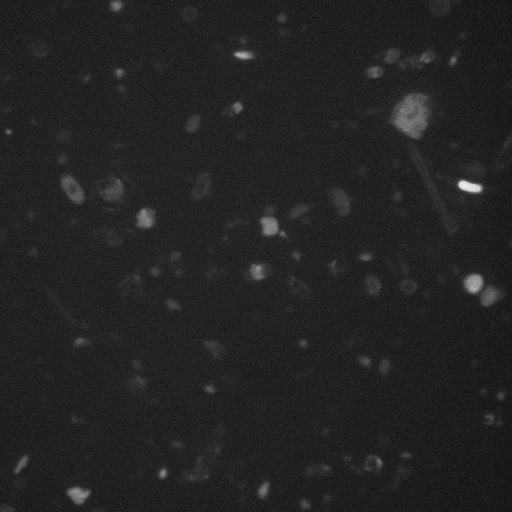

Supplement: S4 File — (ZIP) [file pcbi.1006986.s005.zip › extraitseq4h/KM16_016_4h_Cm_CCCP_22_w2sdcGFP.tif]

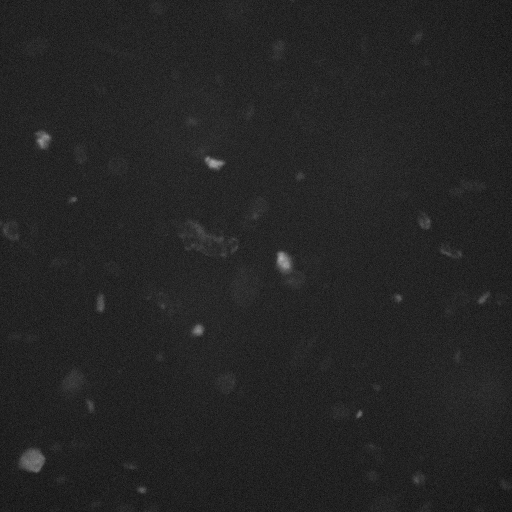

Supplement: S4 File — (ZIP) [file pcbi.1006986.s005.zip › extraitseq4h/KM16_016_4h_Cm_CCCP_13_w1sdcRFP.tif]

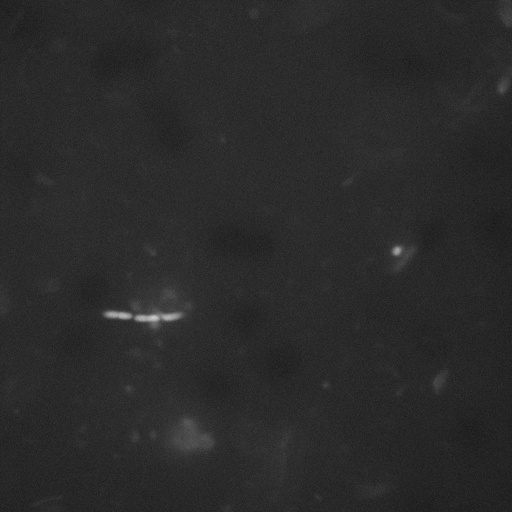

Supplement: S4 File — (ZIP) [file pcbi.1006986.s005.zip › extraitseq4h/KM16_013_4h_19_w2sdcGFP.tif]

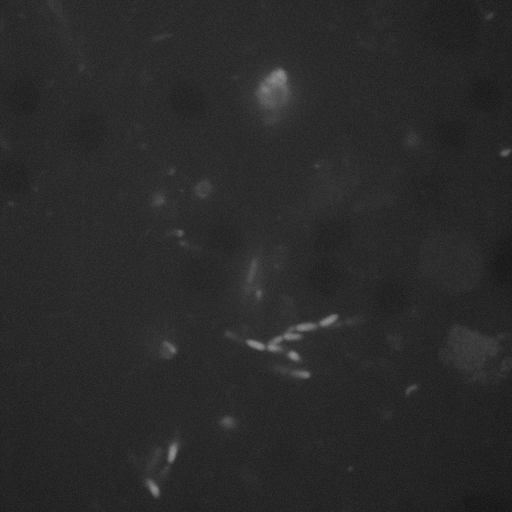

Supplement: S4 File — (ZIP) [file pcbi.1006986.s005.zip › extraitseq4h/KM16_013_4h_18_w2sdcGFP.tif]

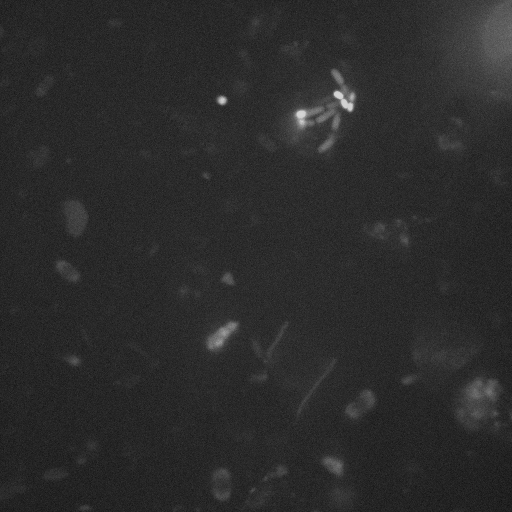

Supplement: S4 File — (ZIP) [file pcbi.1006986.s005.zip › extraitseq4h/KM16_013_4h_6_w1sdcRFP.tif]

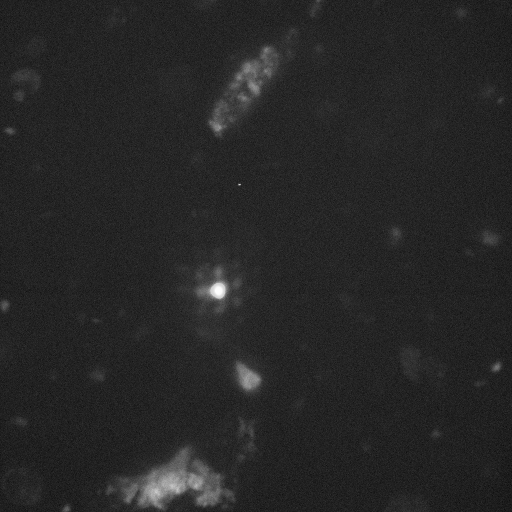

Supplement: S4 File — (ZIP) [file pcbi.1006986.s005.zip › extraitseq4h/KM16_016_4h_Cm_CCCP_17_w1sdcRFP.tif]

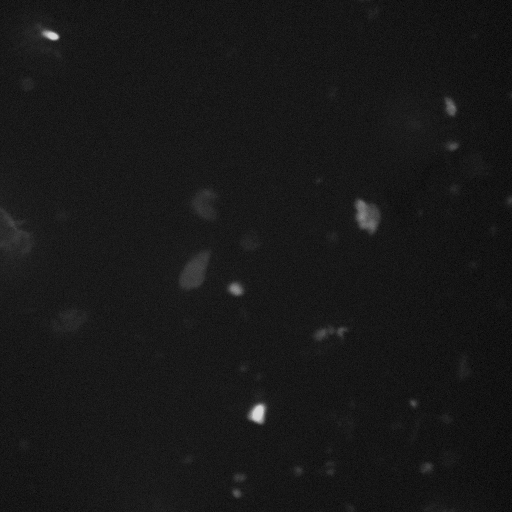

Supplement: S4 File — (ZIP) [file pcbi.1006986.s005.zip › extraitseq4h/KM16_016_4h_Cm_CCCP_5_w2sdcGFP.tif]

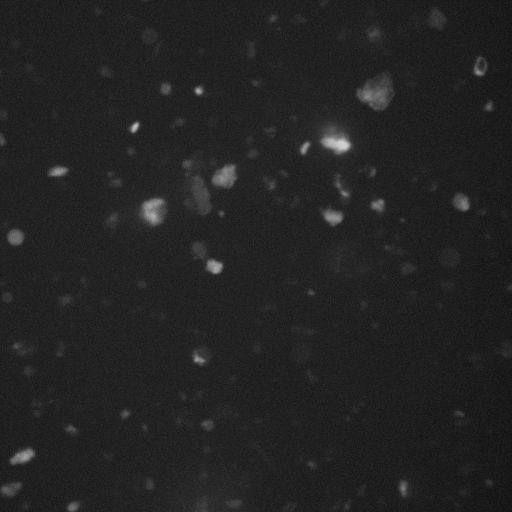

Supplement: S4 File — (ZIP) [file pcbi.1006986.s005.zip › extraitseq4h/KM16_016_4h_Cm_CCCP_21_w2sdcGFP.tif]

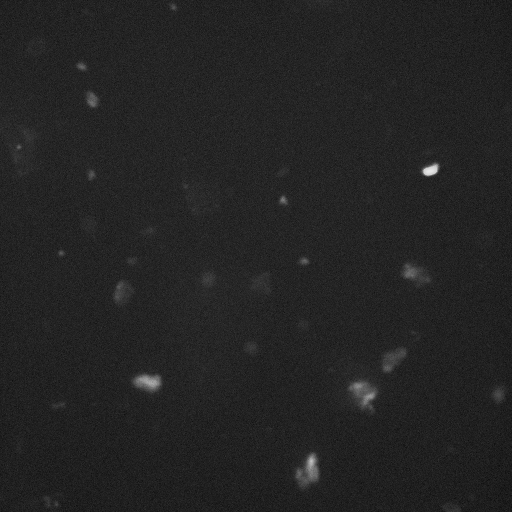

Supplement: S4 File — (ZIP) [file pcbi.1006986.s005.zip › extraitseq4h/KM16_016_4h_PBS_6_w1sdcRFP.tif]

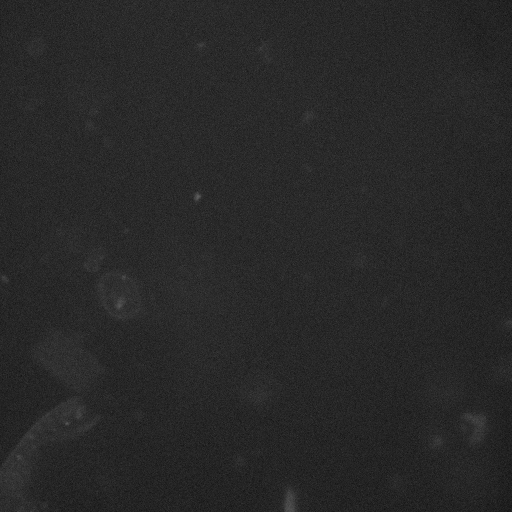

Supplement: S4 File — (ZIP) [file pcbi.1006986.s005.zip › extraitseq4h/KM16_016_4h_PBS_3_w1sdcRFP.tif]

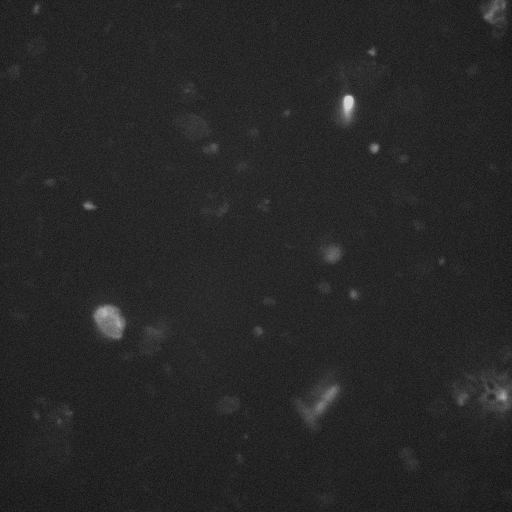

Supplement: S4 File — (ZIP) [file pcbi.1006986.s005.zip › extraitseq4h/KM16_016_4h_Cm_CCCP_14_w1sdcRFP.tif]

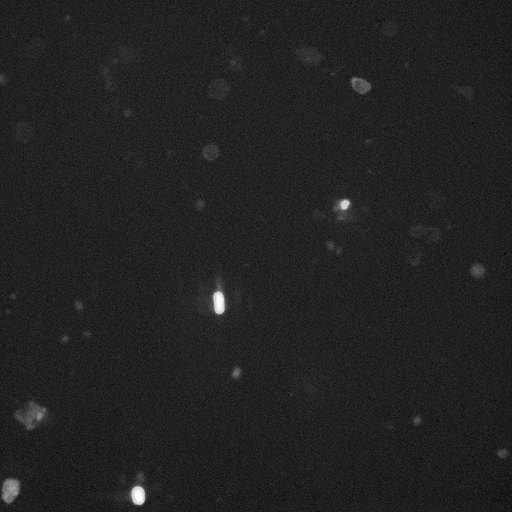

Supplement: S4 File — (ZIP) [file pcbi.1006986.s005.zip › extraitseq4h/KM16_016_4h_PBS_12_w1sdcRFP.tif]

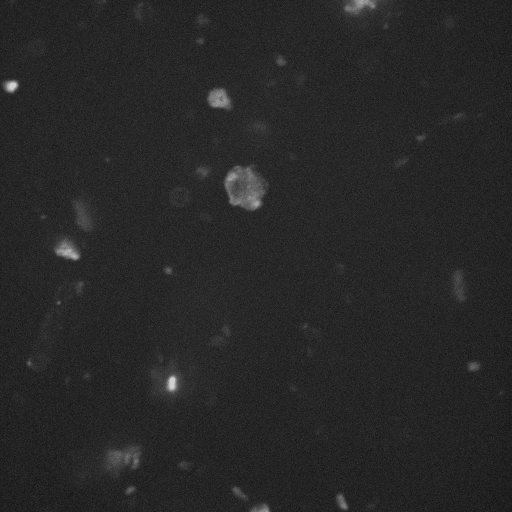

Supplement: S4 File — (ZIP) [file pcbi.1006986.s005.zip › extraitseq4h/KM16_016_4h_PBS_2_w1sdcRFP.tif]

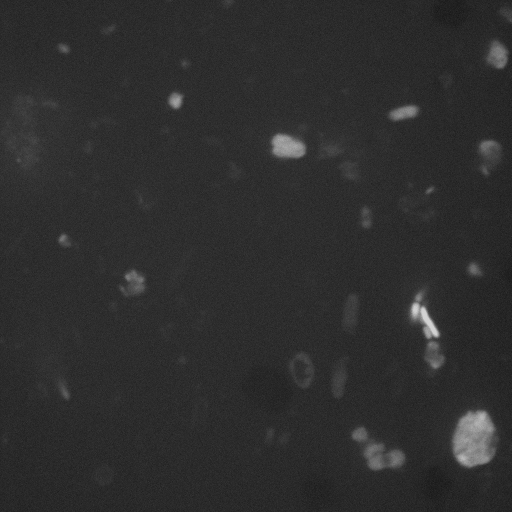

Supplement: S4 File — (ZIP) [file pcbi.1006986.s005.zip › extraitseq4h/KM16_013_4h_2_w2sdcGFP.tif]

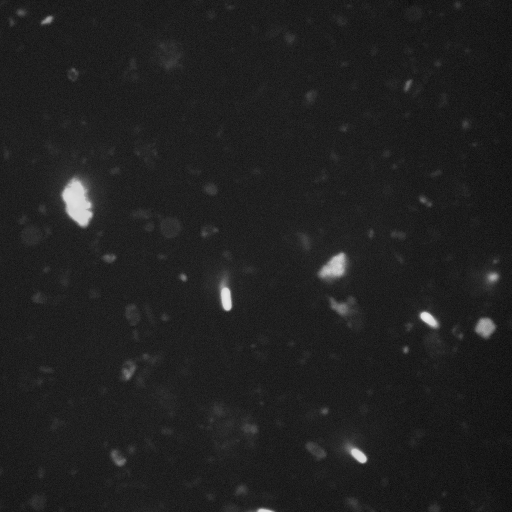

Supplement: S4 File — (ZIP) [file pcbi.1006986.s005.zip › extraitseq4h/KM16_016_4h_PBS_23_w2sdcGFP.tif]

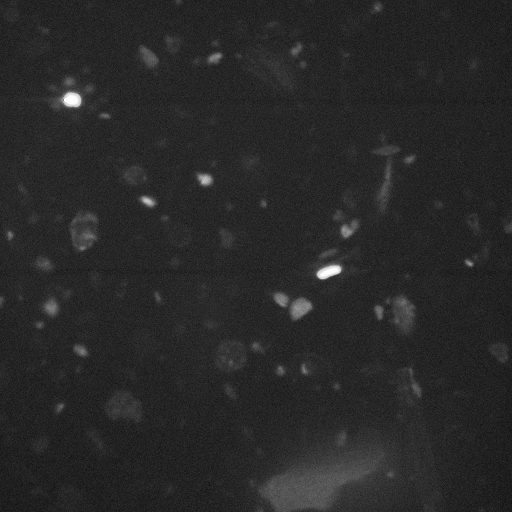

Supplement: S4 File — (ZIP) [file pcbi.1006986.s005.zip › extraitseq4h/KM16_016_4h_PBS_19_w1sdcRFP.tif]

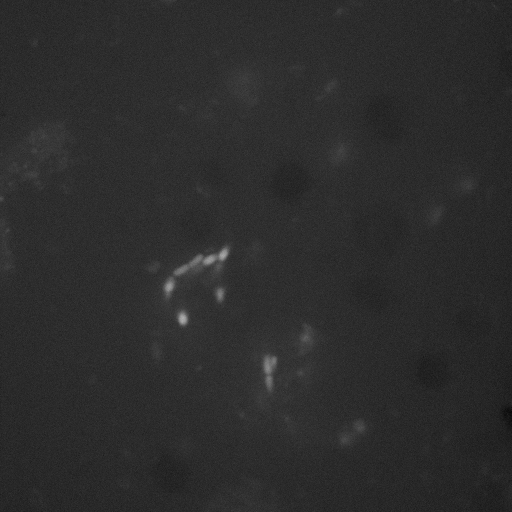

Supplement: S4 File — (ZIP) [file pcbi.1006986.s005.zip › extraitseq4h/KM16_013_4h_1_w2sdcGFP.tif]

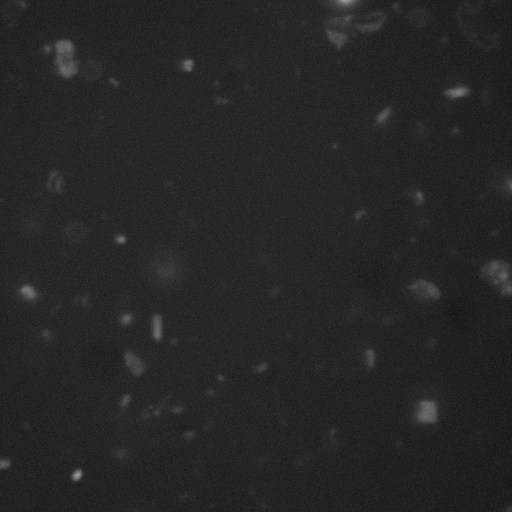

Supplement: S4 File — (ZIP) [file pcbi.1006986.s005.zip › extraitseq4h/KM16_016_4h_PBS_8_w2sdcGFP.tif]

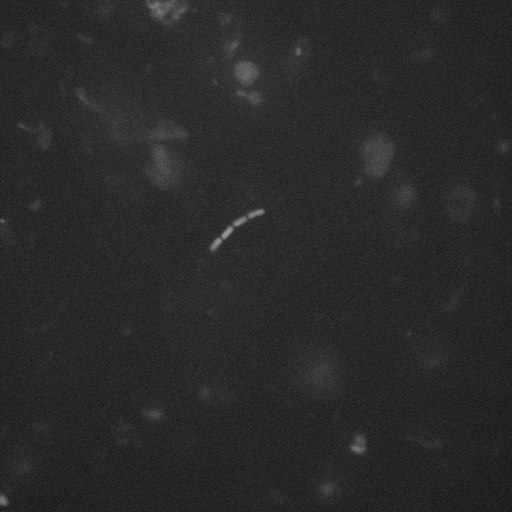

Supplement: S4 File — (ZIP) [file pcbi.1006986.s005.zip › extraitseq4h/KM16_013_4h_11_w1sdcRFP.tif]

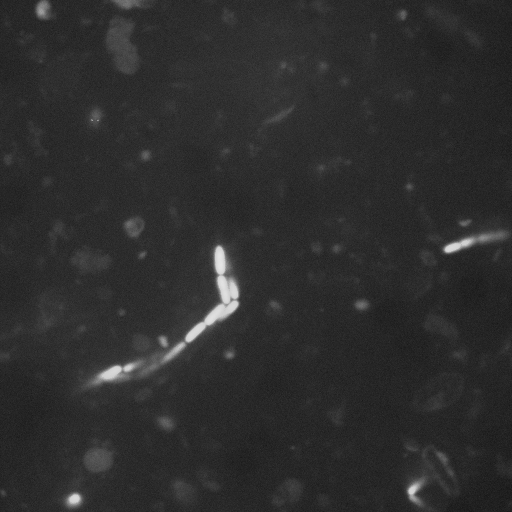

Supplement: S4 File — (ZIP) [file pcbi.1006986.s005.zip › extraitseq4h/KM16_013_4h_29_w2sdcGFP.tif]

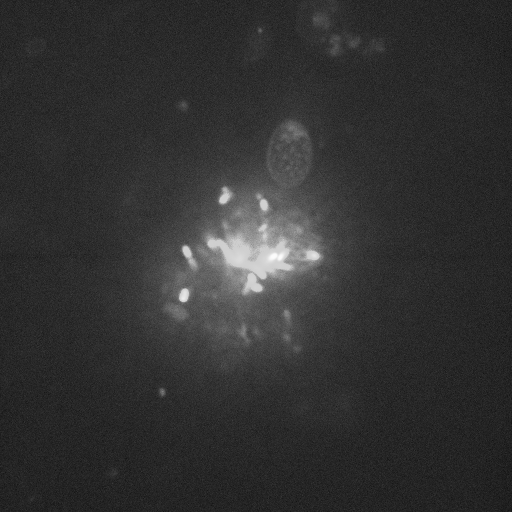

Supplement: S4 File — (ZIP) [file pcbi.1006986.s005.zip › extraitseq4h/KM16_016_4h_PBS_14_w1sdcRFP.tif]

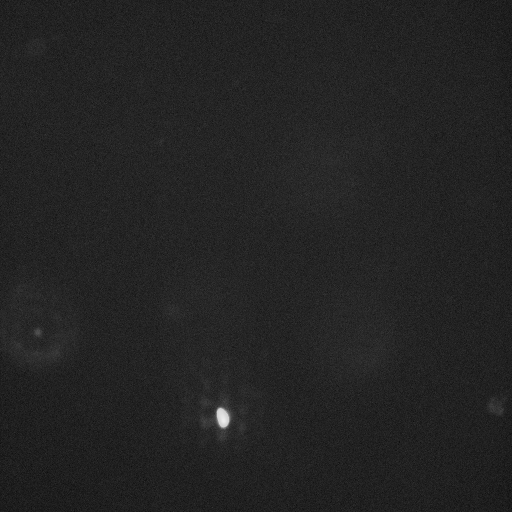

Supplement: S4 File — (ZIP) [file pcbi.1006986.s005.zip › extraitseq4h/KM16_016_4h_Cm_CCCP_16_w1sdcRFP.tif]

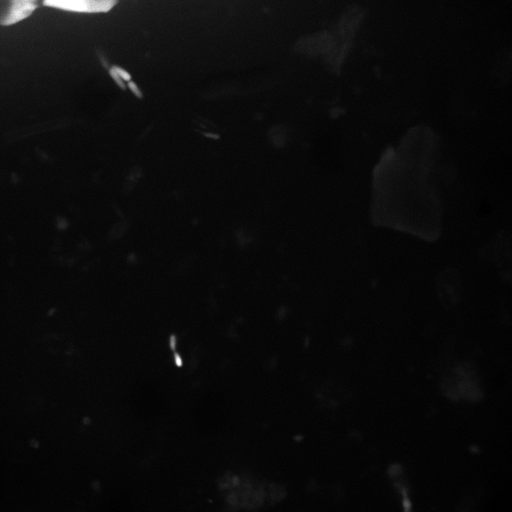

Supplement: S4 File — (ZIP) [file pcbi.1006986.s005.zip › extraitseq4h/KM16_013_4h_7_w2sdcGFP.tif]

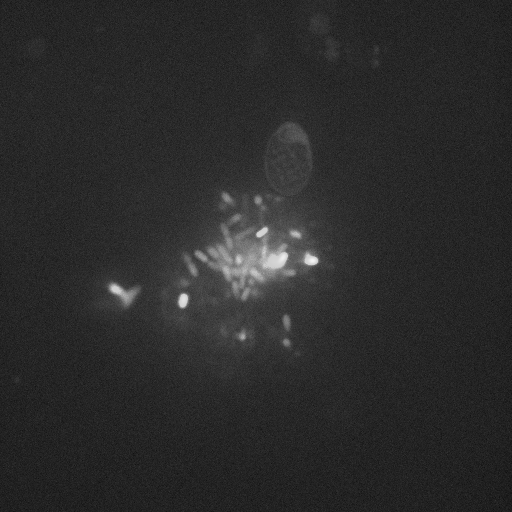

Supplement: S4 File — (ZIP) [file pcbi.1006986.s005.zip › extraitseq4h/KM16_016_4h_PBS_15_w1sdcRFP.tif]

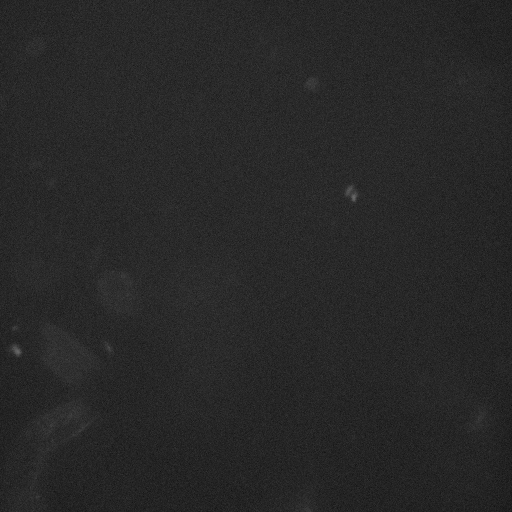

Supplement: S4 File — (ZIP) [file pcbi.1006986.s005.zip › extraitseq4h/KM16_016_4h_PBS_4_w1sdcRFP.tif]

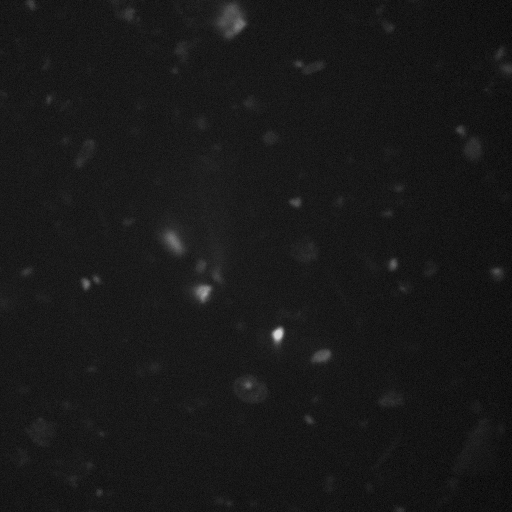

Supplement: S4 File — (ZIP) [file pcbi.1006986.s005.zip › extraitseq4h/KM16_016_4h_PBS_17_w2sdcGFP.tif]

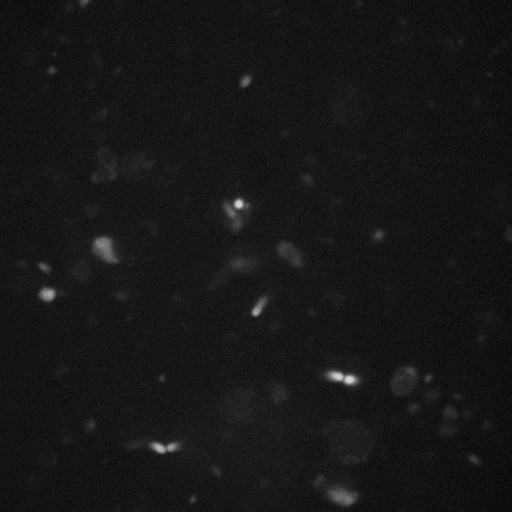

Supplement: S4 File — (ZIP) [file pcbi.1006986.s005.zip › extraitseq4h/KM16_016_4h_PBS_21_w2sdcGFP.tif]

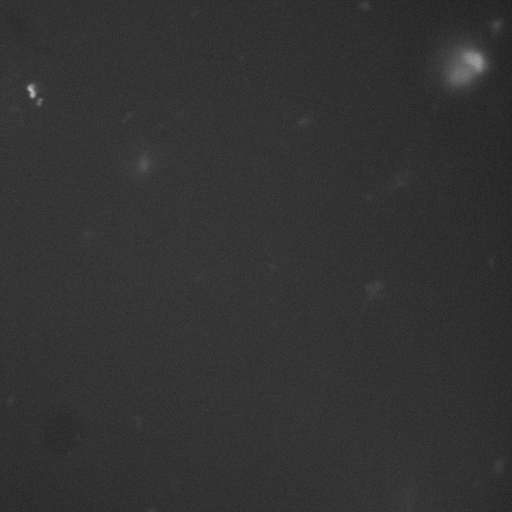

Supplement: S4 File — (ZIP) [file pcbi.1006986.s005.zip › extraitseq4h/KM16_016_4h_PBS_7_w2sdcGFP.tif]

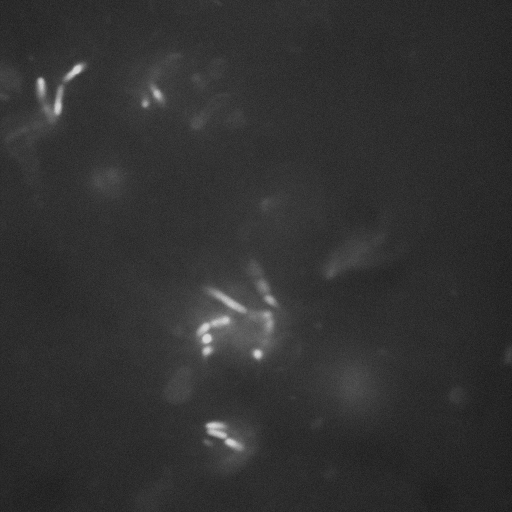

Supplement: S4 File — (ZIP) [file pcbi.1006986.s005.zip › extraitseq4h/KM16_013_4h_34_w2sdcGFP.tif]

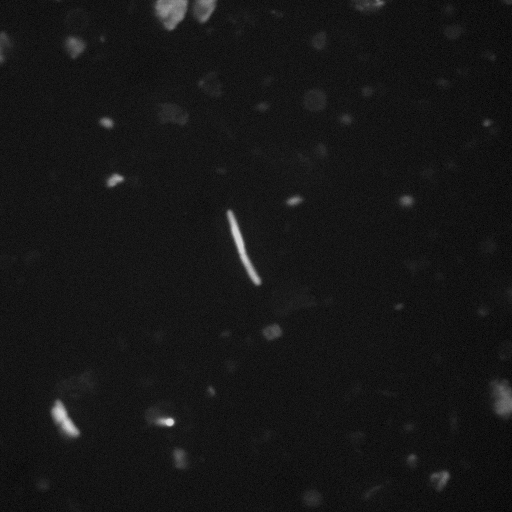

Supplement: S4 File — (ZIP) [file pcbi.1006986.s005.zip › extraitseq4h/KM16_016_4h_PBS_10_w2sdcGFP.tif]

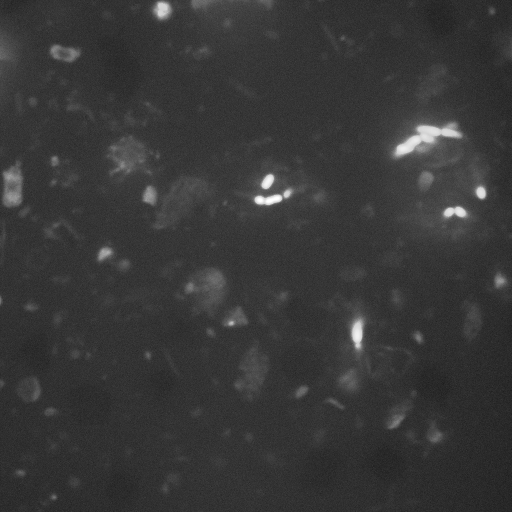

Supplement: S4 File — (ZIP) [file pcbi.1006986.s005.zip › extraitseq4h/KM16_013_4h_16_w2sdcGFP.tif]

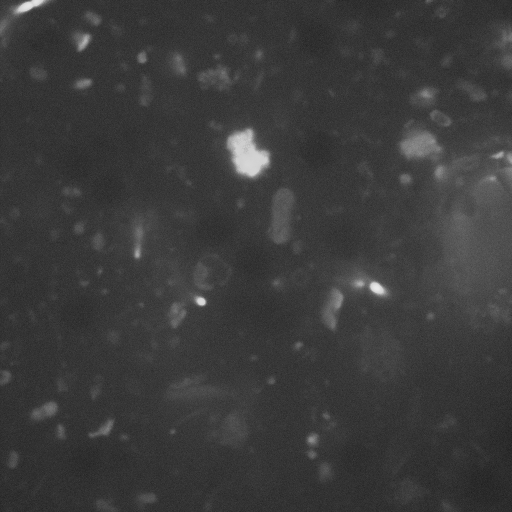

Supplement: S4 File — (ZIP) [file pcbi.1006986.s005.zip › extraitseq4h/KM16_013_4h_31_w2sdcGFP.tif]

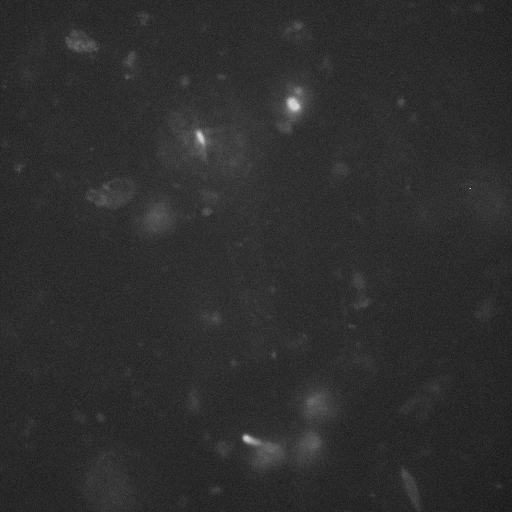

Supplement: S4 File — (ZIP) [file pcbi.1006986.s005.zip › extraitseq4h/KM16_013_4h_15_w1sdcRFP.tif]

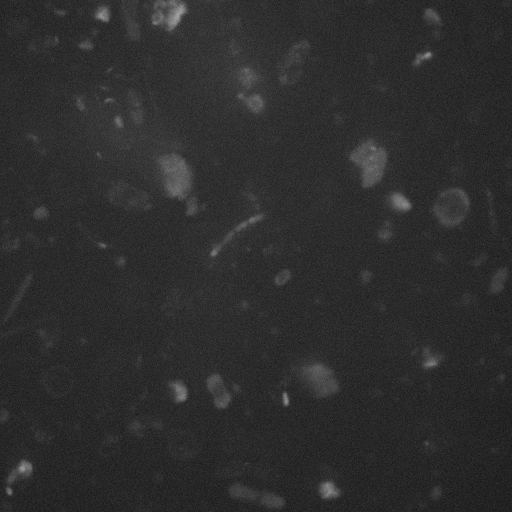

Supplement: S4 File — (ZIP) [file pcbi.1006986.s005.zip › extraitseq4h/KM16_013_4h_10_w1sdcRFP.tif]

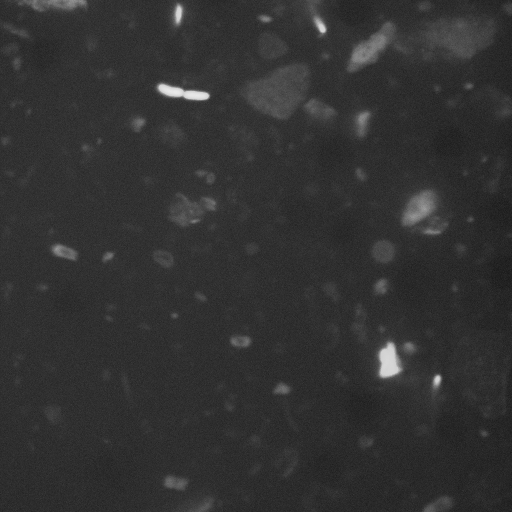

Supplement: S4 File — (ZIP) [file pcbi.1006986.s005.zip › extraitseq4h/KM16_013_4h_23_w2sdcGFP.tif]

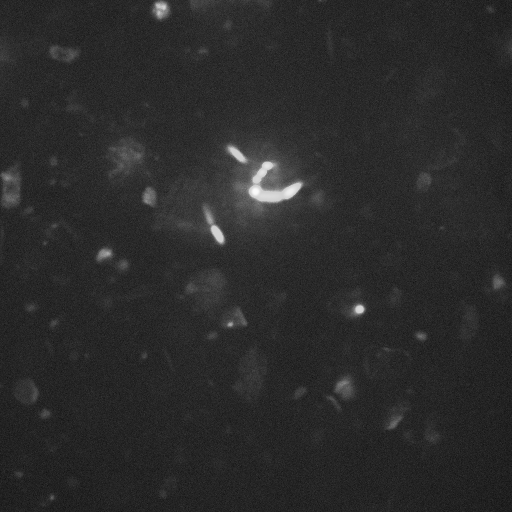

Supplement: S4 File — (ZIP) [file pcbi.1006986.s005.zip › extraitseq4h/KM16_013_4h_16_w1sdcRFP.tif]

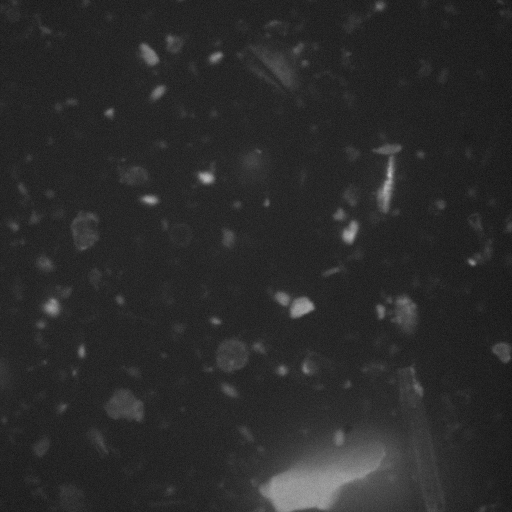

Supplement: S4 File — (ZIP) [file pcbi.1006986.s005.zip › extraitseq4h/KM16_016_4h_PBS_19_w2sdcGFP.tif]

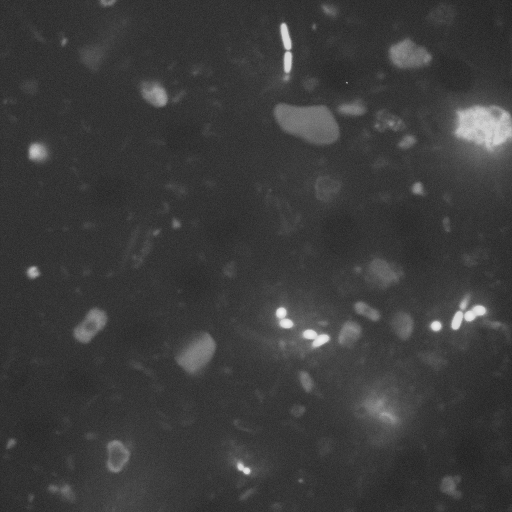

Supplement: S4 File — (ZIP) [file pcbi.1006986.s005.zip › extraitseq4h/KM16_013_4h_20_w2sdcGFP.tif]

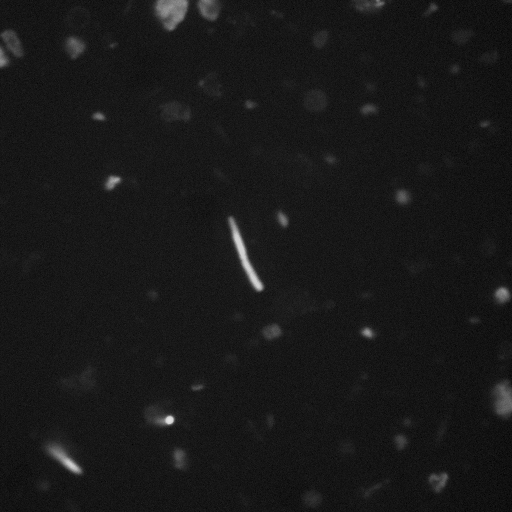

Supplement: S4 File — (ZIP) [file pcbi.1006986.s005.zip › extraitseq4h/KM16_016_4h_PBS_11_w2sdcGFP.tif]

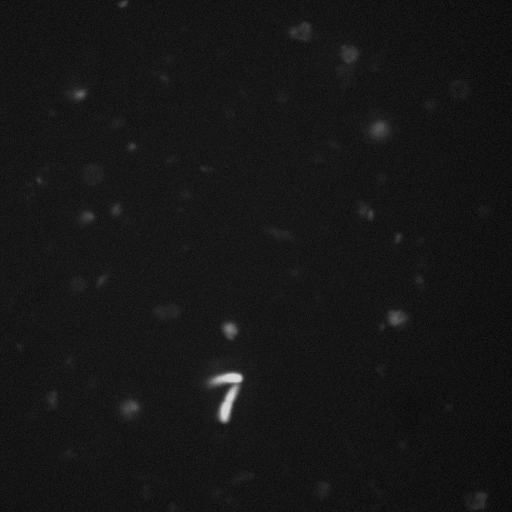

Supplement: S4 File — (ZIP) [file pcbi.1006986.s005.zip › extraitseq4h/KM16_016_4h_Cm_CCCP_9_w2sdcGFP.tif]

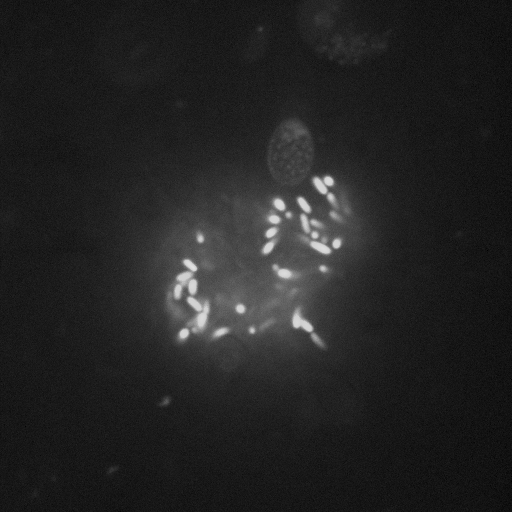

Supplement: S4 File — (ZIP) [file pcbi.1006986.s005.zip › extraitseq4h/KM16_016_4h_PBS_14_w2sdcGFP.tif]

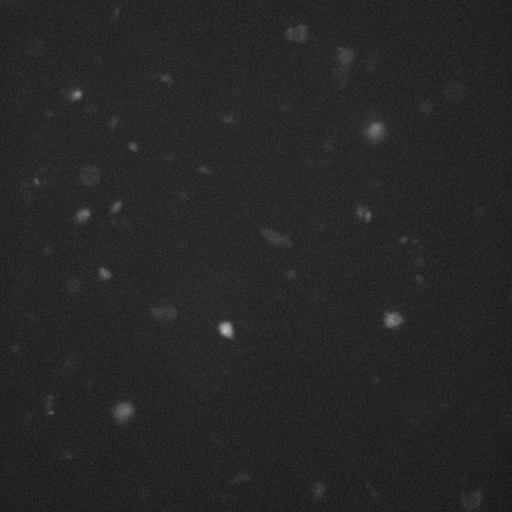

Supplement: S4 File — (ZIP) [file pcbi.1006986.s005.zip › extraitseq4h/KM16_016_4h_Cm_CCCP_9_w1sdcRFP.tif]

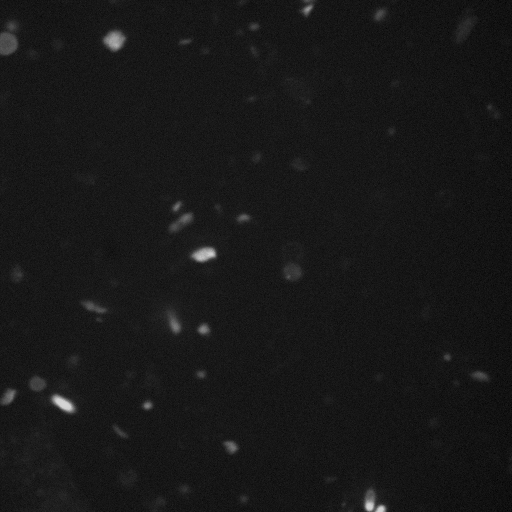

Supplement: S4 File — (ZIP) [file pcbi.1006986.s005.zip › extraitseq4h/KM16_016_4h_PBS_16_w2sdcGFP.tif]

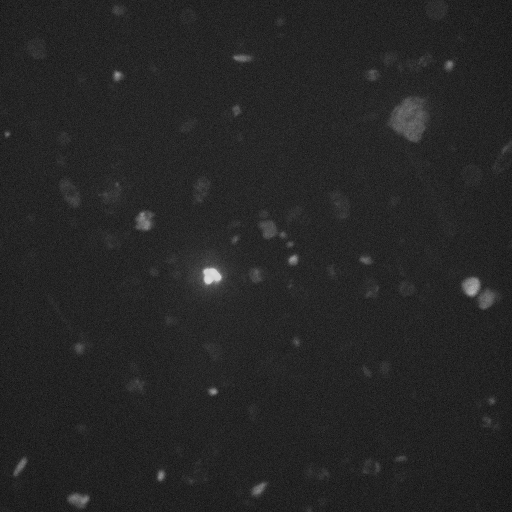

Supplement: S4 File — (ZIP) [file pcbi.1006986.s005.zip › extraitseq4h/KM16_016_4h_Cm_CCCP_22_w1sdcRFP.tif]

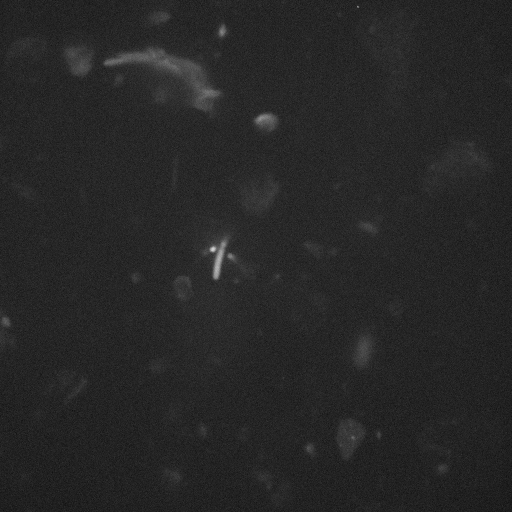

Supplement: S4 File — (ZIP) [file pcbi.1006986.s005.zip › extraitseq4h/KM16_013_4h_9_w1sdcRFP.tif]

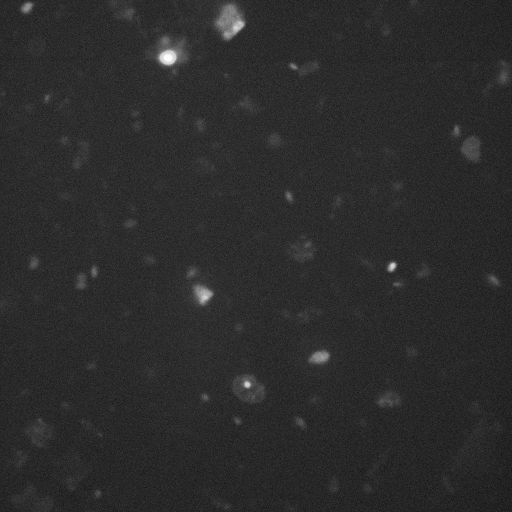

Supplement: S4 File — (ZIP) [file pcbi.1006986.s005.zip › extraitseq4h/KM16_016_4h_PBS_17_w1sdcRFP.tif]

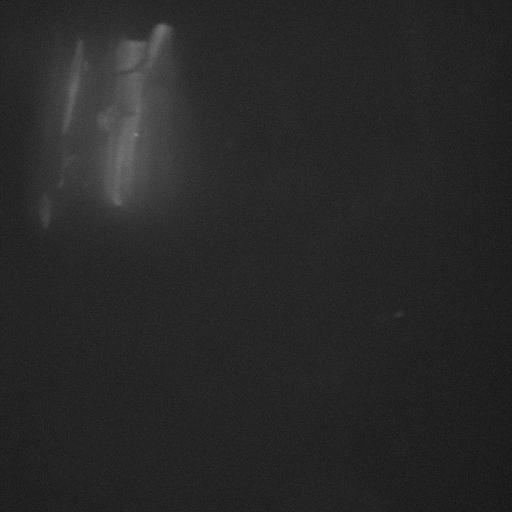

Supplement: S4 File — (ZIP) [file pcbi.1006986.s005.zip › extraitseq4h/KM16_016_4h_Cm_CCCP_11_w2sdcGFP.tif]

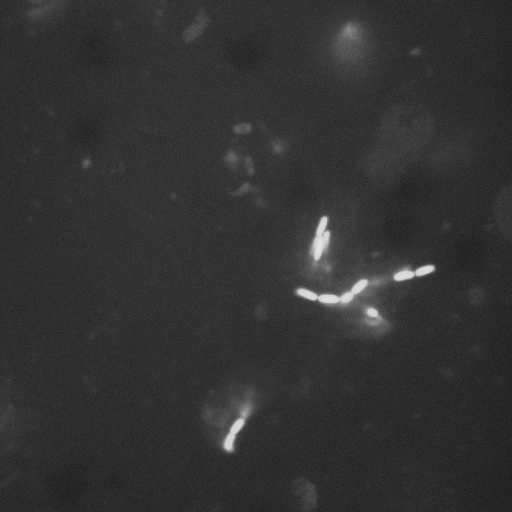

Supplement: S4 File — (ZIP) [file pcbi.1006986.s005.zip › extraitseq4h/KM16_013_4h_14_w2sdcGFP.tif]

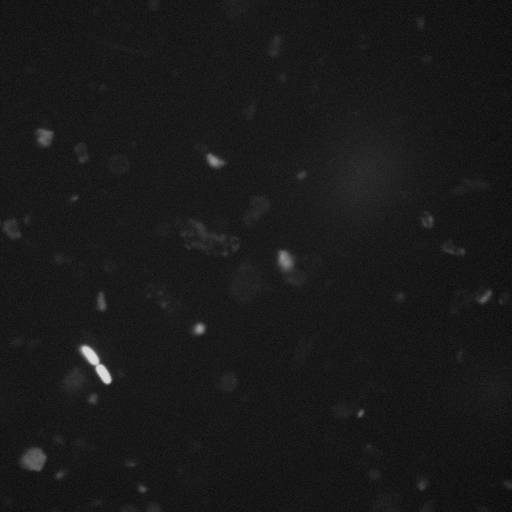

Supplement: S4 File — (ZIP) [file pcbi.1006986.s005.zip › extraitseq4h/KM16_016_4h_Cm_CCCP_13_w2sdcGFP.tif]

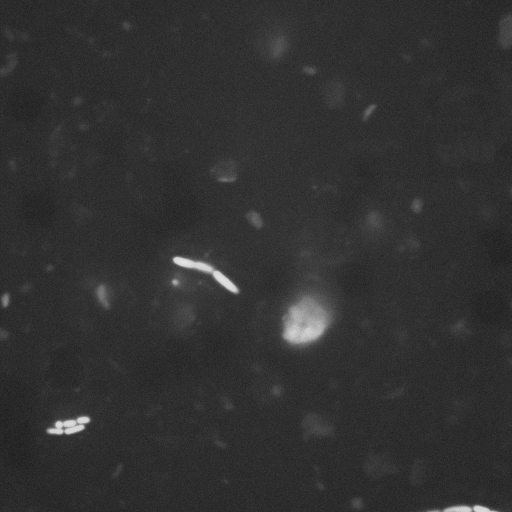

Supplement: S4 File — (ZIP) [file pcbi.1006986.s005.zip › extraitseq4h/KM16_013_4h_27_w2sdcGFP.tif]

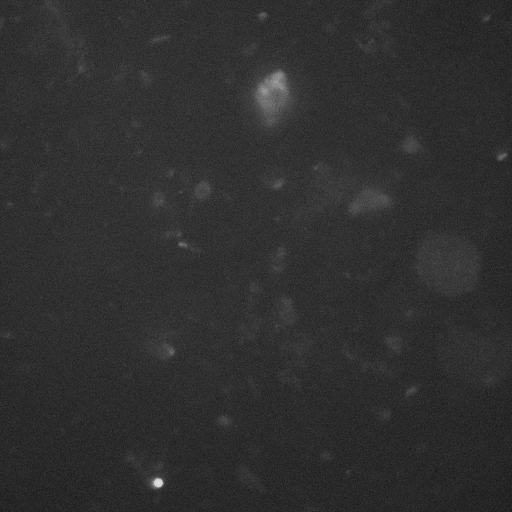

Supplement: S4 File — (ZIP) [file pcbi.1006986.s005.zip › extraitseq4h/KM16_013_4h_18_w1sdcRFP.tif]

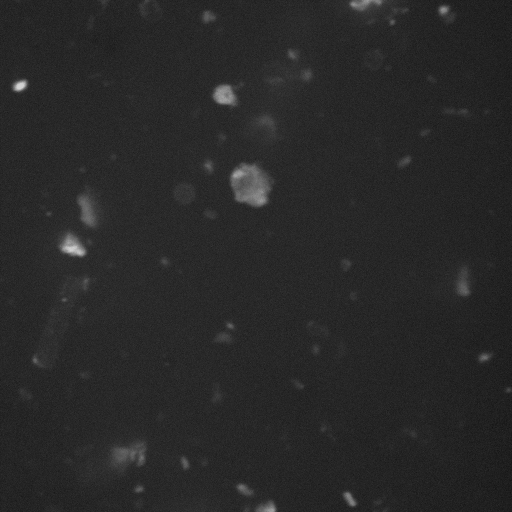

Supplement: S4 File — (ZIP) [file pcbi.1006986.s005.zip › extraitseq4h/KM16_016_4h_PBS_2_w2sdcGFP.tif]

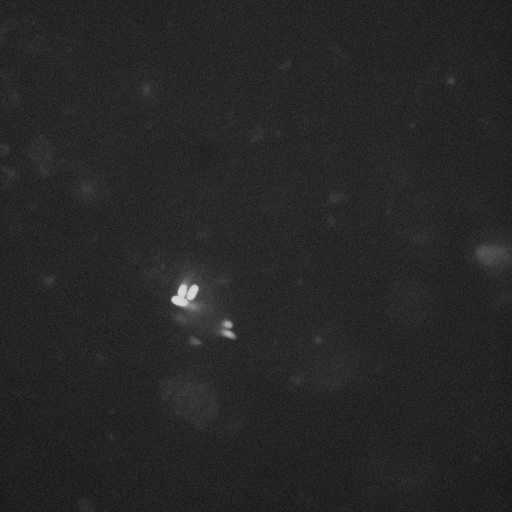

Supplement: S4 File — (ZIP) [file pcbi.1006986.s005.zip › extraitseq4h/KM16_013_4h_13_w1sdcRFP.tif]

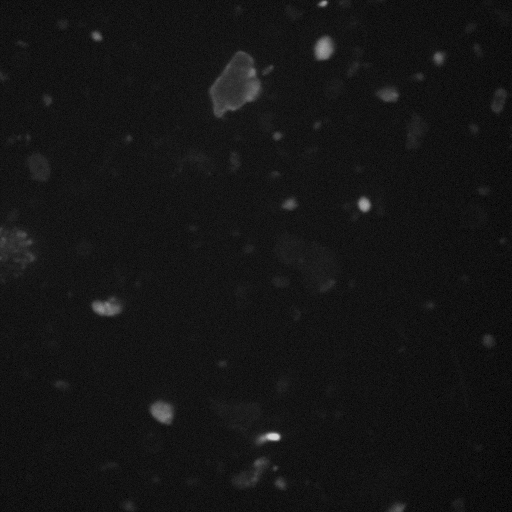

Supplement: S4 File — (ZIP) [file pcbi.1006986.s005.zip › extraitseq4h/KM16_016_4h_Cm_CCCP_18_w2sdcGFP.tif]

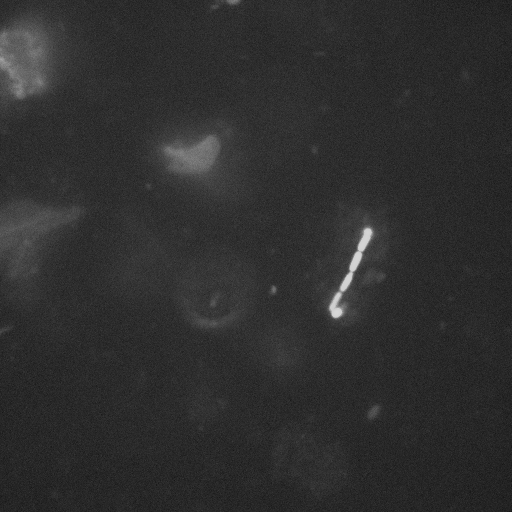

Supplement: S4 File — (ZIP) [file pcbi.1006986.s005.zip › extraitseq4h/KM16_013_4h_25_w1sdcRFP.tif]

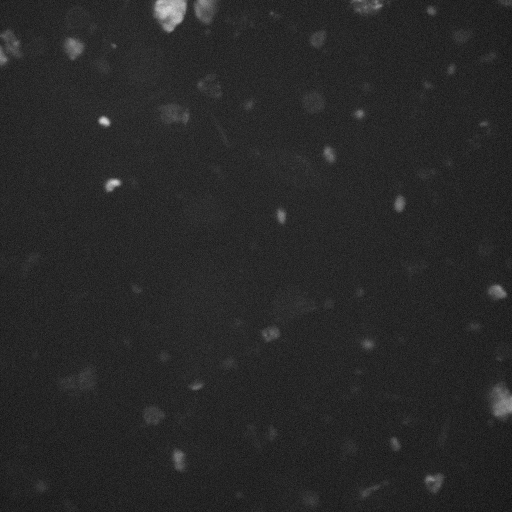

Supplement: S4 File — (ZIP) [file pcbi.1006986.s005.zip › extraitseq4h/KM16_016_4h_PBS_11_w1sdcRFP.tif]

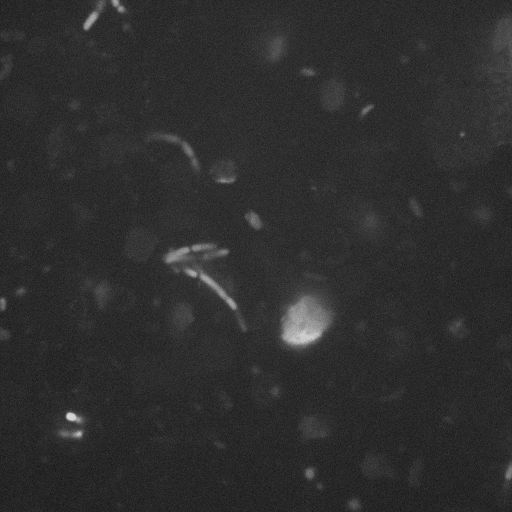

Supplement: S4 File — (ZIP) [file pcbi.1006986.s005.zip › extraitseq4h/KM16_013_4h_27_w1sdcRFP.tif]

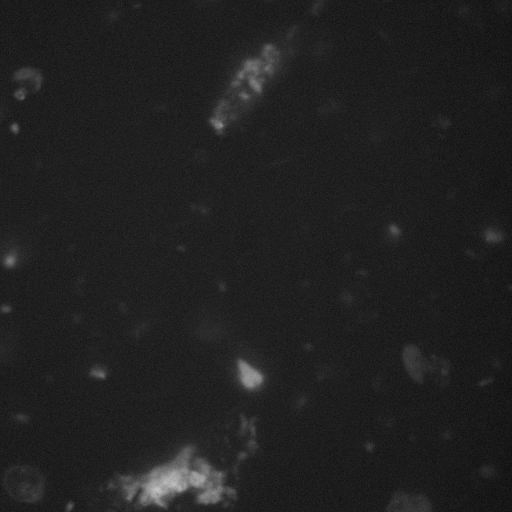

Supplement: S4 File — (ZIP) [file pcbi.1006986.s005.zip › extraitseq4h/KM16_016_4h_Cm_CCCP_17_w2sdcGFP.tif]

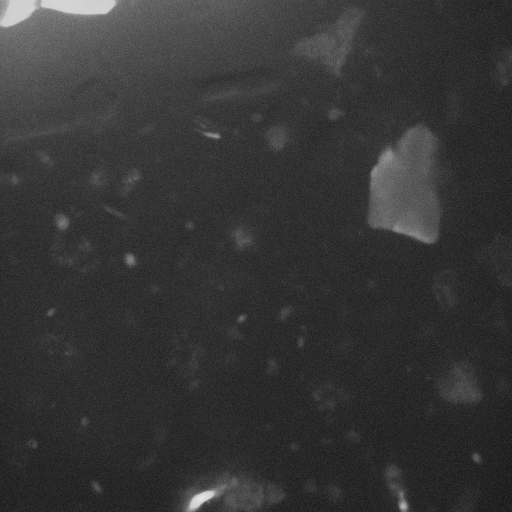

Supplement: S4 File — (ZIP) [file pcbi.1006986.s005.zip › extraitseq4h/KM16_013_4h_7_w1sdcRFP.tif]

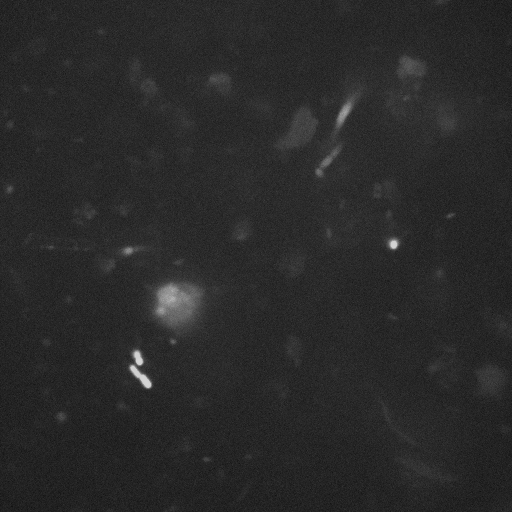

Supplement: S4 File — (ZIP) [file pcbi.1006986.s005.zip › extraitseq4h/KM16_013_4h_22_w1sdcRFP.tif]

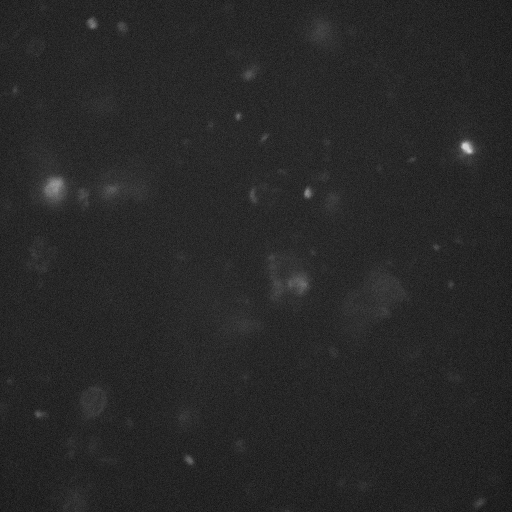

Supplement: S4 File — (ZIP) [file pcbi.1006986.s005.zip › extraitseq4h/KM16_016_4h_Cm_CCCP_20_w1sdcRFP.tif]

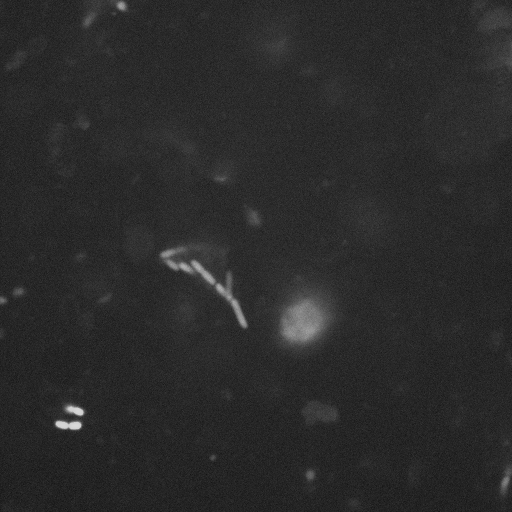

Supplement: S4 File — (ZIP) [file pcbi.1006986.s005.zip › extraitseq4h/KM16_013_4h_26_w1sdcRFP.tif]

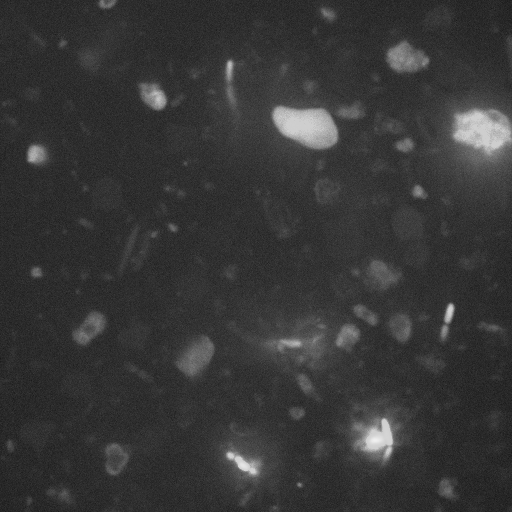

Supplement: S4 File — (ZIP) [file pcbi.1006986.s005.zip › extraitseq4h/KM16_013_4h_20_w1sdcRFP.tif]

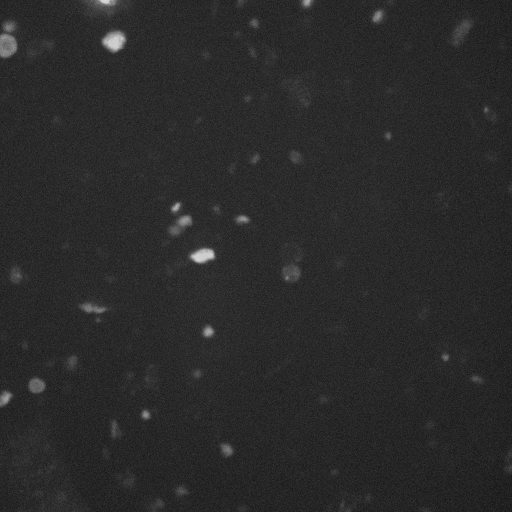

Supplement: S4 File — (ZIP) [file pcbi.1006986.s005.zip › extraitseq4h/KM16_016_4h_PBS_16_w1sdcRFP.tif]
