# Supplementary figures and images for: Enchained growth and cluster dislocation: A possible mechanism for microbiota homeostasis (part 10 of 10)
Source: PLoS Comput Biol. 2019 May 3;15(5):e1006986. doi: 10.1371/journal.pcbi.1006986 (PMC6519844; doi:10.1371/journal.pcbi.1006986)

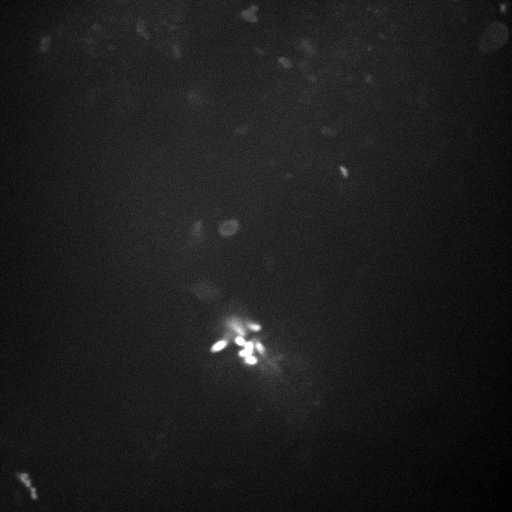

Supplement: S5 File — (ZIP) [file pcbi.1006986.s006.zip › extraitseq5h/KM16_014_5h_21_w2sdcGFP.tif]

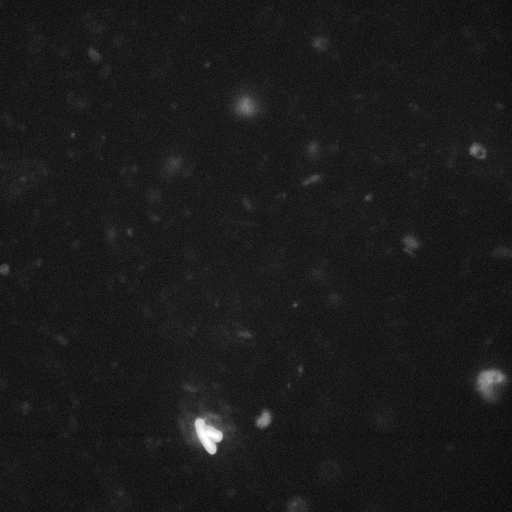

Supplement: S5 File — (ZIP) [file pcbi.1006986.s006.zip › extraitseq5h/KM16_014_5h_19_w1sdcRFP.tif]

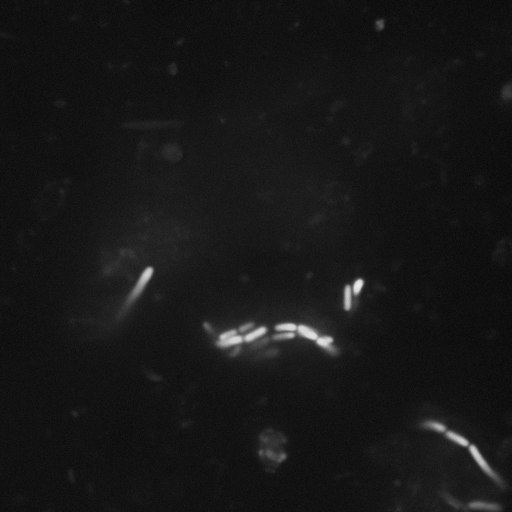

Supplement: S5 File — (ZIP) [file pcbi.1006986.s006.zip › extraitseq5h/KM16_013_5h_13_w2sdcGFP.tif]

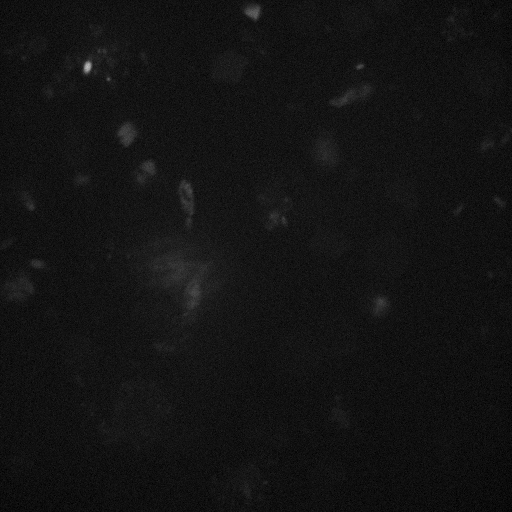

Supplement: S5 File — (ZIP) [file pcbi.1006986.s006.zip › extraitseq5h/KM16_013_5h_1_w1sdcRFP.tif]

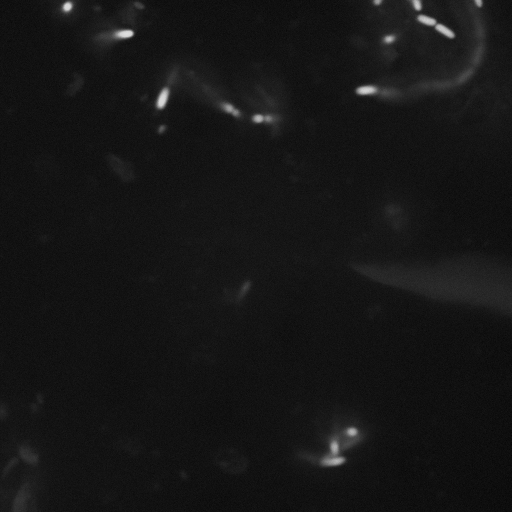

Supplement: S5 File — (ZIP) [file pcbi.1006986.s006.zip › extraitseq5h/KM16_013_5h_22_w2sdcGFP.tif]

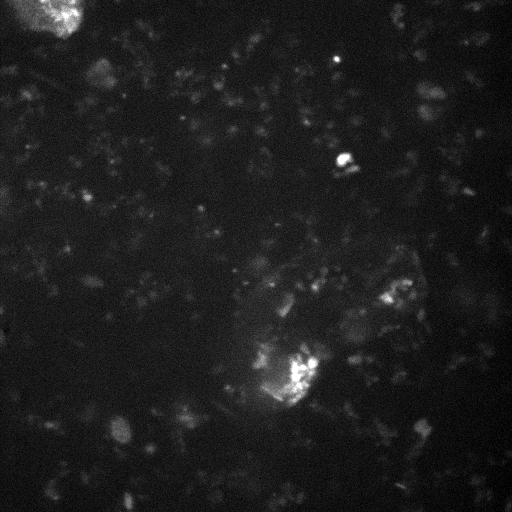

Supplement: S5 File — (ZIP) [file pcbi.1006986.s006.zip › extraitseq5h/KM16_014_5h_16_w2sdcGFP.tif]

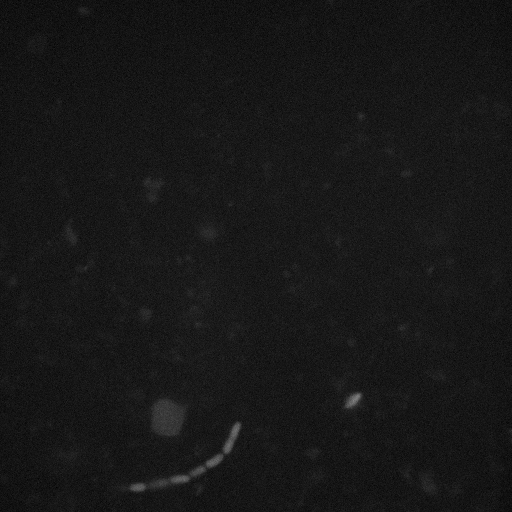

Supplement: S5 File — (ZIP) [file pcbi.1006986.s006.zip › extraitseq5h/KM16_014_5h_9_w1sdcRFP.tif]

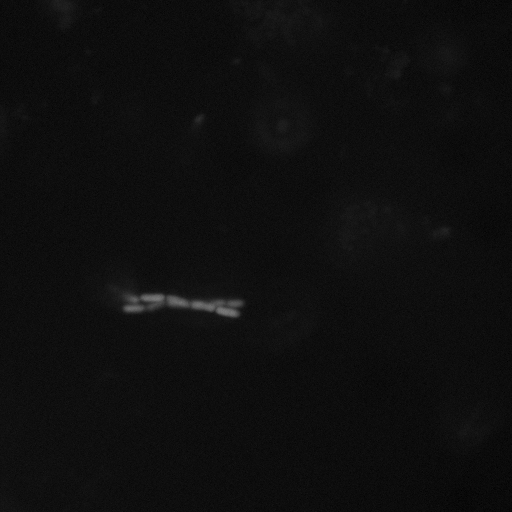

Supplement: S5 File — (ZIP) [file pcbi.1006986.s006.zip › extraitseq5h/KM16_013_5h_10_w2sdcGFP.tif]

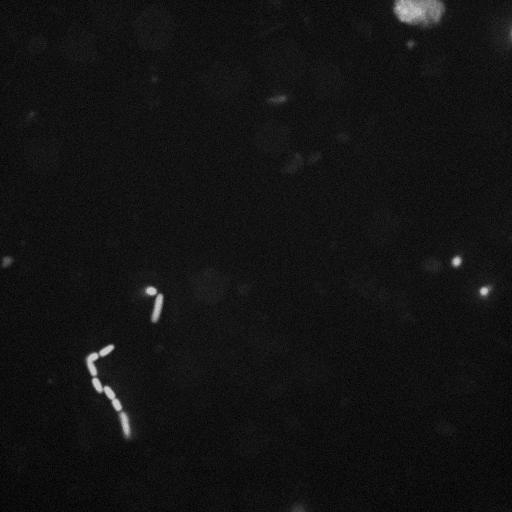

Supplement: S5 File — (ZIP) [file pcbi.1006986.s006.zip › extraitseq5h/KM16_013_5h_23_w1sdcRFP.tif]

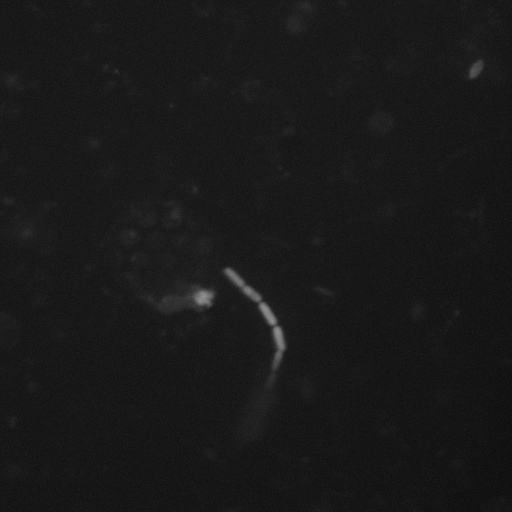

Supplement: S5 File — (ZIP) [file pcbi.1006986.s006.zip › extraitseq5h/KM16_014_5h_4_w2sdcGFP.tif]

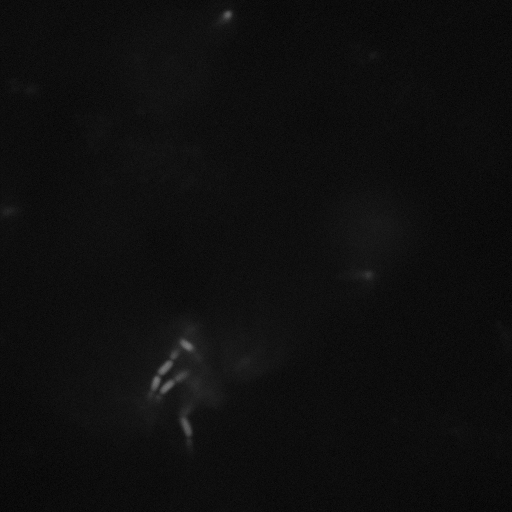

Supplement: S5 File — (ZIP) [file pcbi.1006986.s006.zip › extraitseq5h/KM16_013_5h_26_w2sdcGFP.tif]

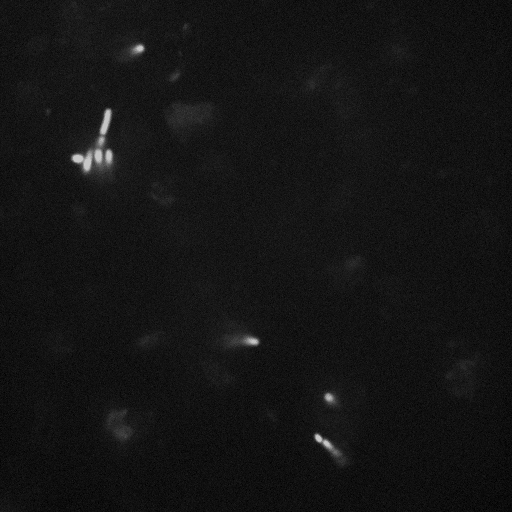

Supplement: S5 File — (ZIP) [file pcbi.1006986.s006.zip › extraitseq5h/KM16_013_5h_25_w1sdcRFP.tif]
